# Organophotoredox/palladium dual catalytic decarboxylative Csp^3^–Csp^3^ coupling of carboxylic acids and π-electrophiles†‡

**DOI:** 10.1039/d0sc02609c

**Published:** 2020-07-16

**Authors:** Kaitie C. Cartwright, Jon A. Tunge

**Affiliations:** Department of Chemistry, The University of Kansas 1567 Irving Rd. Lawrence KS 66045 USA tunge@ku.edu

## Abstract

A dual catalytic decarboxylative allylation and benzylation method for the construction of new C(sp^3^)–C(sp^3^) bonds between readily available carboxylic acids and functionally diverse carbonate electrophiles has been developed. The new process is mild, operationally simple, and has greatly improved upon the efficiency and generality of previous methodology. In addition, new insights into the reaction mechanism have been realized and provide further understanding of the harnessed reactivity.

## Abstract 2

A dual catalytic decarboxylative allylation and benzylation method for the construction of new C(sp^3^)–C(sp^3^) bonds between readily available carboxylic acids and functionally diverse carbonate electrophiles has been developed.

## Graphical abstract

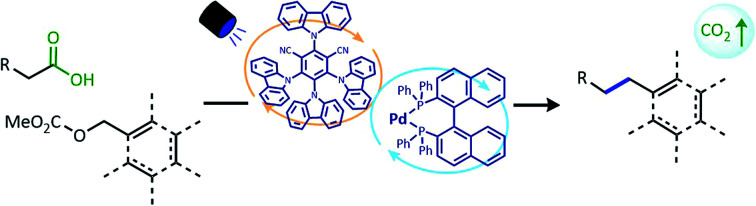

## Introduction

Photoredox catalysis has emerged as a powerful strategy in the functionalization of feed-stock carboxylic acids.^1^ Using the carboxylic acid as a traceless activating group has allowed for the site-specific generation of radical species under mild conditions, while producing carbon dioxide as the only stoichiometric byproduct. Moreover, as compared to traditional anionic decarboxylation, radical decarboxylation often provides a facile and more generally applicable pathway for activation.^2^

One reaction that has benefited from the employment of photoredox-promoted decarboxylation is decarboxylative allylation (DcA). This reaction is a subset of the Tsuji–Trost allylation that distinguishes itself by the use of carboxylic acids as latent carbanions, bypassing the need for pre-formed organometallics or strong alkaline conditions.^3^ Despite being an attractive and powerful transformation, anionic decarboxylation is limited to carboxylic acid substrates which provide sufficient stabilization of the resulting carbanion following the decarboxylation event. A p*K*_a_ of less than 25 is often required for facile decarboxylation, and substrates with p*K*_a_ values between 25 and 30 demand elevated temperatures.^3*a*,4^

**Scheme 1:**
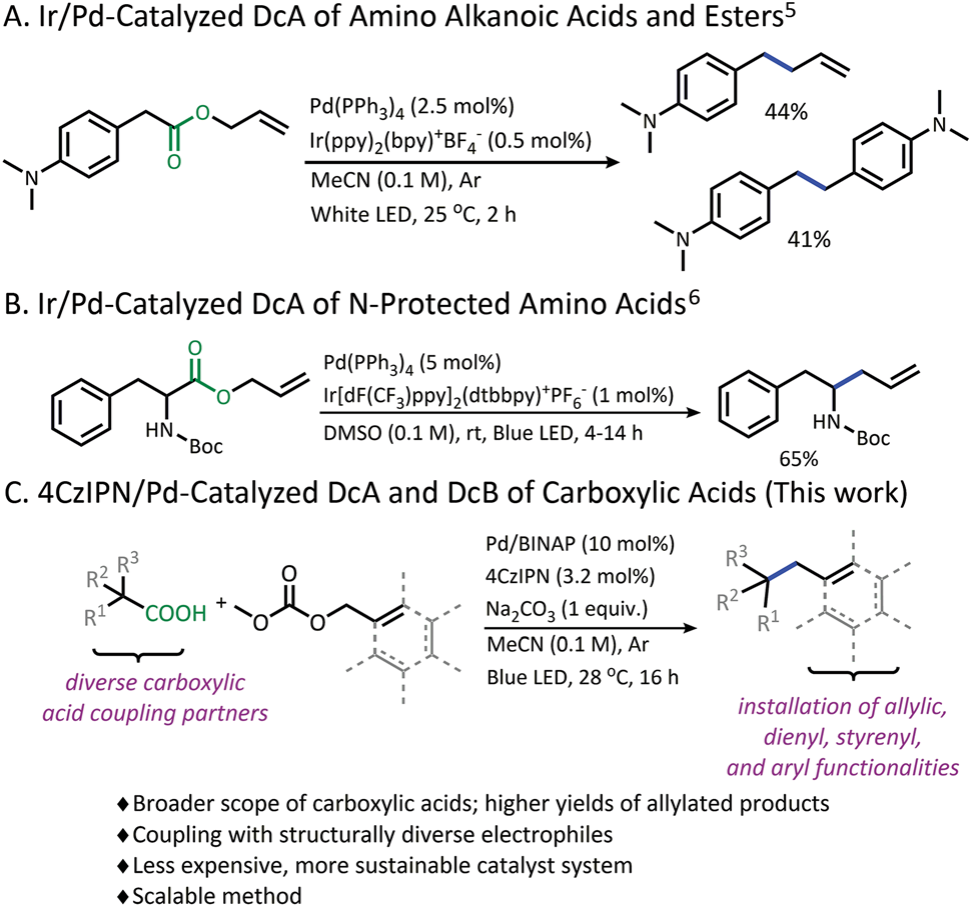
Tunge dual catalytic decarboxylative couplings.

Recently, our lab has developed methodology that overcomes this limitation in p*K*_a_ through the development of a photoredox/Pd dual catalytic coupling strategy (Schemes 1A and B).^5,6^ In this system, an oxidative radical decarboxylation is facilitated by an iridium photosensitizer, which allows for a facile radical decarboxylation. Inverting the electronic demand for the decarboxylation event allows access to carboxylic acid nucleophiles that fall outside the p*K*_a_ requirement for thermal decarboxylation. Once the carbon radical species is generated, combination with an electrophilic Pd-π-allyl intermediate provides a new C(sp^3^)–C(sp^3^) bond.^7^

Although a great advancement in DcA, this chemistry remains underdeveloped. Specifically, the reaction was only demonstrated on activated carboxylic acids that contain α-amino^5,6^ and/or α-benzylic^5^ functionalities which provide significant stabilization to the radical intermediate. Within these substrate classes, the α-amino precursors tend to provide higher yields than benzylic radical precursors due to an undesired homocoupling observed between benzylic radicals (Scheme 1A).^5,6^ Allylation of carboxylic acids without these radical-stabilizing functionalities has remained elusive. Additionally, the desire to utilize this reactivity with more complex electrophilic coupling partners has not been realized. In fact, the original method was only operable with a narrow range of 2-substituted allyl electrophiles, while the more challenging 3-substituted allyl electrophiles were not effectively coupled.^5,6,8^ Finally, the methods reported thus far utilized iridium-based photosensitizers which are ultimately expensive and unsustainable.^9^

Herein, a new organophotoredox/palladium dual catalytic process is described that has greatly improved the utility of this technology through (1) achieving higher yields and greater generality in carboxylic acid nucleophiles, and (2) allowing access to a variety of structurally diverse allylic electrophiles for the installation of various alkenyl, styrenyl, dienyl, and aryl functionalities (Scheme 1C). In addition, evaluating this methodology has provided new insights into the advantages and limitations of radical DcA, as well as the dominant catalytic pathway.

## Results and discussion

**Fig. 1:**
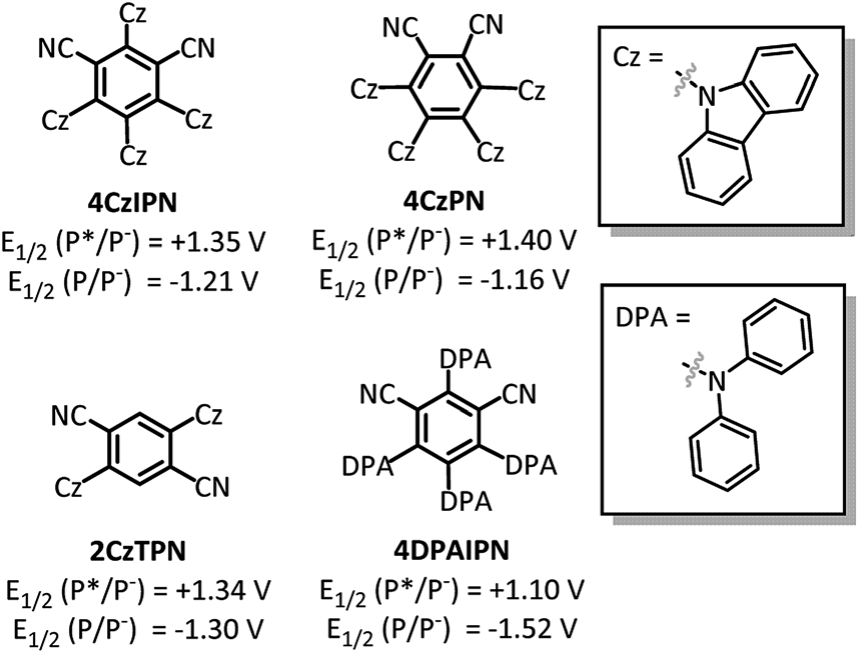
Dicyanobenzene carbazole-based fluorophores.

With the goal of developing a broadly applicable catalyst system for decarboxylative allylation of alkanes, a complete re-evaluation of the catalyst system was undertaken. In 2016, Zhang reported a set of carbazole-based donor–acceptor fluorophores that are inexpensive and easily accessible organic photoredox catalysts.^10^ Further, this group of catalysts possesses a range of redox potentials resembling that of the iridium photosensitizers originally employed (Fig. 1, [Ir(ppy)_2_(bpy)]^+^ BF_4_^−^: P*/*P*^−^ = +0.95 V & *P*/*P*^−^ = −1.05 V; [Ir[dF(CF_3_)ppy]_2_(dtbbpy)]^+^ PF_6_^−^: P*/*P*^−^ = +1.21 V & *P*/*P*^−^ = −1.37 V). Employing the carbazole-based photosensitizers (Fig. 1) in the photoredox-facilitated DcA revealed the **4CzIPN** catalyst to be the most compatible with the transformation (Table S1†).^10*a*,11^ The only other catalyst to provide notable conversion in the DcA was **4CzPN**, but produced less allylated product than **4CzIPN** (Table S1†).

**Scheme 2:**
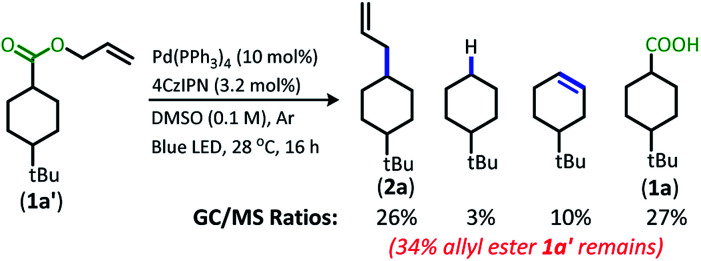
Previous DcA conditions with **4CzIPN** photosensitizer (GC/MS ratios list represent area percent out of 100% of all products and starting materials present in the final reaction mixture).

The change from Ir to **4CzIPN** was first adopted into reaction conditions that were found to be successful in our previous dual catalytic DcA (Scheme 2).^5,6,12^ This reaction produced the allylated product, but also produced an alkane and alkene side product. Additionally, this reaction did not proceed to high conversion.

In order to maximize production of the allylated product, the influence of changing the Pd ligands was investigated (Table 1, see Table S2† for full ligand screening and Table S3† for Pd pre-catalyst evaluation). The change in ligand proved to have a significant influence on the success of the reaction. Change to the ligand resulted in either (1) low conversion, (2) high conversion, but little decarboxylation, (3) the alkene/alkane side products were formed in large quantity, or (4) the allylated product was the major product.

**Table 1:** Ligand evaluation

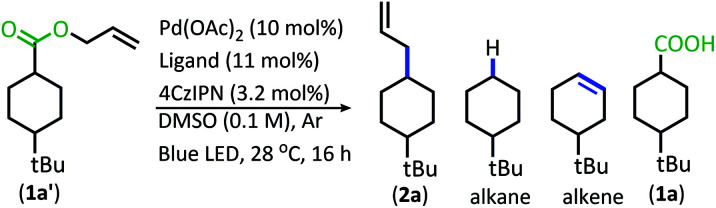						
Entry^a^	Ligand	(**2a**)	Alkane	Alkene	(**1a′**)	(**1a**)
1^b^	BINAP	82	9	9	0	0
2	(*R*)-C_3_-TUNEPHOS	78	8	14	0	0
3	(*R*)-SEGPHOS	76	9	15	0	0
4	(*S*)–SYNPHOS	75	8	17	0	0
5	(*R*)-Tol-BINAP	73	8	19	0	0
6	DTMB SegPhos	70	8	22	0	0

a All reactions were performed on 0.2 mmol scale and product ratios were determined by GC/MS (GC/MS ratios list represent area percent out of 100% of all products and starting materials present in the final reaction mixture).

b **2a** formed as a 60 : 40 *trans* : *cis* mixture.

The ligands that allowed the reaction to proceed with full conversion, complete decarboxylation, and produced the allylated product as the major product consisted of bidentate phosphine ligands with similar molecular scaffolds (Table 1, entries 1–6). Of these catalysts, the BINAP and SegPhos ligands provided a higher percentage of allylated product 2a (Table 1, entries 1 & 3) as compared to their more sterically demanding counterparts, Tol-BINAP and DTBM-SegPhos (Table 1, entries 5 & 6). Gratifyingly, BINAP proved to be the superior ligand and is also one of the least expensive and most readily accessible of the ligand set (Table 1, entry 1).

**Scheme 3:**
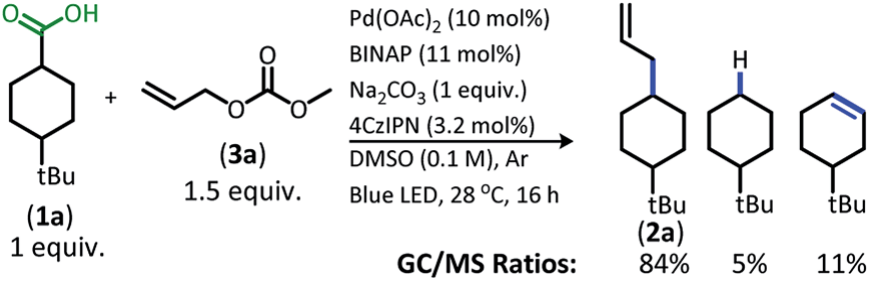
Intermolecular DcA of **1a** (GC/MS ratios list represent area percent out of 100% of all products and starting materials present in the final reaction mixture).

To improve upon the reaction economy and operational simplicity, an intermolecular process that directly utilizes a carboxylic acid for reaction with various allylic carbonates was devised (Table S5†). To our delight, the intermolecular reaction proceeded well. Allyl methyl carbonate was identified as the ideal allylic electrophile and sodium carbonate proved to be the ideal base (Scheme 3). Lastly, the reaction was found to produce comparable results in acetonitrile (73% yield **2a**, 60 : 40 d.r.), which was preferred over the use of dimethyl sulfoxide due to the more straightforward isolation of the allylated product.^13^

With superior decarboxylative allylation conditions established, attention was turned to the allylation of a variety of carboxylic acids (Table 2). Initially, the allylation was performed with *N*-protected amino acids (**2b–q**). No alkene or alkane side products were observed with these substrates. Gratifyingly, the amino acid substrates that underwent allylation with our first-generation method were able to be allylated in similar to increased yields under our new conditions.

**Table 2:** Products from DcA of carboxylic acids^a^^,^^b^^,^^c^

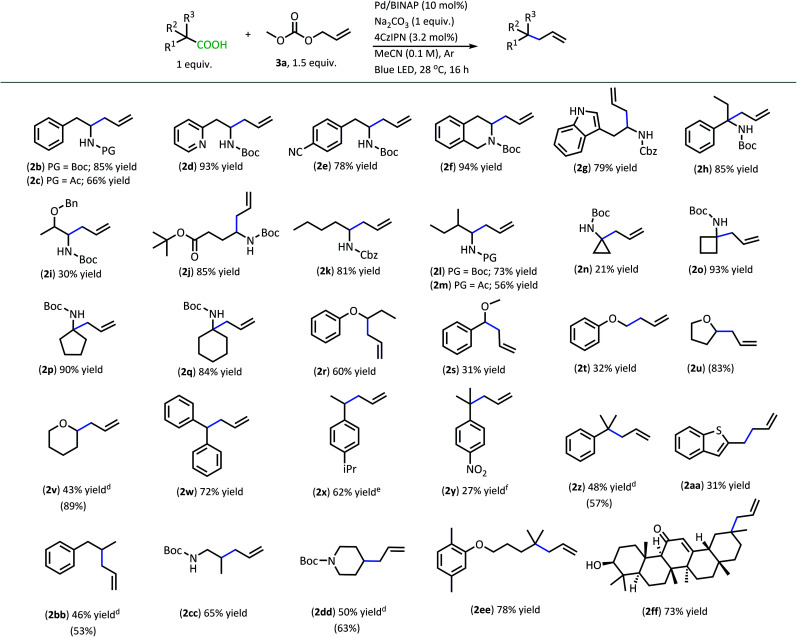

a Reactions were performed on 0.2 mmol scale.

b Isolated yields are shown.

c (Yields) were determined by q^1^H NMR with pyridine as the internal standard for products that were found to be volatile.

d Yields are an average of two reactions.

e 95% pure, see ESI for details.

f Product was isolated with alkane side product and mass adjusted; product previously used in thermal DcA.^17^

Interestingly, under these conditions, carbamate-protected amino acids produced higher yields than their acyl-protected counterparts, in contrast to first-generation conditions that resulted in comparable yields for both groups (**2b–c**, **2l–m**).^6^ The *N*-Boc- and *N*-Cbz-protected amino acid substrates generally performed well with a variety of different side chain functionalities. Substrates containing N-heterocycle side chains proceeded smoothly, allowing for high yields of the allylated alkaloids **2d** and **2g** (93% & 79%, respectively).^14^ Increased steric demand around the carboxylic acid did not interfere with the efficiency of DcA (**2h** & **2l**). Cyclic scaffolds also generally underwent DcA well (**2n–q**). Interestingly, the cyclopropane amino acid substrate **2n** was allylated in low yield (21%) but larger ring sizes such as a cyclobutane (**2o**), cyclopentane (**2p**), and cyclohexane (**2q**) were allylated in high yields.^15^ The DcA of **1b** was successfully run in batch on the 1 mmol scale, which provided **2b** in 93% yield (see ESI† for details).

In addition to the successful decarboxylative allylation of amino acids, other carboxylic acids that produce more reactive radical intermediates were allylated with this method (**2r–2ff**). Substrates that produced monosubstituted and unsubstituted α-oxy radicals were generally allylated in moderate yields (**2r–v**). The alkene and alkane side products do arise in the DcA of these substrates. Benzylic carboxylic acid substrates that provide 3°, 2°, and 1° benzylic radicals upon decarboxylation could be allylated in moderate to good yields (**2w–2aa**). Of these, the substrates **1w** and **1x** that produce 2° benzylic radicals provided yields exceeding 60% (**2w** & **2x**). This is notable since the diphenylmethane nucleophile has previously been employed in anionic allylation under thermal conditions, but the yield of allylated product is much less (10% yield) than what can be obtained in the **4CzIPN**/Pd DcA conditions.^16^ The homodimeric products frequently observed under the Ir/Pd conditions with benzylic carboxylic acids were not observed with the disubstituted benzylic acids allylated under the new conditions. Instead, alkene and/or alkane side products were observed. Conversely, benzylic acid substrate (**1aa**) was able to be allylated in 31% yield (**2aa**) but did produce the homocoupled product in 48% yield.

**Scheme 4:**
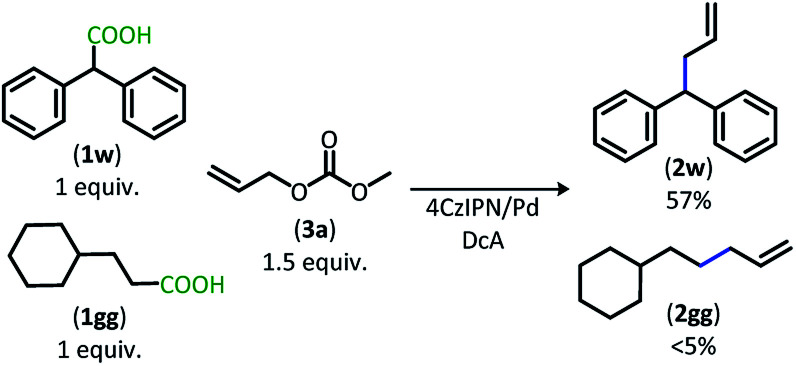
Preferential DcA of disubstituted carboxylic acid.

The dual catalytic DcA was also applicable to carboxylic acids that possess weak radical-stabilizing functionalities (**2a**, **2bb–2ff**). The highest yields of allylated products, 70–80%, were observed with quaternary carboxylic acids such as gemfibrozil (**1ee**) and 18-β-glycyrrhetinic acid (**1ff**). The tertiary carboxylic acids of this group such as β-amino acid (**1cc**) and γ-amino acid (**1dd**), were allylated in moderate yields (65% **2cc** & 50% **2dd**). Unfortunately, unsubstituted acids that do not possess functionalities to provide moderate stabilization of the radical intermediate underwent poor conversion and only produced trace amounts of allylated product. To demonstrate the difference in reactivity, a disubstituted acid (**1w**) was allylated selectively in the presence of an unsubstituted acid under standard reaction conditions (Scheme 4).^18^ Substrate **1gg** remained intact during this reaction.^19^

While substrates that contain strong radical-stabilizing groups such as the α-amino acids only produced the allylated product, more reactive radicals resulted in the formation of the alkene and alkane side products as was observed in the optimization of DcA of **1a’** and **1a**. Potentially, these products form as a result of off-cycle pathways such as disproportionation or β-hydride elimination.^20^

**Scheme 5:**
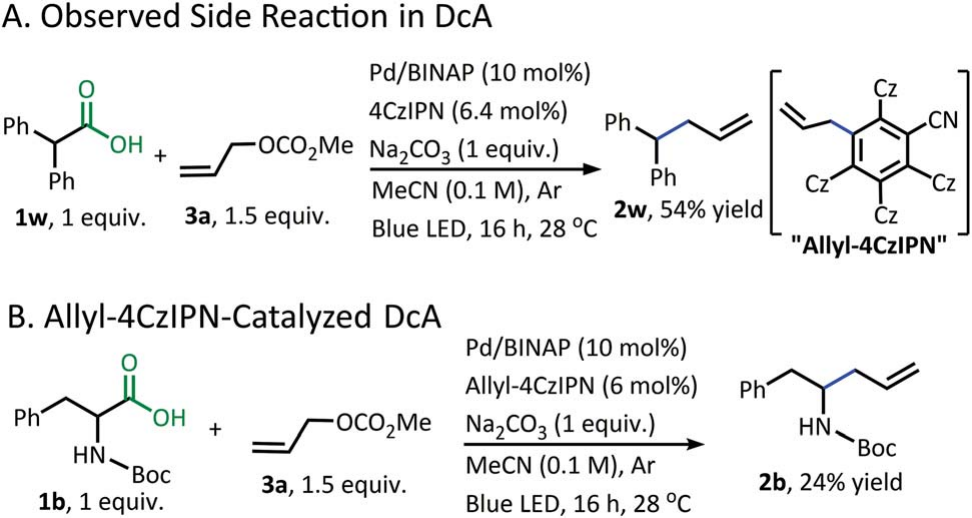
Allylation of **4CzIPN**.

In addition to the alkane- and alkene-forming side reactions, a recent report by König brought to our attention the possibility of competitive radical addition to the **4CzIPN** photocatalyst.^21^ Sure enough, the “Allyl-**4CzIPN**” was isolated along with intact **4CzIPN** after the catalytic DcA of **1w** (Scheme 5A).^22^ When the “Allyl-**4CzIPN**” was utilized as the photocatalyst in the DcA of **1b**, a greatly diminished yield results (Scheme 5B). Thus, the side reaction with **4CzIPN** ultimately degrades the photocatalyst activity.

Apart from the improved accessibility of carboxylic acid nucleophiles, another compelling aspect of this methodology is its efficient coupling of carboxylic acids with more functionally diverse π-electrophiles (Table 3). Allylic carbonates with substituents in the 2-position proceeded well, with the electron-withdrawing phenyl substituent producing higher yield (**4c**, 82%) compared with 2-methyl allyl carbonate (**4b**, 64%). Having Cl in the 2-position unfortunately resulted in a poor yield (**4d**, 14%) and formation of a plethora of side products. This may be due to poor chemoselectivity between allyl carbonate and vinyl halide electrophiles.

**Table 3:** Products from DcA/DcB of **1f**^a^^,^^b^^,^^c^

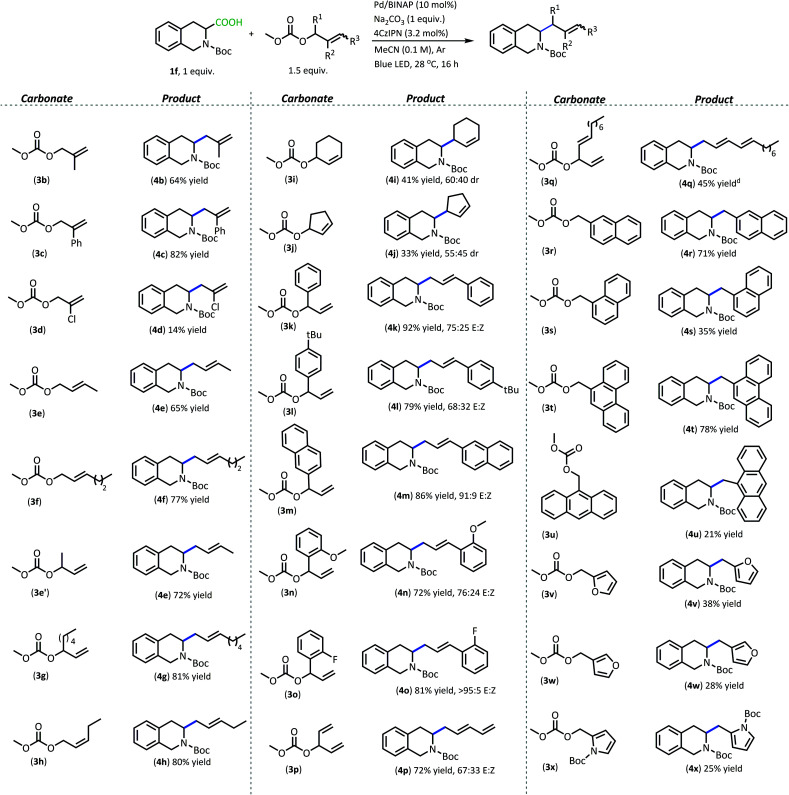

a Reactions were performed on 0.2 mmol scale.

b Isolated yields are shown.

c Isomer ratios were determined by ^1^H NMR.

d Mix of *E*/*Z*-isomers.

1- and 3-substituted allylic carbonates having a variety of carbon chains also operate well in the radical DcA (**4e–4h**). These carbonates functioned comparably regardless of the geometric configuration of the alkene or the structure of the allylic carbonate (branched/linear). They also produced the *E*-configured olefin and the linear product despite the variation in the carbonate starting material. Cyclic allylic carbonates also provided allylated products, however the yields were about half what is realized with their acyclic counterparts (**4i–4j**).

Utilizing branched acyclic allylic carbonates was quite advantageous (*e.g.***3e′***vs.***3e**).^23^ For one, they can be synthesized from aldehydes, providing a plethora of inexpensive options for the electrophilic coupling partner.^24^ Also, utilizing branched carbonates provided cleaner reactions than their 3-substituted allyl carbonate counterparts in some cases. For instance, using carbonates derived from cinnamyl alcohols produced a complex mixture of products under these reaction conditions and provided poor yields of allylated product. In contrast, switching to the branched carbonates allowed for the formation of styrenyl products in good yields (**4k–4o**, 70–95% yield). Potentially, this reaction benefits from a more facile oxidative addition that is achieved with the branched carbonates as opposed to the cinnamyl carbonates. The conjugated olefin products are produced as a mixture of geometric isomers which is likely the result of a background photoisomerization.^24^ Here, the styrenyl products were obtained in higher yields and with greater *E*-selectivity when the aryl moiety contained electron-withdrawing functionalities (**4m** & **4o**, 86% & 81% yield, respectively, >90 : 10 *E* : *Z*) as opposed to electron-donating functionalities (**4l** & **4n**, 79% & 72% yield, respectively, ∼70 : 30 *E* : *Z*). The branched carbonates also proved highly useful for the installation of other conjugated functionalities such as dienes (**4p** & **4q**, 72% & 45% yield, respectively), but these too provided a mix of geometric isomers.

Apart from DcA, decarboxylative benzylation (DcB) with Pd-π-benzyl electrophiles would be an attractive application of dual catalytic couplings.^25^ Through the use of benzylic carbonates, toxic benzylic halides that are traditionally employed in cross-coupling could be avoided.^26^ However, Pd-catalyzed DcB is challenging since aromatic moieties receive additional stability from conjugation, making the barrier for oxidative addition higher.^27^ Therefore, benzylic carbonates with extended conjugation are generally utilized because their lower resonance energies lead to lower activation barriers.^27,28^

Benzylic carbonates with extended conjugation underwent high conversion under our mild reaction conditions. The highest yielding benzylations are those that install naphthalene (**4r**, 71% yield) and phenanthrene tethers (**4t**, 78% yield). The benzylic carbonates react to full conversion to produce the cross-coupled product and the mass-balance is typically made up of the benzyl dimer.

The regiochemistry of the alkylcarbonate group does influence the reaction yield. With the naphthalene system, the higher yield is obtained when the naphthalene is substituted in the 2-position (**3r**) *vs.* the 1-position (**3s**) (**4r** & **4s** isolated in 71% & 35% yield, respectively). The reaction with carbonate (**3s**) resulted in 46% yield of naphthalene dimer. Similarly, the 9-anthracenyl carbonate (**3u**) provides the cross-coupled product (**4u**) in just 21% yield due to favorable formation of the homocoupled bianthracenyl dimer.

Heteroaromatic systems also underwent successful cross-coupling, but with lower yields.^14^ Compared to the aromatic hydrocarbon systems, the heteroaromatic benzylic carbonates produced a variety of oligomer side products. Nevertheless, these carbonates have been utilized to provide the cross-coupled product between **1f** and furyl carbonates as well as with pyrrolyl carbonate, with yields between 20–40% (**4v–x**). Like the naphthalene system, the regiochemistry of the carbonate leaving group influences the yield of the reaction. With the furyl carbonate, the 2-alkyl carbonate (**3v**) provided higher yields than the analogous 3-substitution (**3w**). An *N*-Boc pyrrole group was also able to be installed through this coupling, albeit with a low isolated yield that is reminiscent of what was observed with the related furyl carbonates (**4x**).

After exploration of scope and reactivity, mechanistic probes were utilized to shed light on the dominant catalytic cycle. First, it is important to note that the reaction does not proceed (<5% yield) without the Pd, photocatalyst, or light, indicating that this process relies on a light-promoted dual catalytic system (Table S6†).

**Scheme 6:**
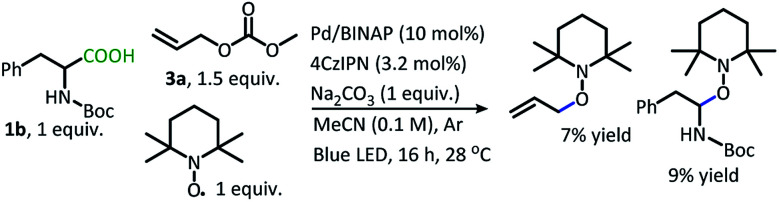
DcA in the presence of TEMPO.

When the radical trap, TEMPO, is incorporated into the reaction, the allylated product is not produced (Scheme 6). Instead, conversion is interrupted, and low yields of TEMPO trapped products result. This result supports the formation of radicals along the reaction pathway.

A Stern–Volmer analysis was completed to assess the photocatalytic processes taking place. The potential fluorescence emission quenching of **4CzIPN** with diphenylcarboxylate tetrabutylammonium salt, as well as with a variety of Pd species, was investigated (see ESI† for experimental details). Quenching of **4CzIPN** with diphenylcarboxylate tetrabutylammonium salt was observed, which was not surprising since **4CzIPN** (+1.35 V)^10^ is sufficiently reactive to oxidize the carboxylate (+1.0 to +1.3 V).^18^ However, the observed **4CzIPN** quenching with the carboxylate salt was significantly less efficient (Fig. S2†) than the observed quenching with relevant Pd species investigated.

The first Pd species investigated was the Pd(OAc)_2_/BINAP pre-catalyst. When these reactions were assembled, an initial stirring of the Pd(OAc)_2_ pre-catalyst and BINAP was performed to allow for reduction of the Pd(ii) to Pd(0).^29^ However, this process was not anticipated to fully reduce the Pd(ii) species and thus higher oxidation states of Pd are expected to be present at the onset of the reaction.^29^ The mixture of Pd(OAc)_2_/BINAP was found to be an efficient quencher of **4CzIPN** (∼10-fold more than carboxylate, Fig. S3†). Similar Pd species are reported to have reduction potentials between −1.1 and −1.3 V, which places the potentials for the Pd catalyst reduction close to the excited state oxidation potential of **4CzIPN** (−1.04 V).^10,30–32^ If such a reduction initiates the cycle, the resulting **4CzIPN** radical cation (+1.52 V)^10^ should easily oxidize carboxylates (+1.0 to +1.3 V)^17^ in the reaction, thus turning over the catalyst.

Alternatively, it is possible that the dominant catalytic pathway proceeds *via* oxidative addition of Pd(0) and allyl carbonate to provide the cationic π-allyl-Pd. This species is anticipated to be highly relevant to the mechanistic pathway, thus its ability to quench **4CzIPN** was also considered. After an evaluation of reported redox potentials, the cationic π-allyl-Pd is not expected to be reduced by **4CzIPN** as the potential reported for this species is −1.35 V, which is not a favorable match for oxidative quenching of **4CzIPN** (−1.04 V).^10,33^ In agreement with this redox potential mismatch, a π-allyl-PdCl dimer/BINAP mixture did not quench **4CzIPN** (Fig. S4†). However, π-allyl-Pd(OAc)/BINAP was found to be an efficient quencher of **4CzIPN** (∼10-fold more efficient than carboxylate, Fig. S4†). The high quenching ability of the BINAP complex of π-allyl-Pd(OAc) as opposed to π-allyl-PdCl points to a carboxylate oxidation pathway reminiscent of other metal-carboxylate species.^34^ Furthermore, the higher quenching constant for the π-allyl-PdOAc complex, as compared to tetrabutylammonium carboxylate, indicates the key role of the Pd complex in facilitating quenching.

After the Pd-facilitated quenching and decarboxylation events, there are several possibilities for the C–C bond formation. First, the possibility of a radical-polar cross-over event must be considered.^21,35^ But, due to the large negative reduction potentials of most alkyl radicals utilized herein (<−1.3 V),^36^ this pathway is not favorable and thus determined to be irrelevant in the dominant catalytic cycle. An alternate route is the radical addition to a π-allyl-Pd intermediate. This addition can occur at the allyl ligand resulting in the new C–C bond or through coordination to the Pd centre followed by inner-sphere reductive elimination to form the new C–C bond.^6,37^ In either event, the cross-coupled product would be liberated along with a Pd(i) species. This species is expected to have a reduction potential around −1.26 V (ref. 31) which would be well-matched with the **4CzIPN** radical anion (−1.21 V).^10^ The decreased efficiency seen with **4CzPN** may be due to the less negative oxidation potential (−1.16 V)^10^ that is not as well-matched for Pd(i) reduction as that of **4CzIPN**, further supporting the relevancy of a Pd(i) reduction to the catalytic cycle (Table S1†).

**Scheme 7:**
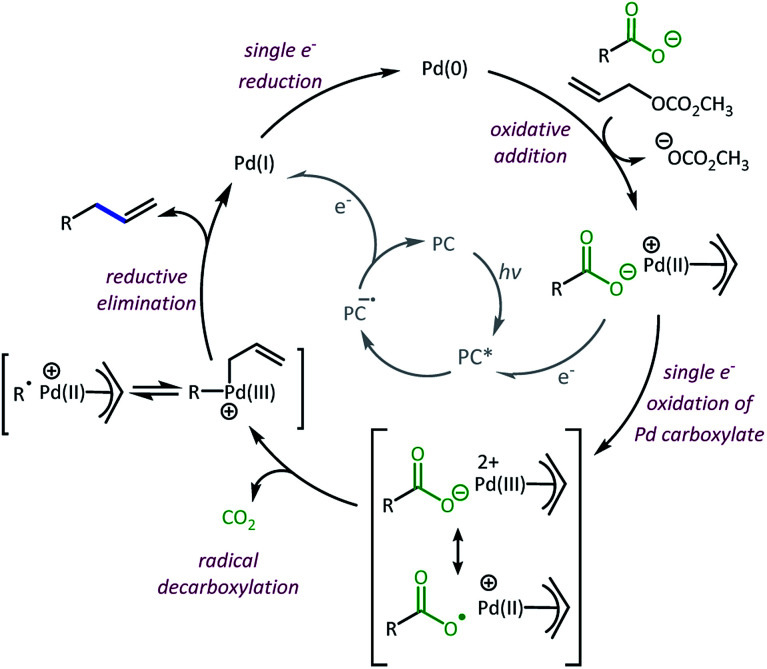
Proposed dominant catalytic pathway.^38,39^

Taken together, we believe the dominant catalytic pathway to proceed *via* a reductive quenching pathway in which the excited **4CzIPN** is quenched by a π-allyl-Pd carboxylate species (Scheme 7).^38^ Single electron transfer from carboxylate to Pd then facilitates decarboxylation and the formation of a carbon radical. Rebound of the radical with the π-allyl-Pd results in the formation of the allylated product and a Pd(i). The Pd(i) can be reduced by one electron *via* the **4CzIPN** radical anion to complete both catalytic cycles.^39^

## Conclusions

A catalytic, mild, operationally simple, and minimal waste-producing decarboxylative method for the site-specific installation of allylic, dienyl, styrenyl, and benzylic functionalities on carboxylic acids has been realized. This method has drastically improved upon our previously reported methodology through the change to a more sustainable and less expensive organophotocatalyst which also led to higher yields across a much broader substrate scope. In addition, the cross-coupling methodology described herein provides a general way to incorporate diverse electrophiles as well as utilize carboxylic acid nucleophiles that do not undergo decarboxylation under thermal control. Thus, the methodology herein may provide a simple and general approach towards building molecular complexity from easily accessible and inexpensive starting materials that would be otherwise difficult to achieve.

## Conflicts of interest

There are no conflicts to declare.

## Supplementary Material

SC-011-D0SC02609C-s001
