# Supplementary material for: Organophotoredox/palladium dual catalytic decarboxylative Csp3–Csp3 coupling of carboxylic acids and π-electrophiles
Source: Chem Sci. 2020 Jul 16;11(31):8167–75. doi: 10.1039/d0sc02609c (PMC8163213; doi:10.1039/d0sc02609c)
Supplement: SC-011-D0SC02609C-s001 [file SC-011-D0SC02609C-s001.pdf]

Electronic Supplementary Material (ESI) for Chemical Science.
This journal is © The Royal Society of Chemistry 2020

Organophotoredox/Palladium Dual Catalytic Decarboxylative Csp^3-Csp^3 Coupling of Carboxylic Acids and π-Electrophiles

Kaitie C. Cartwright and Jon A. Tunge*

Supporting Information

Table of Contents:

1. General Considerations 2

2. Experimental Procedures .... 3 - 4

3. Additional Reaction Optimization Tables .... 5 - 8

4. Fluorescence Quenching Experimental Details .... 9 - 11

5. Alternative Mechanism Outlines .... 11 - 12

6. Compound Characterization .... 13 - 26

7. References .... 26 - 27

8. Spectral Data .... 28 - 85

1. General considerations

All *N*-Boc and *N*-Cbz amino acids are commercially available and were used without further purification. *N*-Acetyl amino acids, 1-[(1,1-dimethylethoxy)carbonyl]-L-prolyl-L-phenylalanine, *N*-acetyl-norleucine, and *N*-acetyl-glutamic acid are commercially available and were used without further purification. Other acyl-protected variants were previously made in our lab.[1] All additional carboxylic acid substrates are commercially available and were utilized without further purification. Allyl methyl carbonate was purchased from Oakwood and used without further purification. Other allylic and benzylic carbonates were synthesized by procedures described herein. The carbazole fluorophore photocatalysts were synthesized from corresponding fluorocyanobenzenes and carbazole, purchased from Sigma-Aldrich, following reported literature procedure.[2] The $Pd(OAc)_2$ (reagent grade, >99%) pre-catalyst was purchased from Sigma-Aldrich. The BINAP ligand (95%) was purchased from Chem-Impex. Anhydrous solvents were purchased from Acros. Final decarboxylative allylation and benzylation reactions were run in a screw threaded tube from Chemglass (CLS-4208). Kessil H150 Blue LED grow lights provided 450 nm light. One light was used per reaction vessel and placed 2 mm from the blue plastic rim that surrounds the face of the light (Figure S1). A 2.0 mL solution of acetonitrile (MeCN) had an internal temperature of 28 ^{o}C after 1 hour under standard reaction conditions. Purification was accomplished with column chromatography using silica gel (60 Å porosity, 230 x 400 mesh, standard grade) which was purchased from Sorbent Technologies (catalog # 30930M-25). TLC analysis was performed (fluorescence quenching and potassium permanganate acid stain) with silica gel HL TLC plates with UV254 purchased from Sorbent Technologies. ^{1}H and ^{13}C NMR spectra were obtained on a Bruker ADVANCE 500 DRX equipped with a QNP cryoprobe. These spectra were referenced to residual protio solvent signals. HRMS data was obtained on an ESI LC-TOF Micromass LCT (Waters). HRMS data was collected using ESI mass spectrometry. GC/MS data was acquired on Shimadzu GCMS-QP2010 SE. Fluorescence quenching analysis for Stern-Volmer relationship data was obtained using PTI fluorometer (S/N 1266) with LPS-220 lamp and 710 photomultiplier system. UV-visual data was acquired with Ocean Optics DT-MINI-2-GS.

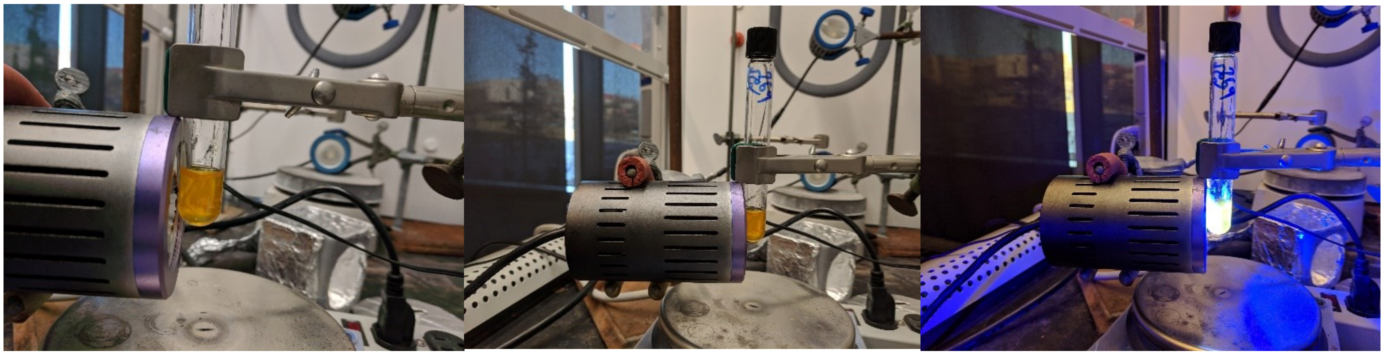

Figure S1: Irradiation set-up with Kessil H150 blue LED lamp.

2. Experimental Procedures

2.1 General Procedure for the Synthesis of Allyl and Benzyl Carbonates

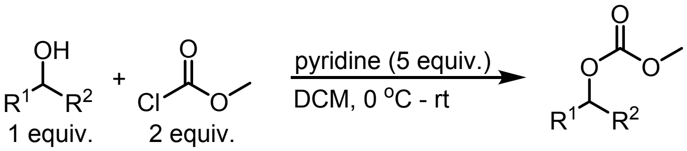

Allylic and benzylic carbonates were prepared from the corresponding allylic and benzylic alcohols according to the modified literature procedure reported by Trost.[3] A flame-dried Schlenk flask with stir bar under an atmosphere of N_2 was charged with the allylic or benzylic alcohol (1 equiv.) and anhydrous DCM (0.2 M). The resulting solution was cooled to 0 °C. Methyl chloroformate (2 equiv.) was then added via syringe followed by the dropwise addition of pyridine (5 equiv.) via syringe. The reaction was allowed to stir ~14 hours while it warmed to room temperature. The reaction mixture was then quenched with saturated aqueous ammonium chloride (~ half reaction volume). The organics were collected, and the aqueous collection was extracted with DCM (3x, 20 mL). The combined organics were dried over $MgSO_4$ and concentrated under reduced pressure to provide the crude product. Products were purified via silica flash chromatography in 1:5-1:20 EtOAc:Hexanes.

2.2 General procedure for the synthesis of branched allylic alcohols

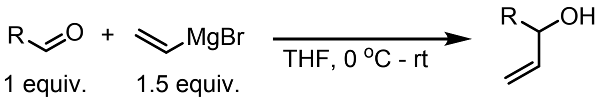

A flame-dried Schlenk flask with stir bar under N_2 was charged with aldehyde (1 equiv., 9.4 mmol) and THF (70 mL, 0.13 M). The reaction mixture was cooled to 0 °C using an ice bath. Vinyl magnesium bromide (1.4 equiv., 13.2 mmol) was added via syringe and the reaction was allowed to warm to room temperature while stirring ~14 h. The reaction was quenched with 1 M HCl (~20 mL) and THF was removed under reduce pressure. The remaining aqueous solution was extracted with DCM (3x, ~20 mL), dried with $MgSO_4$, and concentrated under reduced pressure. The resulting allylic alcohols were utilized without further purification.

2.3 Reduction of aromatic aldehydes

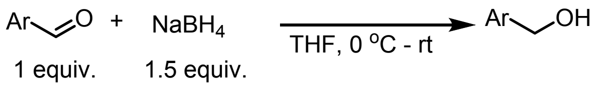

A round bottom flask with stir bar was charged with the corresponding aldehyde (1 equiv., 2.4 mmol) and THF (10 mL, 0.24 M). The reaction mixture was cooled to 0 °C. Sodium borohydride (1.5 equiv., 3.6 mmol, 0.14 g) was then added and the reaction was stirred while warming to room temperature for 1 hour. After, the reaction was quenched with H_2O (~10 mL) and THF was removed under reduced pressure. The remaining aqueous solution was extracted with DCM (3x, ~10 mL), dried with $MgSO_4$, and concentrated under reduced pressure to provide the desired benzylic alcohol. The benzylic alcohols synthesized were used without further purification.

2.4 4CzIPN/Pd Dual Catalytic Allylation and Benzylation of Carboxylic Acids

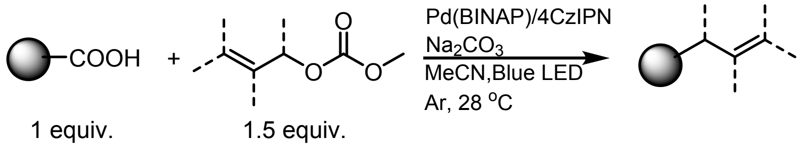

In an argon atmosphere glove box, a 10-mL screw-threaded glass tube equipped with stir bar was charged with $Pd(OAc)_2$ (10 mol%, 0.02 mmol, 0.0045 g), BINAP (11 mol%, 0.022 mmol, 0.0145 g), and MeCN (1 mL). The reaction vessel was sealed and allowed to stir for 30 minutes during which time the reaction mixture goes from a yellow, heterogenous solution to a homogenous orange solution. After the pre-stir, the corresponding allylic or benzylic carbonate (1.5 equiv., 0.3 mmol), the corresponding carboxylic acid (1 equiv., 0.2 mmol), sodium carbonate (1 equiv., 0.2 mmol, 0.0212 g), and 4CzIPN (3.2 mol%, 0.0064 mmol, 0.005 g) were added to the tube followed by MeCN (1 mL) so that the total reaction volume is 2 mL (0.1 M). The tube is sealed and removed from the glovebox. The reaction was stirred and irradiated with one 32 W Kessil blue LED lamp for 16 hours and reaches 28 ^{o}C after the first hour of irradiation (Figure S1). After such time, the reaction was removed from the light, the tube was opened, and the solvent was removed under reduced pressure. The resulting product was purified via silica gel flash chromatography in either 1:5-1:40 EtOAc:Hexanes or 1:10 Et_2O:Pentane for products with molecular weights under 150 g/mol.

2.5 Larger scale DcA Procedure

The procedure outlined in 2.4 was utilized but with a 20-mL borosilicate glass Biotage microwave vial (Biotage no. 355631) sealed with Biotage cap (Biotage no. 352298). The vial was charged with $Pd(OAc)_2$ (10 mol%, 0.1 mmol, 0.0225 g), BINAP (11 mol%, 0.11 mmol, 0.0725 g), and MeCN (5 mL). The catalyst solution was allowed to stir 30 minutes. Boc-Phe-OH (**1b**) (1 equiv., 1 mmol, 0.265 g), allyl methyl carbonate (1.5 equiv., 1.5 mmol, 0.17 mL), sodium carbonate (1 equiv., 1 mmol, 0.106 g), and 4CzIPN (3.2 mol%, 0.032 mmol, 0.025 g) were added followed by MeCN (5 mL) so the total reaction volume was 10 mL (0.1 M). The vial was then sealed with crimper and irradiated in the light set-up displayed in Figure S1. After 16 hours of irradiation, the vial was removed from the light, MeCN was removed under reduced pressure, and the allylated product (**2b**) was purified via flash chromatography on silica gel in 1:20 EtOAc:Hexanes.

3. Additional Reaction Optimization Tables

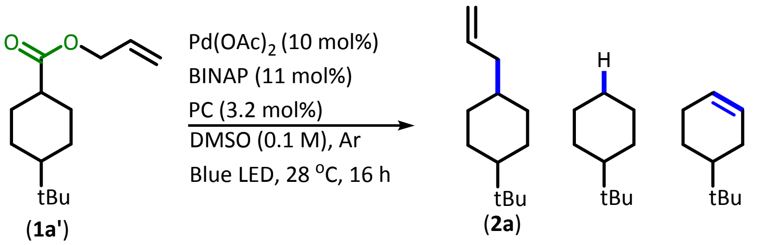

Entry	PC	(2a)	alkane	alkene	(1a')	(1a)
1	4CzIPN	82	9	9	0	0
2	4CzPN	48	4	4	24	20
3	4DPAIPN	0	1	1	98	0
4	2CzTPN	5	2	1	92	0
5	$Ir[dF(CF_3)ppy]_2(dtbbpy)^+PF_6^-$	84	6	10	0	0

Table S1: Carbazole-based donor-acceptor fluorophores in DcA
[a] All product ratios (X%/100%) reported were determined by GC/MS.

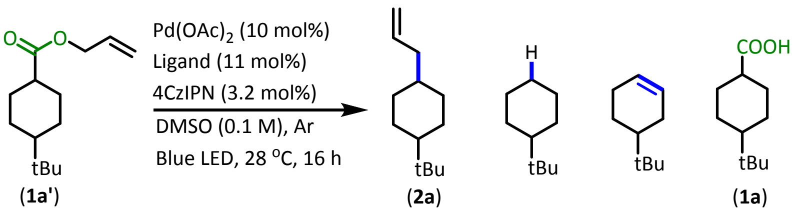

Entry	Ligand	(2a)	alkane	alkene	(1a')	(1a)
1	BINAP	82	9	9	0	0
2	(R)-C_3-TUNEPHOS	78	8	14	0	0
3	(R)-SEGPHOS	76	9	15	0	0
4	(S)-SYNPHOS	75	8	17	0	0
5	(R)-Tol-BINAP	73	8	19	0	0
6	DTBM Segphos	70	8	22	0	0
7	DPEPhos	70	8	22	0	0
8	Xantphos	56	12	32	0	0
9	DIFLUORPHOS	51	3	6	0	14
10	(S,S)-DIOP	40	5	9	0	45
11	(S,S)-CHIRAPHOS	32	29	39	0	0
12	(R)-MOP[b]	19	21	10	50	0
13	PhanePhos	9	1	3	0	87
14	tBu-BOX	0	50	50	0	0
15	(R,R)-ANDEN-PhenylTrost	0	0	0	0	100
16	HiersoPhos	0	0	0	0	100
17	MeOBIPHEP[c]	trace	trace	trace	0	trace
18	MonoPhos	0	0	0	>99	trace
19	Phosphoramidite[d]	0	0	0	>99	trace
20	PCy_3[b]	0	0	0	100	0
21	PhDavePhos	0	0	0	100	0
22	TFP	0	0	0	100	0
23	$P(p\text{-}CF_3Ph)_3$[b]	0	0	0	100	0
24	XPhos	0	0	0	100	0

Table S2: Palladium catalyst evaluation
[a] All reactions were performed on 0.2 mmol scale and product ratios (X%/100%) were determined by GC/MS. [b] Monodentate ligand loading 22 mol%. [c] Many side products. [d] (+)-*N,N*-Bis[(1R)-1-phenylethyl]-dinaphtho[2,1-d:1',2'-f][1,3,2]dioxaphosphepin-4-amine.

In addition to the change in Pd catalyst, the catalyst concentration and the ratio of Pd catalyst to photocatalyst were investigated (Table S4). A ~3:1 ratio of Pd:4CzIPN was found to provide full conversion to predominately the allylated product. Of the reactions performed with this ideal catalyst ratio, the 10 mol % Pd catalyst loading and 3.2 mol % 4CzIPN

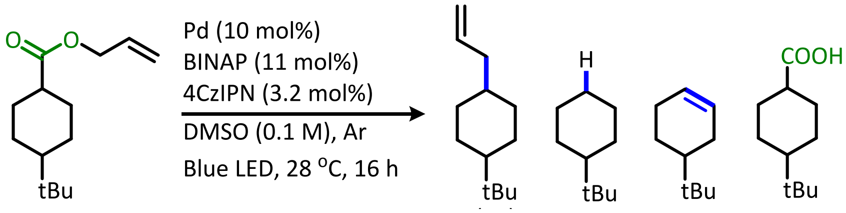

Table S3: Palladium pre-catalyst screening
[a] All product ratios (X%/100%) reported were determined by GC/MS.

1	$Pd(OAc)_2$	82	9	9	0	0
2	$Pd(dba)_2$	77	15	8	0	0
3	$Pd_2(dba)_3$	75	17	8	0	0
4		59	10	31	0	0
	$PdCl_2$					

were identified to be optimal as these were the lowest catalyst loadings that provided the highest yield of allylated product.

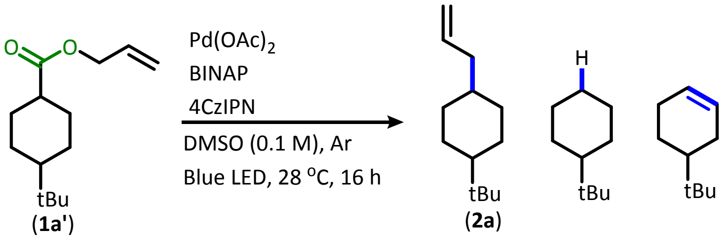

Entry	Pd(OAc)$_2$ (mol%)	BINAP (mol%)	4CzIPN (mol%)	(2a)	alkane	alkene
1	20	21	6.4	86	5	8
2	10	11	5	67	12	7
3	10	11	3.2	82	9	9
4	10	10	0.6	64	7	11
5	5	6	1.6	71	15	14
6	5	6	5	28	9	9
7	5	6	10	20	6	6
8	2.5	3.5	0.6	1	1	1

Table S4: Catalyst loading

[a] All product ratios reported were determined by GC/MS. Product mixtures that don't add to 100% contained unconverted allylester and free carboxylic acid.

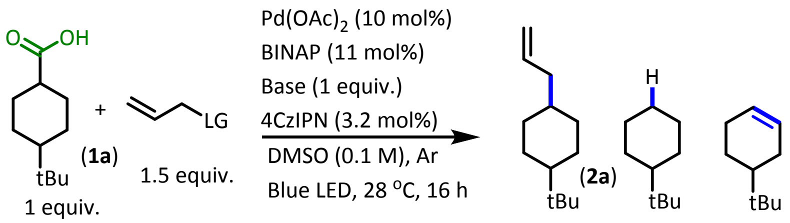

Entry	Electrophile	base	(2a)	alkane	alkene
1	allyl methyl carbonate	Na_2CO_3	84	5	11
2	allyl chloride	Na_2CO_3	68	9	23
3	allyl phosphate	Na_2CO_3	43	0	0
4	allyl methyl carbonate	Cs_2CO_3	71	8	21
5	allyl methyl carbonate	NaOAc	61	7	9
6	allyl methyl carbonate	Na_2HPO_4	57	4	8
7	allyl methyl carbonate	K_2CO_3	56	6	11
8	allyl methyl carbonate	Na_3PO_4	56	5	17
9	allyl methyl carbonate	Li_2CO_3	54	5	7
10	allyl methyl carbonate	LiOAc	32	3	5
11					

Table S5: Intermolecular allylation of carboxylic acid **1a**

[a] All product ratios reported were determined by GC/MS

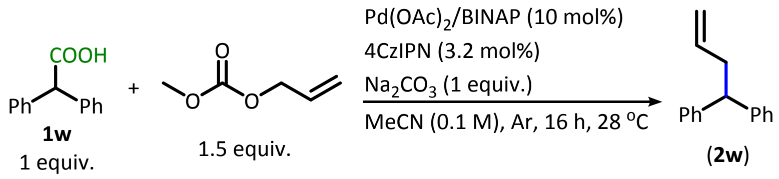

Pd/BINAP	4CzIPN	Blue LED	Yield 2w
✓	✓	✓	72%
✓	✓	-	0%
✓	-	✓	2%
-	✓	✓	0%

Table S6: Catalyst and light dependence in DcA

[a] Reactions were performed on 0.2 mmol scale. [b] Yield of **2w** in control reactions were determined by ^{1}H NMR with pyridine internal standard.

4. Fluorescence Experiments

Acetonitrile utilized in the quenching experiments was degassed via three cycles of freeze-pump-thaw.[4] Palladium catalyst stock solutions were allowed to stir for 1 hour before use. Samples were prepared in an argon atmosphere glove box. All samples contained 3.75 μM of 4CzIPN taken from a 7.5 μM stock solution. Reported counts are an average of four data points.

4.1 Carboxylate Salt Quenching Data

Tetrabutylammonium diphenylcarboxylate was synthesized via literature procedure.[5] Samples were prepared via dilutions from a 40 mM (Set A) and a 1.5 mM (Set B) stock solution in acetonitrile.

Set A [carboxylate]	Io/I	Counts		Set B [carboxylate]	Io/I	Counts
20 mM	1.80	25707.96		0.75 mM	1.03	15772.78
10 mM	1.43	32399.68		0.375 mM	1.00	16167.85
5 mM	1.27	36496.80		0.1875 mM	0.96	16858.6
2.5 mM	1.19	38893.24		0.09375 mM	1.05	15498.2
1.25 mM	1.09	42358.62		0.04688 mM	1.04	15617
0 mM	1	46228.8		0 mM	1	16221.03

Table S7: Fluorescence quenching data of 4CzIPN with tetrabutylammonium carboxylate salt

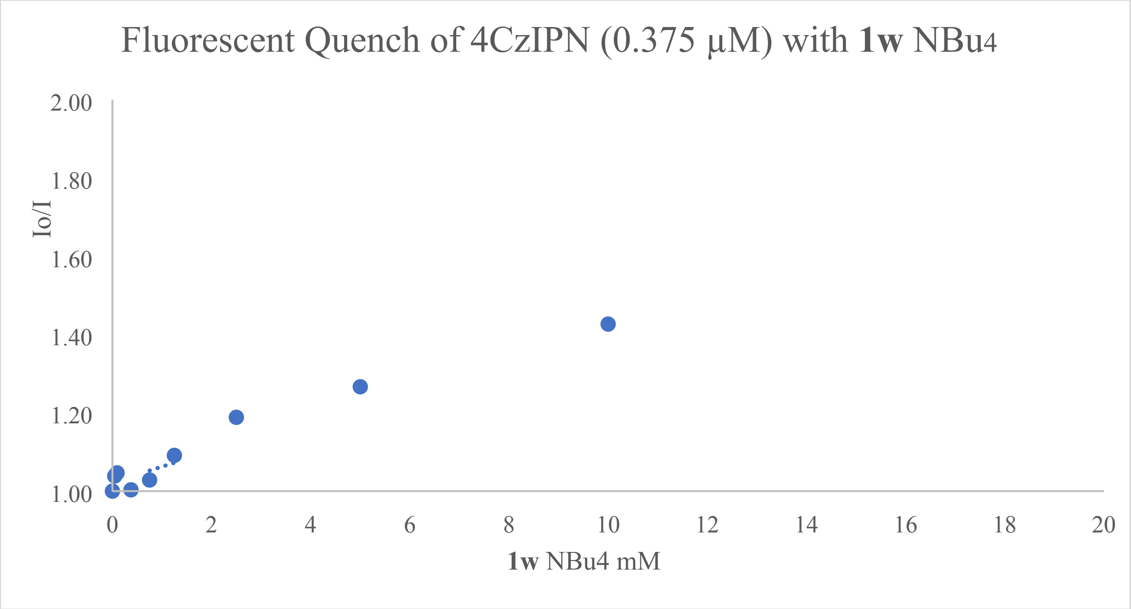

Figure S2: Stern-Volmer Plot of 4CzIPN quench with tetrabutylammonium carboxylate salt

4.2 Palladium Pre-Catalyst Quenching Data

<u>Sample Set A</u>: A solution of $Pd(OAc)_2$ (1.5 mM) and BINAP (1.6 mM) in acetonitrile was allowed to stir for one hour under argon. This stock solution was utilized to make fluorescence quenching samples.

<u>Sample Set B</u>: A solution of $Pd(OAc)_2$ (1.5 mM) and BINAP (4.5 mM) in acetonitrile was stirred at 60 ^{o}C for 48 hours under argon. This solution was utilized to make fluorescence quenching samples.

Set A				Set B		
[Pd]	Counts	Io/I		[Pd]	Counts	Io/I
0 mM	44911.5	1		0 mM	45341.98	1
0.75 mM	28066.2	1.600199		0.75 mM	31926.73	1.420189
0.375 mM	32561.13	1.379298		0.375 mM	23916.5	1.895845
0.1875 mM	35705.43	1.257834		0.1875 mM	25278.18	1.79372
0.09375 mM	36484.73	1.230967		0.09375 mM	41620.18	1.089423
0.04688 mM	39121.68	1.147995		0.04688 mM	42318.75	1.071439

Table S8: Pd/BINAP fluorescence quenching data

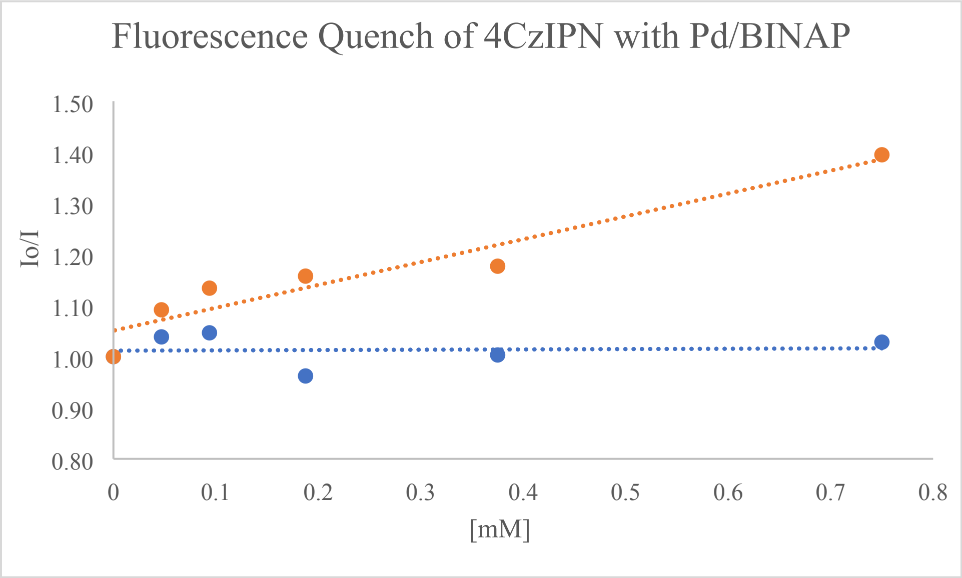

Figure S3: Stern-Volmer Plot of 4CzIPN quench with $Pd(OAc)_2$/BINAP

4.3 Palladium Allyl Quenching Data

Pd(Allyl)Cl Dimer/BINAP Quencher: A solution of Pd(Allyl)Cl Dimer (1.5 mM) and BINAP (1.6 mM) in acetonitrile was allowed to stir for one hour under argon. This solution was then utilized to make fluorescence quenching samples.

Pd(Allyl)OAc Dimer/BINAP Quencher: A solution of Pd(Allyl)OAc Dimer was synthesized following literature procedure.[6] A solution of Pd(Allyl)OAc Dimer (1.5 mM) and BINAP (1.6 mM) in acetonitrile was allowed to stir for one hour under argon. This solution was then utilized to make fluorescence quenching samples.

[Pd(Allyl)Cl Dimer]	Counts	Io/I		[PdAllylOAc Dimer]	Counts	Io/I
0 mM	39540.6	1		0 mM	43931.1	1
0.75 mM	42969.4	0.92		0.75 mM	31490.03	1.40
0.375 mM	37601.9	1.05		0.375 mM	37325.1	1.18
0.188 mM	49986.7	0.79		0.188 mM	37951.08	1.16
0.094 mM	44939.3	0.87		0.094 mM	38739.03	1.13
0.047 mM	46110.1	0.86		0.047 mM	40242.68	1.09

Table S9: PdAllyl fluorescence quenching data

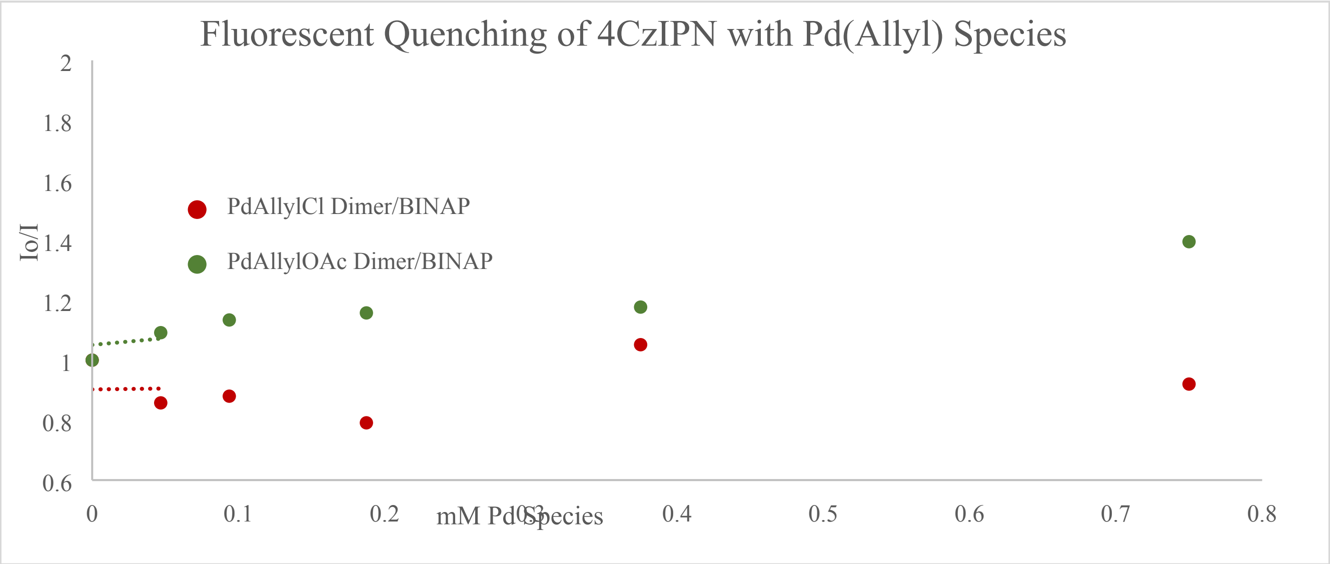

Figure S4: Stern-Volmer plot of 4CzIPN quench with PdAllyl species

5. Additional Mechanism Outlines

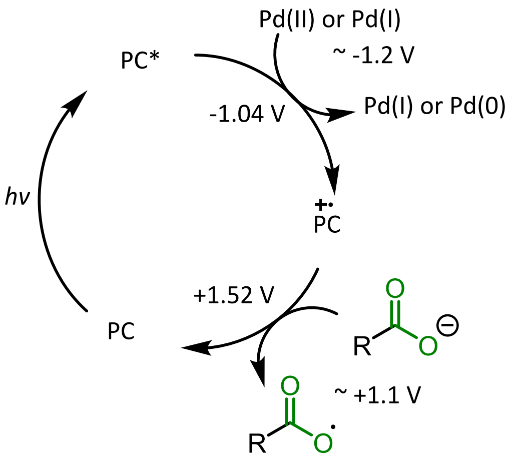

Scheme S1: Hypothetical pre-catalyst reduction pathway

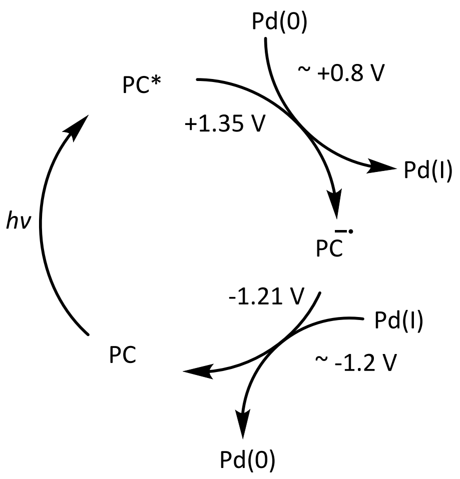

Scheme S2: Possible unproductive catalytic pathway

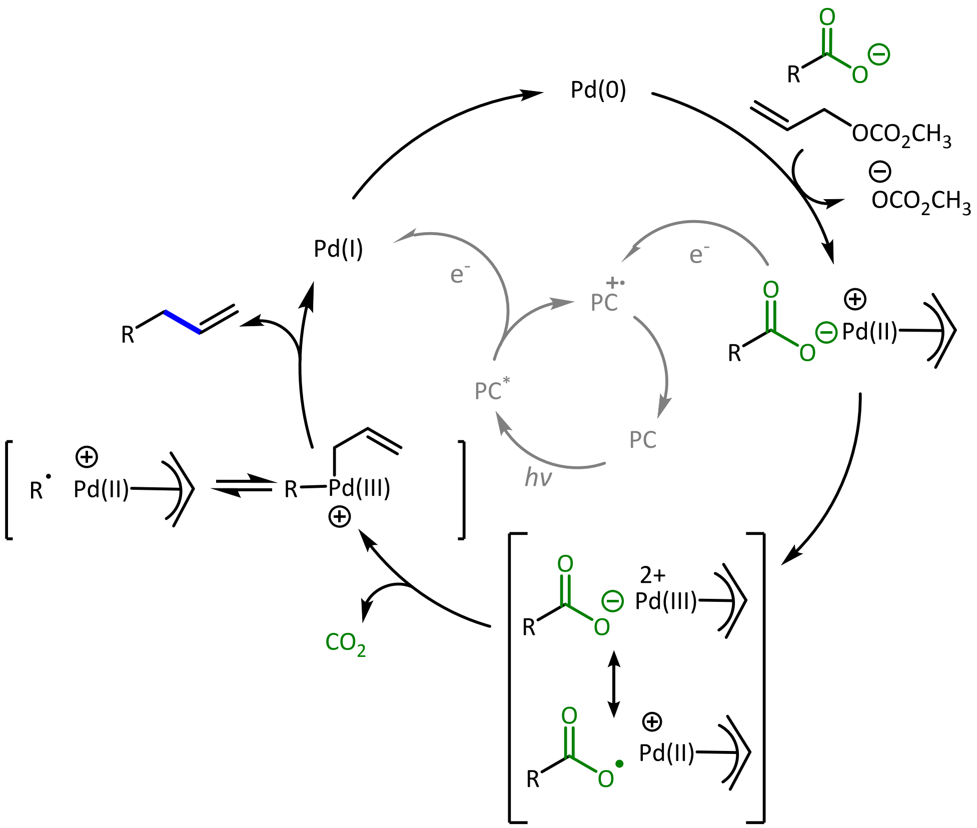

Scheme S3: Hypothetical oxidative quenching pathway

6. Compound Characterizations

5.1 Decarboxylative Allylation Products from Carboxylic Acids and Allyl Methyl Carbonate Synthesized via General Procedure 2.4.

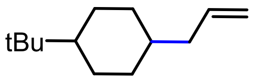

1-allyl-4-(tert-butyl)cyclohexane (**2a**). Product volatile, 73% yield determined from crude reaction mixture by q^{1}H NMR with pyridine as the internal standard. Product matched previously reported literature specification.[7] ^{1}H NMR (500 MHz, $CDCl_3$): Mix of diastereomers (60:40 d.r.): δ 5.89–5.72 (m, 1H), 5.08–4.94 (m, 2H), [2.12 (dt) & 1.96 (dt), ∑2H], 1.83–1.63 (m, 5H), 1.54–1.41 (m, 2H), 1.35–1.20 (m, 2H), 1.19–1.07 (m, 1H), 0.87–0.85 (m, 9H). ^{13}C NMR (126 MHz, $CDCl_3$) δ 138.8, 138.0, 115.3, 115.1, 48.6, 48.3, 42.0, 38.0, 35.8, 33.6, 32.5, 30.3, 27.7, 27.7, 27.4, 27.4, 21.7.

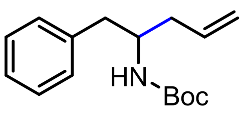

tert-butyl (1-phenylpent-4-en-2-yl)carbamate (**2b**). Isolated 44.3 mg, 85% yield as a light yellow amorphous solid. Product matched previously reported literature specification.[8] ^{1}H NMR (500 MHz, $CDCl_3$): δ 7.23-7.11 (m, 2H), 7.14–7.04 (m, 3H), 5.69 (ddt, *J* = 17.2, 10.3, 7.0 Hz, 1H), 5.32–4.66 (m, 2H), 4.30 (broad s, 1H), 3.80 (broad s, 1H), 2.66 (td, *J* = 16.5, 13.6, 6.6 Hz, 2H), 2.15 (dt, *J* = 13.7, 6.3 Hz, 1H), 2.08–1.94 (m, 1H), 1.30 (s, 9H). ^{13}C NMR (126 MHz, $CDCl_3$): δ 155.5, 138.2, 134.3, 129.6, 128.5, 126.5, 118.1, 79.2, 51.2, 40.6, 38.3, 28.4.

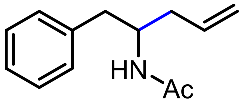

N-(1-phenylpent-4-en-2-yl)acetamide (**2c**). Isolated 26.9 mg, 66% yield as a yellow oil. Product matched previously reported literature specification.[8] ^{1}H NMR (500 MHz, $CDCl_3$): δ 7.33–7.27 (m, 2H), 7.25–7.15 (m, 3H), 6.06–5.54 (m, 1H), 5.30 (s, 1H), 5.27–4.82 (m, 2H), 4.70–4.01 (m, 1H), 2.81 (h, *J* = 6.5 Hz, 2H), 2.31-2.24 (m, 1H), 2.21–2.00 (m, 1H), 1.92 (s, 3H). ^{13}C NMR (126 MHz, $CDCl_3$): δ 169.6, 138.0, 134.5, 129.5, 128.6, 126.6, 118.2, 49.6, 40.1, 37.9, 23.6.

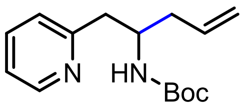

tert-butyl (1-(pyridin-2-yl)pent-4-en-2-yl)carbamate (**2d**). Isolated 41.0 mg, 93% yield as a yellow oil. Product matched previously reported literature specification.[8] ^{1}H NMR (500 MHz, $CDCl_3$): δ 8.52 (s, 1H), 7.58 (t, *J* = 7.7 Hz, 1H), 7.22–6.82 (m, 2H), 5.80 (ddt, *J* = 17.2, 10.4, 7.1 Hz, 1H), 5.28 (broad s, 1H), 5.05 (dd, *J* = 13.5, 8.9 Hz, 2H), 4.00 (p, *J* = 6.9 Hz, 1H), 3.14–2.66 (m, 2H), 2.23 (qt, *J* = 14.0, 6.7 Hz, 2H), 1.36 (s, 9H). ^{13}C NMR (126 MHz, $CDCl_3$): δ 159.0, 155.5, 149.3, 136.5, 134.8, 124.1, 121.5, 117.8, 79.0, 50.6, 42.0, 38.7, 28.3.

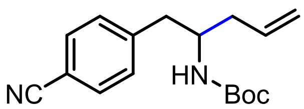

tert-butyl (1-(4-cyanophenyl)pent-4-en-2-yl)carbamate (**2e**). Isolated 44.5 mg, 78% yield as a white solid. Product matched previously reported literature specification.[8] ^{1}H NMR (500 MHz, $CDCl_3$): δ 7.57 (d, *J* = 7.9 Hz, 2H), 7.30 (d, *J* = 7.8 Hz, 2H), 5.77 (ddt, *J* = 17.1, 10.2, 7.0 Hz, 1H), 5.22–4.96 (m, 2H), 4.42 (broad s, 1H), 3.90 (broad s, 1H), 2.83 (d, *J* = 6.8 Hz, 2H), 2.24 (dt, *J* = 13.3, 6.3 Hz, 1H), 2.13 (dt, *J* = 14.3, 7.2 Hz, 1H), 1.37 (s, 9H). ^{13}C NMR (126 MHz, $CDCl_3$): δ 155.4, 144.2, 134.0, 132.2, 130.4, 128.7, 119.1, 118.6, 110.4, 79.6, 51.0, 41.2, 38.4, 28.2.

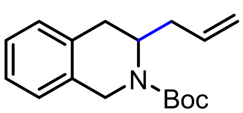

tert-butyl 3-allyl-3,4-dihydroisoquinoline-2(1H)-carboxylate (**2f**). Isolated 51.3 mg, 94% yield as a colorless oil. Product matched previously reported literature specification.[8] ^{1}H NMR (500 MHz, $CDCl_3$): δ 7.21-7.14 (m, 2H), 7.11 (d, *J* = 6.3 Hz, 2H), 5.78 (dq, *J* = 16.7, 7.8 Hz, 1H), 5.08–4.90 (m, 2H), 4.79 (broad s, 1H), 4.72–4.35 (m, 1H), 4.25 (d, 1H), 3.03 (dd, *J* = 15.8, 5.8 Hz, 1H), 2.69 (d, *J* = 15.8 Hz, 1H), 2.46–2.15 (m, 1H), 2.04 (dt, *J* = 14.2, 7.3 Hz, 1H), 1.50 (s, 9H). ^{13}C NMR (126 MHz, $CDCl_3$): δ 155.1, 135.3, 133.0, 129.5, 129.1, 126.7, 126.3, 117.3, 80.1, 50.0, 48.4, 43.2, 42.6, 37.0, 36.4, 32.9, 32.4, 28.5.

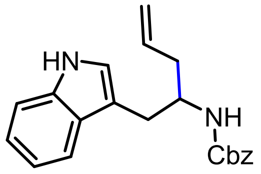

benzyl (1-(1H-indol-3-yl)pent-4-en-2-yl)carbamate (**2g**). Isolated 52.7 mg, 79% yield as an orange oil. ^{1}H NMR (500 MHz, $CDCl_3$): δ 8.14 (broad s, 1H), 7.66 (d, *J* = 7.5 Hz, 1H), 7.41–7.32 (m, 6H), 7.24–7.16 (m, 1H), 7.13 (d, *J* = 7.5 Hz, 1H), 6.97 (broad s, 1H), 5.82 (ddt, *J* = 14.1, 10.5, 7.0 Hz, 1H), 5.16–5.04 (m, 4H), 4.75 (d, *J* = 8.4 Hz, 1H), 4.12–4.07 (m, 1H), 2.98 (tq, *J* = 14.7, 6.8, 6.1 Hz, 2H), 2.35 (dt, *J* = 13.3, 6.2 Hz, 1H), 2.19 (dt, *J* = 15.0, 7.5 Hz, 1H). ^{13}C NMR (126 MHz, $CDCl_3$): δ 156.1, 136.3, 134.6, 128.6, 128.2, 122.8, 122.1, 119.6, 119.1, 118.1, 112.0, 111.2, 66.6, 51.2, 38.5, 30.1. HRMS: Calc'd $C_{21}H_{22}N_2O_2Na$ (M+Na) = 357.1579, found 357.1582. IR (film): 2925, 2854, 1700, 1653, 1559, 1521, 1507, 1457, 1430, 1419, 1340, 1260, 1219, 1058, 1027, 913 cm^{-1}.

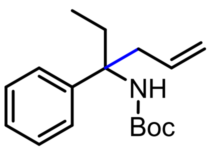

tert-butyl (3-phenylhex-5-en-3-yl)carbamate (**2h**). Isolated 46.3 mg, 85% yield as a yellow oil. ^{1}H NMR (500 MHz, $CDCl_3$): (mix of rotamers) δ 7.43-7.27 (m, 3H), 7.18–7.00 (m, 2H), 5.55 (dq, *J* = 17.2, 8.9, 8.4 Hz, 1H), 5.23 – 4.91 (m, 2H), 4.77 (broad s, 1H), 2.97–2.75 (m, 1H), 2.72 (q, *J* = 7.6 Hz, 1H), 2.68–2.53 (m, 1H), 2.07–1.80 (m, 2H), 1.39 (broad d, 9H), 1.20 (t, *J* = 7.7 Hz, 2H), 0.70 (t, *J* = 7.4 Hz, 3H). ^{13}C NMR (126 MHz, $CDCl_3$): (mix of rotamers) δ 162.2, 155.5, 133.7, 128.7, 128.2, 126.5, 125.8, 118.7, 82.0, 60.5, 41.6, 31.6, 28.5, 28.4, 8.1. HRMS: Calc'd $C_{17}H_{29}N_2O_2$ (M+NH$_4$) = 293.2229, found 293.2212. IR (film): 3422, 3356, 3287, 2976, 2935, 2880, 1724, 1699, 1494, 1446, 1391, 1365, 1271, 1252, 1170, 1082, 1001, 916 cm^{-1}.

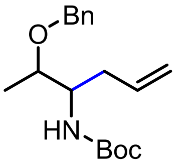

tert-butyl (2-(benzyloxy)hex-5-en-3-yl)carbamate (**2i**). Isolated 14.5 mg, 30% yield as a colorless oil. ^{1}H NMR (500 MHz, $CDCl_3$): δ 7.53–7.27 (m, 5H), 5.93–5.57 (m, 1H), 5.16–4.93 (m, 2H), 4.79 (d, *J* = 9.6 Hz, 1H), 4.61 (d, *J* = 11.5 Hz, 1H), 4.38 (d, *J* = 11.5 Hz, 1H), 3.78–3.29 (m, 2H), 2.31 (hept, *J* = 7.0 Hz, 2H), 1.43 (s, 9H), 1.20 (d, *J* = 6.2 Hz, 3H). ^{13}C NMR (126 MHz, $CDCl_3$): δ 156.2, 138.6, 135.3, 128.3, 127.8, 117.3, 79.3, 74.7, 71.0, 54.6, 37.6, 28.3, 16.3. HRMS: Calc'd $C_{18}H_{28}NO_3$ (M+H) = 306.2069, found 306.2065. IR (film): 3447, 3343, 2978, 2930, 2871, 1718, 1715, 1700, 1507, 1457, 1399, 1366, 1274, 1172, 1066, 993, 916 cm^{-1}.

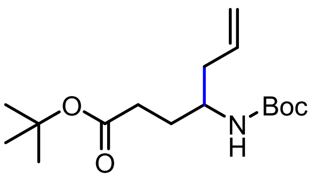

tert-butyl 4-((tert-butoxycarbonyl)amino)hept-6-enoate (**2j**). Isolated 51.1 mg, 85% yield as a yellow oil. ^{1}H NMR (500 MHz, $CDCl_3$): δ 5.82–5.70 (m, 1H), 5.14–4.88 (m, 2H), 4.41 (d, *J* = 9.3 Hz, 1H), 3.61 (tt, *J* = 10.6, 5.2 Hz, 1H), 2.27 (t, *J* = 7.5 Hz, 2H), 2.20 (q, *J* = 7.1 Hz, 2H), 1.79 (dtd, *J* = 15.4, 7.9, 4.9 Hz, 1H), 1.61 (p, *J* = 8.0 Hz, 1H), 1.43 (s, 9H), 1.41 (s, 9H). ^{13}C NMR (126 MHz, $CDCl_3$): δ 172.8, 154.8, 134.1, 118.0, 80.4, 78.9, 50.0, 39.6, 32.3, 29.4, 28.3, 27.9. HRMS: Calc'd $C_{16}H_{29}NO_4Na$ (M+Na) = 322.1994, found 322.1981. IR (film): 3354, 2978, 2933, 1717, 1691, 1525, 1452, 1392, 1367, 1251, 1220, 1170, 1053, 993, 914 cm^{-1}.

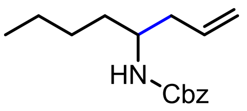

benzyl oct-1-en-4-ylcarbamate (**2k**). Isolated 42.6 mg, 81% yield as a light yellow oil. Product matched previously reported literature specification.[9] ^{1}H NMR (500 MHz, $CDCl_3$): δ 7.56–7.28 (m, 5H), 5.77 (dq, *J* = 16.6, 7.5 Hz, 1H), 5.25–4.90 (m, 2H), 4.57 (d, *J* = 9.0 Hz, 1H), 3.71 (q, *J* = 7.3 Hz, 1H), 2.23 (ddt, *J* = 42.2, 14.2, 6.7 Hz, 2H), 1.49 (dd, *J* = 11.2, 5.7 Hz, 1H), 1.43–1.18 (m, 6H), 0.90 (d, *J* = 6.1 Hz, 3H). ^{13}C NMR (126 MHz, $CDCl_3$): δ 156.1, 136.8, 134.4, 128.6, 128.2, 117.9, 66.6, 50.8, 39.6, 34.4, 28.2, 22.7, 14.1.

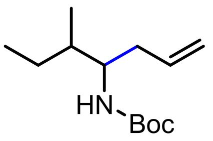

tert-butyl (5-methylhept-1-en-4-yl)carbamate (**2l**). Isolated 33.1 mg, 73% yield as a colorless oil. ^{1}H NMR (500 MHz, $CDCl_3$): (mix of rotamers) δ 5.76 (ddtd, *J* = 17.0, 9.7, 7.1, 2.5 Hz, 1H), 5.09–4.99 (m, 2H), 4.42-4.24 (m, 1H), 3.69-3.48 (m, 1H), 2.30-1.98 (m, 2H), 1.53-1.44 (m, 2H), 1.42 (s, 9H), 1.22–1.01 (m, 1H), 0.93–0.80 (m, 6H). ^{13}C NMR (126 MHz, $CDCl_3$): (mix of rotamers) δ 155.9, 135.3, 117.2, 117.1, 79.0, 54.3, 53.4, 38.4, 38.0, 37.7, 36.2, 28.5, 26.4, 25.2, 15.3, 14.1,

11.8. HRMS: Calc'd $C_{13}H_{24}NO_2$ (M-H) = 226.1807, found 226.1811. IR (film): 2965, 2933, 1734, 1700, 1696, 1684, 1653, 1559, 1539, 1521, 1517, 1490, 1465, 1437, 1248, 1219, 1175 cm^{-1}.

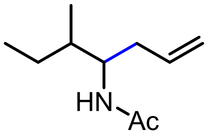

N-(5-methylhept-1-en-4-yl)acetamide (**2m**). Isolated 19.0 mg, 56% yield as a colorless oil. ^{1}H NMR (500 MHz, CD_3CN): (mix of diastereomers, d.r. 56:44) δ 6.12 (broad d, 1H), 5.86–5.65 (m, 1H), 5.11–4.90 (m, 2H), [3.89 (tt, *J* = 9.5, 4.9 Hz) & 3.77 (tdd, *J* = 9.6, 5.8, 4.1 Hz) ∑1H], 2.32–2.19 (m, 1H), 2.15–1.98 (m, 1H), 1.83 (d, 3H), [1.46 (dtd, *J* = 13.0, 6.9, 6.3, 2.6 Hz) & 1.37 (tt, *J* = 13.1, 7.3 Hz) ∑2H], 1.18–1.02 (m, 1H), 0.94–0.81 (m, 6H). ^{13}C NMR (126 MHz, CD_3CN): (mix of diastereomers) δ 170.3, 170.1, 137.1, 136.9, 116.9, 116.8, 53.5, 52.5, 39.2, 39.0, 37.8, 36.3, 27.0, 26.0, 23.1, 15.5, 14.5, 12.0, 11.9. HRMS: Calc'd $C_{10}H_{19}NONa$ (M+Na) = 192.1364, found 192.1373. IR (film): 3287, 3077, 2963, 2933, 2876, 1646, 1554, 1457, 1437, 1374, 1297, 1150, 993, 958, 913 cm^{-1}.

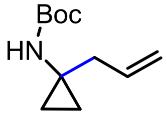

tert-butyl (1-allylcyclopropyl)carbamate (**2n**). Isolated 8.4 mg, 21% yield as a colorless oil. Product matched previously reported literature specification.[10] ^{1}H NMR (500 MHz, $CDCl_3$): δ 5.89–5.69 (m, 1H), 5.15–4.95 (m, 2H), 4.86 (broad s, 1H), 2.30 (broad d, 2H), 1.43 (s, 9H), 0.73 (broad t, 2H), 0.64 (broad t, 2H). ^{13}C NMR (126 MHz, $CDCl_3$): δ 155.7, 135.2, 117.3, 79.0, 40.6, 32.9, 28.2, 13.0.

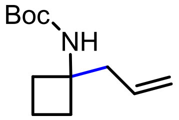

tert-butyl (1-allylcyclobutyl)carbamate (**2o**). Isolated 39.4 mg, 93% yield as a colorless oil. ^{1}H NMR (500 MHz, $CDCl_3$): δ 5.77 (ddt, *J* = 16.0, 11.6, 7.3 Hz, 1H), 5.13–5.07 (m, 2H), 4.61 (broad s, 1H), 2.50 (d, *J* = 7.3 Hz, 2H), 2.35–2.12 (m, 2H), 2.00 (ddd, *J* = 12.7, 9.2, 3.7 Hz, 2H), 1.87 (ddq, *J* = 14.1, 9.4, 4.0 Hz, 1H), 1.81–1.68 (m, 1H), 1.43 (s, 9H). ^{13}C NMR (126 MHz, $CDCl_3$): δ 154.2, 134.1, 118.5, 79.1, 55.6, 42.2, 32.1, 28.7, 14.2. HRMS: Calc'd $C_{12}H_{25}N_2O_2$ ($M+NH_4$) = 229.1916, found 229.1925. IR (film): 3352, 3076, 2978, 2937, 1717, 1696, 1507, 1391, 1365, 1278, 1250, 1172, 1066, 995, 914 cm^{-1}.

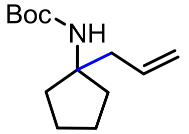

tert-butyl (1-allylcyclopentyl)carbamate (**2p**). Isolated 40.5 mg, 90% yield as an amorphous colorless solid. ^{1}H NMR (500 MHz, $CDCl_3$): δ 5.86–5.65 (m, 1H), 5.10–4.91 (m, 2H), 4.42 (broad s, 1H), 2.48 (d, *J* = 7.5 Hz, 2H), 1.89-1.77 (m, 2H), 1.76–1.64 (m, 2H), 1.65-1.55 (m, 4H), 1.41 (s, 9H). ^{13}C NMR (126 MHz, $CDCl_3$): δ 154.8, 134.9, 117.7, 79.0, 63.3, 42.0, 37.7, 28.5, 23.4. HRMS: Calc'd $C_{13}H_{23}NO_2Li$ (M+Li) = 232.1889, found 232.1887. IR (film): 3448, 3357, 3075, 2976, 2872, 1718, 1695, 1517, 1490, 1452, 1390, 1365, 1276, 1245, 1172, 1091, 989, 912 cm^{-1}.

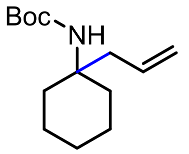

tert-butyl (1-allylcyclohexyl)carbamate (**2q**). Isolated 40.7 mg, 84% yield as a colorless oil. ^{1}H NMR (500 MHz, $CDCl_3$): δ 5.76 (ddt, *J* = 17.6, 10.5, 7.4 Hz, 1H), 5.14–4.82 (m, 2H), 4.25 (broad s, 1H), 2.44 (d, *J* = 7.6 Hz, 2H), 1.91 (d, *J* = 13.3 Hz, 2H), 1.60–1.43 (m, 5H), 1.42 (s, 9H), 1.36–1.17 (m, 3H). ^{13}C NMR (126 MHz, $CDCl_3$): δ 154.5, 134.2, 118.1, 78.6, 54.3, 35.0, 28.4, 25.8, 21.7. HRMS: Calc'd $C_{14}H_{25}NO_2Na$ (M+Na) = 262.1783, found 262.1786. IR (film): 3450, 3366, 3075, 2977, 2931, 2858, 1722, 1700, 1496, 1448, 1390, 1365, 1248, 1220, 1167, 1086, 973, 913 cm^{-1}.

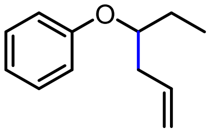

(hex-5-en-3-yloxy)benzene (**2r**). Isolated 21.3 mg, 60% yield as a colorless oil. ^{1}H NMR (500 MHz, $CDCl_3$): δ 7.26–7.18 (m, 2H), 6.93–6.85 (m, 3H), 5.84 (ddt, *J* = 17.2, 10.0, 7.0 Hz, 1H), 5.19–4.94 (m, 2H), 4.20 (p, *J* = 5.8 Hz, 1H), 2.40 (dtdd, *J* = 15.6, 14.4, 7.1, 5.8 Hz, 2H), 1.76–1.62 (m, 2H), 0.95 (t, *J* = 7.4 Hz, 3H). ^{13}C NMR (126 MHz, $CDCl_3$): δ 158.6, 134.5, 129.5, 120.6, 117.3, 116.1, 78.8, 37.9, 26.5, 9.6. HRMS: Calc'd $C_{12}H_{20}ON$ ($M+NH_4$) = 194.1545, found 194.1535. IR (film): 2971, 2331, 1700, 1653, 1599, 1559, 1496, 1241, 1220 cm^{-1}.

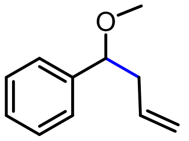

(1-methoxybut-3-en-1-yl)benzene (**2s**). Isolated 10.0 mg, 31% yield as a colorless oil. Product matched previously reported literature specification.[11] ^{1}H NMR (500 MHz, CD_3CN): δ 7.39–7.34

(m, 2H), 7.31–7.25 (m, 3H), 5.82–5.71 (m, 1H), 5.04–4.93 (m, 2H), 4.20 (dd, *J* = 7.3, 5.9 Hz, 1H), 3.15 (s, 3H), 2.56–2.47 (m, 1H), 2.38 (dtt, *J* = 14.3, 7.2, 1.3 Hz, 1H). ^{13}C NMR (126 MHz, CD_3CN): δ 143.0, 136.1, 129.3, 128.5, 127.7, 117.1, 84.3, 56.7, 42.9.

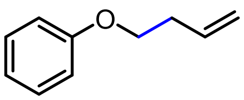

(but-3-en-1-yloxy)benzene (**2t**). Isolated 9.6 mg, 32% yield as a colorless oil. Product matched previously reported literature specification.[12] ^{1}H NMR (500 MHz, $CDCl_3$): δ 7.38–7.24 (m, 2H), 7.02–6.87 (m, 3H), 5.92 (ddt, *J* = 17.1, 10.3, 6.7 Hz, 1H), 5.23–5.07 (m, 2H), 4.02 (t, *J* = 6.7 Hz, 2H), 2.55 (qt, *J* = 6.7, 1.4 Hz, 2H). ^{13}C NMR (126 MHz, $CDCl_3$): δ 159.0, 134.6, 129.6, 120.9, 117.3, 114.9, 67.3, 33.8.

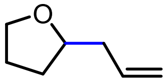

2-allyltetrahydrofuran (**2u**). Product volatile, 83% yield determined from crude reaction mixture by q^{1}H NMR with pyridine internal reference standard. Product matched previously reported literature specification.[13] ^{1}H NMR (500 MHz, $CDCl_3$): δ 5.93–5.79 (m, 1H), 5.16–4.99 (m, 2H), 3.85–3.76 (m, 2H), 3.71–3.59 (m, 1H), 2.03–1.94 (m, 6H).

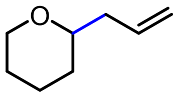

2-allyltetrahydro-2H-pyran (**2v**). Isolated 10.9 mg, 43% yield as a colorless oil. Product matched previously reported literature specification.[14] ^{1}H NMR (500 MHz, $CDCl_3$): δ 5.77 (ddt, *J* = 17.2, 10.2, 7.0 Hz, 1H), 5.05–4.91 (m, 2H), 3.91 (ddt, *J* = 11.5, 4.2, 1.9 Hz, 1H), 3.36 (td, *J* = 11.7, 2.5 Hz, 1H), 3.28–3.21 (m, 1H), 2.25–2.17 (m, 1H), 2.15–2.06 (m, 1H), 1.78–1.72 (m, 1H), 1.54 (ddt, *J* = 14.5, 4.6, 1.9 Hz, 1H), 1.46–1.37 (m, 2H), 1.26–1.15 (m, 2H). ^{13}C NMR (126 MHz, $CDCl_3$): δ 135.3, 116.7, 68.8, 41.3, 31.6, 30.5, 26.2, 23.6.

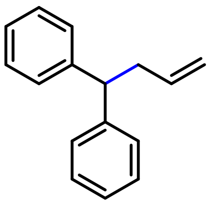

but-3-ene-1,1-diyldibenzene (**2w**). Isolated 30.2 mg, 72% yield as a colorless oil. Product matched previously reported literature specification.[15] ^{1}H NMR (500 MHz, $CDCl_3$): δ 7.41–7.26 (m, 10H), 5.81 (ddt, *J* = 17.0, 10.2, 6.8 Hz, 1H), 5.12 (dq, *J* = 17.1, 1.6 Hz, 1H), 5.04 (ddt, *J* = 10.2, 2.3, 1.2 Hz, 1H), 4.14–4.05 (m, 1H), 2.91 (ddt, *J* = 8.0, 6.7, 1.4 Hz, 2H). ^{13}C NMR (126 MHz, $CDCl_3$): δ 144.6, 137.0, 129.1, 128.5, 128.1, 126.3, 116.4, 51.4, 40.1.

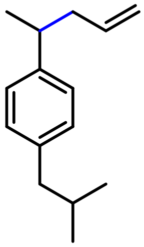

1-isobutyl-4-(pent-4-en-2-yl)benzene (**2x**). Isolated 24.6 mg, 62% yield as a colorless oil. Contains ~5% alkene product, 1-isobutyl-4-vinylbenzene. Product **2x** matched previously reported literature specification.[16] ^{1}H NMR (500 MHz, $CDCl_3$): δ 7.08 (m, 4H), 5.78–5.66 (m, 1H), 5.03–4.92 (m, 2H), 2.76 (h, *J* = 7.1 Hz, 1H), 2.49–2.42 (m, 2H), 2.42 – 2.33 (m, 1H), 2.26 (dtd, *J* = 13.9, 7.6, 1.2 Hz, 1H), 1.85 (dh, *J* = 13.5, 6.7 Hz, 1H), 1.24 (dd, *J* = 7.0, 1.1 Hz, 3H), 0.90 (d, *J* = 6.5 Hz, 6H). ^{13}C NMR (126 MHz, $CDCl_3$): δ 144.4, 139.3, 137.5, 129.1, 126.8, 115.9, 45.2, 42.9, 39.5, 30.4, 22.6, 21.6.

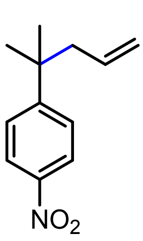

1-(2-methylpent-4-en-2-yl)-4-nitrobenzene (**2y**). Isolated 24.3 mg total, 27% yield allylated product **2y** and 23% yield alkane product *1-isopropyl-4-nitrobenzene* (**2y'**). Allylated product matched previously reported literature specification.[15] ^{1}H NMR (500 MHz, $CDCl_3$): (**2y** & **2y'**) δ 8.18–8.13 (m, **2y** & **2y'**, 4H), 7.51– 7.47 (m, **2y**, 2H), 7.40–7.34 (m, **2y'**, 2H), 5.57–5.42 (m, **2y**, 1H), 5.01–4.92 (m, **2y**, 2H), 3.02 (hept, *J* = 6.9 Hz, **2y'**, 1H), 2.39 (dd, *J* = 7.3, 1.3 Hz, **2y**, 2H), 1.35 (s, **2y**, 6H), 1.29 (d, *J* = 6.9 Hz, **2y'**, 2H). ^{13}C NMR (126 MHz, $CDCl_3$): (**2y** & **2y'**) δ 157.2, 156.7, 134.3, 127.4, 127.0, 123.8, 123.4, 118.1, 48.7, 38.6, 34.4, 28.5, 23.7.

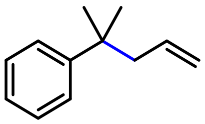

(2-methylpent-4-en-2-yl)benzene (**2z**). Isolated an average of 15.3 mg, 48% yield over two reactions as a colorless oil. Product matched previously reported literature specification.[15] ^{1}H NMR (500 MHz, $CDCl_3$): δ 7.39–7.26 (m, 4H), 7.22–7.15 (m, 1H), 5.56 (ddt, *J* = 17.3, 10.1, 7.2

Hz, 1H), 5.00–4.92 (m, 2H), 2.40–2.35 (m, 2H), 1.32 (s, 6H). ^{13}C NMR (126 MHz, $CDCl_3$): δ 149.4, 135.7, 128.2, 126.0, 125.7, 117.0, 48.8, 37.7, 28.4, 22.0.

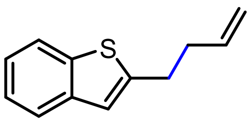

2-(but-3-en-1-yl)benzo[b]thiophene (**2aa**). Isolated 11.8 mg, 31% yield as a colorless oil. Product matched previously reported literature specification.[17] ^{1}H NMR (500 MHz, $CDCl_3$): δ 7.86 (ddd, *J* = 8.1, 3.4, 1.0 Hz, 1H), 7.75 (ddd, *J* = 15.6, 7.8, 1.2 Hz, 1H), 7.44–7.32 (m, 2H), 7.12 (s, 1H), 5.94 (ddt, *J* = 16.9, 10.2, 6.5 Hz, 1H), 5.12 (dq, *J* = 17.2, 1.9 Hz, 1H), 5.04 (ddt, *J* = 10.0, 2.8, 1.4 Hz, 1H), 2.98–2.92 (m, 2H), 2.53 (tdd, *J* = 7.6, 6.5, 1.2 Hz, 2H). ^{13}C NMR (126 MHz, $CDCl_3$): δ 140.8, 139.4, 138.4, 136.6, 124.5, 124.2, 123.3, 122.0, 121.6, 115.6, 33.6, 28.5.

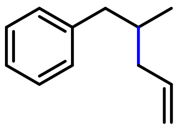

(2-methylpent-4-en-1-yl)benzene (**2bb**). Isolated an average of 16.4 mg, 46% yield over two reactions as a colorless oil. Product matched previously reported literature specification.[18] ^{1}H NMR (500 MHz, $CDCl_3$): δ 7.32–7.27 (m, 2H), 7.22–7.15 (m, 3H), 5.89–5.77 (m, 1H), 5.07–5.01 (m, 2H), 2.68 (dd, *J* = 13.4, 6.1 Hz, 1H), 2.44–2.32 (m, 1H), 2.14 (dddd, *J* = 13.7, 6.9, 5.5, 1.4 Hz, 1H), 2.00–1.92 (m, 1H), 1.89–1.79 (m, 1H), 0.89 (d, *J* = 6.6 Hz, 3H). ^{13}C NMR (126 MHz, $CDCl_3$): δ 141.5, 137.5, 129.3, 128.3, 125.8, 116.1, 43.2, 41.1, 35.2, 19.6.

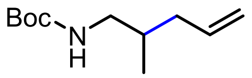

tert-butyl (2-methylpent-4-en-1-yl)carbamate (**2cc**). Isolated 25.9 mg, 65% yield as a colorless oil. ^{1}H NMR (500 MHz, $CDCl_3$): δ 5.77 (ddt, *J* = 17.1, 10.0, 7.1 Hz, 1H), 5.06–4.96 (m, 2H), 4.56 (broad s, 1H), 3.08 (dt, *J* = 12.9, 6.3 Hz, 1H), 2.94 (dt, *J* = 13.4, 6.6 Hz, 1H), 2.10 (dt, *J* = 13.4, 6.4 Hz, 1H), 1.90 (dt, *J* = 14.4, 7.4 Hz, 1H), 1.69 (dp, *J* = 11.6, 5.7, 4.7 Hz, 1H), 1.44 (s, 9H), 0.90 (d, *J* = 6.7 Hz, 3H). ^{13}C NMR (126 MHz, $CDCl_3$): δ 156.2, 136.8, 116.5, 79.3, 46.2, 38.8, 33.9, 28.4, 17.3. HRMS: Calc'd $C_{11}H_{21}NO_2Na$ (M+Na) = 222.1470, found 222.1461. IR (film): 2975, 2928, 1700, 1680, 1653, 1159, 1507, 1219, 1174 cm^{-1}.

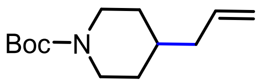

tert-butyl 4-allylpiperidine-1-carboxylate (**2dd**). Isolated 23.8 mg, 53% yield as a colorless oil. Product matched previously reported literature specification.[19] ^{1}H NMR (500 MHz, $CDCl_3$): δ 5.81–5.70 (m, 1H), 5.03–4.92 (m, 2H), 4.06 (broad s, 1H), 3.35 (t, *J* = 5.5 Hz, 2H), 2.72-2.61 (broad m, 1H), 1.99 (t, *J* = 7.0 Hz, 1H), 1.68-1.61 (m, 1H), 1.58-1.53 (m, 1H), 1.52-1.47 (m, 2H), 1.44 (s, 9H), 1.08 (qd, 2H).^{13}C NMR (126 MHz, $CDCl_3$): δ 155.0, 136.7, 115.7, 79.3, 41.0, 36.1, 32.0, 28.6, 24.6.

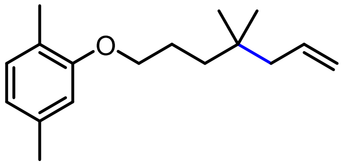

2-((4,4-dimethylhept-6-en-1-yl)oxy)-1,4-dimethylbenzene (**2ee**). Isolated 38.7 mg, 78% yield as a colorless oil. ^{1}H NMR (500 MHz, $CDCl_3$): δ 7.03 (d, *J* = 7.4 Hz, 1H), 6.73–6.63 (m, 2H), 5.86 (ddt, *J* = 17.5, 10.3, 7.4 Hz, 1H), 5.11–4.98 (m, 2H), 3.93 (t, *J* = 6.6 Hz, 2H), 2.34 (s, 3H), 2.21 (s, 3H), 2.02 (d, *J* = 7.5 Hz, 2H), 1.79 (dq, *J* = 13.2, 6.7 Hz, 2H), 1.42–1.35 (m, 2H), 0.93 (s, 6H). ^{13}C NMR (126 MHz, $CDCl_3$): δ 157.5, 136.8, 136.0, 130.7, 124.0, 121.0, 117.2, 112.4, 69.0, 46.8, 38.2, 33.3, 27.4, 24.6, 21.7, 16.2. HRMS: Calc'd $C_{17}H_{25}O$ (M-H) = 245.1905, found 245.1909. IR (film): 3074, 2954, 2925, 2868, 1616, 1586, 1509, 1471, 1414, 1387, 1285, 1265, 1158, 1130, 1039, 997, 912 cm^{-1}.

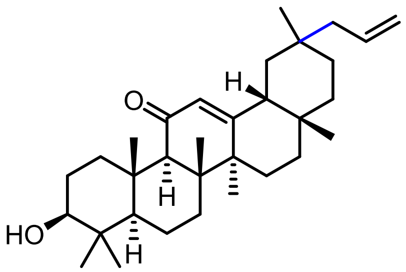

(4aR,6aS,6bR,8aR,10S,12aS,12bR,14bR)-2-allyl-10-hydroxy-2,4a,6a,6b,9,9,12a-heptamethyl-1,3,4,4a,5,6,6a,6b,7,8,8a,9,10,11,12,12a,12b,14b-octadecahydropicen-13(2H)-one (**2ff**). Isolated 68.5 mg, 73% yield as a colorless oil. ^{1}H NMR (500 MHz, $CDCl_3$): (mix of rotamers) δ 5.86–5.68 (m, 1H), 5.61-5.53 (m, 1H), 5.07–4.94 (m, 2H), 3.21 (dd, *J* = 11.3, 5.2 Hz, 1H), 2.78 (dt, *J* = 13.5, 3.2 Hz, 1H), 2.33

(s, 1H), 2.21-1.94 (m, 3H), 1.92 (d, *J* = 7.5 Hz, 1H), 1.81 (tt, *J* = 13.7, 3.9 Hz, 1H), 1.71-1.54 (m, 5H), 1.51-1.37 (m, 4H), 1.36 (s, 3H), 1.31–1.23 (m, 3H), 1.20–1.14 (m, 2H), 1.12 (s, 6H), 1.05 (dt, *J* = 13.6, 3.4 Hz, 1H), 1.00 (s, 3H), 0.98–0.89 (m, 2H), 0.89-0.82 (m, 6H), 0.80 (s, 3H). ^{13}C NMR (126 MHz, $CDCl_3$): (mix of rotamers) δ 200.5, 200.4, 170.6, 170.5, 134.9, 134.8, 128.4, 128.3, 128.2, 117.4, 117.3, 78.9, 61.9, 55.1, 55.1, 50.3, 47.3, 47.0, 45.5, 43.5, 43.5, 43.3, 42.8, 41.5, 41.0, 39.6, 37.2, 36.2, 36.0, 34.1, 33.7, 33.5, 33.0, 32.9, 32.9, 32.7, 32.4, 32.3, 29.6, 28.9, 28.9, 28.2, 27.5, 26.8, 26.6, 26.6, 26.5, 23.5, 22.5, 21.4, 18.9, 17.6, 16.5, 15.7. HRMS: Calc'd $C_{32}H_{49}O_2$ (M-H) = 465.3733, found 465.3721. IR (film): 2968, 2949, 2926, 2866, 1653, 1648, 1636, 1617, 1457, 1387, 1260, 1218, 1210, 1134, 1091, 1044, 994, 913, 801, 791, 785, 779, 775, 767, 744 cm^{-1}.

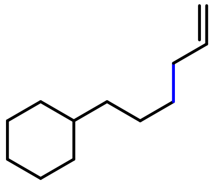

hex-5-en-1-ylcyclohexane (**2gg**). Product is commercially available. Yield found the be 15% by q^{1}H NMR with pyridine internal standard from the crude DcA reaction mixture. Allylated product and alkane product were isolated together. See Section 8 for ^{1}H and ^{13}C spectra.

5.2 Allylic and Benzylic Carbonates

Allylic carbonates **3b**, **3c**, **3d**, **3e**, **3f**, **3i**, **3p** were made previously in our lab.[20]

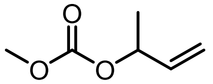

but-3-en-2-yl methyl carbonate (**3e'**). General procedure 2.1 provided the allylic carbonate from but-3-en-2-ol. Product matched previously reported literature specification.[21] ^{1}H NMR (500 MHz, $CDCl_3$): δ 5.92-5.80 (m, 1H), 5.35-5.25 (dt, 1H), 5.22-5.14 (m, 2H), 3.78 (s, 3H), 1.37 (d, 3H). ^{13}C NMR (126 MHz, $CDCl_3$): δ 155.3, 137.2, 116.7, 75.4, 55.2, 20.2.

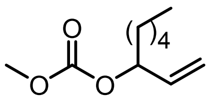

methyl oct-1-en-3-yl carbonate (**3g**). General procedure 2.2 was used to make the allylic alcohol, oct-1-en-3-ol, from hexanal. General procedure 2.1 provided the allylic carbonate from oct-1-en-3-ol. Product matched previously reported literature specification.[22] ^{1}H NMR (500 MHz, $CDCl_3$): δ 5.86-5.74 (m, 1H), 5.30 (dt, 1H), 5.20 (dt, 1H), 5.04 (q, 1H), 3.78 (s, 3H), 1.75-1.55 (m, 2H), 1.40-1.23 (m, 6H), 0.88 (t, 3H). ^{13}C NMR (126 MHz, $CDCl_3$): δ 155.5, 136.2, 117.5, 79.8, 54.8, 34.3, 31.7, 24.8, 22.6, 14.1.

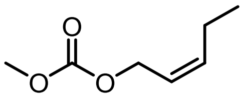

(Z)-methyl pent-2-en-1-yl carbonate (**3h**). General procedure 2.1 provided the allylic carbonate from (Z)-pent-2-en-1-ol. Product matched previously reported literature specification.[23] ^{1}H NMR (500 MHz, $CDCl_3$): δ 5.72-5.59 (m, 1H), 5.59-5.44 (m, 1H), 4.74-4.60 (m, 2H), 3.77 (s, 3H), 2.19-2.04 (m, 2H), 1.03-0.91 (t, 3H). ^{13}C NMR (126 MHz, $CDCl_3$): δ 156.2, 138.1, 122.5, 64.0, 55.1, 21.3, 14.4.

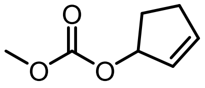

cyclopent-2-en-1-yl methyl carbonate (**3j**). The allylic alcohol, cyclopent-2-en-1-ol, was synthesized from the corresponding enone using previously reported procedure for the Luche reduction.[24] General procedure 2.1 provided the allylic carbonate. Product matched previously reported literature specification.[23] ^{1}H NMR (500 MHz, $CDCl_3$): δ 6.17-6.12 (m, 1H), 5.90-5.84 (m, 1H), 5.66-5.58 (m, 1H), 3.77 (s, 3H), 2.60-2.47 (m, 1H), 2.38-2.22 (m, 2H), 1.97-1.86 (m, 1H). ^{13}C NMR (126 MHz, $CDCl_3$): δ 155.7, 138.7, 128.8, 84.4, 54.6, 31.2, 29.8.

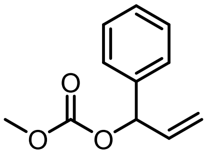

methyl (1-phenylallyl) carbonate (**3k**). General procedure 2.2 was used to make the allylic alcohol, 1-phenylprop-2-en-1-ol, from benzaldehyde. General procedure 2.1 provided the allylic carbonate from 1-phenylprop-2-en-1-ol. Product matched previously reported literature specification.[22] ^{1}H NMR (500 MHz, $CDCl_3$): δ 7.44-7.29 (m, 5H), 6.12-5.98 (m, 2H),

5.35 (dt, 1H), 5.28 (dt, 1H), 3.79 (3H). ^{13}C NMR (126 MHz, $CDCl_3$): δ 155.2, 138.4, 135.4, 128.8, 128.6, 127.2, 117.6, 80.8, 55.0.

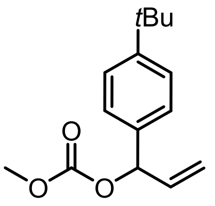

1-(4-(tert-butyl)phenyl)allyl methyl carbonate (**3l**). General procedure 2.2 was used to make the allylic alcohol, 1-(4-(tert-butyl)phenyl)prop-2-en-1-ol, from 4-tertbutylbenzaldehyde. General procedure 2.1 provided the allylic carbonate from 1-(4-(tert-butyl)phenyl)prop-2-en-1-ol. Product matched previously reported literature specification.[22] ^{1}H NMR (500 MHz, $CDCl_3$): δ 7.41-7.36 (m, 2H), 7.33-7.27 (m, 2H), 6.10-5.99 (m, 2H), 5.39-5.31 (m, 1H), 5.29-5.23 (m, 1H), 3.77 (s, 3H), 1.31 (s, 9H). ^{13}C NMR (126 MHz, $CDCl_3$): δ 155.4, 152.7, 135.7, 135.1, 126.7, 125.5, 117.1, 80.0, 54.7, 34.9, 31.2.

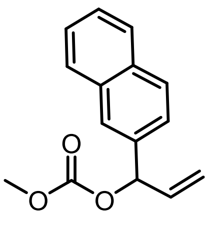

methyl (1-(naphthalen-2-yl)allyl) carbonate (**3m**). General procedure 2.2 was used to make the allylic alcohol, 1-(naphthalen-2-yl)prop-2-en-1-ol, from 2-naphthaldehyde. General procedure 2.1 provided the allylic carbonate from 1-phenylprop-2-en-1-ol. Product matched previously reported literature specification.[22] ^{1}H NMR (500 MHz, $CDCl_3$): δ 7.77-7.58 (m, 4H), 7.41-7.26 (m, 3H), 6.17-6.07 (m, 1H), 6.04-5.89 (m, 1H), 5.28 (dt, 1H), 5.17 (dt, 1H), 3.62 (s, 3H). ^{13}C NMR (126 MHz, $CDCl_3$): δ 155.2, 135.9, 135.7, 133.4, 133.3, 128.7, 128.3, 127.8, 126.5, 126.5, 126.4, 124.8, 117.9, 80.5, 55.4.

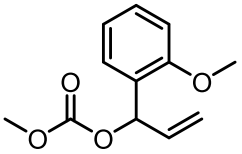

1-(2-methoxyphenyl)allyl methyl carbonate (**3n**). General procedure 2.2 was used to make the allylic alcohol, 1-(2-methoxyphenyl)prop-2-en-1-ol, from 2-methoxybenzaldehyde. General procedure 2.1 provided the allylic carbonate from 1-(2-methoxyphenyl)prop-2-en-1-ol. Product matched previously reported literature specification.[26] ^{1}H NMR (500 MHz, $CDCl_3$): δ 7.36 (dd, *J* = 7.6, 1.7 Hz, 1H), 7.32–7.26 (m, 2H), 6.97 (t, *J* = 7.5 Hz, 1H), 6.89 (d, *J* = 8.2 Hz, 1H), 6.05 (ddd, *J* = 16.6, 10.4, 5.8 Hz, 1H), 5.31 (dt, *J* = 17.2, 1.4 Hz, 1H), 5.21 (dt, *J* = 10.4, 1.3 Hz, 1H), 3.85 (s, 3H), 3.78 (s, 3H). ^{13}C NMR (126 MHz, $CDCl_3$): δ 156.6, 155.2, 135.5, 129.5, 127.4, 127.0, 120.9, 116.7, 110.9, 74.7, 55.7, 54.9.

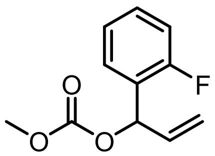

1-(2-fluorophenyl)allyl methyl carbonate (**3o**). General procedure 2.2 was used to make the allylic alcohol, 1-(2-fluorophenyl)prop-2-en-1-ol, from 2-fluorobenzaldehyde. General procedure 2.1 provided the allylic carbonate from 1-(2-fluorophenyl)prop-2-en-1-ol. Product matched previously reported literature specification.[26] ^{1}H NMR (500 MHz, $CDCl_3$): δ 7.41 (td, *J* = 7.5, 1.8 Hz, 1H), 7.38–7.26 (m, 1H), 7.19–7.11 (m, 1H), 7.06 (ddd, *J* = 9.7, 8.2, 1.2 Hz, 1H), 6.39 (dd, *J* = 6.1, 1.5 Hz, 1H), 6.06 (ddd, *J* = 16.8, 10.4, 6.0 Hz, 1H), 5.36 (dq, *J* = 17.2, 1.0 Hz, 1H), 5.29 (dt, *J* = 10.4, 1.2 Hz, 1H), 3.79 (s, 3H). ^{13}C NMR (126 MHz, $CDCl_3$): δ 160.1 (d, *J* = 248.6 Hz), 154.9, 134.7, 130.2 (d, *J* = 8.4 Hz), 128.3 (d, *J* = 3.9 Hz), 125.9 (d, *J* = 13.5 Hz), 124.5 (d, *J* = 3.9 Hz), 117.9, 115.8 (d, 21.5 Hz), 74.3, 55.0.

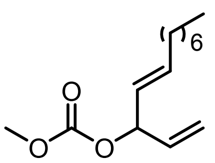

(E)-dodeca-1,4-dien-3-yl methyl carbonate (**3q**). General procedure 2.2 was used to make the allylic alcohol, (E)-dodeca-1,4-dien-3-ol, from (E)-dec-2-enal. General procedure 2.1 provided the allylic carbonate from (E)-dodeca-1,4-dien-3-ol. ^{1}H NMR (500 MHz, $CDCl_3$): δ 5.92–5.74 (m, 2H), 5.52–5.43 (m, 2H), 5.34–5.19 (m, 2H), 3.77 (s, 3H), 2.05 (q, *J* = 7.5 Hz, 2H), 1.37 (p, *J* = 6.9 Hz, 2H), 1.32–1.21 (m, 8H), 0.88 (t, *J* = 6.8 Hz, 3H). ^{13}C NMR (126 MHz, $CDCl_3$): δ 155.2, 136.4, 135.5, 126.3, 117.5, 79.5, 54.8, 32.4, 31.9, 29.2, 29.2, 28.9, 22.8, 14.2. HRMS: Calc'd $C_{14}H_{23}O_3$ (M-H) = 239.1647, found 239.1636. IR (film): 2956, 2927, 2856, 1751, 1700, 1647, 1559, 1507, 1465, 1437, 1262, 966, 934 cm^{-1}.

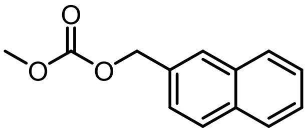

methyl (naphthalen-2-ylmethyl) carbonate (**3r**). General procedure 2.1 provided the benzylic carbonate from naphthalen-2-ylmethanol. Product matched previously reported literature specification.[27] ^{1}H NMR (500 MHz, $CDCl_3$): δ 7.88-7.80 (m, 4H), 7.55-7.44 (m, 3H), 5.33 (s, 2H), 3.81 (s, 3H). ^{13}C NMR (126 MHz, $CDCl_3$): δ 156.7, 133.4, 133.3, 132.8, 128.6, 128.2, 127.9, 127.6, 126.5, 126.5, 125.9, 69.9, 55.1.

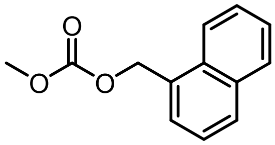

methyl (naphthalen-1-ylmethyl) carbonate (**3s**). General procedure 2.1 provided the benzylic carbonate from naphthalen-1-ylmethanol. Product matched previously reported literature specification.[28] ^{1}H NMR (500 MHz, $CDCl_3$): δ 8.15–7.98 (m, 1H), 7.98–7.79 (m, 2H), 7.65–7.38 (m, 4H), 5.64 (s, 2H), 3.80 (s, 3H). ^{13}C NMR (126 MHz, $CDCl_3$): δ 155.9, 133.9, 131.7, 130.9, 129.8, 128.9, 127.9, 126.9, 126.1, 125.4, 123.6, 68.1, 55.0.

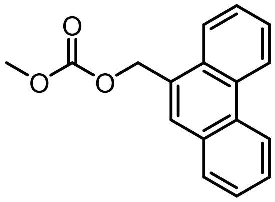

methyl (phenanthren-9-ylmethyl) carbonate (**3t**). General procedure 2.3 provided phenanthren-9-ylmethanol from phenanthrene-9-carbaldehyde. General procedure 2.1 provided the benzylic carbonate from phenanthren-9-ylmethanol. ^{1}H NMR (500 MHz, $CDCl_3$): δ 8.74 (dd, *J* = 7.4, 1.9 Hz, 1H), 8.68 (d, *J* = 8.3 Hz, 1H), 8.13–8.02 (m, 1H), 7.90 (dd, *J* = 7.9, 1.4 Hz, 1H), 7.87 (s, 1H), 7.75–7.64 (m, 3H), 7.61 (ddd, *J* = 8.0, 6.8, 1.2 Hz, 1H), 5.69 (s, 2H), 3.82 (s, 3H). ^{13}C NMR (126 MHz, $CDCl_3$): δ 155.9, 131.2, 130.9, 130.8, 130.3, 129.3, 129.1, 128.8, 127.5, 127.2, 127.0, 126.9, 124.3, 123.4, 122.7, 68.5, 55.1. HRMS: Calc'd $C_{17}H_{13}O_3$ (M-H) = 265.0865, found 265.0872. IR (film): 2974, 1751, 1696, 1684, 1653, 1559, 1534, 1507, 1465, 1395, 1268, 1219 cm^{-1}.

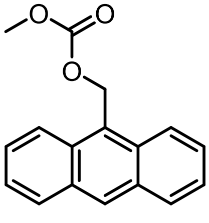

anthracen-9-ylmethyl methyl carbonate (**3u**). General procedure 2.3 provided anthracen-9-ylmethanol from anthracene-9-carbaldehyde. General procedure 2.1 provided the benzylic carbonate from anthracen-9-ylmethanol. ^{1}H NMR (500 MHz, $CDCl_3$): δ 8.53 (s, 1H), 8.39 (dq, *J* = 8.9, 1.0 Hz, 2H), 8.04 (ddt, *J* = 8.5, 1.4, 0.7 Hz, 2H), 7.59 (ddd, *J* = 8.9, 6.6, 1.4 Hz, 2H), 7.54–7.46 (m, 2H), 6.24 (s, 2H), 3.80 (s, 3H). ^{13}C NMR (126 MHz, $CDCl_3$): δ 155.2, 131.5, 131.3, 129.7, 129.3, 127.0, 125.3, 124.1, 62.5, 55.1. HRMS: Calc'd $C_{17}H_{14}O_3$ (M+) = 266.0943, found 266.0945. IR (film): 3065, 1749, 1704, 1680, 1653, 1559, 1534, 1507, 1457, 1267, 1258, 936 cm^{-1}.

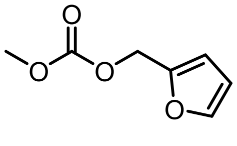

furan-2-ylmethyl methyl carbonate (**3v**). General procedure 2.1 provided the benzylic carbonate from furan-2-ylmethanol. Product matched previously reported literature specification.[29] ^{1}H NMR (500 MHz, $CDCl_3$): δ 7.50 (dt, *J* = 1.6, 0.8 Hz, 1H), 7.40 (t, *J* = 1.7 Hz, 1H), 6.45 (dd, *J* = 1.8, 0.8 Hz, 1H), 5.04 (s, 2H), 3.78 (s, 3H). ^{13}C NMR (126 MHz, $CDCl_3$): δ 155.4, 148.7, 143.4, 111.3, 110.5, 61.2, 54.8.

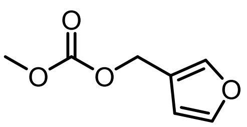

furan-3-ylmethyl methyl carbonate (**3w**). General procedure 2.1 provided the benzylic carbonate from furan-3-ylmethanol. Product matched previously reported literature specification.[30] ^{1}H NMR (500 MHz, $CDCl_3$): δ 7.59–7.43 (m, 1H), 7.40 (t, *J* = 1.7 Hz, 1H), 6.46 (dd, *J* = 1.9, 0.9 Hz, 1H), 5.04 (s, 2H), 3.79 (s, 3H). ^{13}C NMR (126 MHz, $CDCl_3$): δ 155.9, 143.6, 142.1, 119.4, 110.6, 61.1, 55.1.

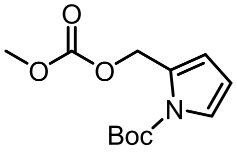

tert-butyl 2-(((methoxycarbonyl)oxy)methyl)-1H-pyrrole-1-carboxylate (**3x**). General procedure 2.3 provided tert-butyl 2-(hydroxymethyl)-1H-pyrrole-1-carboxylate from tert-butyl 2-formyl-1H-pyrrole-1-carboxylate. General procedure 2.1 provided the benzylic carbonate from tert-butyl 2-(hydroxymethyl)-1H-pyrrole-1-carboxylate. ^{1}H NMR (500

MHz, $CDCl_3$): δ 7.29 (dd, *J* = 3.3, 1.8 Hz, 1H), 6.44–6.23 (m, 1H), 6.13 (t, *J* = 3.3 Hz, 1H), 5.33 (d, *J* = 0.7 Hz, 2H), 3.79 (s, 3H), 1.59 (s, 9H). ^{13}C NMR (126 MHz, $CDCl_3$): δ 155.7, 149.0, 128.2, 123.2, 116.7, 109.7, 84.4, 61.8, 55.3, 28.0. HRMS: Calc'd $C_{12}H_{17}NO_5Na$ (M+Na) = 278.1004, found 278.0992. IR (film): 2980, 1749, 1700, 1647, 1559, 1507, 1457, 1373, 1345, 1319, 1272, 1138, 936 cm^{-1}.

5.3 Products of Decarboxylative Allylation and Benzylation of ***1f****.*

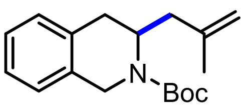

tert-butyl (R)-3-(2-methylallyl)-3,4-dihydroisoquinoline-2(1H)-carboxylate (**4b**) was synthesized via general procedure 2.4. Isolated 36.5 mg, 64% yield as a colorless oil. ^1H NMR (500 MHz, $CDCl_3$): (mix of rotamers) δ 7.23–7.04 (m, 4H), 4.85-4.69 (m, 2H), 4.63-4.68 (m, 2H), 4.28 (d, *J* = 17.1 Hz, 1H), 3.02 (dd, *J* = 15.8, 5.8 Hz, 1H), 2.68 (dd, *J* = 15.7, 2.1 Hz, 1H), 2.21 (dd, *J* = 13.5, 7.5 Hz, 1H), 2.02–1.86 (m, 1H), 1.80 (s, 3H), 1.50 (s, 9H). ^{13}C NMR (126 MHz, $CDCl_3$): (mix of rotamers) δ 155.0, 133.3, 132.9, 129.6, 129.2, 126.7, 126.3, 113.4, 112.9, 79.8, 48.3, 46.8, 43.0, 42.5, 40.7, 40.0, 32.6, 32.2, 28.6, 22.2. HRMS: Calc'd $C_{18}H_{26}NO_2$ (M+H) = 288.1964, found 288.1966. IR (film): 3368, 3072, 2978, 2933, 2252, 1762, 1700, 1457, 1392, 1368, 1291, 1245, 1164, 1048, 947, 899 cm^{-1}.

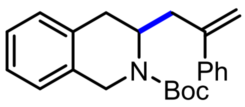

tert-butyl (R)-3-(2-phenylallyl)-3,4-dihydroisoquinoline-2(1H)-carboxylate (**4c**) was synthesized via general procedure 2.4. Isolated 57.4 mg, 82% yield as a light-yellow oil. ^1H NMR (500 MHz, $CDCl_3$): (mix of rotamers) δ 7.46 (d, *J* = 7.6 Hz, 2H), 7.34 (t, *J* = 7.5 Hz, 2H), 7.32–7.24 (m, 1H), 7.23-7.12 (m, 3H), 7.06 (d, *J* = 7.3 Hz, 1H), 5.34 (broad s, 1H), 4.92 (broad s, 1H), 4.85–4.42 (m, 2H), 4.32 (d, *J* = 17.0 Hz, 1H), 2.90 (dd, *J* = 15.7, 5.7 Hz, 1H), 2.77 (dd, *J* = 13.9, 6.6 Hz, 1H), 2.70 (dd, *J* = 15.7, 2.2 Hz, 1H), 2.41 (dd, *J* = 14.0, 8.8 Hz, 1H), 1.42 (broad s, 9H). ^{13}C NMR (126 MHz, $CDCl_3$): (mix of rotamers) δ 154.5, 145.0, 140.2, 133.3, 129.4, 128.5, 127.7, 126.7, 126.3, 115.3, 79.9, 48.6, 42.8, 37.9, 32.2, 28.6. HRMS: Calc'd $C_{23}H_{26}NO_2$ (M-H) = 348.1964, found 348.1980. IR (film): 3392, 3010, 2978, 2930, 2850, 1761, 1695, 1684, 1448, 1392, 1367, 1245, 1218, 1166, 1123, 1067, 1029, 947, 901 cm^{-1}.

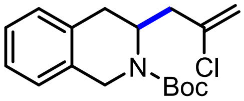

tert-butyl (S)-3-(2-chloroallyl)-3,4-dihydroisoquinoline-2(1H)-carboxylate (**4d**) was synthesized via general procedure 2.4. Isolated 8.6 mg, 14% yield as a colorless oil. ^1H NMR (500 MHz, $CDCl_3$): (mix of rotamers) δ 7.24–7.07 (m, 4H), 5.20 (d, 1H), 5.06-4.96 (m, 1H), 4.95–4.69 (m, 2H), 4.27 (dd, *J* = 24.6, 17.0 Hz, 1H), 3.05 (td, *J* = 15.8, 5.7 Hz, 1H), 2.92–2.64 (m, 1H), 2.56–2.35 (m, 1H), 2.31–2.09 (m, 1H), 1.52-1.43 (m, 9H).^{13}C NMR (126 MHz, $CDCl_3$): (mix of rotamers) δ 139.7, 129.1, 128.5, 126.9, 126.7, 126.6, 126.5, 126.3, 115.0, 80.3, 48.5, 42.8, 42.1, 32.4, 29.9, 29.8, 28.7. HRMS: Calc'd $C_{17}H_{26}ClN_2O_2$ (M+NH_4) = 290.1994, found 290.1996. IR (film): 2977, 2902, 1700, 1684, 1653, 1558, 1534, 1507, 1457, 1219, 1162, 804 cm^{-1}.

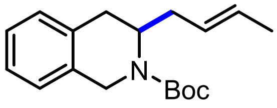

tert-butyl (R,E)-3-(but-2-en-1-yl)-3,4-dihydroisoquinoline-2(1H)-carboxylate (**4e**) was synthesized via general procedure 2.4. Isolated as a colorless oil; 37.0 mg, 65% yield with **3e** and 41.4 mg, 72% yield with **3e'**. ^1H NMR (500 MHz, $CDCl_3$): (mix of rotamers) δ 7.22–7.05 (m, 4H), 5.46—5.30 (m, 2H), 4.87—4.69 (m, 1H), 4.63—4.30 (m, 1H), 4.23 (d, *J* = 17.0 Hz, 1H), 3.02 (dd, *J* = 15.9, 5.8 Hz, 1H), 2.68 (d, *J* = 15.8 Hz, 1H), 2.15 (dt, *J* = 13.8, 6.1 Hz, 1H), 1.96 (t, *J* = 10.0 Hz, 1H), 1.64 (broad s, 3H), 1.50 (broad s, 9H). ^{13}C NMR (126 MHz, $CDCl_3$): (mix of rotamers) δ 155.2, 133.0, 129.6, 129.2, 127.8, 127.7, 126.6, 126.2, 79.7, 50.3, 48.6, 43.1, 42.5, 35.7, 35.2, 33.0, 32.3, 28.6, 18.1. HRMS: Calc'd $C_{18}H_{25}NO_2Na$ (M+Na) = 310.1783, found 310.1785. IR (film): 3356, 2978, 2933, 2253, 1701, 1698, 1694, 1682, 1457, 1423, 1344, 1257, 1219, 1168, 1130, 1025, 968, 910 cm^{-1}.

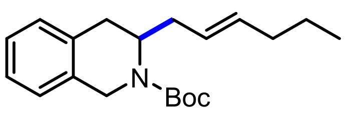

tert-butyl (R,E)-3-(hex-2-en-1-yl)-3,4-dihydroisoquinoline-2(1H)-carboxylate (**4f**) was synthesized via general procedure 2.4. Isolated 48.4 mg, 77% yield as a colorless oil. ^{1}H NMR (500 MHz, $CDCl_3$): δ 7.22–7.05 (m, 4H), 5.40–5.32 (m, 2H), 4.78 (broad s, 1H), 44.64-4.29 (m, 1H), 4.23 (d, *J* = 17.0 Hz, 1H), 3.01 (dd, *J* = 15.8, 5.8 Hz, 1H), 2.69 (d, *J* = 15.8 Hz, 1H), 2.16 (dt, *J* = 13.5, 6.5 Hz, 1H), 2.02-1.90 (m, 3H), 1.49 (s, 9H), 1.35 (h, *J* = 7.3 Hz, 2H), 0.88 (t, *J* = 7.4 Hz, 3H). ^{13}C NMR (126 MHz, $CDCl_3$): δ 155.2, 133.3, 126.7, 126.2, 79.7, 34.8, 28.6, 22.7, 13.7. HRMS: Calc'd $C_{20}H_{30}NO_2$ (M+H) = 316.2277, found 316.2266. IR (film): 3031, 2963, 2930, 2873, 1703, 1695, 1457, 1392, 1368, 1340, 1255, 1219, 1167, 1127, 1020, 969, 938, 905, 857 cm^{-1}.

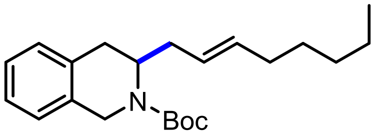

tert-butyl (R,E)-3-(oct-2-en-1-yl)-3,4-dihydroisoquinoline-2(1H)-carboxylate (**4g**) was synthesized via general procedure 2.4. Isolated 55.4 mg, 81% yield as a colorless oil. ^{1}H NMR (500 MHz, $CDCl_3$): δ 7.24–7.03 (m, 4H), 5.42—5.28 (m, 2H), 4.84—4.66 (m, 1H), 4.65–4.31 (m, 1H), 4.23 (d, *J* = 17.0 Hz, 1H), 3.01 (dd, *J* = 15.8, 5.8 Hz, 1H), 2.69 (d, *J* = 15.8 Hz, 1H), 2.21–2.12 (m, 1H), 2.01—1.88 (m, 3H), 1.49 (s, 9H), 1.38—1.16 (m, 6H), 0.89 (t, *J* = 7.0 Hz, 3H). ^{13}C NMR (126 MHz, $CDCl_3$): δ 155.2, 133.6, 133.1, 126.6, 126.4, 126.2, 79.7, 32.7, 31.5, 29.3, 28.6, 22.7, 14.2. HRMS: Calc'd $C_{22}H_{33}NO_2Li$ (M+Li) = 350.2671, found 350.2691. IR (film): 3352, 2956, 2929, 2858, 1700, 1420, 1392, 1367, 1341, 1248, 1166, 1128, 969 cm^{-1}.

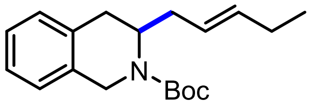

tert-butyl (R,E)-3-(pent-2-en-1-yl)-3,4-dihydroisoquinoline-2(1H)-carboxylate (**4h**) was synthesized via general procedure 2.4. Isolated 48.1 mg, 80% yield as a colorless oil. ^{1}H NMR (500 MHz, $CDCl_3$): (mix of rotamers) δ 7.22–7.05 (m, 4H), 5.46—5.28 (m, 2H), 4.84—4.70 (m, 1H), 4.64—4.30 (m, 1H), 4.23 (d, *J* = 17.0 Hz, 1H), 3.02 (dd, *J* = 15.8, 5.8 Hz, 1H), 2.68 (d, *J* = 15.8 Hz, 1H), 2.16 (dt, *J* = 13.5, 7.0 Hz, 1H), 1.98 (dt, *J* = 12.7, 6.3 Hz, 3H), 1.49 (s, 9H), 0.95 (t, *J* = 7.5 Hz, 3H). ^{13}C NMR (126 MHz, $CDCl_3$): (mix of rotamers) δ 203.7, 183.5, 181.6, 177.6, 175.1, 174.7, 173.9, 128.3, 98.9, 97.1, 91.7, 91.2, 84.2, 83.7, 81.3, 80.8, 77.2, 74.3, 62.4. HRMS: Calc'd $C_{19}H_{27}NO_2$ (M+) = 301.2042, found 301.2054. IR (film): 3352, 3009, 2975, 2933, 1705, 1703, 1697, 1693, 1456, 1392, 1367, 1342, 1255, 1172, 1128, 1029, 939, 864 cm^{-1}.

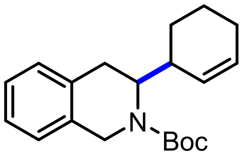

tert-butyl (S)-3-((S)-cyclohex-2-en-1-yl)-3,4-dihydroisoquinoline-2(1H)-carboxylate (**4i**) was synthesized via general procedure 2.4. Isolated 25.4 mg, 41% yield as a colorless oil. ^{1}H NMR (500 MHz, $CDCl_3$): (mix of rotamers) δ 7.14–6.96 (m, 4H), 5.76–5.36 (m, 2H), 4.87 (dd, *J* = 35.4, 16.4 Hz, 1H), 4.29–4.05 (m, 1H), 2.98–2.66 (m, 2H), 2.03 (broad s, 1H), 1.93–1.86 (m, 2H), 1.71–1.48 (m, 3H), 1.46—1.35 (m, 9H), 1.27–1.11 (m, 2H). ^{13}C NMR (126 MHz, $CDCl_3$): δ 159.4, 130.0, 129.3, 129.0, 127.4, 126.3, 126.0, 79.6, 29.6, 28.4, 28.4, 28.3, 28.2, 28.2, 28.0, 28.0. HRMS: Calc'd $C_{20}H_{28}NO_2$ (M+H) = 314.2120, found 314.2118. IR (film): 2928, 2860, 1704, 1689, 1653, 1559, 1502, 1457, 1368, 1219, 1167 cm^{-1}.

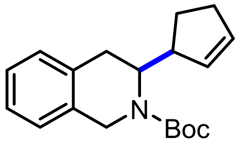

tert-butyl (S)-3-((S)-cyclopent-2-en-1-yl)-3,4-dihydroisoquinoline-2(1H)-carboxylate (**4j**) was synthesized via general procedure 2.4. Isolated 19.7 mg, 33% yield as a colorless oil. ^{1}H NMR (500 MHz, $CDCl_3$): (mix of rotamers) δ 7.22–7.04 (m, 4H), 5.85–5.48 (m, 1H), 5.09–4.54 (m, 2H), 4.37–4.03 (m, 2H), 3.05 (dd, *J* = 16.1, 5.7 Hz, 1H), 2.87–2.68 (m, 2H), 2.46–2.16 (m, 2H), 1.88 (dqd, *J* = 21.1, 8.6, 4.6 Hz, 1H), 1.75–1.62 (m, 1H), 1.49 (s, 9H). ^{13}C NMR (126 MHz, $CDCl_3$): (mix of rotamers) δ 155.2, 133.2, 132.9, 131.8, 129.5, 126.5, 126.2, 79.8, 46.7, 46.4, 38.8, 32.4, 32.0, 31.8, 28.6, 28.6, 28.0. HRMS: Calc'd $C_{19}H_{25}NO_2Na$ (M+Na) = 322.1783, found 322.1794. IR (film): 2974, 2930, 2905, 2848, 1693, 1454, 1392, 1348, 1252, 1219, 1170, 1116, 1097, 978, 913, 882, 763 cm^{-1}.

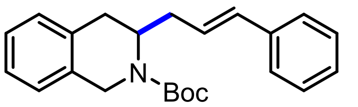

tert-butyl (R)-3-cinnamyl-3,4-dihydroisoquinoline-2(1H)-carboxylate (**4k**) was synthesized via general procedure 2.4. Isolated 69.9 mg, 92% yield, 75:25 E:Z as a colorless oil. ^{1}H NMR (500 MHz, $CDCl_3$): (Mix of E/Z isomers and rotamers) δ 7.41–7.27 (m, 3H), 7.25–6.93 (m, 6H), [6.51 (d, *J* = 11.7 Hz) & 6.33 (d, *J* = 15.9 Hz), ∑1H], 6.25–5.91 (m) & 5.65 (p, *J* = 8.3, 7.8 Hz), ∑1H], 5.04–4.46 (m, 2H), [4.31 (d, *J* = 17.0 Hz) & 4.16–4.09 (m), ∑1H], 3.06 (ddd, *J* = 23.2, 17.1, 4.7 Hz, 1H), 2.90–2.65 (m, 1H), 2.55–2.31 (m, 1H), 2.30–2.13 (m, 1H), 1.56–1.24 (m, 9H). ^{13}C NMR (126 MHz, $CDCl_3$): (Mix of E/Z isomers and rotamers) δ 155.1, 140.0, 137.5, 133.0, 132.3, 131.1, 128.7, 128.6, 128.1, 127.2, 126.7, 126.6, 126.4, 126.2, 116.4, 115.7, 79.9, 69.8, 55.0, 53.9, 52.8, 52.7, 52.5, 52.4, 50.8, 50.5, 48.6, 43.2, 43.0, 42.8, 42.5, 36.3, 35.8, 33.1, 32.6, 31.3, 31.0, 30.7, 29.8, 28.7, 28.6, 28.3. HRMS: Calc'd $C_{23}H_{27}NO_2Na$ (M+Na) = 372.1939, found 372.1938. IR (film): 3065, 3027, 2977, 2930, 1699, 1457, 1419, 1405, 1368, 1340, 1255, 1219, 1166, 1123, 912 cm^{-1}.

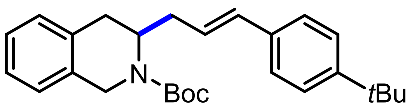

tert-butyl (R,E)-3-(3-(4-(tert-butyl)phenyl)allyl)-3,4-dihydroisoquinoline-2(1H)-carboxylate (**4l**) was synthesized via general procedure 2.4. Isolated 64.4 mg, 79% yield, 68:32 E:Z as a colorless oil. ^{1}H NMR (500 MHz, $CDCl_3$): (Mix of E/Z isomers and rotamers) δ 7.37–7.27 (m, 3H), 7.25–6.97 (m, 5H), [6.48 (d, *J* = 11.7 Hz) & 6.31 (d, *J* = 15.9 Hz), ∑1H], [6.23–5.94 (m) & 5.62 (dt, *J* = 13.0, 7.6 Hz), ∑1H], 5.03–4.41 (m, 2H), [4.37–4.25 (m) & 4.18–4.10 (m), ∑1H], 3.33–2.99 (m, 1H), 2.89–2.67 (m, 1H), 2.56–2.33 (m, 1H), 2.28–2.14 (m, 1H), 1.55—1.45 (m, 9H), 1.35–1.27 (m, 9H). ^{13}C NMR (126 MHz, $CDCl_3$): (Mix of E/Z isomers and rotamers) δ 154.9, 150.0, 140.0, 138.4, 134.5, 132.7, 131.9, 130.6, 129.5, 129.5, 128.2, 127.7, 127.3, 127.2, 126.5, 126.3, 126.1, 126.0, 125.5, 125.2, 124.8, 115.9, 115.1, 79.7, 66.9, 53.7, 52.4, 52.1, 51.9, 42.9, 42.3, 34.3, 31.3, 31.2, 30.4, 29.6, 28.5, 28.4. HRMS: Calc'd $C_{27}H_{35}NO_2Na$ (M+Na) = 428.2565, found 428.2585. IR (film): 3008, 2966, 2905, 2868, 2249, 1699, 1684, 1457, 1400, 1394, 1366, 1269, 1255, 1219, 1168, 1121, 1016, 968, 911, 857 cm^{-1}.

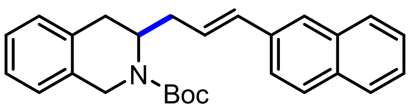

tert-butyl (R,E)-3-(3-(naphthalen-2-yl)allyl)-3,4-dihydroisoquinoline-2(1H)-carboxylate (**4m**) was synthesized via general procedure 2.4. Isolated 69.1 mg, 86% yield, 91:9 E:Z as a yellow oil. ^{1}H NMR (500 MHz, $CDCl_3$): (Mix of E/Z isomers and rotamers) δ 7.83–7.74 (m, 3H), 7.66 (s, 1H), 7.57 (dd, *J* = 8.6, 1.7 Hz, 1H), 7.49–7.41 (m, 2H), 7.25–7.12 (m, 4H), [6.67 (d, *J* = 11.5 Hz) & 6.49 (d, *J* = 15.8 Hz), ∑1H], 6.40–6.21 (m, 1H), 5.16–4.47 (m, 2H), [4.36 (d, *J* = 17.0 Hz) & 4.17 (d, *J* = 15.4 Hz), ∑1H], 3.10 (td, *J* = 16.1, 5.4 Hz, 1H), 2.90–2.69 (m, 1H), 2.62–2.41 (m, 1H), 2.30 (broad s, 1H), [1.69-1.62 (m), & 1.53-1.42 (m), ∑9H]. ^{13}C NMR (126 MHz, $CDCl_3$): (Mix of E/Z isomers and rotamers) δ 155.2, 133.7, 132.9, 128.2, 128.0, 127.7, 126.8, 126.4, 126.3, 125.7, 123.5, 80.0, 52.8, 52.6, 50.6, 50.5, 48.6, 43.2, 43.0, 42.9, 42.6, 36.4, 36.0, 33.2, 32.7, 29.8, 28.7, 28.4. HRMS: Calc'd $C_{27}H_{29}NO_2Na$ (M+Na) = 422.2096, found 422.2117. IR (film): 3056, 3007, 2975, 2928, 2846, 1690, 1457, 1405, 1364, 1325, 1241, 1170, 1118, 1096, 1007, 963, 909, 859 cm^{-1}.

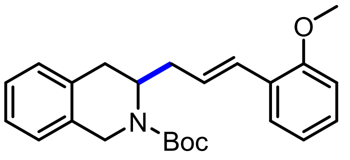

tert-butyl (R,E)-3-(3-(2-methoxyphenyl)allyl)-3,4-dihydroisoquinoline-2(1H)-carboxylate (**4n**) was synthesized via general procedure 2.4. Isolated 54.3 mg, 72% yield, 76:24 E:Z as a colorless oil. ^{1}H NMR (500 MHz, $CDCl_3$): (mix of E/Z isomers and rotamers) δ [7.44-7.36 (m) & 7.25-7.08 (m) & 7.04-6.79 (m), ∑8H], [6.67 (d) & 6.59 (d), ∑1H], [6.35-6.0 (m) & 5.75-5.66 (m), ∑1H], 5.10-4.56 (m, 2H), [4.35-4.27 (m) & 4.19-4.12 (m), ∑1H], [3.82 (s) & 3.80 (s) & 3.67 (s) & 3.61 (s), ∑3H], 3.12-2.97 (m, 1H), 2.88-2.65 (m, 1H), 2.48-2.34 (m, 1H), 2.37-2.16 (m, 1H), [1.55-1.44 (m) & 1.34-1.24 (m), ∑9H]. ^{13}C NMR (126 MHz, $CDCl_3$): (mix of E/Z isomers and rotamers) δ 156.4, 155.1, 139.7, 138.8, 133.1, 129.9, 129.7, 129.3, 128.2, 127.8, 127.6, 126.7, 126.5, 126.3, 126.2, 126.0, 121.0, 120.7, 120.1, 116.3, 111.1, 110.9, 79.8, 55.5, 55.5, 55.3, 55.2, 43.0, 42.3, 31.3, 30.9, 28.7,

28.5, 28.3. HRMS: Calc'd $C_{24}H_{28}NO_3$ (M-H) = 378.2069, found 378.2041. IR (film): 2974, 2836, 1695, 1688, 1554, 1489, 1457, 1395, 1363, 1243, 1219, 1169, 1117, 1096, 1028, 1006, 971 cm^{-1}.

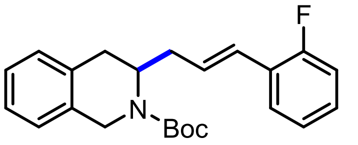

tert-butyl (R,E)-3-(3-(2-fluorophenyl)allyl)-3,4-dihydroisoquinoline-2(1H)-carboxylate (**4o**) was synthesized via general procedure 2.4. Isolated 59.7 mg, 81% yield, >95:5 E:Z as a light yellow oil. ^{1}H NMR (500 MHz, $CDCl_3$): (mix of E/Z isomers and rotamers) δ [7.44-7.32 (m) & 7.23-7.10 (m) & 7.09-6.92 (m), ∑8H], 6.46 (d, 1H), 6.30-6.18 (m, 1H), 5.06-4.46 (m, 2H), 4.34-4.25 (m, 1H), 3.14-2.98 (m, 1H), 2.80-2.64 (m, 1H), 2.47-2.32 (m, 1H), 2.30-2.16 (m, 1H), [1.52-1.41 (m) & 1.33-1.22 (m), ∑9H]. ^{13}C NMR (126 MHz, $CDCl_3$): (mix of E/Z isomers and rotamers) δ160 (d, *J* = 246.7 Hz), 155.1, 132.9, 130.0, 129.9, 129.7, 129.4 (d, *J* = 16.6 Hz), 127.3 (d, *J* = 4.5 Hz), 126.8, 126.4, 124.6 (d, 3.7 Hz), 124.1 (d, 4.0 Hz), 115.7, 115.6, 79.9, 63.6, 50.4, 48.6, 43.3, 42.7, 36.7, 36.2, 33.1, 32.7, 28.6. HRMS: Calc'd $C_{23}H_{25}FNO_2$ (M-H) = 366.1869, found 366.1845. IR (film): 2975, 2930, 2844, 1695, 1684, 1559, 1487, 1395, 1355, 1319, 1219, 1169, 1120, 1096, 1007, 968 cm^{-1}.

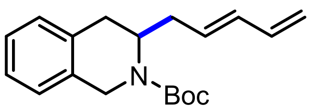

tert-butyl (R,E)-3-(penta-2,4-dien-1-yl)-3,4-dihydroisoquinoline-2(1H)-carboxylate (**4p**) was synthesized via general procedure 2.4. Isolated 43.4 mg, 72% yield, 67:33 E:Z as a colorless oil. ^{1}H NMR (500 MHz, $CDCl_3$): (Mix of E/Z-isomers) δ 7.23–7.04 (m, 4H), 6.28 (dt, *J* = 17.0, 10.3 Hz, 1H), [6.06 (t) & 5.98 (dd), ∑1H], 5.72– 5.36 (m, 1H), 5.21–4.93 (m, 2H), 4.87–4.69 (m, 1H), 4.69—4.34 (m, 1H), [4.26 (d, *J* = 15, Hz) & 4.25 (d, *J* = 15 Hz), ∑1H], 3.03 (ddd, *J* = 16.7, 11.1, 5.8 Hz, 1H), 2.69 (ddd, *J* = 15.8, 5.7, 2.2 Hz, 1H), [2.35 (dtd, *J* = 14.5, 7.3, 1.6 Hz) & 2.26 (dt, *J* = 14.9, 7.7 Hz), ∑1H], [2.19 (broad s) & 2.08 (broad s), ∑1H], [1.50 (s) & 1.49 (s), ∑9H]. ^{13}C NMR (126 MHz, $CDCl_3$): (Mix of E/Z isomers) δ 155.1, 137.1, 133.4, 132.9, 132.1, 131.6, 131.2, 129.5, 129.2, 126.7, 126.3, 115.9, 115.6, 79.8, 50.3, 48.5, 43.2, 42.7, 35.7, 35.3, 33.1, 32.4, 28.6. HRMS: Calc'd $C_{19}H_{29}N_2O_2$ ($M+NH_4$) = 317.2229, found 317.2244. IR (film): 2975, 2930, 2846, 2359, 1700, 1684, 1635, 1457, 1407, 1395, 1354, 1340, 1254, 1243, 1219, 1169, 1119, 1096, 1006, 904 cm^{-1}.

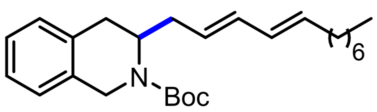

tert-butyl (R)-3-((2E,4E)-dodeca-2,4-dien-1-yl)-3,4-dihydroisoquinoline-2(1H)-carboxylate (**4q**) was synthesized via general procedure 2.4. Isolated 36.1 mg, 45% yield as a colorless oil. ^{1}H NMR (500 MHz, $CDCl_3$): (Mix of E/Z isomers and rotamers) δ 7.23-7.02 (m, 4H), [6.37-6.15 (m) & 6.04-5.86 (m), ∑2H], 5.70-5.19 (m, 2H), 5.10-4.69 (m, 2H), 4.66-4.35 (m, 1H), 4.30-4.05 (m, 1H), 3.09-2.82 (m, 2H), 2.76-2.61 (m, 1H), 2.36-1.91 (m, 2H), 1.52-1.46 (m, 9H), 1.37-1.57 (m, 10H), 0.92-0.83 (m, 3H). ^{13}C NMR (126 MHz, $CDCl_3$): δ 155.0, 139.4, 138.9, 133.0, 132.9, 132.8, 130.1, 129.4, 129.3, 129.1, 128.2, 126.5, 126.3, 126.1, 126.0, 125.9, 115.4, 114.9, 79.7, 51.2, 50.8, 50.2, 49.6, 49.3, 48.4, 43.1, 42.5, 32.8, 32.6, 31.9, 31.8, 29.7, 29.4, 29.2, 29.2, 28.5, 27.7, 22.7. HRMS: Calc'd $C_{26}H_{39}NO_2Li$ (M+Li) = 404.3141, found 404.3139. IR (film): 2956, 2926, 2854, 1704, 1652, 1559, 1507, 1457, 1395, 1219, 1170, 1117 cm^{-1}.

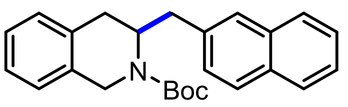

tert-butyl (R)-3-(naphthalen-2-ylmethyl)-3,4-dihydroisoquinoline-2(1H)-carboxylate (**4r**) was synthesized via general procedure 2.4. Isolated 53.0 mg, 71% yield as a colorless oil. ^{1}H NMR (500 MHz, $CDCl_3$): (mix of rotamers) δ 7.84–7.74 (m, 3H), 7.54 (broad s, 1H), 7.45 (h, *J* = 6.7 Hz, 2H), 7.38–7.27 (m, 1H), 7.25–7.16 (m, 3H), 7.15–7.11 (m, 1H), 4.93–4.57 (m, 2H), 4.44 (broad d, 1H), 3.03-2.89 (m, 2H), 2.67 (broad d, 2H), 1.46–1.34 (m, 9H). ^{13}C NMR (126 MHz, $CDCl_3$): δ 155.0, 133.7, 132.4, 128.2, 127.8, 127.7, 127.6, 126.9, 126.5, 126.0, 125.5, 80.0, 52.2, 43.2, 39.1, 32.6, 28.6, 28.5. HRMS: Calc'd $C_{25}H_{27}NO_2$ (M+) = 373.2042, found 373.2046. IR (film): 3052, 3010, 2976, 2923, 2856, 1700, 1507, 1457, 1392, 1367, 1243, 1218, 1167, 1123, 1016, 931, 891 cm^{-1}.

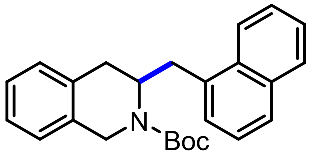

tert-butyl (R)-3-(naphthalen-1-ylmethyl)-3,4-dihydroisoquinoline-2(1H)-carboxylate (**4s**) was synthesized via general procedure 2.4. Isolated 22.9 mg, 35% yield as a colorless oil. ^{1}H NMR (500 MHz, $CDCl_3$): (mix of rotamers) δ 8.28—8.06 (m, 1H), 7.91–7.82 (m, 1H), 7.75 (d, *J* = 8.3 Hz, 1H), 7.50 (q, *J* = 7.5, 6.9 Hz, 2H), 7.36 (t, *J* = 7.6 Hz, 1H), 7.30—7.20 (m, 3H), 7.15—7.06 (m, 2H), 5.02—4.68 (m, 2H), 4.52 (d, *J* = 17.0 Hz, 1H), 3.42—3.16 (m, 1H), 3.03-2.78 (m, 2H), 2.71–2.46 (m, 1H), 1.57–1.19 (m, 9H). ^{13}C NMR (126 MHz, $CDCl_3$): δ (mix of rotamers) 155.1, 135.1, 134.1, 133.2, 132.7, 132.4, 129.4, 128.9, 127.7, 127.4, 126.8, 126.5, 125.7, 124.1, 79.9, 50.4, 43.1, 36.0, 32.8, 28.6, 28.4. HRMS: Calc'd $C_{25}H_{28}NO_2$ (M+H) = 374.2120, found 374.2123. IR (film): 2975, 2930, 1690, 1457, 1457, 1391, 1363, 1291, 1219, 1165, 1118 cm^{-1}.

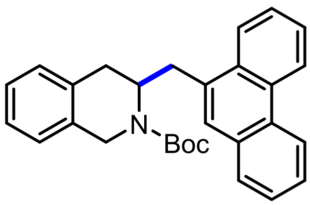

tert-butyl (R)-3-(phenanthren-9-ylmethyl)-3,4-dihydroisoquinoline-2(1H)-carboxylate (**4t**) was synthesized via general procedure 2.4. Isolated 66.3 mg, 78% yield as a colorless oil. ^{1}H NMR (500 MHz, $CDCl_3$): (mix of rotamers) δ 8.77 (d, *J* = 8.0 Hz, 1H), 8.68 (d, *J* = 8.1 Hz, 1H), 8.44–8.21 (m, 1H), 7.79 (d, *J* = 7.7 Hz, 1H), 7.69 (d, *J* = 7.6 Hz, 2H), 7.61 (dt, *J* = 22.1, 7.2 Hz, 2H), 7.44–7.27 (m, 4H), 7.18–7.05 (m, 1H), 5.13–4.71 (m, 2H), 4.59 (d, *J* = 16.3 Hz, 1H), 3.54–3.27 (m, 1H), 3.04–2.80 (m, 2H), 2.69 (d, *J* = 16.0 Hz, 1H), 1.62–1.24 (m, 9H). ^{13}C NMR (126 MHz, $CDCl_3$): (mix of rotamers) δ 155.0, 133.3, 131.8, 131.4, 130.9, 130.0, 129.3, 128.2, 126.8, 126.6, 126.4, 124.6, 123.4, 122.5, 80.0, 50.0, 43.3, 36.5, 32.7, 28.7, 28.6, 28.5, 28.4. HRMS: Calc'd $C_{29}H_{29}NO_2$ (M+) = 423.2198, found 423.2199. IR (film): 2976, 2930, 1689, 1497, 1476, 1452, 1392, 1365, 1248, 1219, 1168, 1121, 1095, 1014 cm^{-1}.

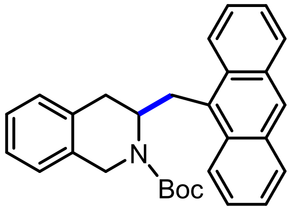

tert-butyl (S)-3-(anthracen-9-ylmethyl)-3,4-dihydroisoquinoline-2(1H)-carboxylate (**4u**) was synthesized via general procedure 2.4. Isolated 17.4 mg, 21% yield as a yellow oil. ^{1}H NMR (500 MHz, $CDCl_3$): (mix of rotamers) δ 8.38 (s, 1H), 8.00 (d, *J* = 8.4 Hz, 3H), 7.45 (t, *J* = 7.4 Hz, 2H), 7.40 (d, *J* = 7.2 Hz, 2H), 7.32 (d, *J* = 6.4 Hz, 2H), 7.25–7.20 (m, 2H), 6.92 (d, *J* = 7.2 Hz, 1H), 5.01 (d, *J* = 28.8 Hz, 2H), 4.62 (d, *J* = 17.2 Hz, 1H), 3.81–3.63 (m, 2H), 2.93-2.72 (m, 1H), 2.50-2.31 (m, 1H), 1.63-1.32 (m, 9H). ^{13}C NMR (126 MHz, $CDCl_3$): (mix of rotamers) δ 154.8, 134.3, 133.0, 131.7, 131.0, 130.6, 130.1, 129.2, 126.7, 126.6, 125.6, 125.0, 124.9, 51.1, 43.3, 32.3, 30.0, 28.6. HRMS: Calc'd $C_{29}H_{28}NO_2$ (M-H) = 422.2120, found 422.2135. IR (film): 2975, 2364, 1868, 1730, 1684, 1550, 1502, 1429, 1387, 1218, 1167, 1117, 1005 cm^{-1}.

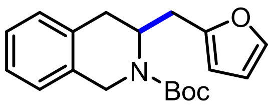

tert-butyl (S)-3-(furan-2-ylmethyl)-3,4-dihydroisoquinoline-2(1H)-carboxylate (**4v**) was synthesized via general procedure 2.4. Isolated 23.9 mg, 38% yield as a colorless oil. ^{1}H NMR (500 MHz, $CDCl_3$): (mix of rotamers) δ 7.36 (s, 1H), 7.23–7.08 (m, 5H), 6.35-6.22 (m, 1H), 4.86–4.68 (m, 1H), 4.62-4.43 (m, 1H), 4.29 (d, *J* = 17.0 Hz, 1H), 3.00 (dd, *J* = 15.9, 5.7 Hz, 1H), 2.68 (d, *J* = 15.8 Hz, 1H), 2.60 (dd, *J* = 14.5, 7.0 Hz, 1H), 2.42—2.27 (m, 1H), 1.48 (d, 9H).^{13}C NMR (126 MHz, $CDCl_3$): (mix of rotamers) δ 155.0, 142.9, 140.0, 133.0, 128.8, 126.9, 126.6, 121.6, 111.4, 80.0, 51.0, 49.2, 43.4, 42.8, 32.6, 32.0, 28.6, 28.6, 27.9, 27.1. HRMS: Calc'd $C_{19}H_{23}NO_3Na$ (M+Na) = 336.1576, found 336.1566. IR (film): 2878, 2930, 1764, 1692, 1456, 1411, 1367, 1245, 1164, 1121, 1011 cm^{-1}.

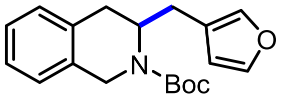

tert-butyl (S)-3-(furan-3-ylmethyl)-3,4-dihydroisoquinoline-2(1H)-carboxylate (**4w**) was synthesized via general procedure 2.4. Isolated 17.7 mg, 28% yield as a colorless oil. ^{1}H NMR (500 MHz, CD_3CN): (mix of rotamers) δ 7.42 (s, 1H), 7.23–7.08 (m, 5H), 6.35 (s, 1H), 4.76 (d, *J* = 17.1 Hz, 1H), 4.66—4.51 (m, 1H), 4.23 (d, *J* = 16.9 Hz, 1H), 2.99 (dd, *J* = 16.2, 5.8 Hz, 1H), 2.70 (dd, *J* = 16.0, 2.4 Hz, 1H), 2.54 (dd, *J* = 14.4, 8.4 Hz, 1H), 2.38 (dd, *J* = 14.5, 6.8 Hz, 1H), 1.39 (s, 9H). ^{13}C NMR

(126 MHz, CD_3CN): (mix of rotamers) δ 155.5, 143.9, 141.0, 134.3, 133.9, 130.1, 127.6, 127.0, 122.9, 112.5, 80.0, 51.6, 42.8, 33.5, 28.3. HRMS: Calc'd $C_{19}H_{22}NO_3$ (M-H) = 312.1600, found 312.1597. IR (film): 2976, 2930, 1771, 1734, 1700, 1684, 1653, 1559, 1456, 1419, 1395, 1363, 1244, 1219, 1165, 1097, 1010, 954 cm^{-1}.

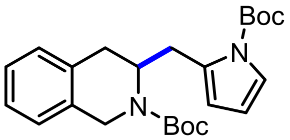

tert-butyl (S)-3-((1-(tert-butoxycarbonyl)-1H-pyrrol-2-yl)methyl)-3,4-dihydroisoquinoline-2(1H)-carboxylate (**4x**) was synthesized via general procedure 2.4. Isolated 21.0 mg, 25% yield as a colorless oil. ^1H NMR (500 MHz, $CDCl_3$): (mix of rotamers) δ 7.24–7.05 (m, 5H), [6.10–5.80 (m) & 5.72 – 5.35 (m), ∑2H], 5.10–4.64 (m, 2H), 4.60–4.19 (m, 1H), 3.37–2.64 (m, 4H), 1.65–1.48 (m, 9H), 1.41—1.21 (m, 9H). ^{13}C NMR (126 MHz, $CDCl_3$): (mix of rotamers) δ 155.3, 155.2, 150.4, 149.6, 132.7, 129.4, 126.5, 126.5, 126.3, 121.3, 113.6, 110.5, 83.5, 49.4, 42.3, 34.1, 31.0, 28.6, 28.4, 28.3, 28.2, 16.5. HRMS: Calc'd $C_{24}H_{32}N_2O_4Na$ (M+Na) = 435.2260, found 435.2274. IR (film): 2978, 2931, 2344, 1734, 1700, 1663, 1635, 1534, 1498, 1457, 1363, 1324, 1219, 1162, 1125 cm^{-1}.

7. References

1. K. C. Cartwright, J. A. Tunge *ACS Catal.* 2018, **8**, 12, 11801-11806.

2. J. Luo, J. Zhang *ACS Catal.* 2016, **6**, 2, 873-877.

3. B. M. Trost, J. R. Miller, C. M. Hoffman, *J. Am. Chem. Soc*. 2011, **133**, 8165-8167.

4. D. F. Shriver, M. A. Drezdn, *The Manipulation of Air Sensitive Compounds.* 2nd ed. Wiley & Sons: New York, NY (1986).

5. H. Yokoi, T. Nakano, W. FuJita, K. Ishiguro, Y. Sawaki, *J. Am. Chem. Soc.* 1998, **120**, 48, 12453-12458.

6. M. R. Churchill, R. Mason, *Nature*, 1964, **204**, 777.

7. J. Grignon, C. Servens, M. Pereyre, *Journal of Organometallic Chemistry* 1975, **96**, 225-35.

8. S. B. Lang, K. O'Nele, J. A. Tunge, Chem. Eur. J. 2015, 51, 18589-18593.

9. Y.-G. Suh, J. Jang, H. Yun, S. M. Han, D. Shin, J.-K. Jung, J.-W. Jung *Org. Lett.* 2011, **13**, 21, 5920-5923.

10. K. P. Melnykov, A. N. Artemenko, B. O. Ivanenko, Y. M. Sokolenko, P. S. Nosik, E. N. Ostapchuk, O. O. Grygorenko, D. M. Volochnyuk, S. V. Ryabukhin *CS Omega* 2019, **4**, 4, 7498-7515.

11. C. Romano, C. Mazet *J. Am. Chem. Soc*. 2018, **140**, 13, 4743-4750.

12. K.-F. Hu, X.-S. Ning, J.-P. Qu, Y.-B. Kang *J. Org. Chem*. 2018, **83**, 18, 11327-11332.

13. P. P. Singh, S. Gudup, H. Aruri, U. Singh, S. Ambala, M. Yadav, S. D. Sawant, R. A. Vishwakarma *Org. Biomol. Chem.*, 2012, **10**, 1587-1597.

14. W. Sittiwong, M. W. Richardson, C. E. Schiaffo, T. J. Fisher, P. H. Dussault *Beilstein J. Org. Chem*. 2013, **9**, 1526–1532.

15. J. Holz, C. Pfeffer, H. Zuo, D. Beierlein, G. Richter, E. Klemm, R. Peters *Angew. Chem. Int. Ed*. 2019, **58**, 10330–10334.

16. S. H. Kima, C. Shina, A. N. Paea, H. Y. Koha, M. H. Changa, B. Y. Chung, Y. S. Cho *Synthesis* 2004, **10**, 1581-1584.

17. S. Datta, C.-L. Chang, K.-L. Yeh, R.-S. Liu *J. Am. Chem. Soc.* 2003, **125**, 31, 9294-9295.

18. G.-Z. Wang, *J.* *J*iang, X.-S. Bu, *J.-J.* Dai, *J.* Xu, Y. Fu, H.-*J.* Xu *Org. Lett.* 2015, **17**, 15, 3682-3685.

19. L. Gonnard, A. GuØrinot, *J.* Cossy *Chem. Eur. J.* 2015, **21**, 12797–12803.

20. T. Ma*J*i, *J.* A. Tunge *Organic Letters*, 2015, **17**(19), 4766-4769.

21. K. Takizawa, T. Sekino, S. Sato, T. Yoshino, M. Ko*J*ima, S. Matsunaga *Angew. Chem. Int. Ed*. 2019, **58**, 9199–9203.

22. Y. Zhou, B. Breit, *Chem. Eur. J.* 2017, **23**, 18156–18160.

23. H. Chen, X. *J*ia, Y. Yu, Q. Qian, H. Gong, *Angew. Chem. Int. Ed.* 2017, **56**, 13103–13106.

24. *J*. Nugent, E. Matousova, M. G. Banwell, *J. Org. Chem.* 2017, **82**, 12569-12589.

25. *J*.-P. Genet, S. *J*uge, S. Achi, S. Mallart, *J*. R. Montes, G. Levif *Tetrahedron* 1988, **44**, 5263-5275.

26. K.-Y. Ye, X. Zhang, L.-X. Dai, S.-L. You, *J. Org. Chem.* 2014, **79**, 24, 12106-12110.

27. Z. Mi, *J*. Tang, Z. Guan, W. Shi, H. Chen, *Eur. J. Org. Chem*. 2018, 4479–4482.

28. G. Blessley, P. Holden, M. Walker, *J*. M. Brown, V. Gouverneur *Org. Lett.* 2012, **14**, 11, 2754-2757.

29. I. Franzoni, L. Guénéeb, C. Mazet, *Org. Biomol. Chem.*, 2015, **13**, 6338-6343.

30. T. Mukai, K. Hirano, T. Satoh, M. Miura *Org. Lett.* 2010, **12**, 6, 1360-1363.

8. Spectral Data

(3q)

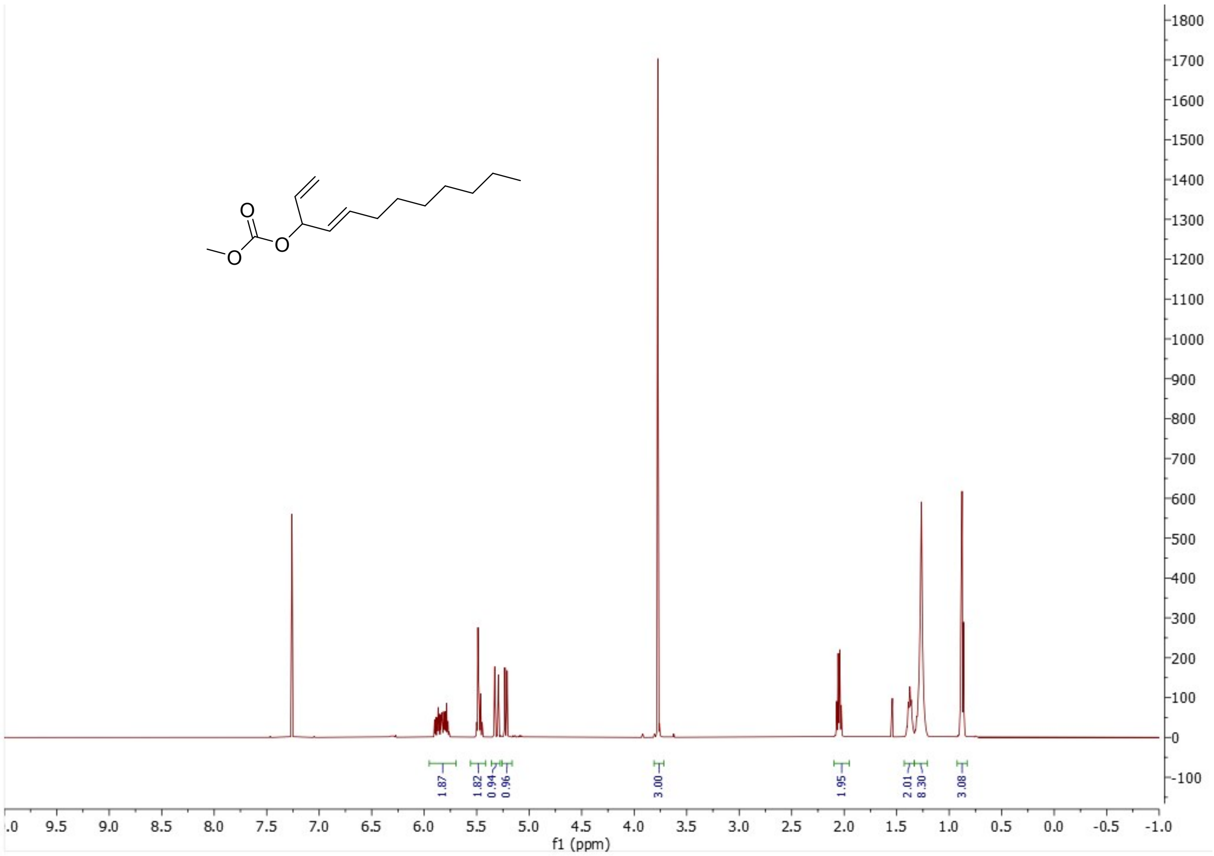
1.87
1.82
0.94
0.96
3.00
1.95
2.01
8.30
3.08
f1 (ppm)

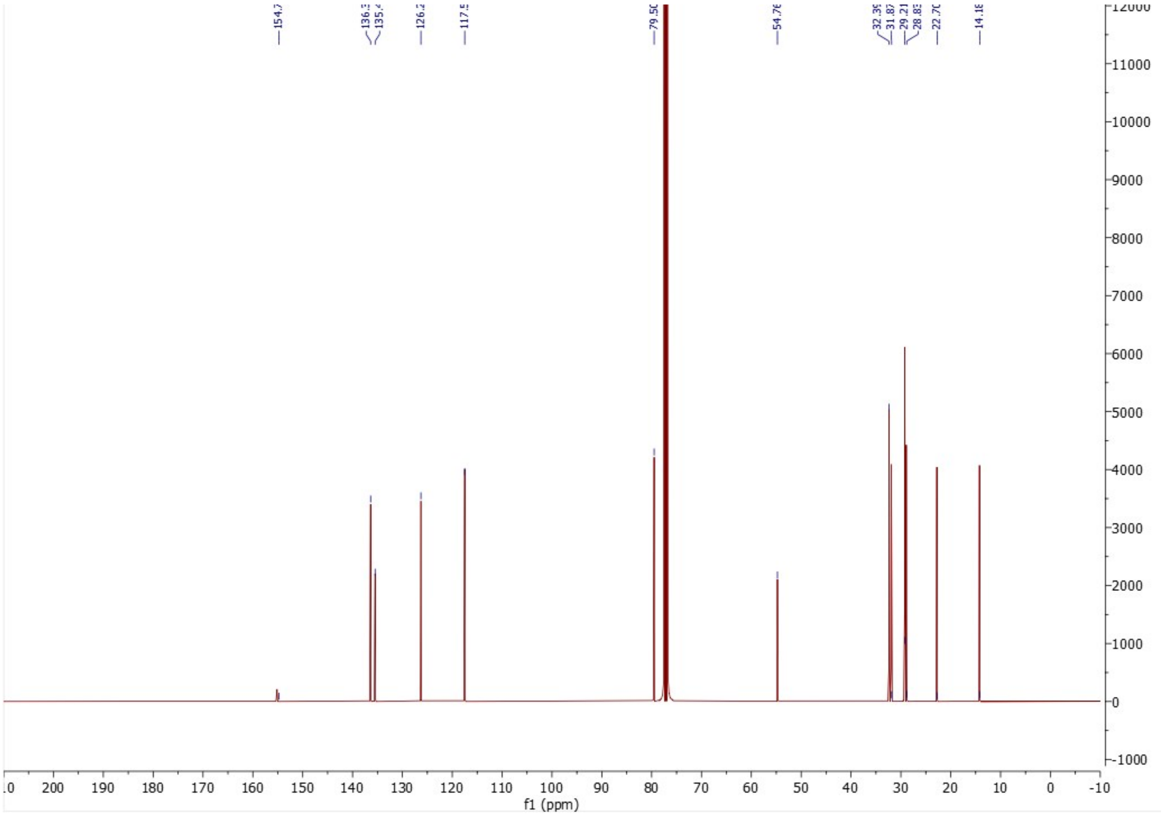
117.5
126.2
79.50
54.76
32.39
31.87
29.21
28.83
22.70
14.18
f1 (ppm)

(3t)

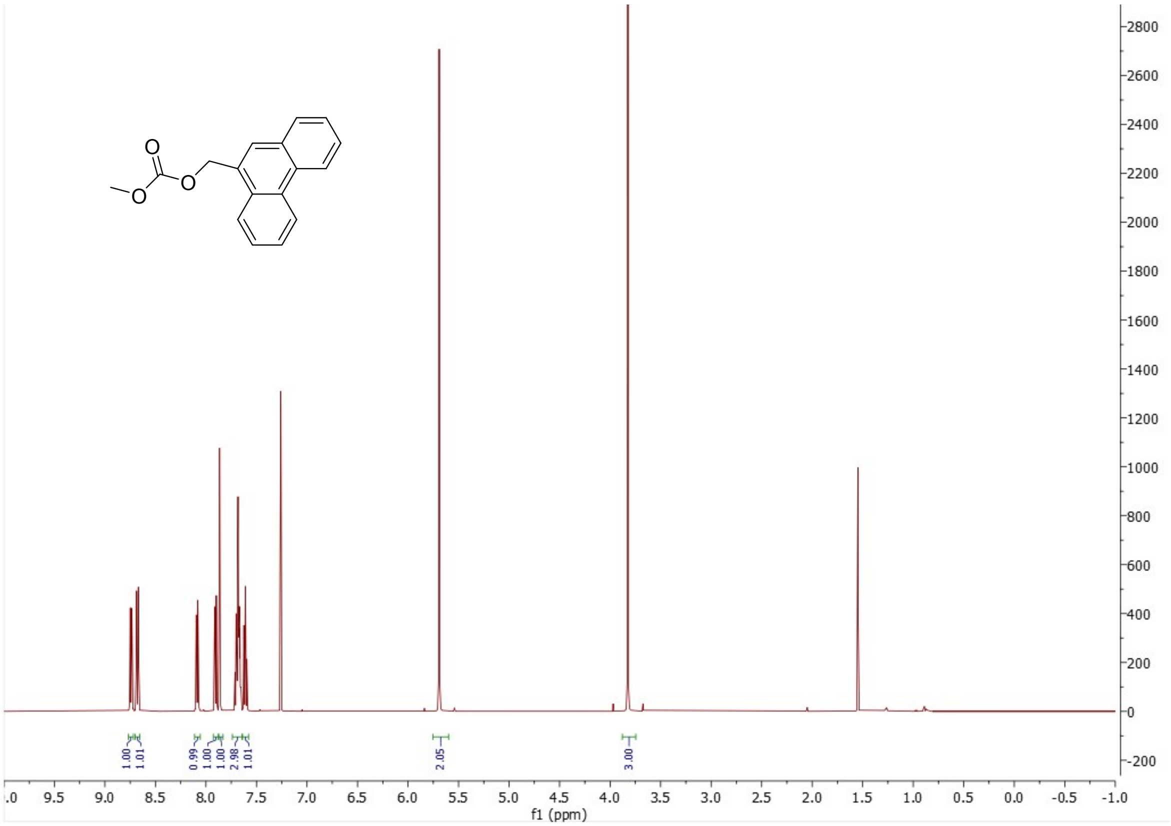

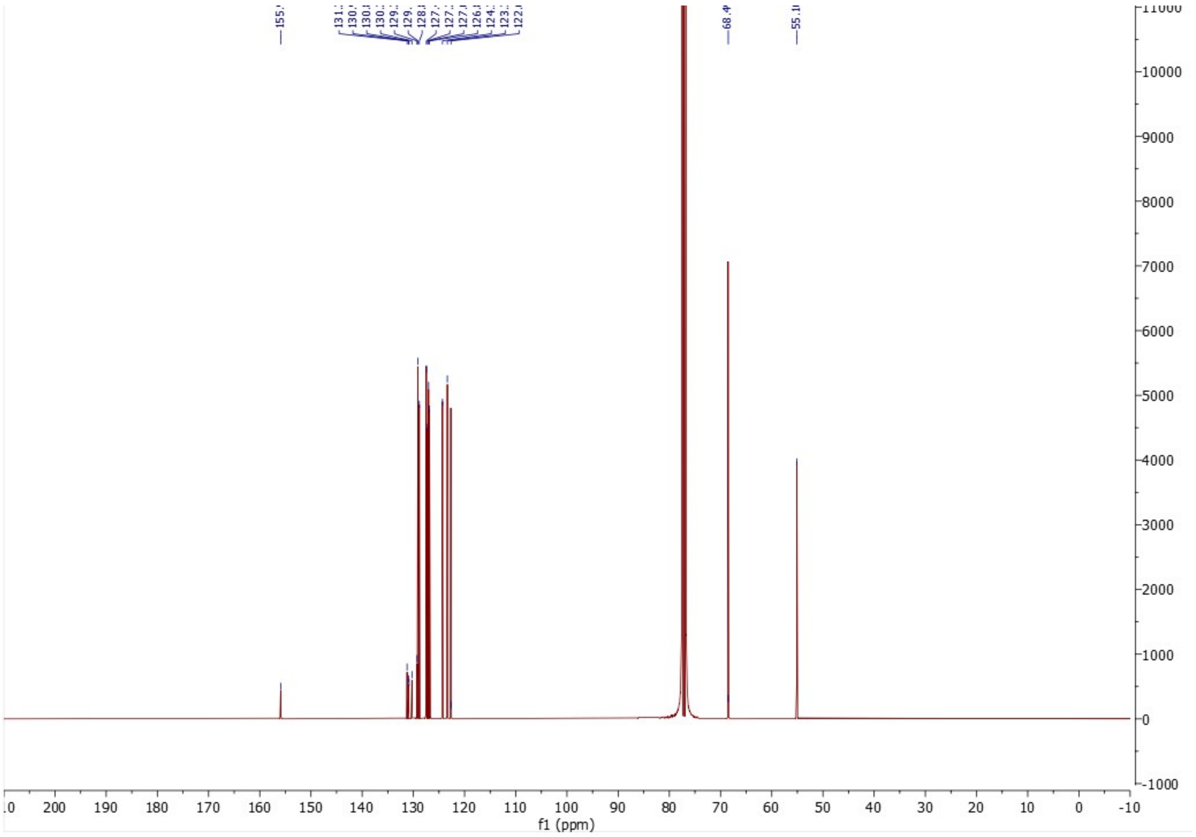

(3x)

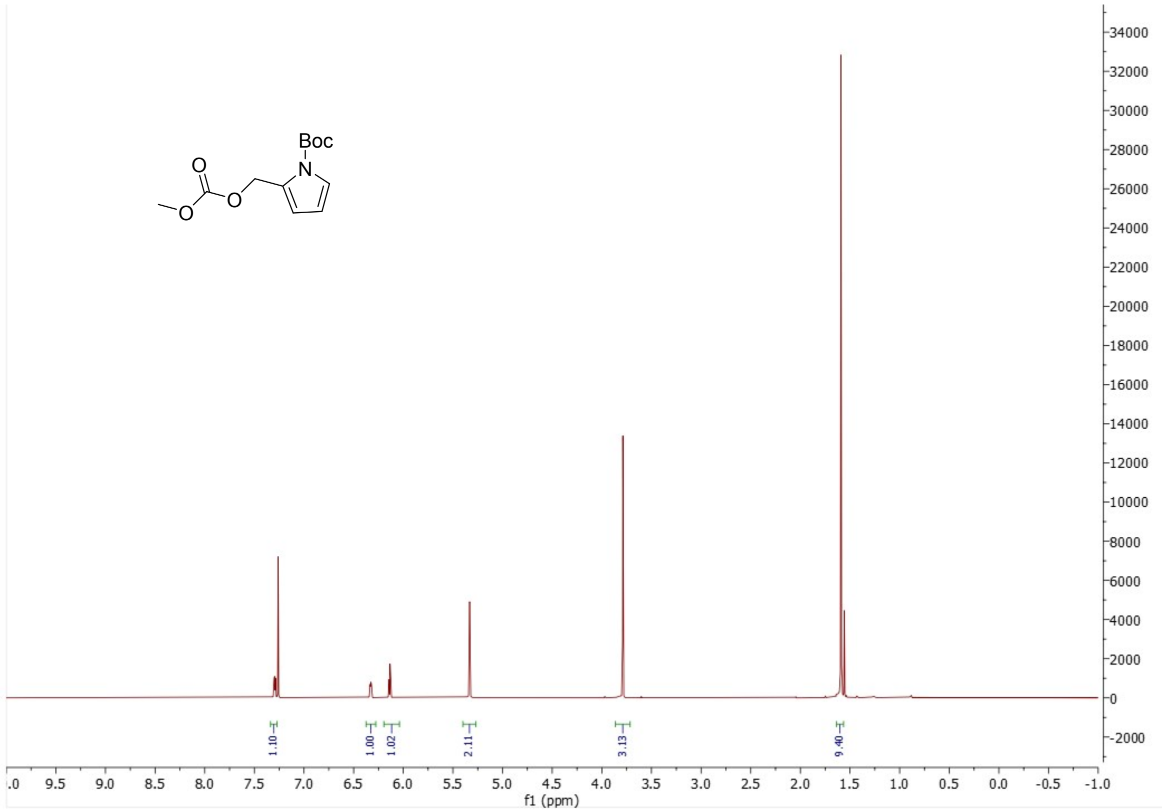
Boc
N
O
O
O
1.10
1.00
1.02
2.11
3.13
9.40
f1 (ppm)

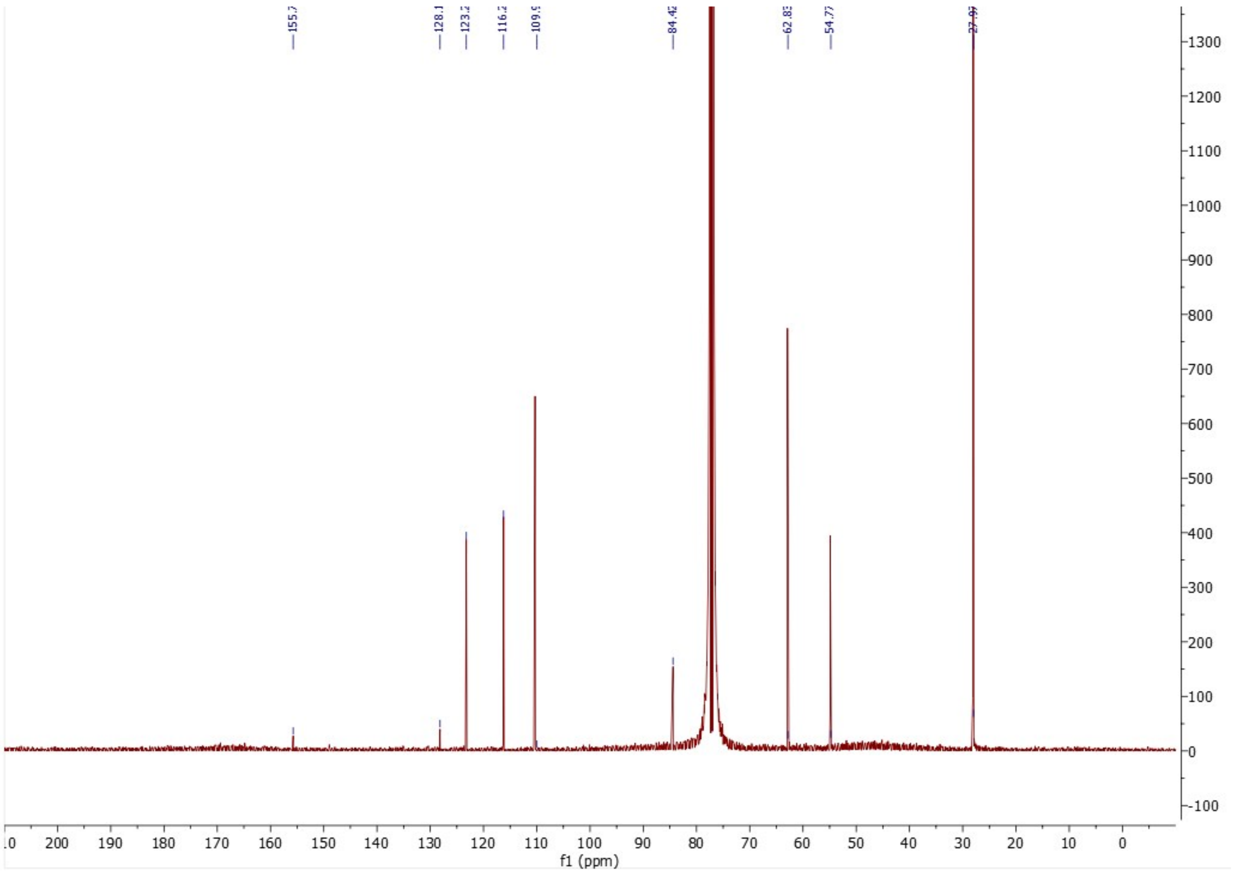
155.7
128.1
123.2
109.9
84.42
62.83
54.77
f1 (ppm)

(2b)

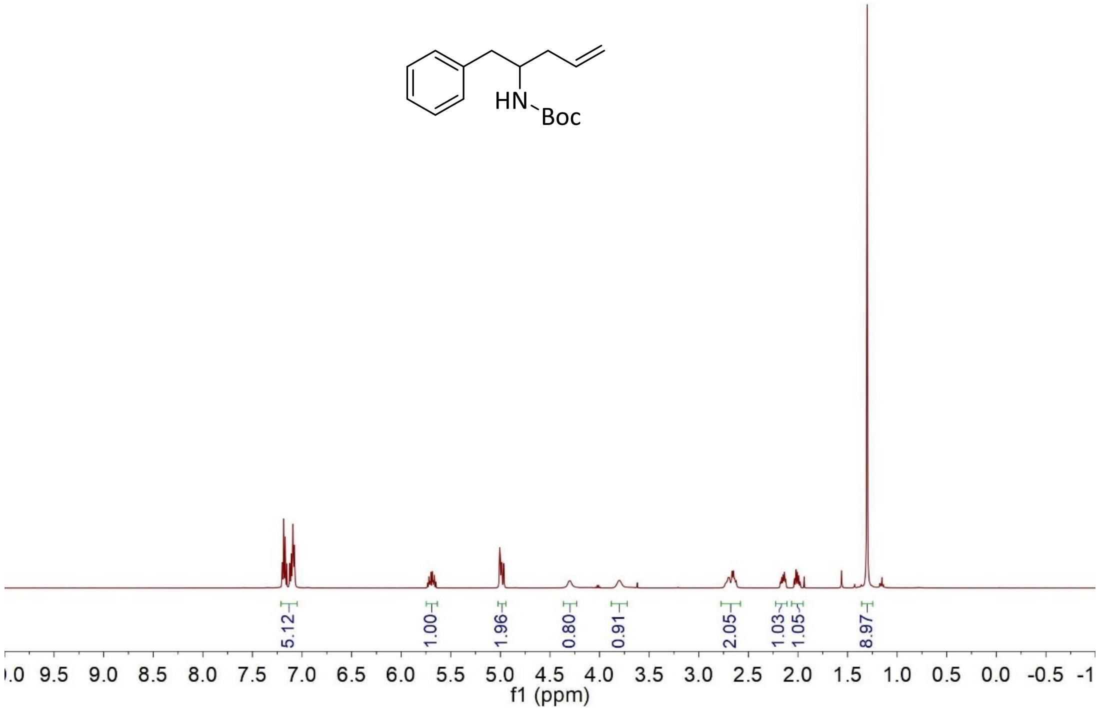
HN
Boc
5.12
1.00
1.96
0.80
0.91
2.05
1.03
1.05
8.97
f1 (ppm)

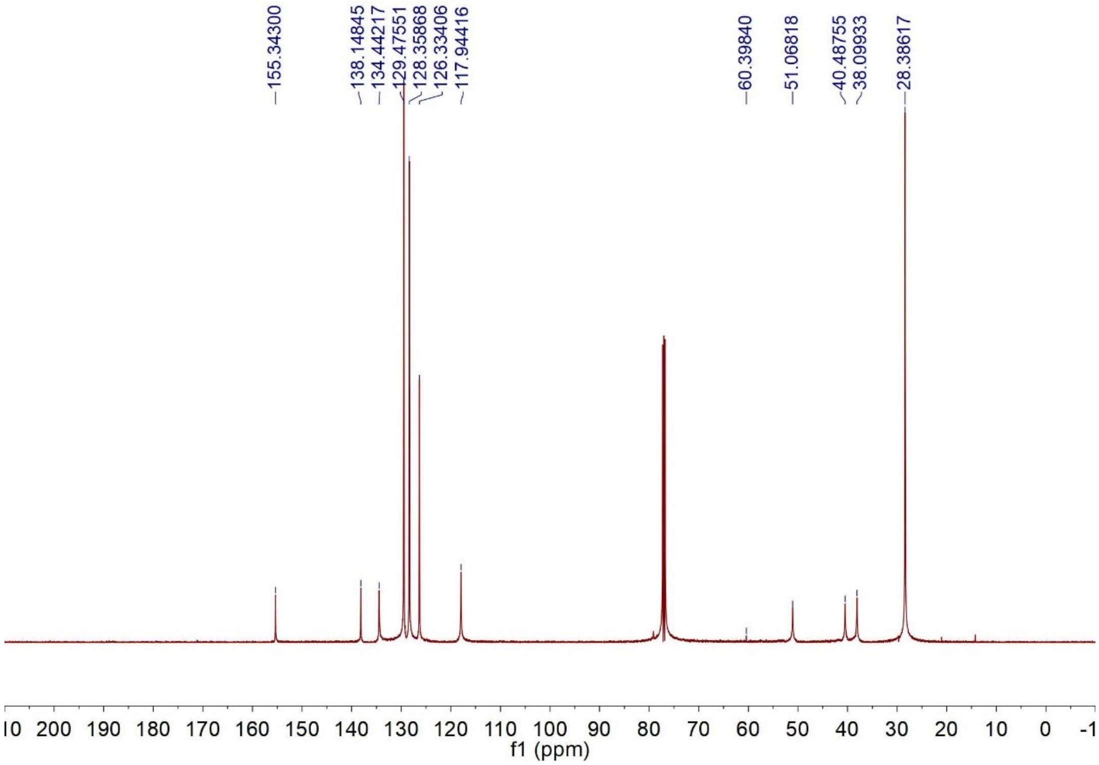
155.34300
138.14845
134.44217
129.47551
128.35868
126.33406
117.94416
60.39840
51.06818
40.48755
38.09933
28.38617
f1 (ppm)

(2c)

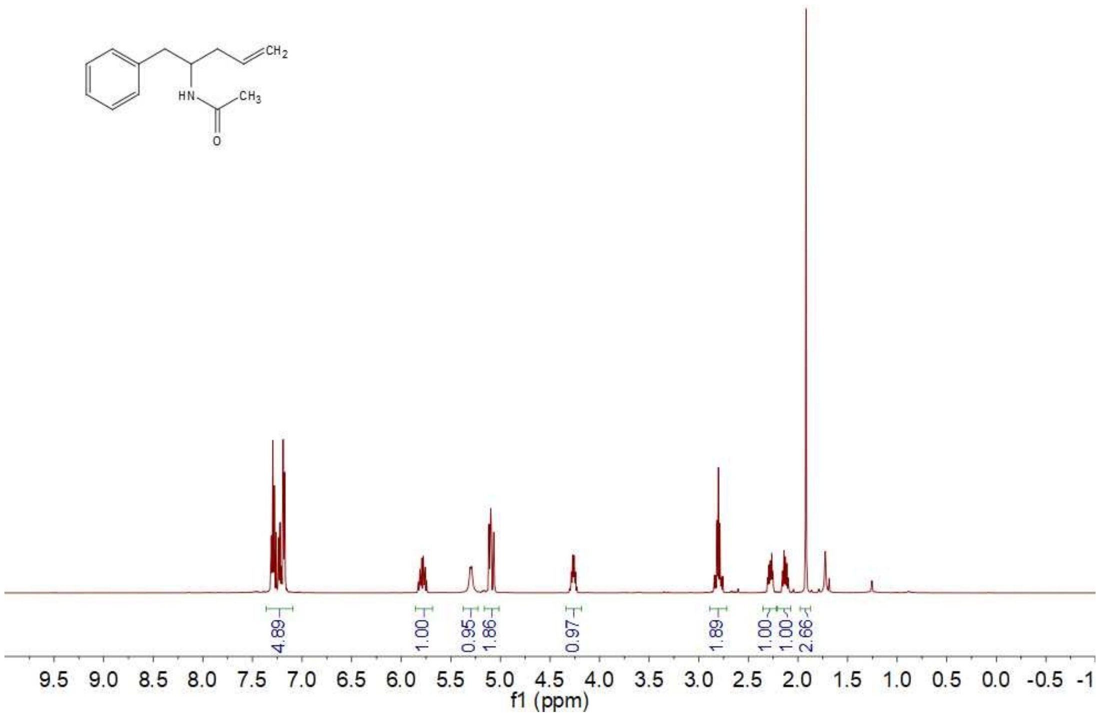
CH2
HN
CH3
O
4.89
1.00
0.95
1.86
0.97
1.89
1.00
1.00
2.66
9.5 9.0 8.5 8.0 7.5 7.0 6.5 6.0 5.5 5.0 4.5 4.0 3.5 3.0 2.5 2.0 1.5 1.0 0.5 0.0 -0.5 -1
f1 (ppm)

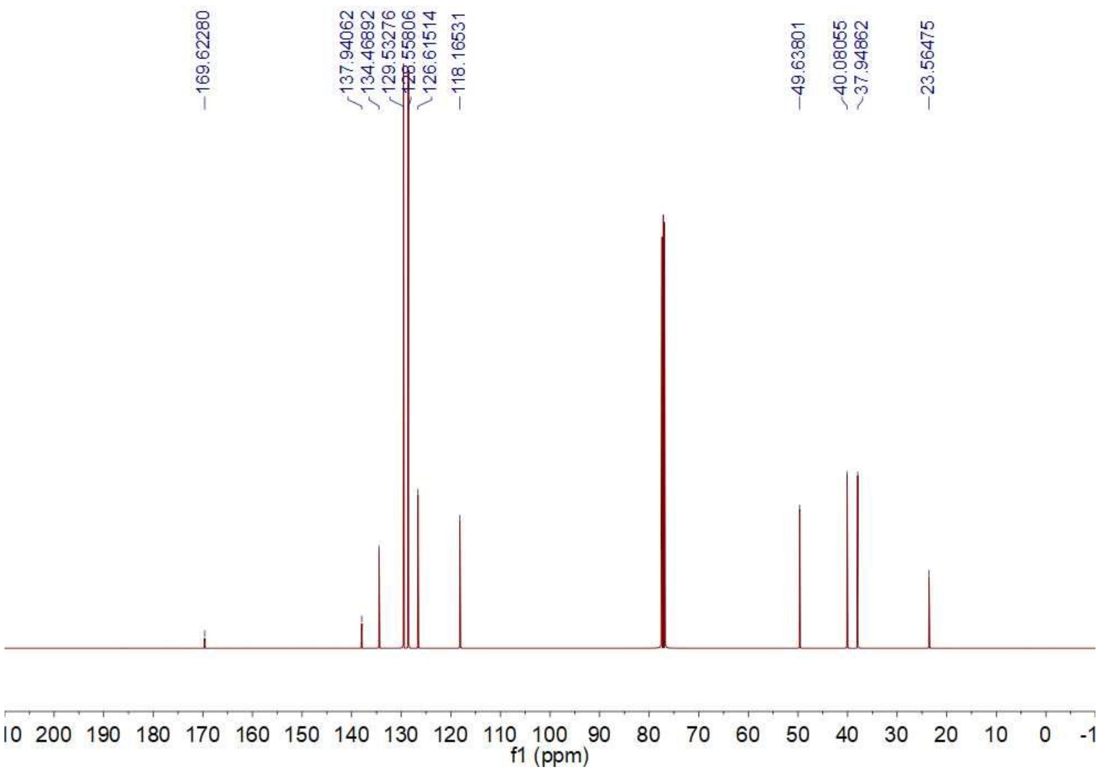
169.62280
137.94062
134.46892
129.53276
128.55806
126.61514
118.16531
49.63801
40.08055
37.94862
23.56475
10 200 190 180 170 160 150 140 130 120 110 100 90 80 70 60 50 40 30 20 10 0 -1
f1 (ppm)

(2d)

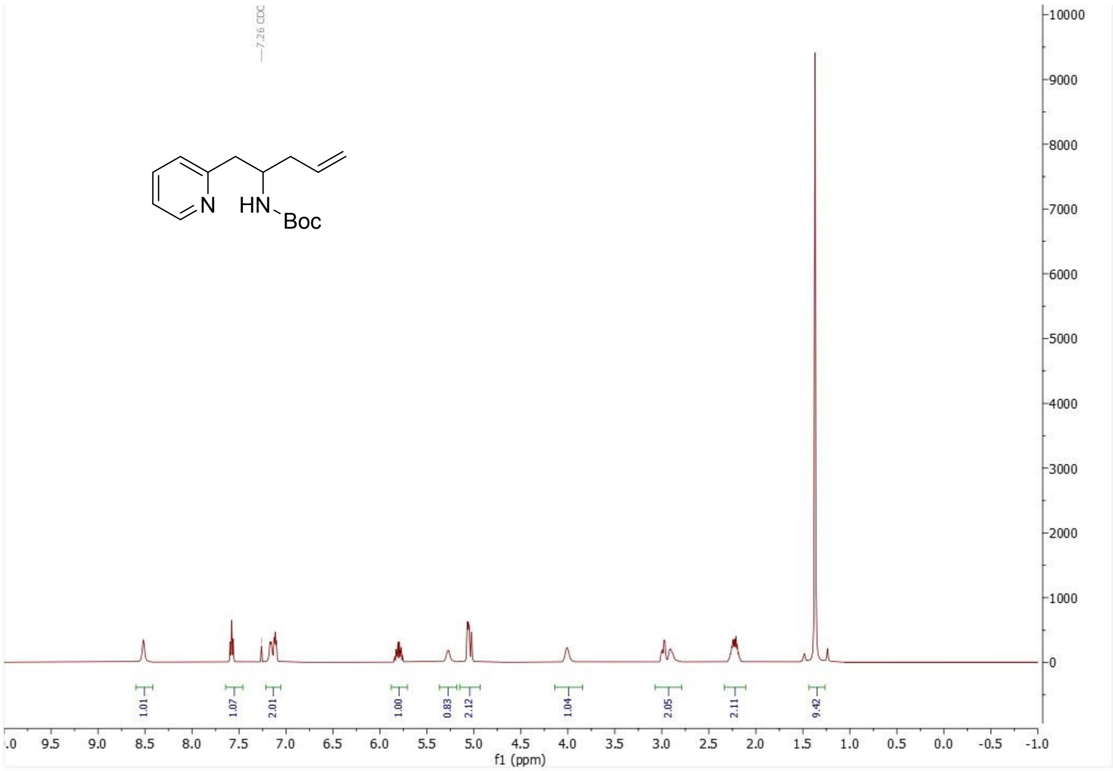

7.26 CDC
HN
Boc
N
1.01
1.07
2.01
1.00
0.83
2.12
1.04
2.05
2.11
9.42
f1 (ppm)

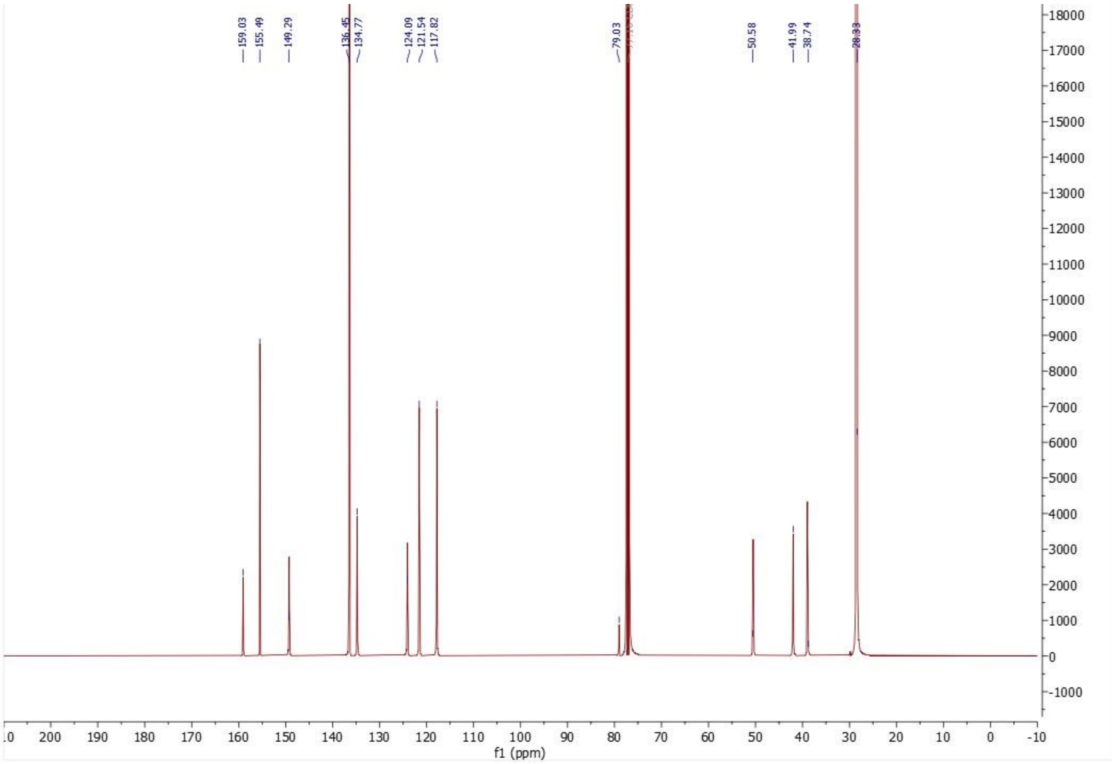

159.03
155.49
149.29
136.45
134.77
124.09
121.54
117.82
79.03
50.58
41.99
38.74
28.33
f1 (ppm)

(2e)

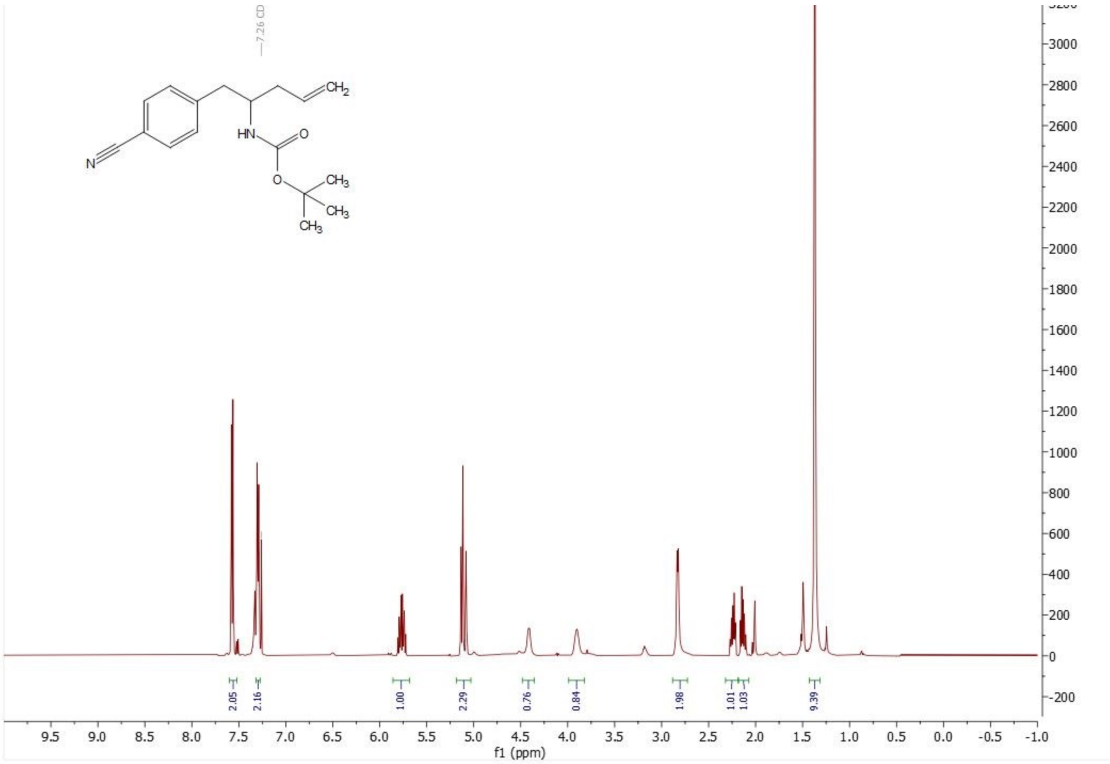

7.26 CD
CH2
HN
O
N
CH3
2.05
2.16
1.00
2.29
0.76
0.84
1.98
1.01
1.03
9.39
f1 (ppm)

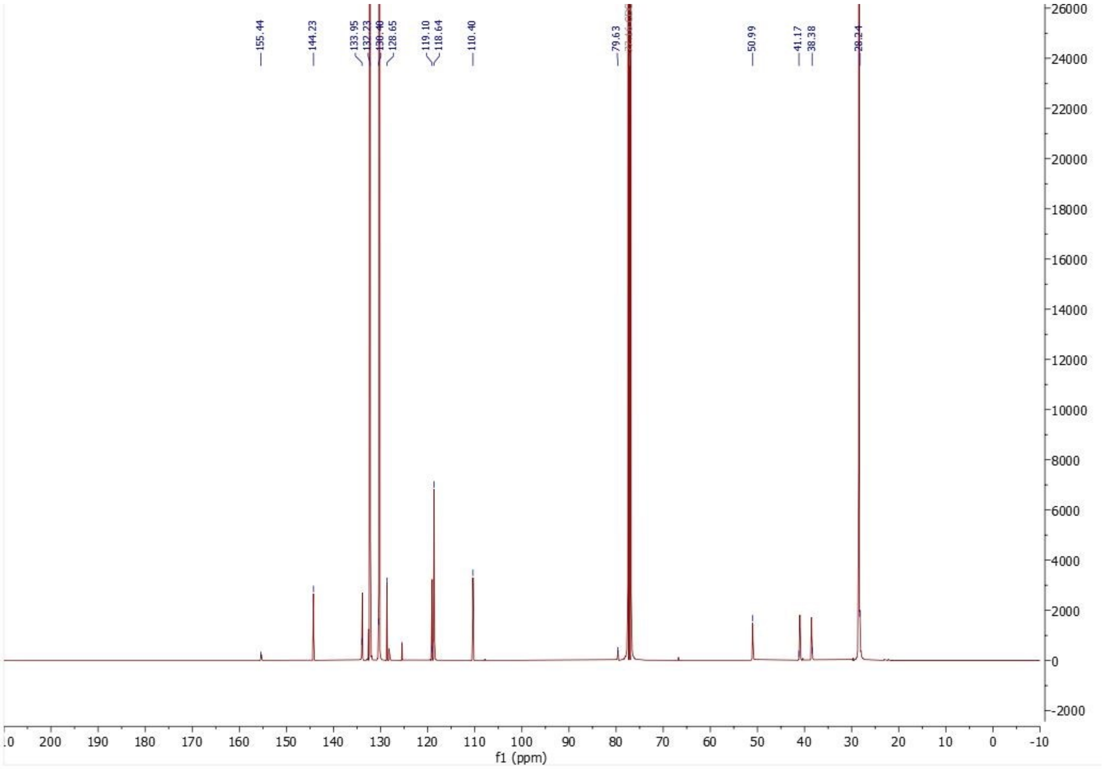

155.44
144.23
133.95
132.23
128.65
119.10
118.64
110.40
79.63
50.99
41.17
38.38
f1 (ppm)

(2f)

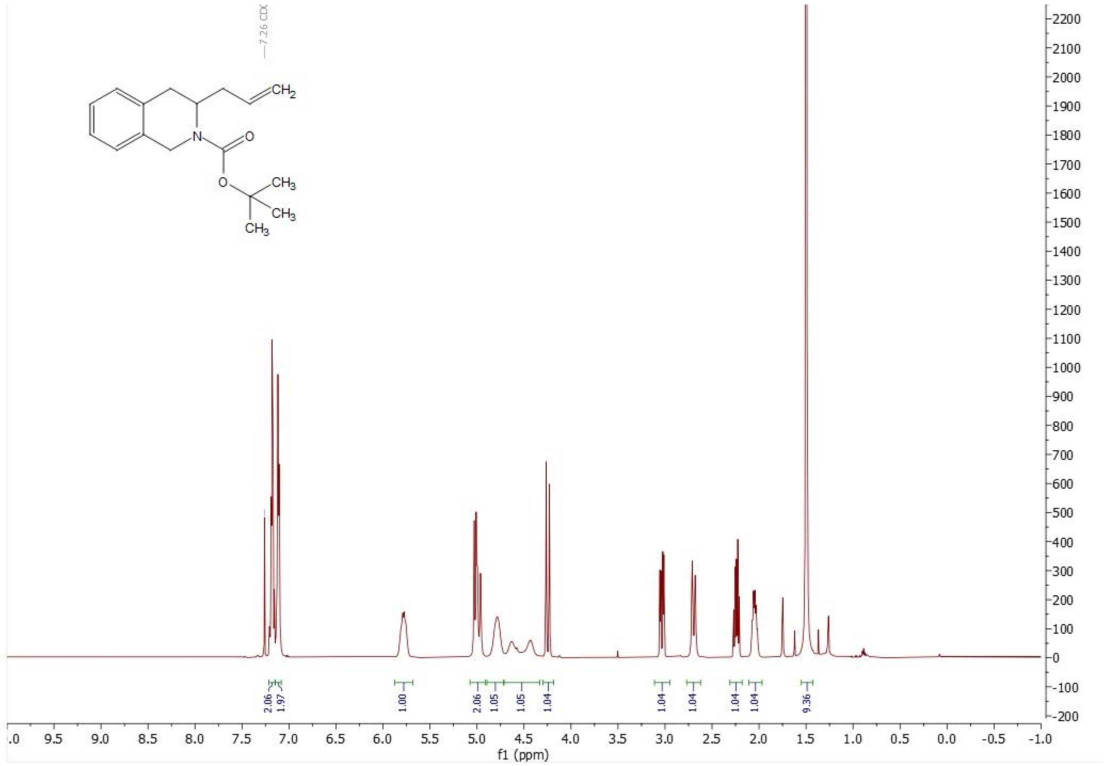
7.26 CDC
CH2
N
O
CH3
2.06
1.97
1.00
2.06
1.05
1.05
1.04
1.04
1.04
1.04
1.04
9.36
f1 (ppm)

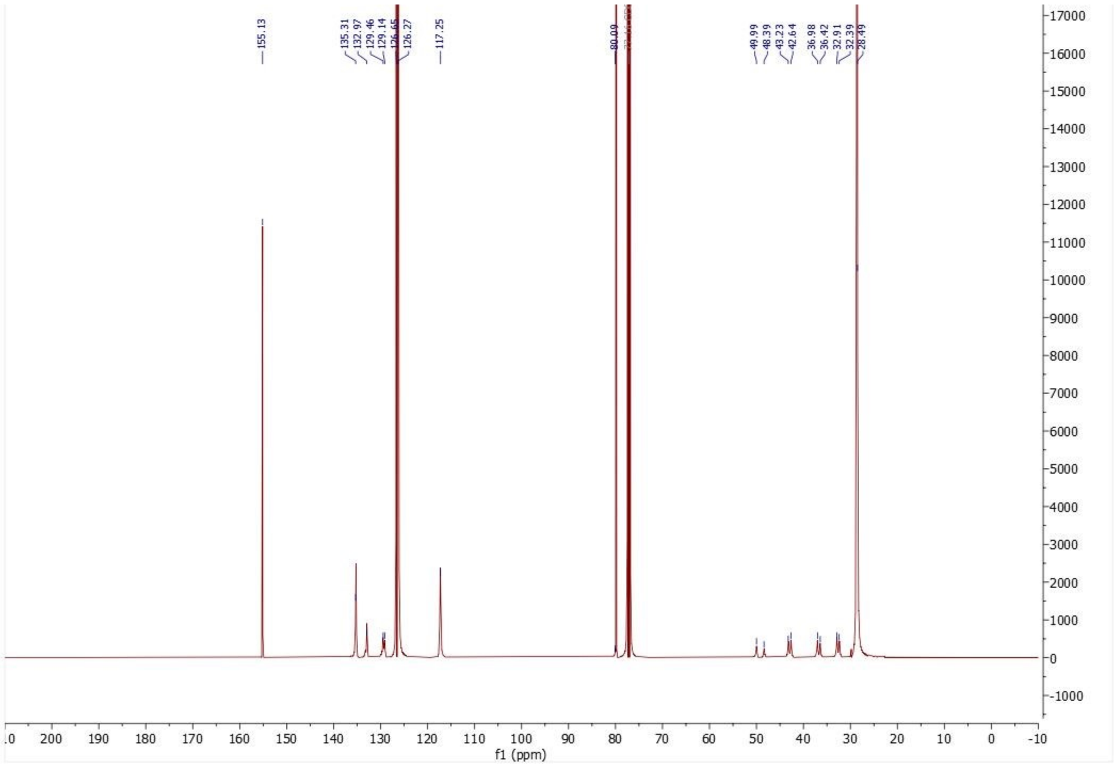
155.13
135.31
132.97
129.46
129.14
126.65
126.27
117.25
80.09
49.99
48.39
43.23
42.64
36.98
36.42
32.91
32.39
28.49
f1 (ppm)

(2g)

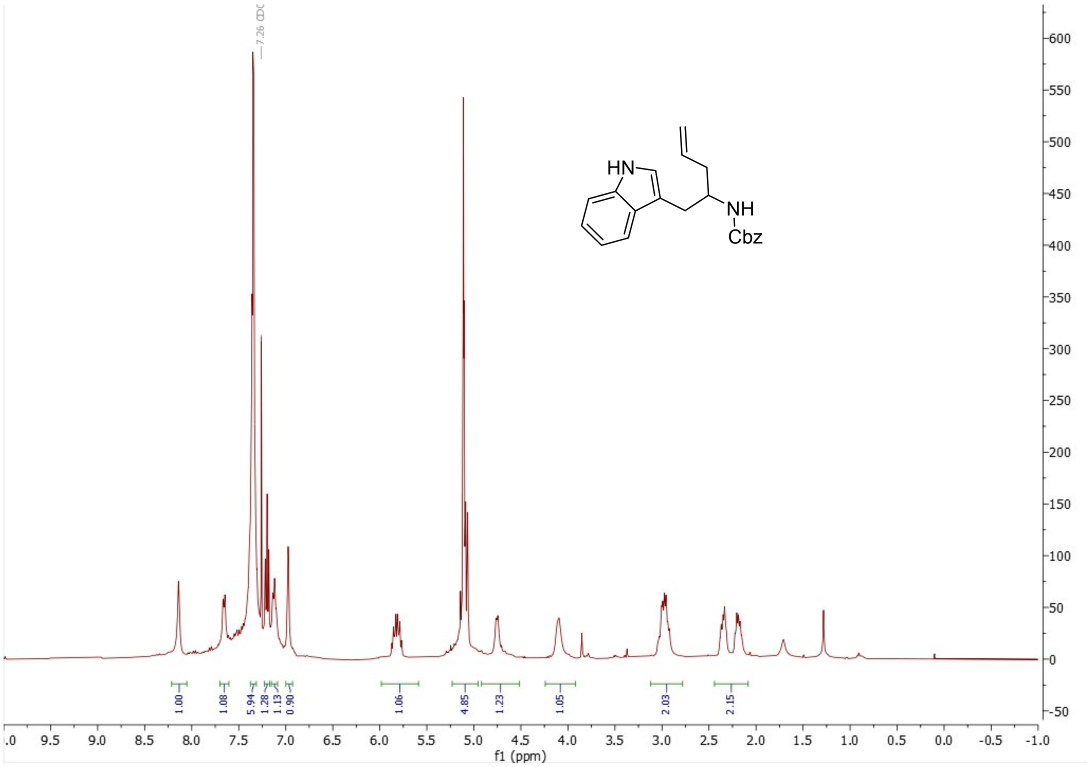
7.26 CDC
HN
NH
Cbz
1.00
1.08
5.94
1.28
1.13
0.90
1.06
4.85
1.23
1.05
2.03
2.15
f1 (ppm)

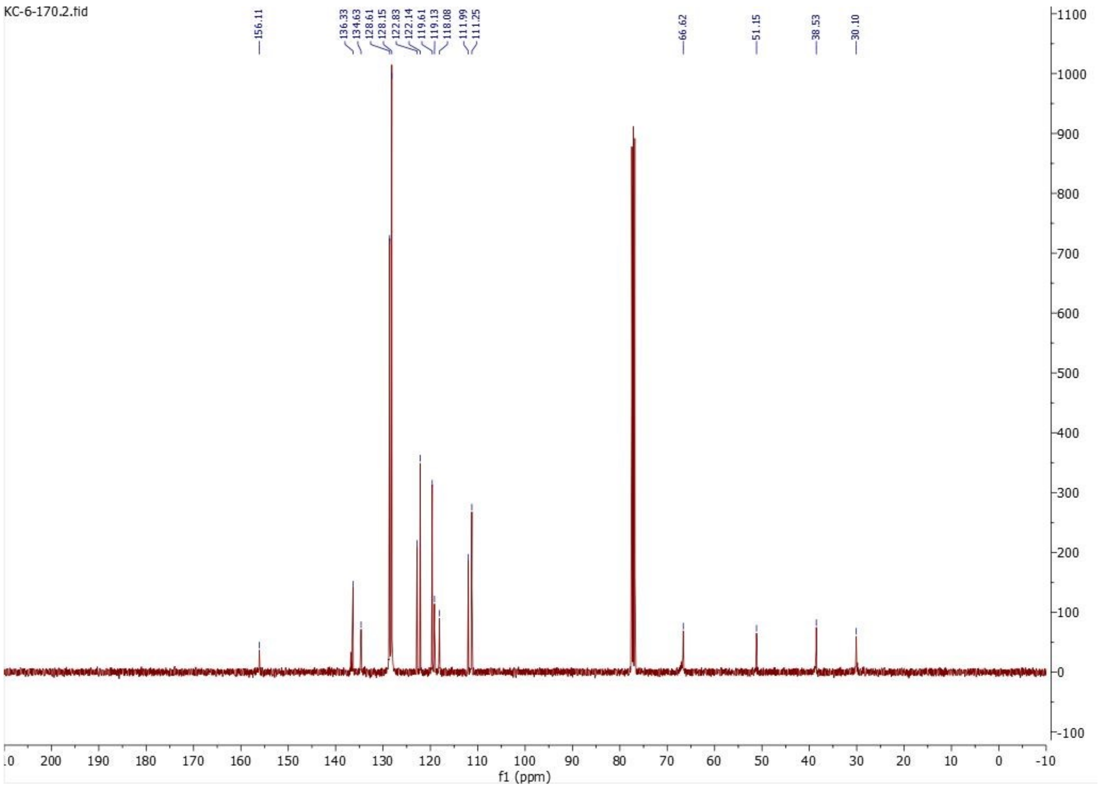
KC-6-170.2.fid
156.11
136.33
134.63
128.61
128.15
122.83
122.14
119.61
119.13
118.08
111.99
111.25
66.62
51.15
38.53
30.10
f1 (ppm)

(2h)

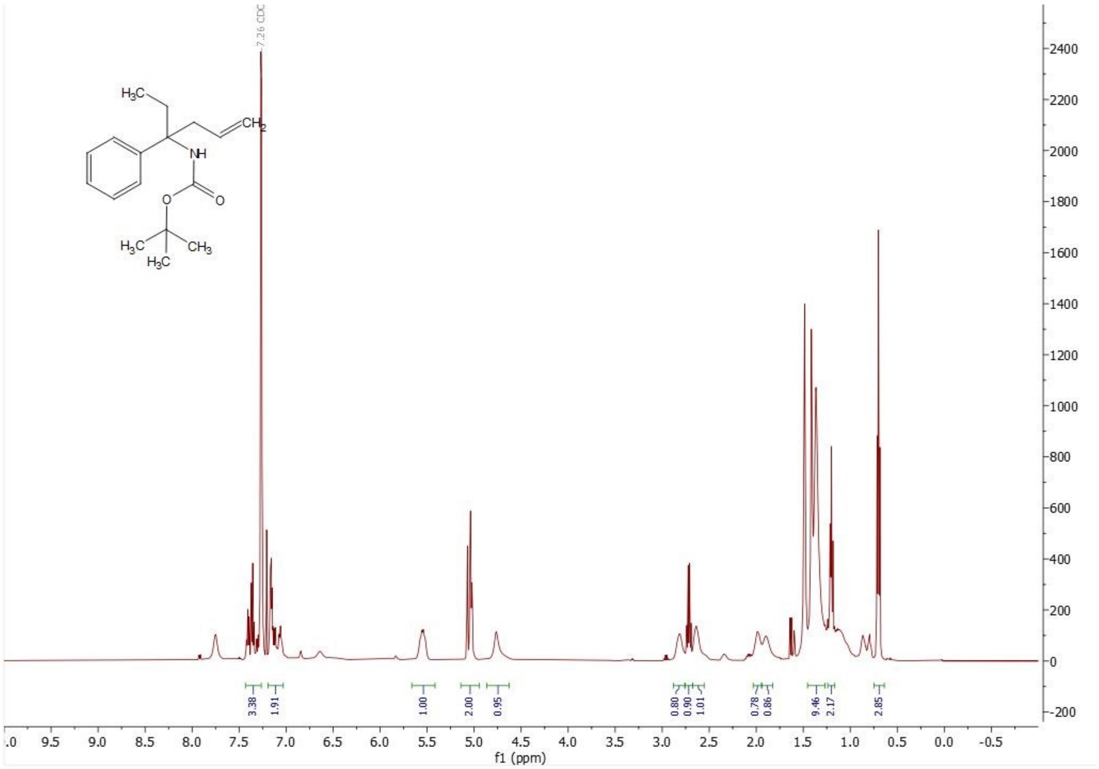

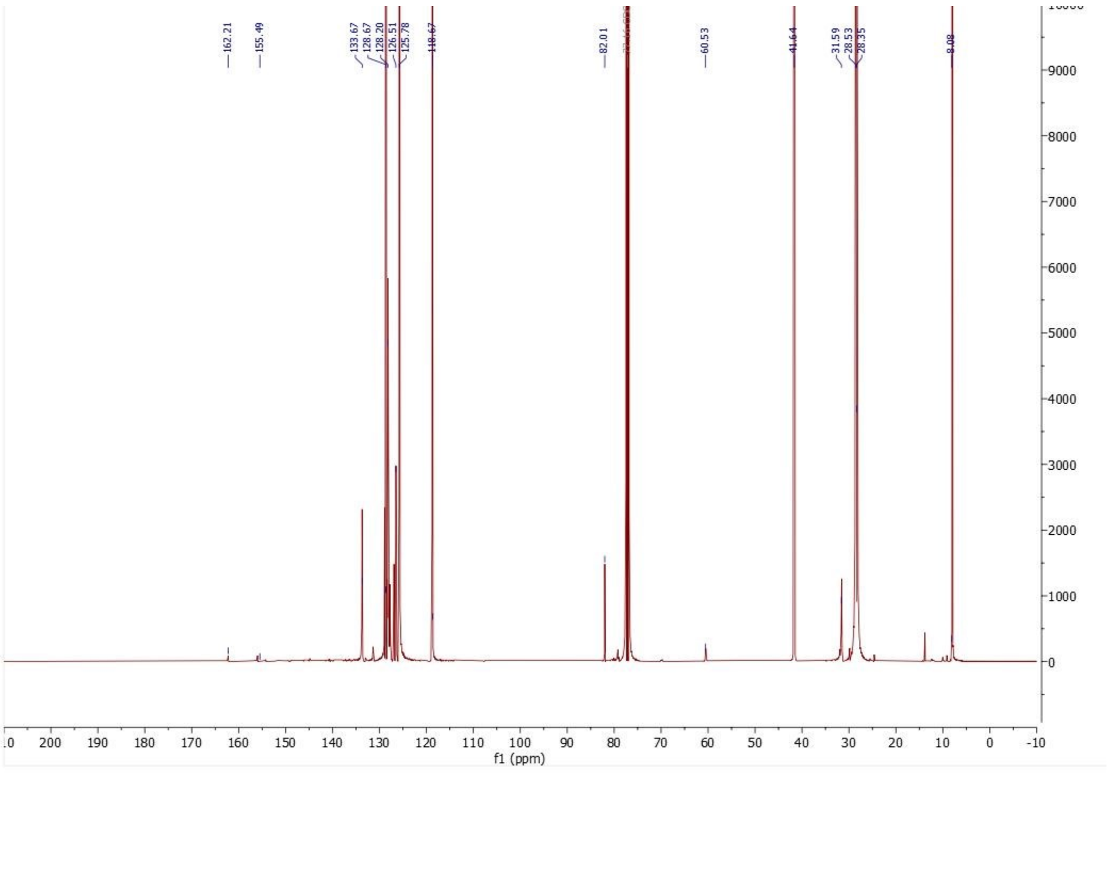

(2i)

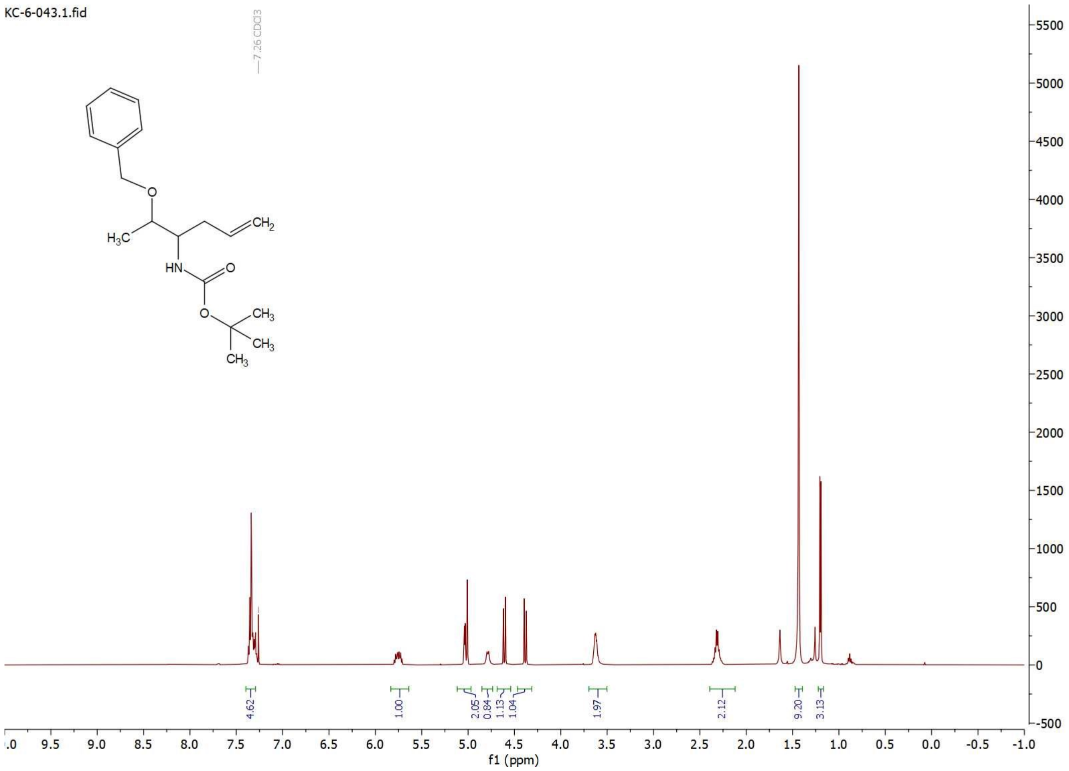

KC-6-043.1.fid
7.26 CDCl3
H3C
O
HN
O
O
CH2
CH3
CH3
CH3
4.62
1.00
2.05
0.84
1.13
1.04
1.97
2.12
9.20
3.13
f1 (ppm)

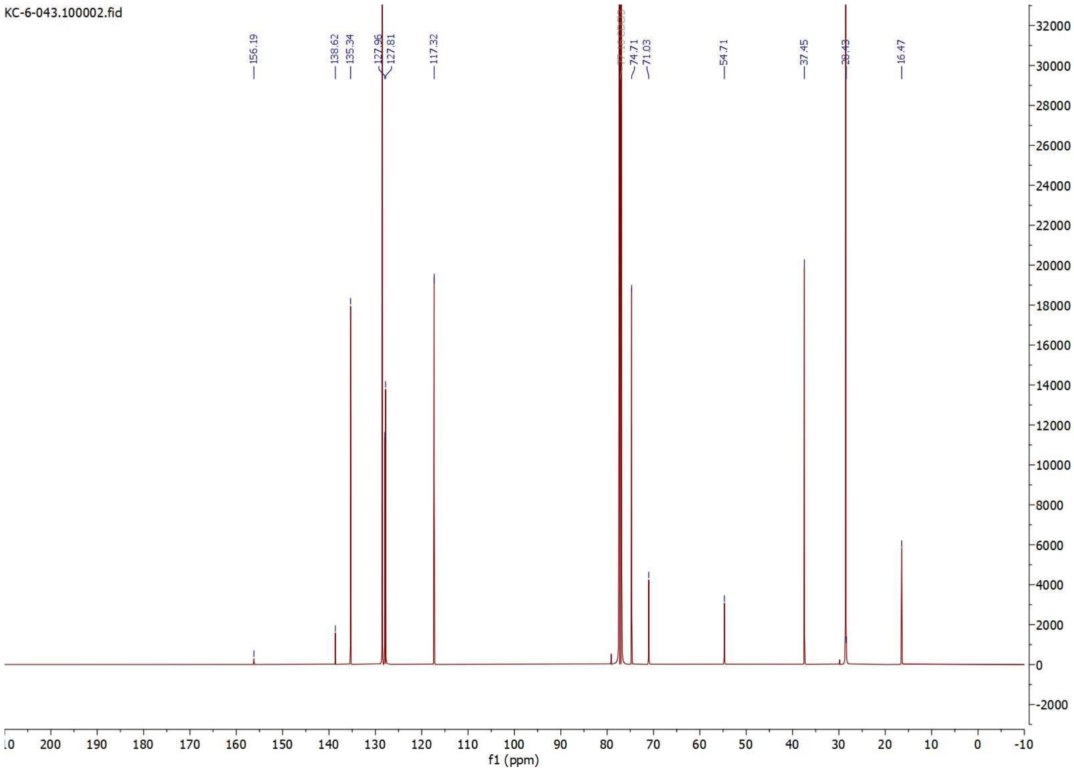

KC-6-043.100002.fid
156.19
138.62
135.34
127.96
127.81
117.32
74.71
71.03
54.71
37.45
28.43
16.47
f1 (ppm)

(2j)

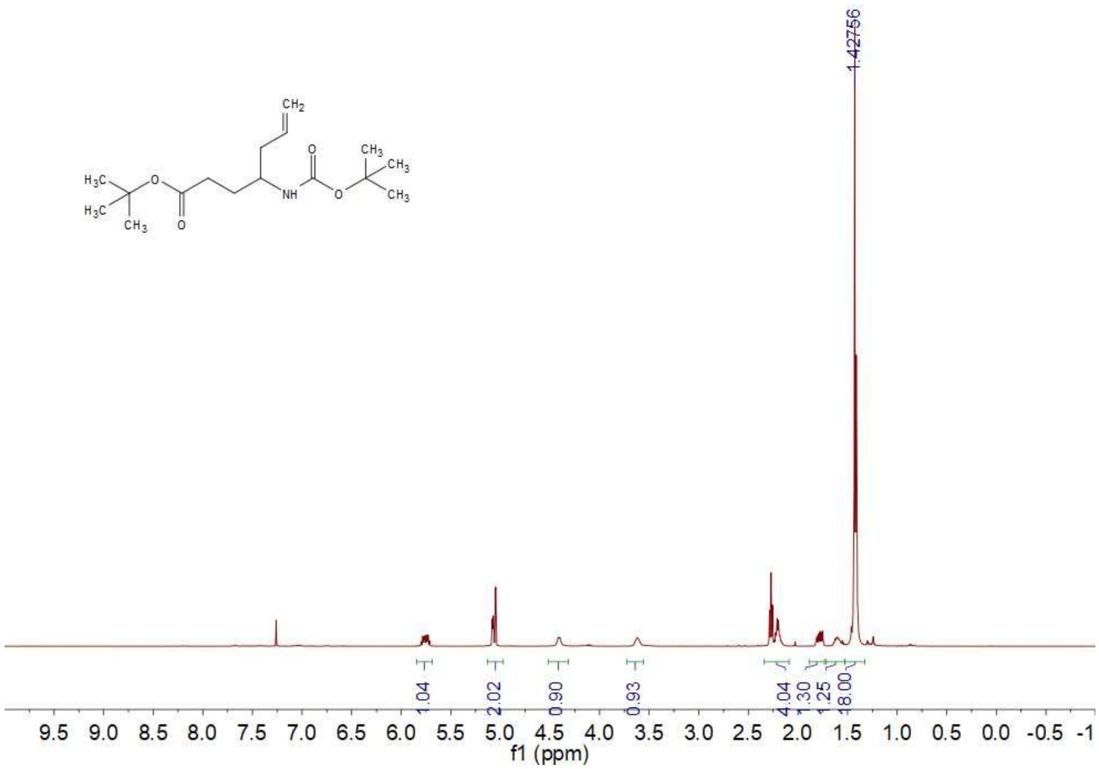
CH2
CH3
CH3
CH3
H3C
H3C
CH3
O
O
O
O
NH
1.42756
1.04
2.02
0.90
0.93
4.04
1.30
1.25
18.00
9.5 9.0 8.5 8.0 7.5 7.0 6.5 6.0 5.5 5.0 4.5 4.0 3.5 3.0 2.5 2.0 1.5 1.0 0.5 0.0 -0.5 -1
f1 (ppm)

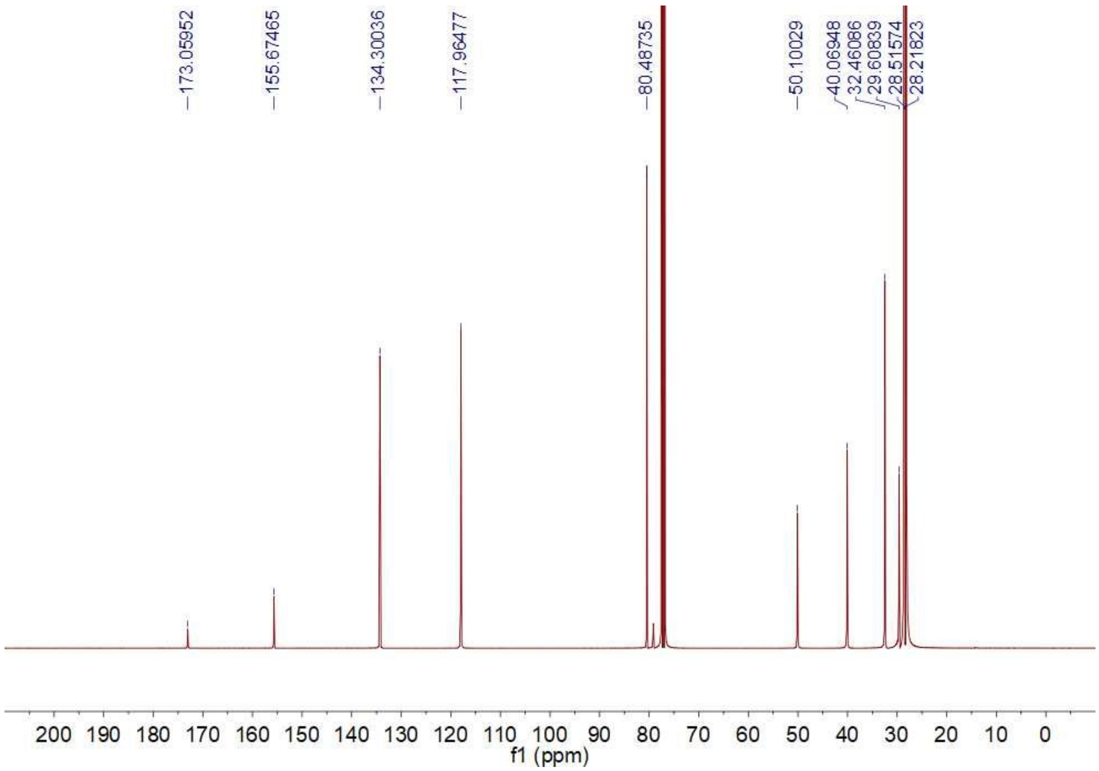
173.05952
155.67465
134.30036
117.96477
80.48735
50.10029
40.06948
32.46086
29.60839
28.51574
28.21823
200 190 180 170 160 150 140 130 120 110 100 90 80 70 60 50 40 30 20 10 0
f1 (ppm)

(2k)

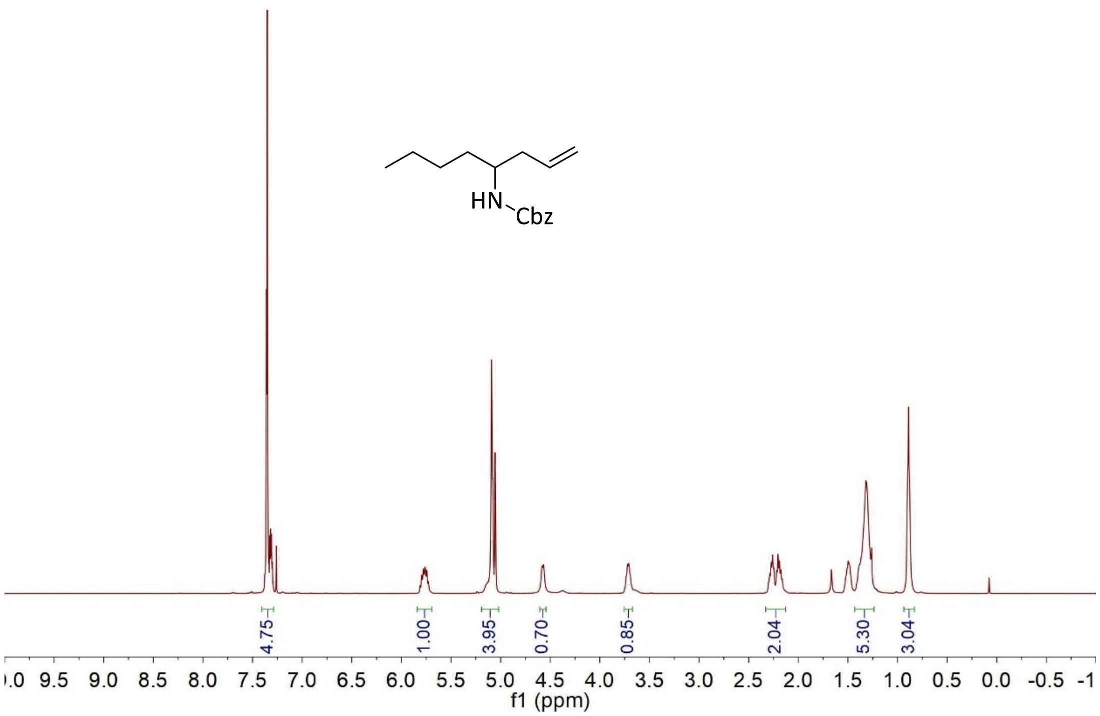
HN
Cbz
4.75
1.00
3.95
0.70
0.85
2.04
5.30
3.04
f1 (ppm)

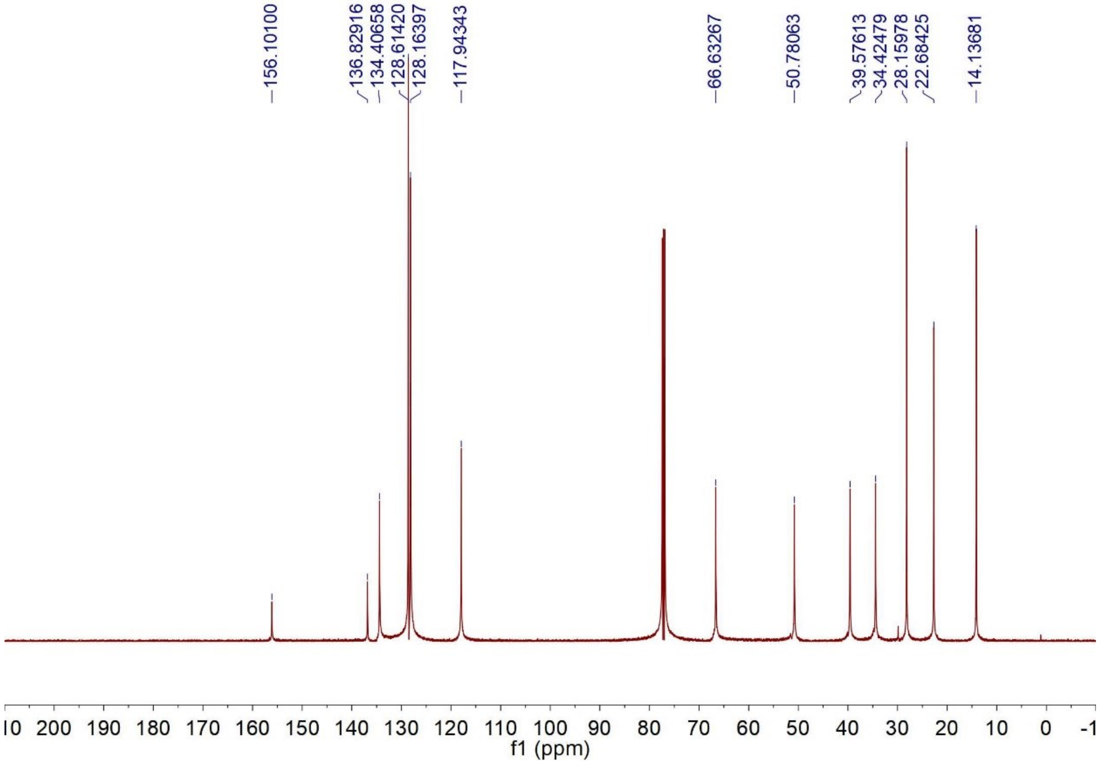
156.10100
136.82916
134.40658
128.61420
128.16397
117.94343
66.63267
50.78063
39.57613
34.42479
28.15978
22.68425
14.13681
f1 (ppm)

(2l)

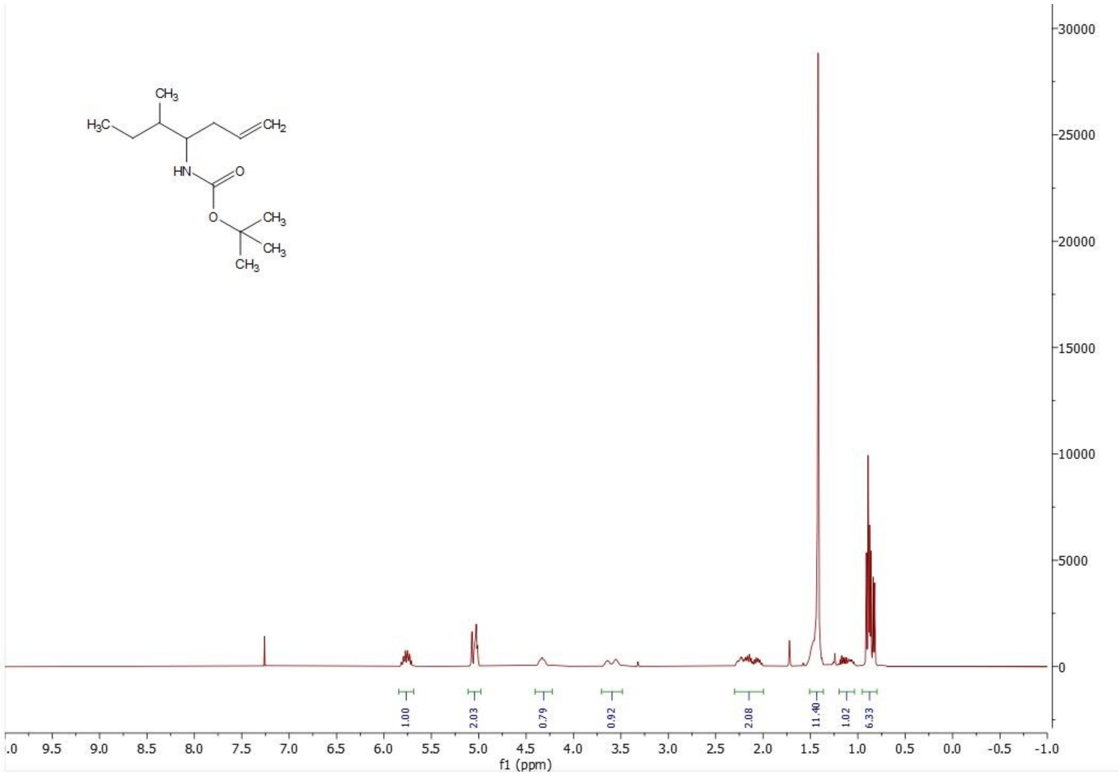

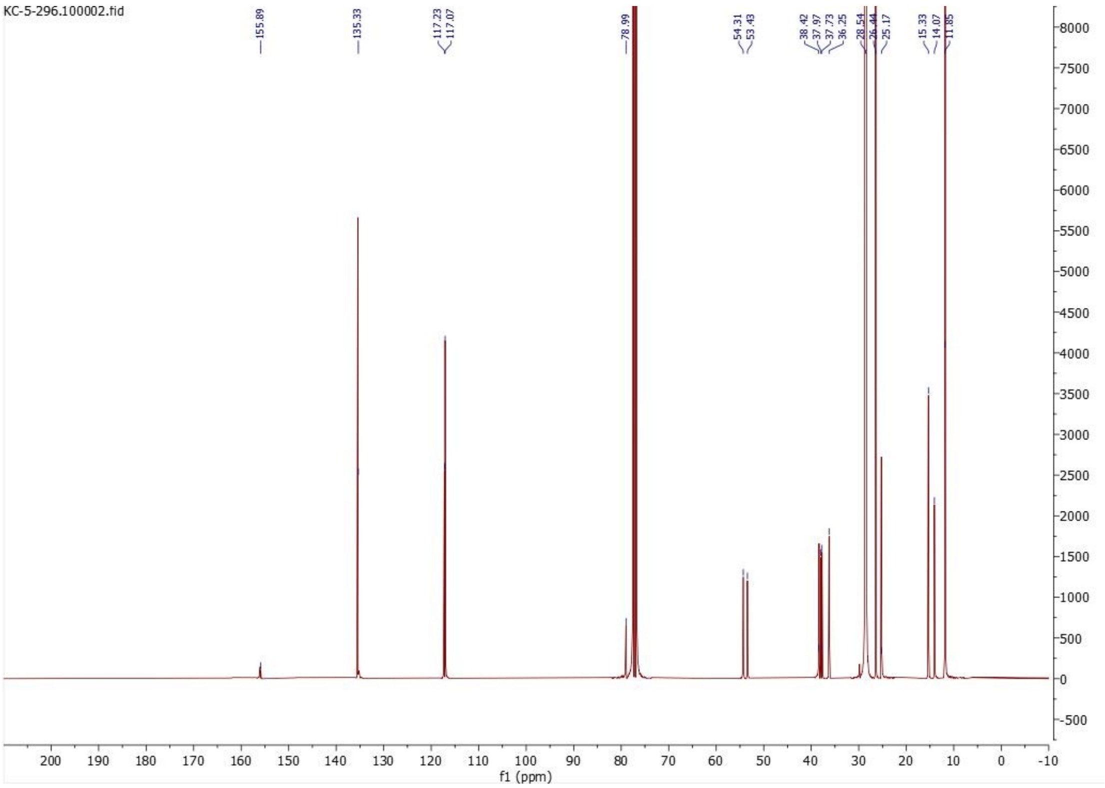

(2m)

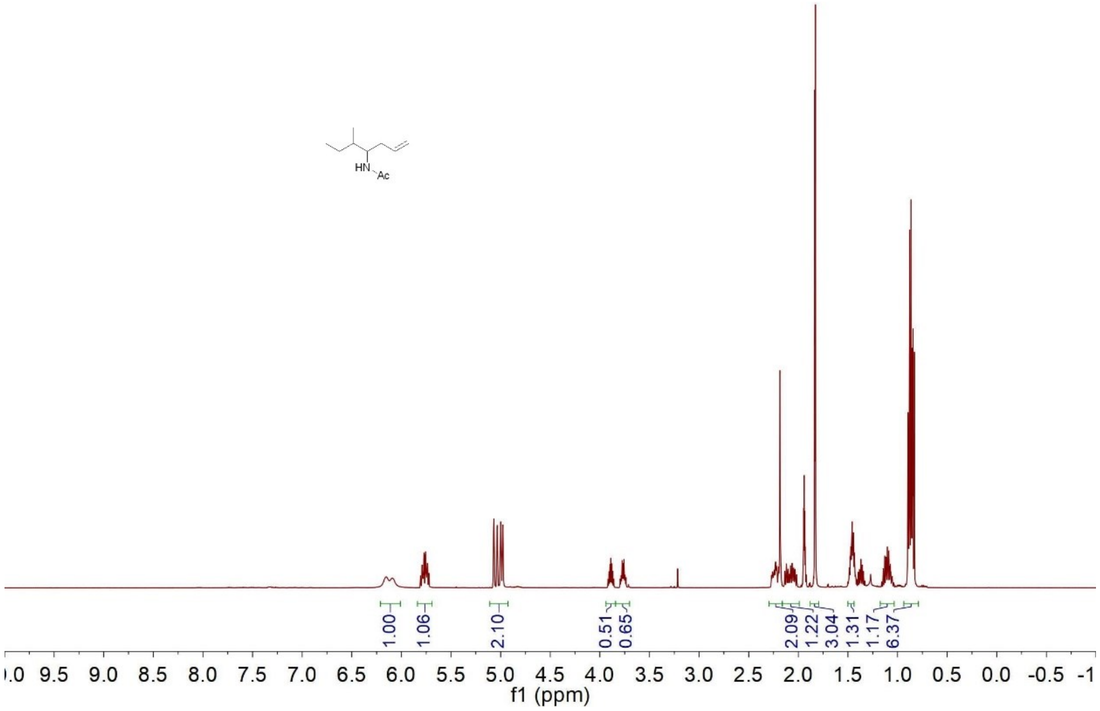
HN
Ac
1.00
1.06
2.10
0.51
0.65
2.09
1.22
3.04
1.31
1.17
6.37
f1 (ppm)

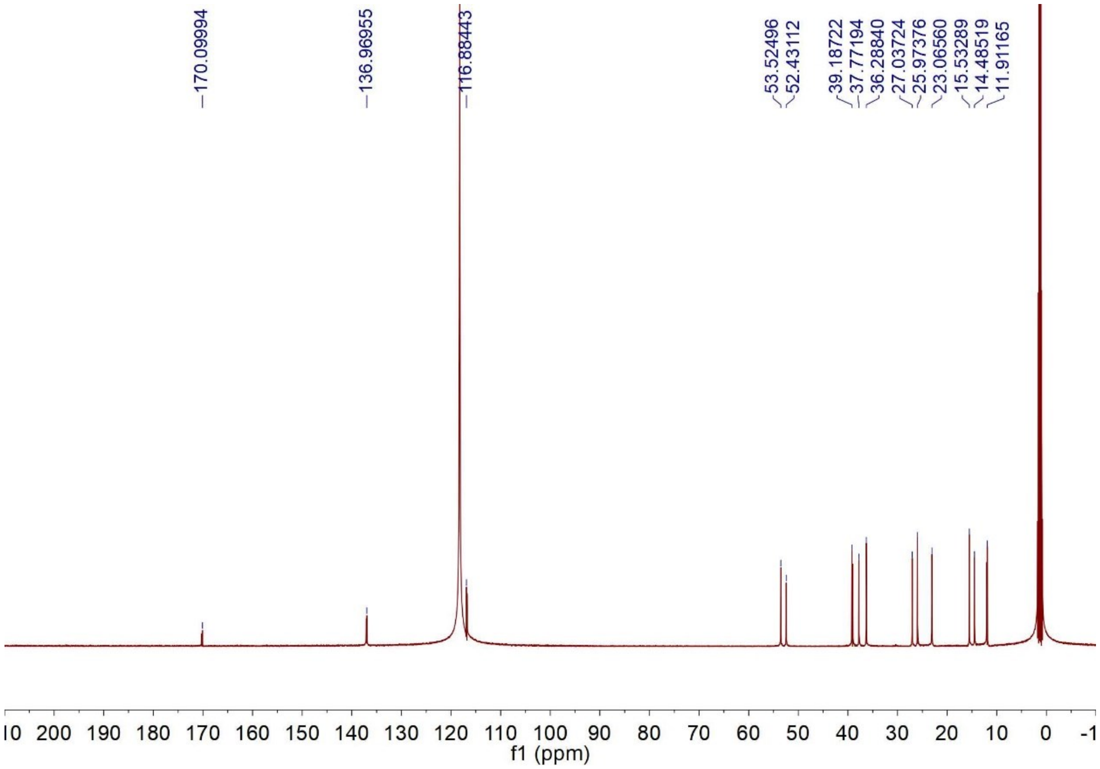
170.09994
136.96955
116.88443
53.52496
52.43112
39.18722
37.77194
36.28840
27.03724
25.97376
23.06560
15.53289
14.48519
11.91165
f1 (ppm)

(2n)

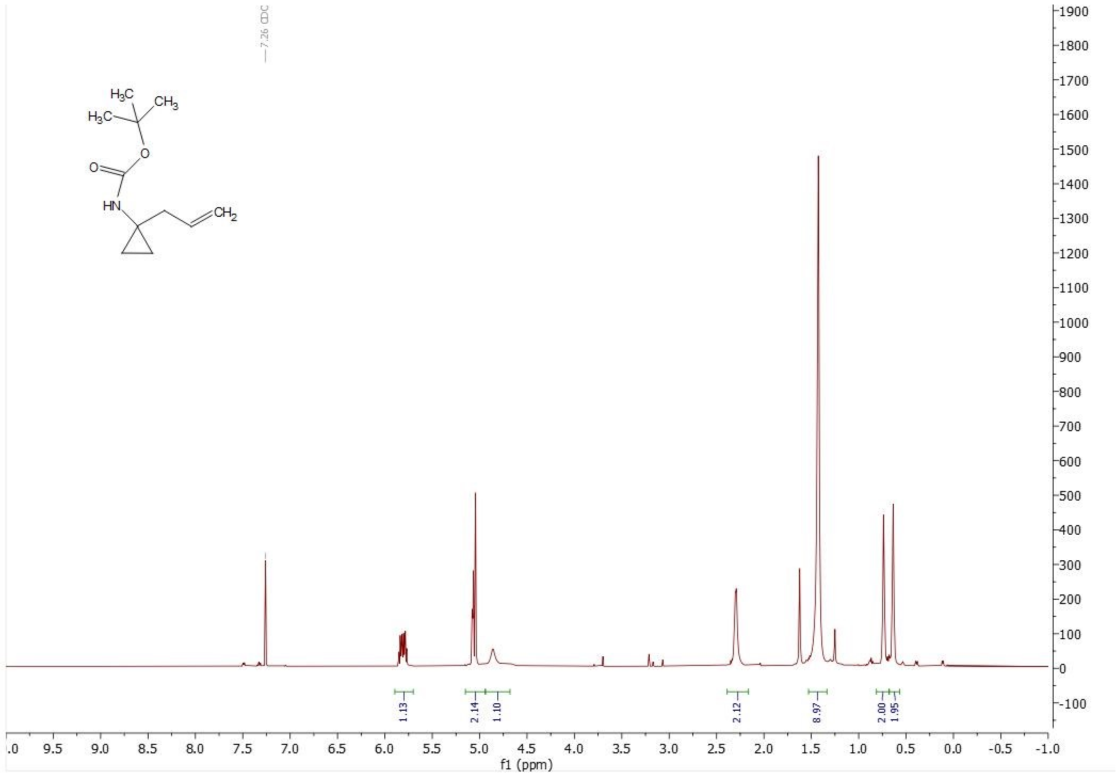

7.26 CDC
H3C
CH3
H3C
O
O
HN
CH2
1.13
2.14
1.10
2.12
8.97
2.00
1.95
f1 (ppm)

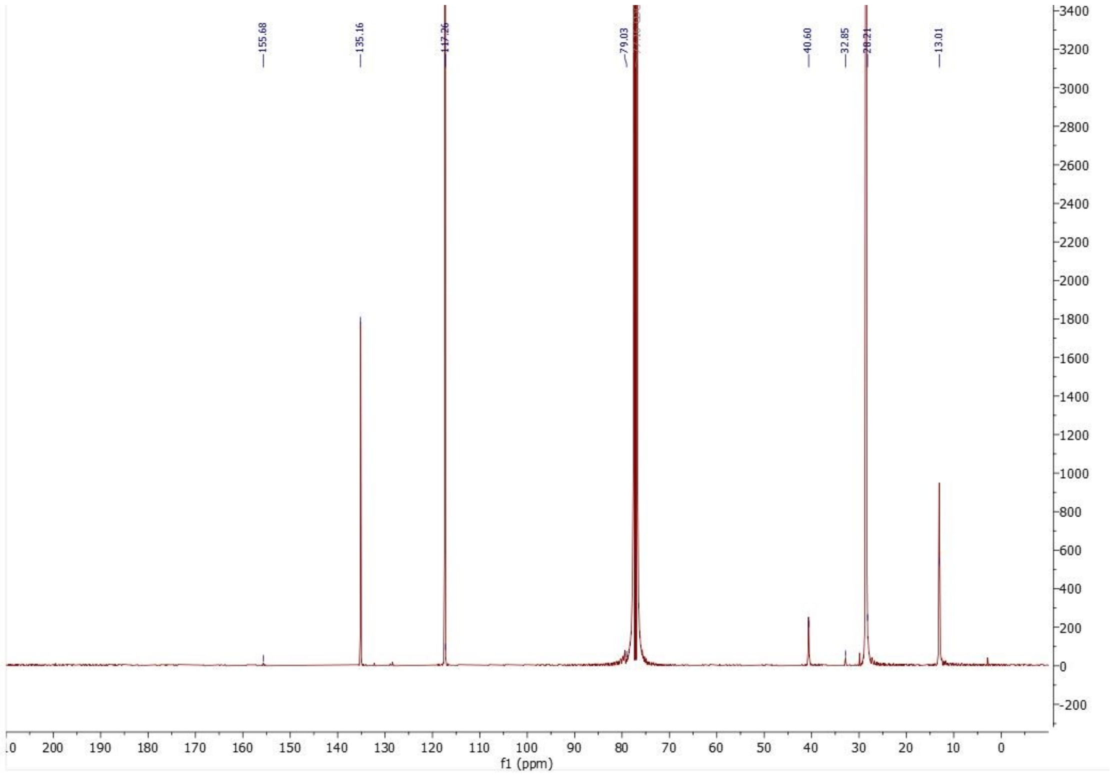

155.68
135.16
117.26
79.03
40.60
32.85
28.21
13.01
f1 (ppm)

(2o)

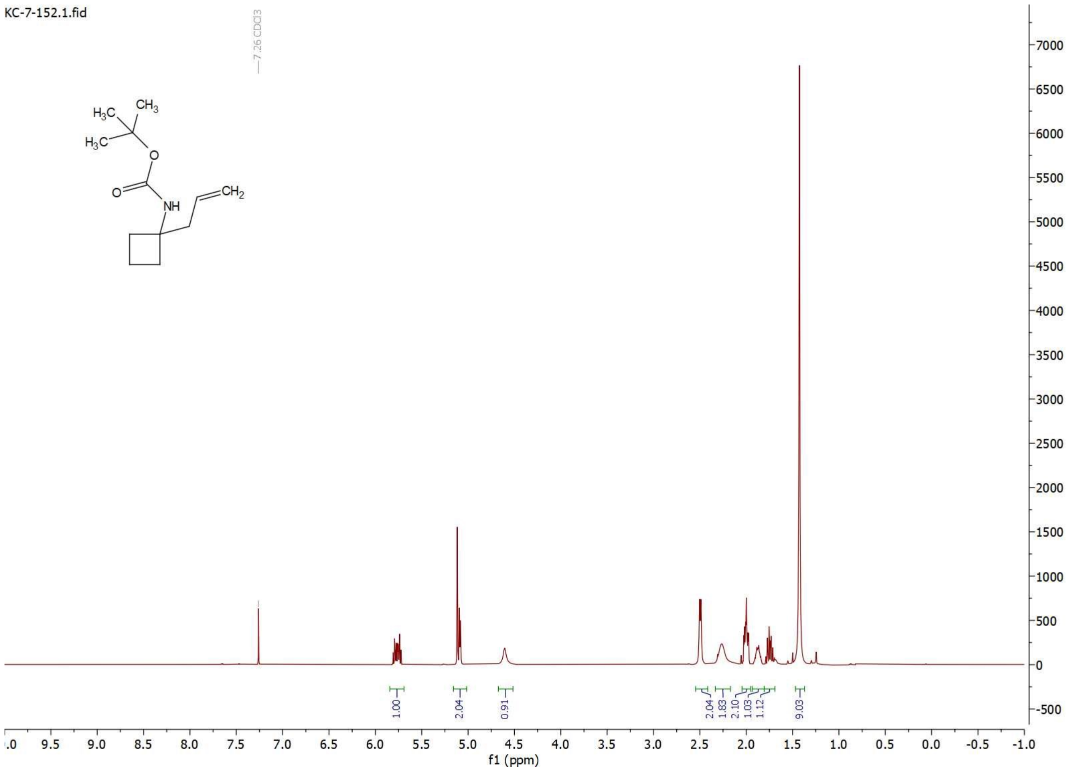
KC-7-152.1.fid
7.26 CDCl3
H3C
CH3
H3C
O
O
NH
CH2
1.00
2.04
0.91
2.04
1.83
2.10
1.03
1.12
9.03
f1 (ppm)

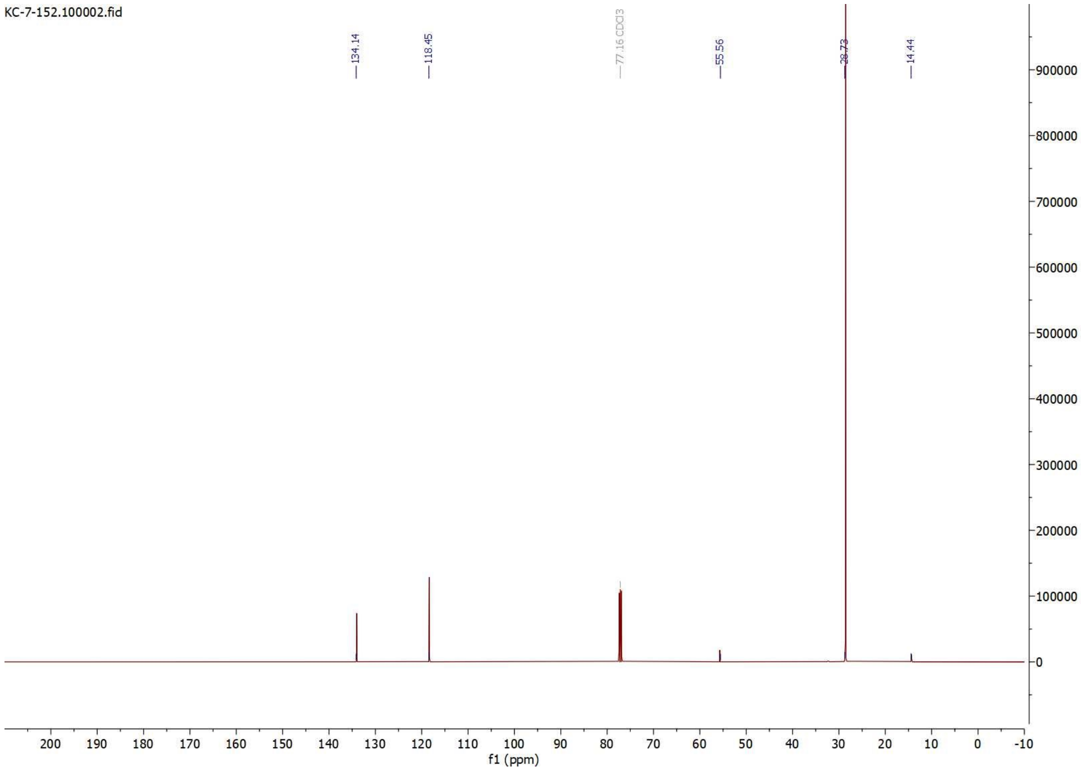
KC-7-152.100002.fid
134.14
118.45
77.16 CDCl3
55.56
28.73
14.44
f1 (ppm)

(2p)

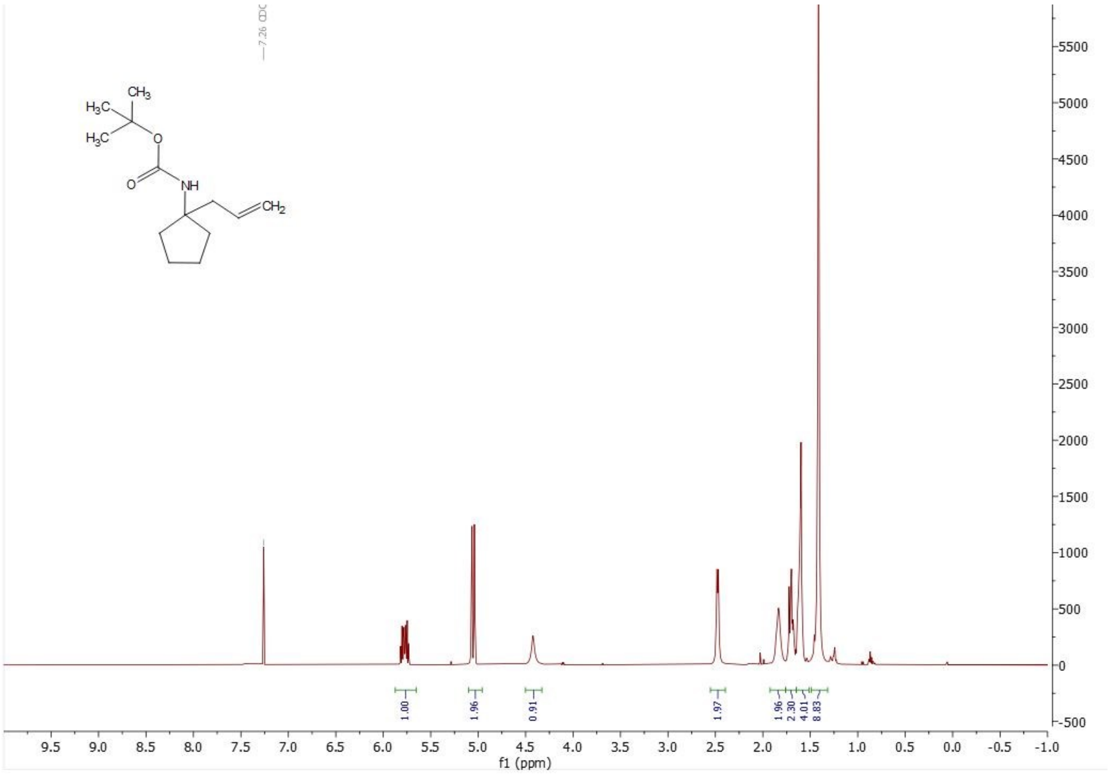

7.26 CDC
H_3C
CH_3
H_3C
O
O
NH
CH_2
1.00
1.96
0.91
1.97
1.96
2.30
4.01
8.83
f1 (ppm)

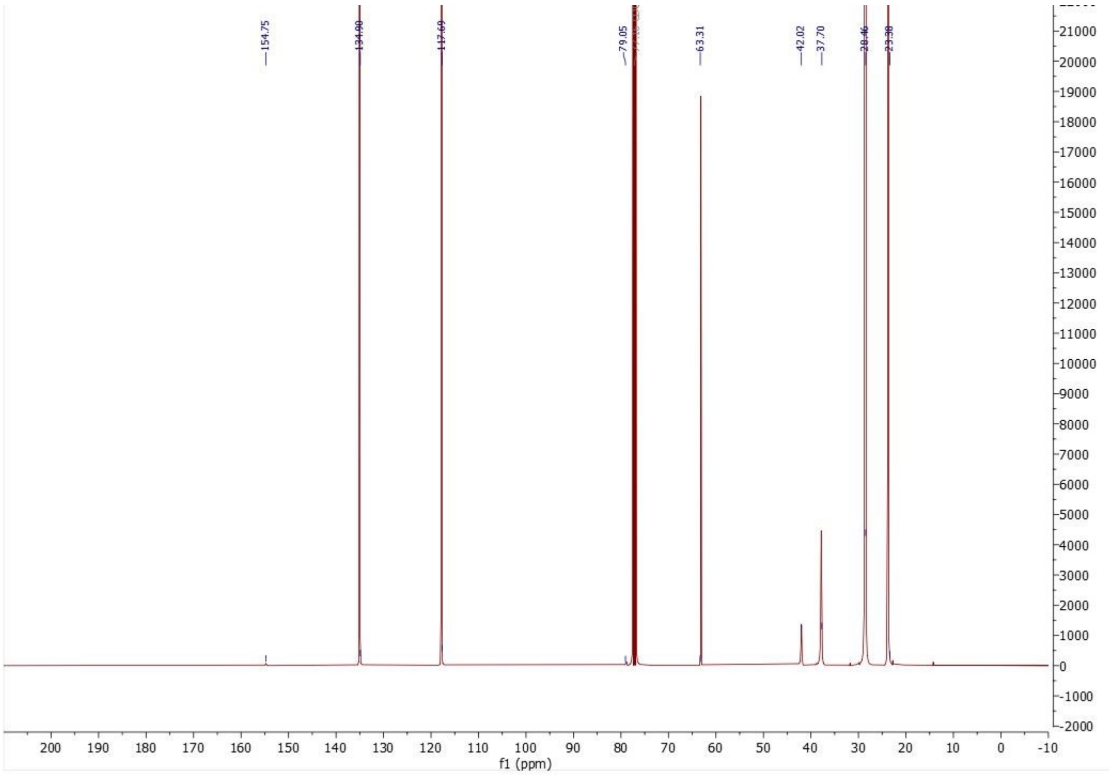

154.75
134.90
117.69
79.05
63.31
42.02
37.70
28.46
23.38
f1 (ppm)

(2q)

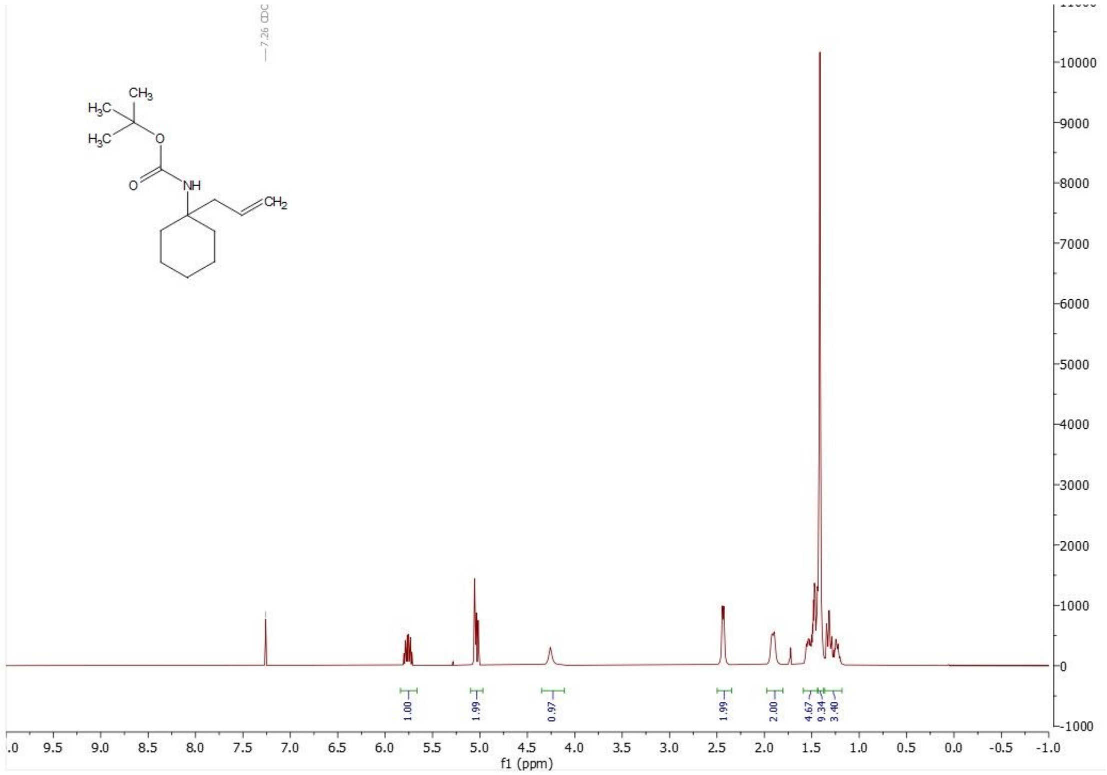

7.26 CDC
CH3
H3C
H3C
O
O
NH
CH2
1.00
1.99
0.97
1.99
2.00
4.67
9.34
3.40
f1 (ppm)

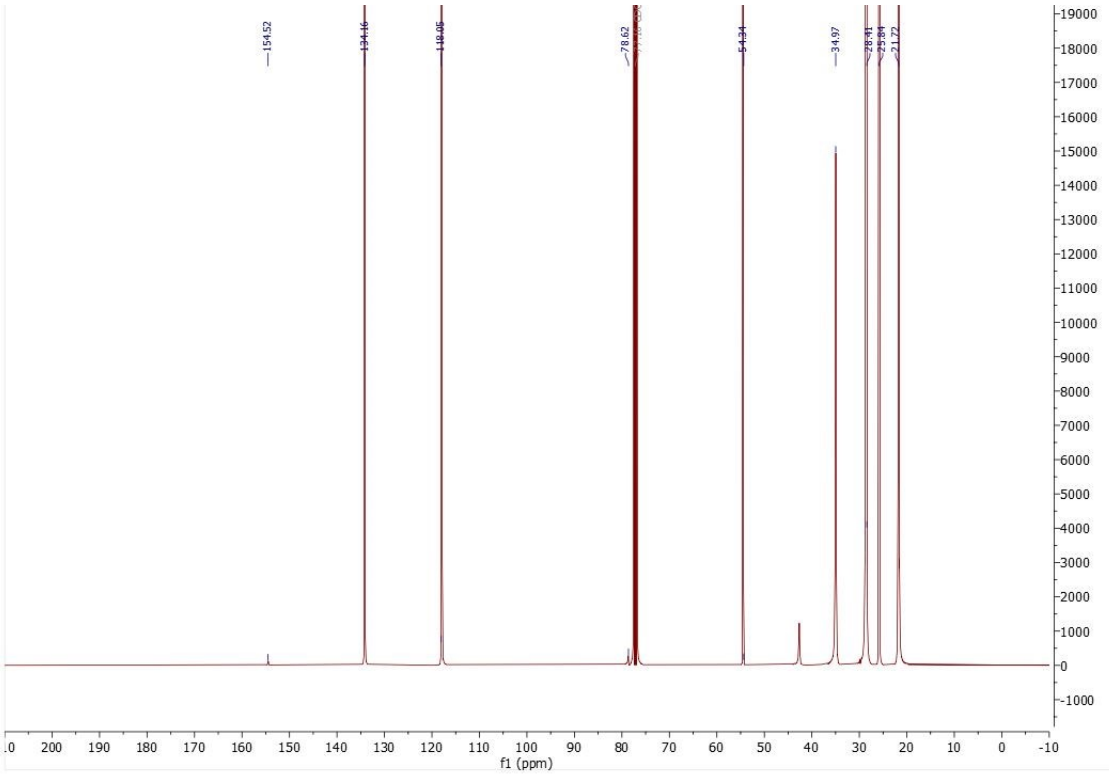

154.52
134.16
118.05
78.62
54.34
34.97
28.41
25.84
21.72
f1 (ppm)

(2r)

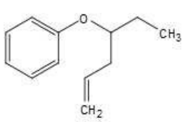

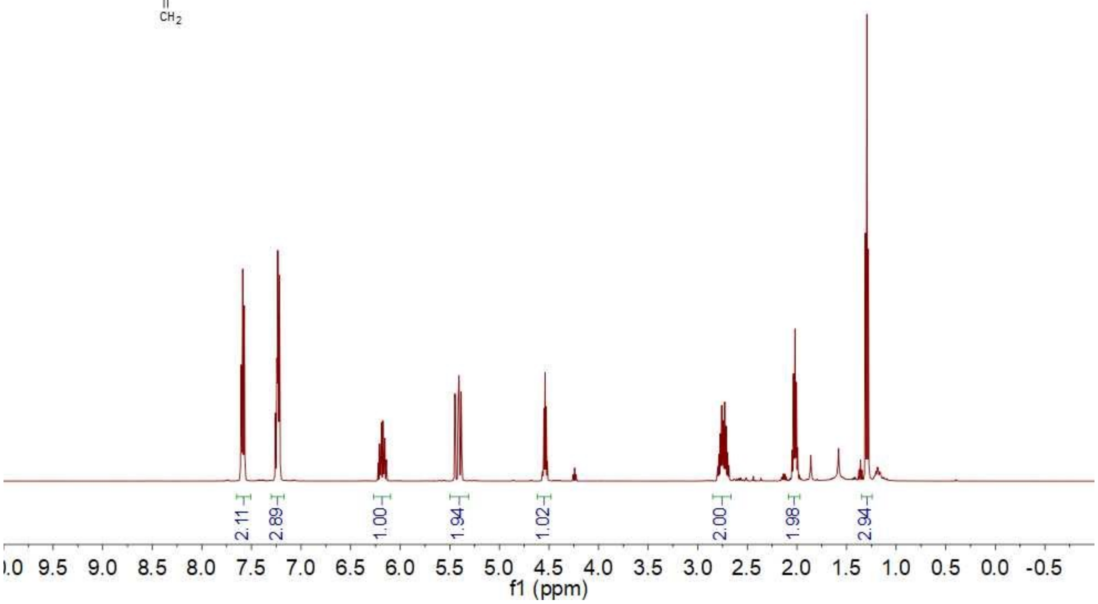
2.11
2.89
1.00
1.94
1.02
2.00
1.98
2.94
f1 (ppm)

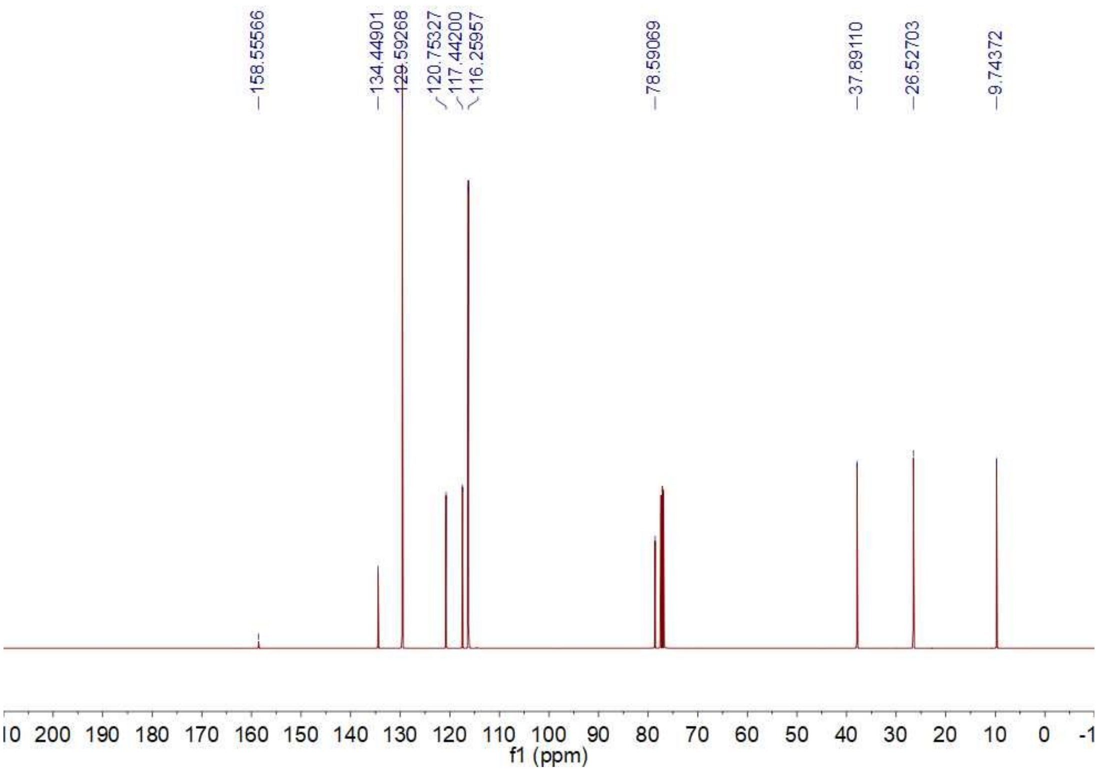
158.55566
134.44901
129.59268
120.75327
117.44200
116.25957
78.59069
37.89110
26.52703
9.74372
f1 (ppm)

(2s)

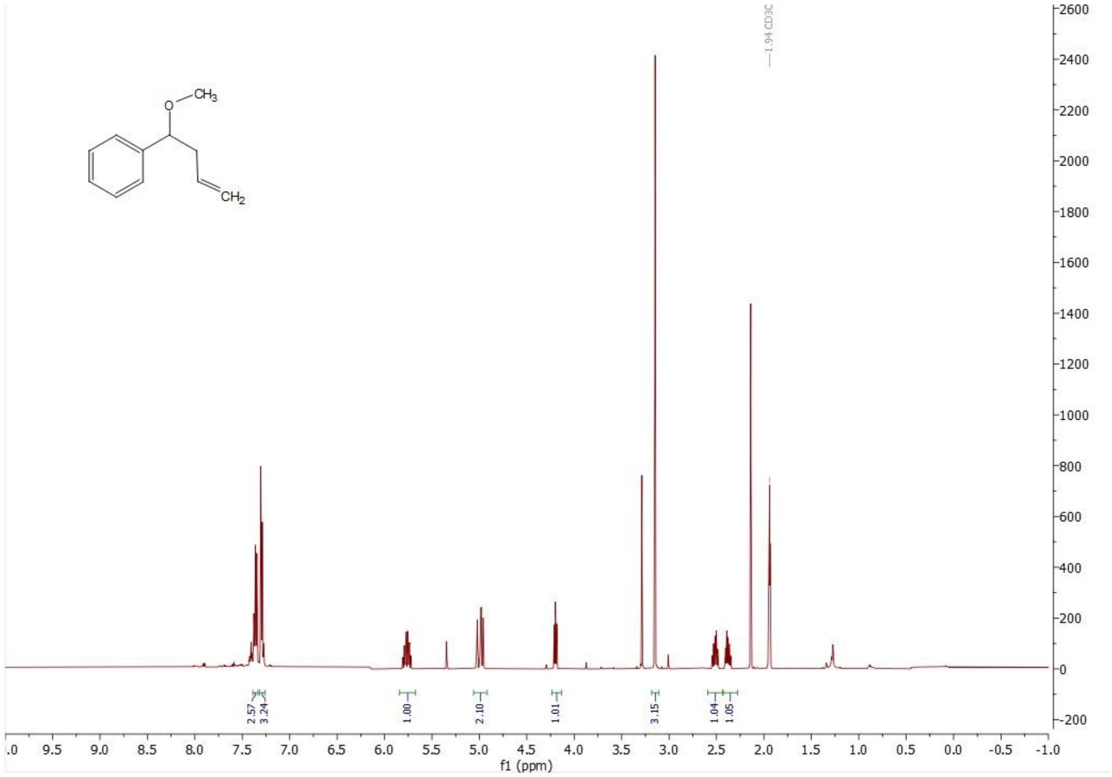

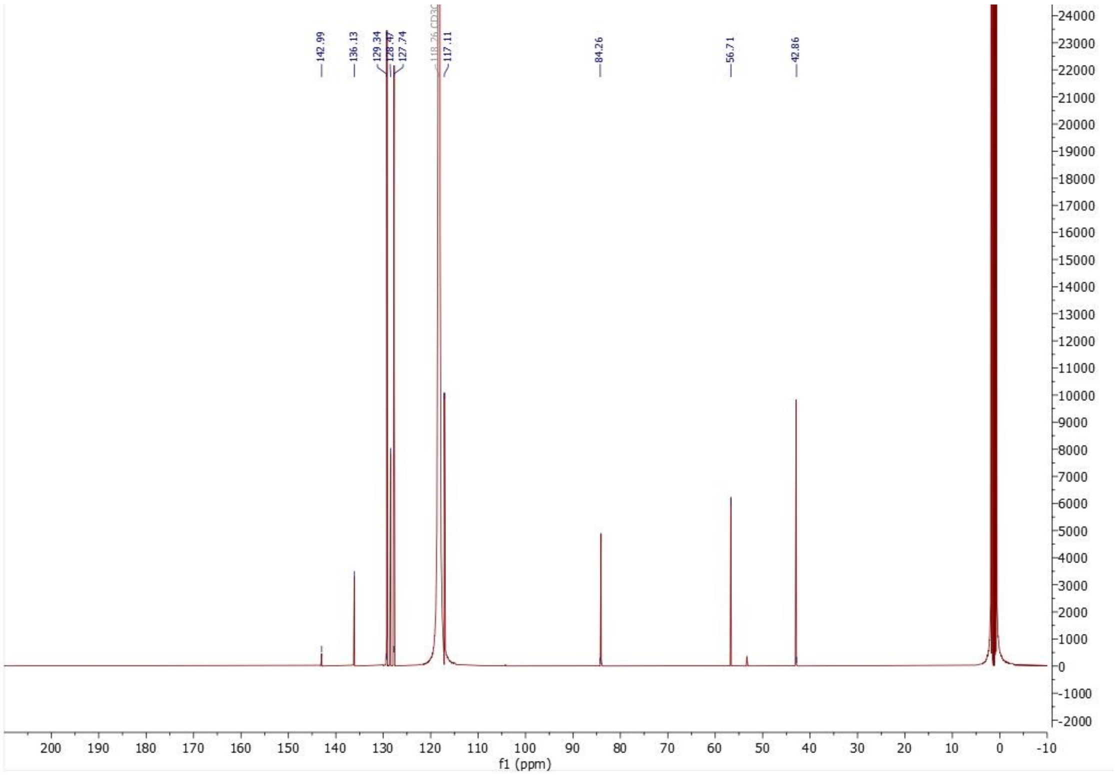

(2t)

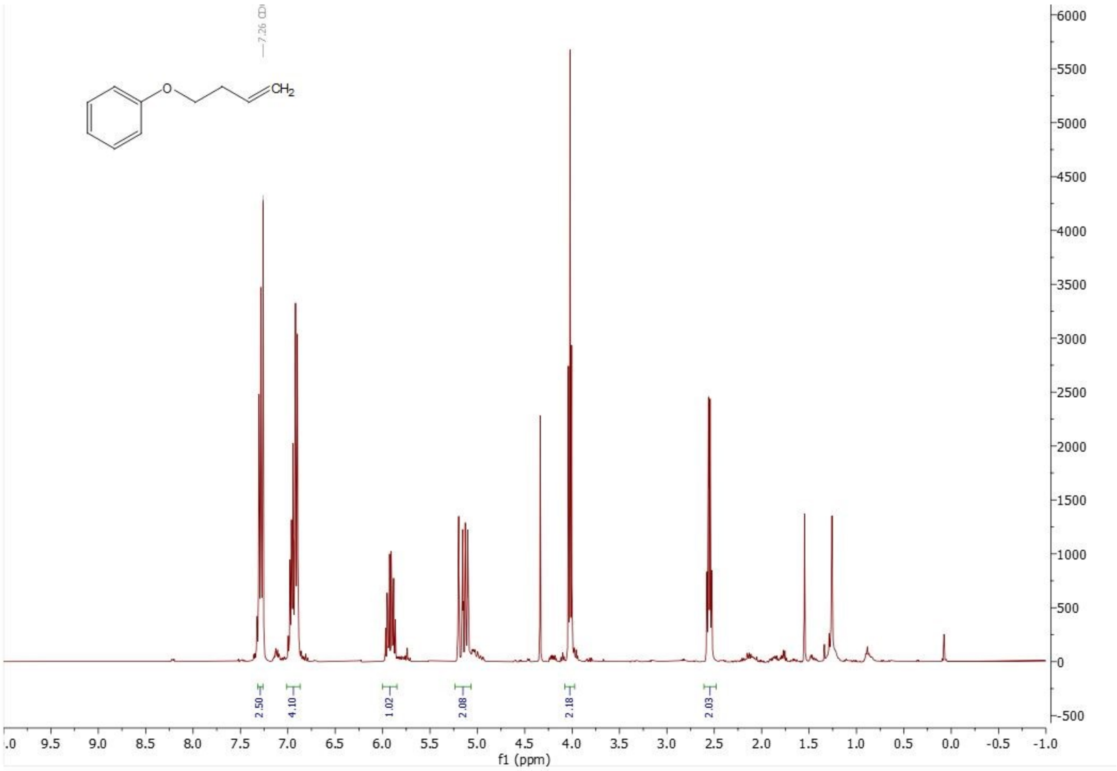
7.26 CDCl3
O
CH2
2.50
4.10
1.02
2.08
2.18
2.03
f1 (ppm)

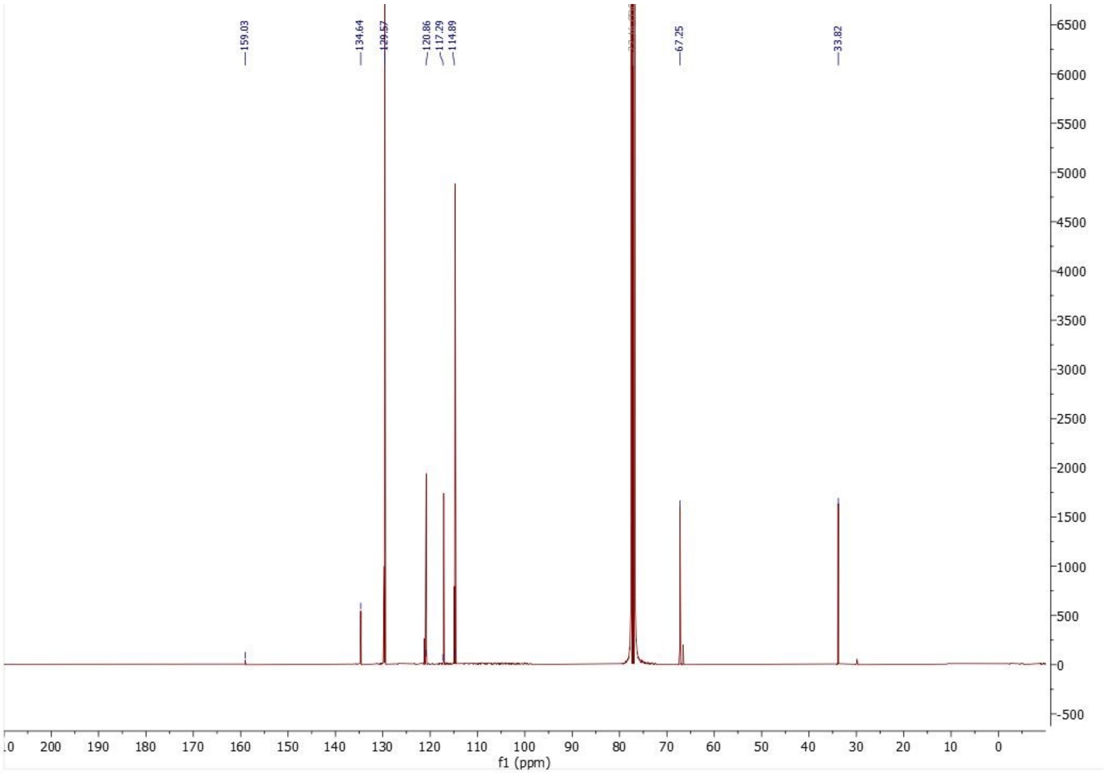
159.03
134.64
129.57
120.86
117.29
114.89
67.25
33.82
f1 (ppm)

(2v)

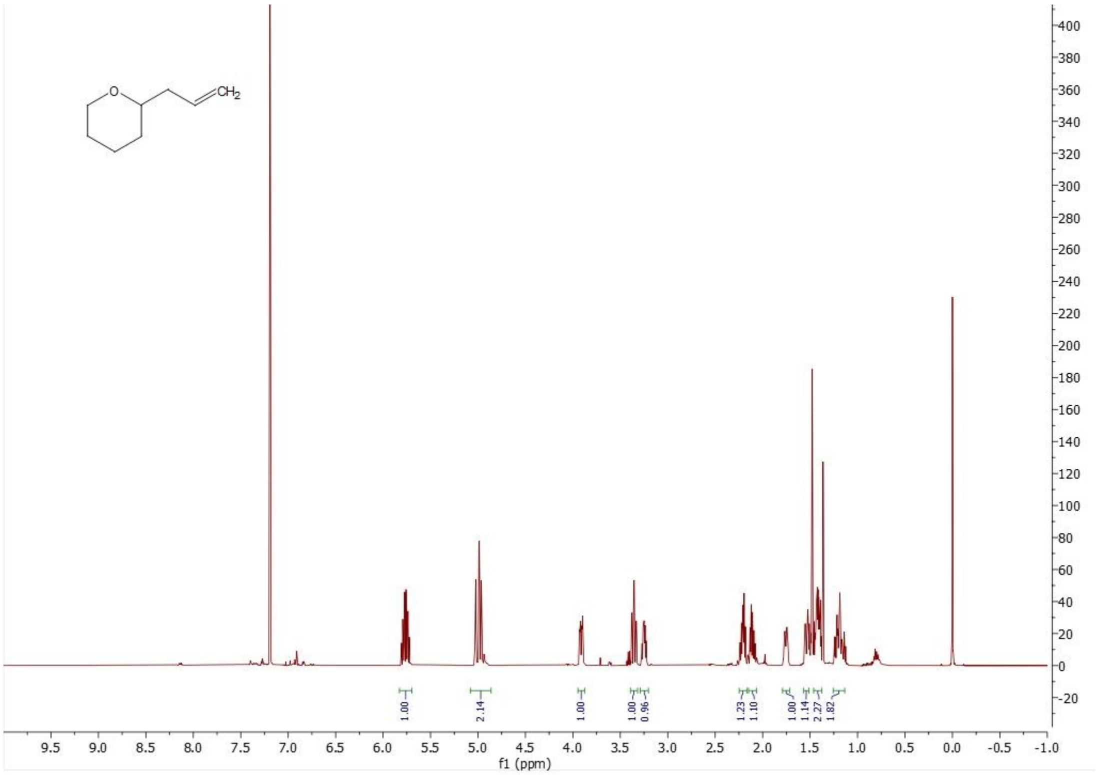
O
CH2
1.00
2.14
1.00
1.00
0.96
1.23
1.10
1.00
1.14
2.27
1.82
f1 (ppm)

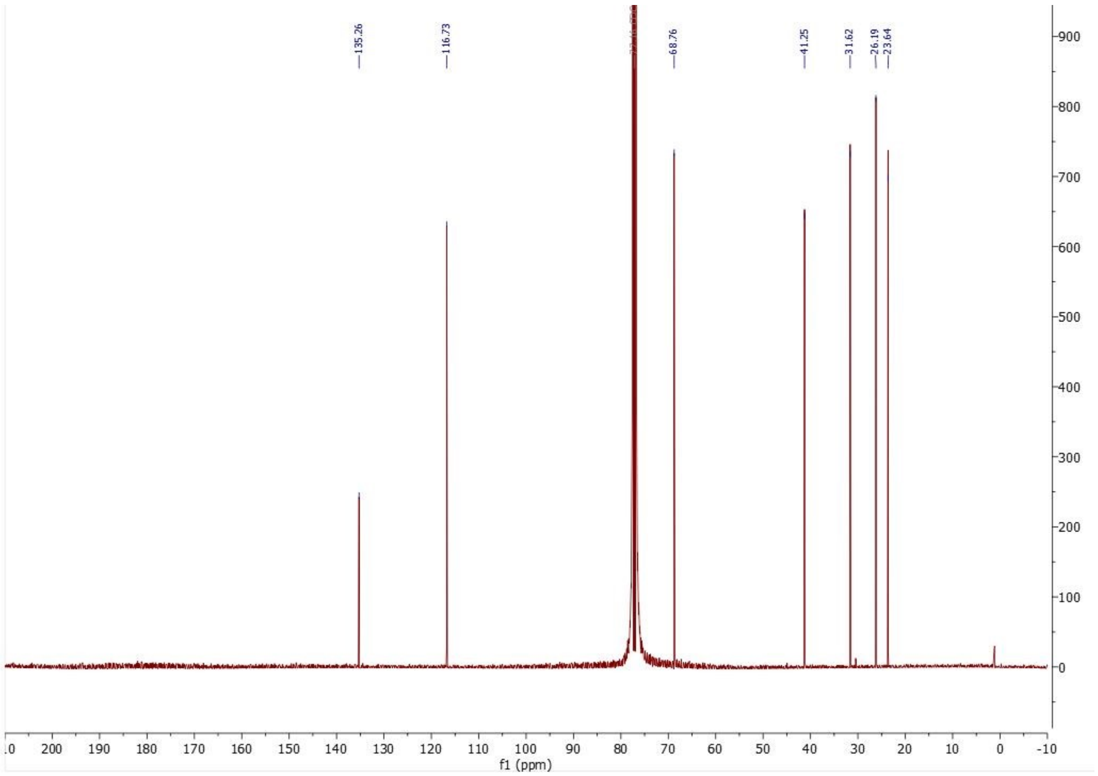
135.26
116.73
68.76
41.25
31.62
26.19
23.64
f1 (ppm)

(2w)

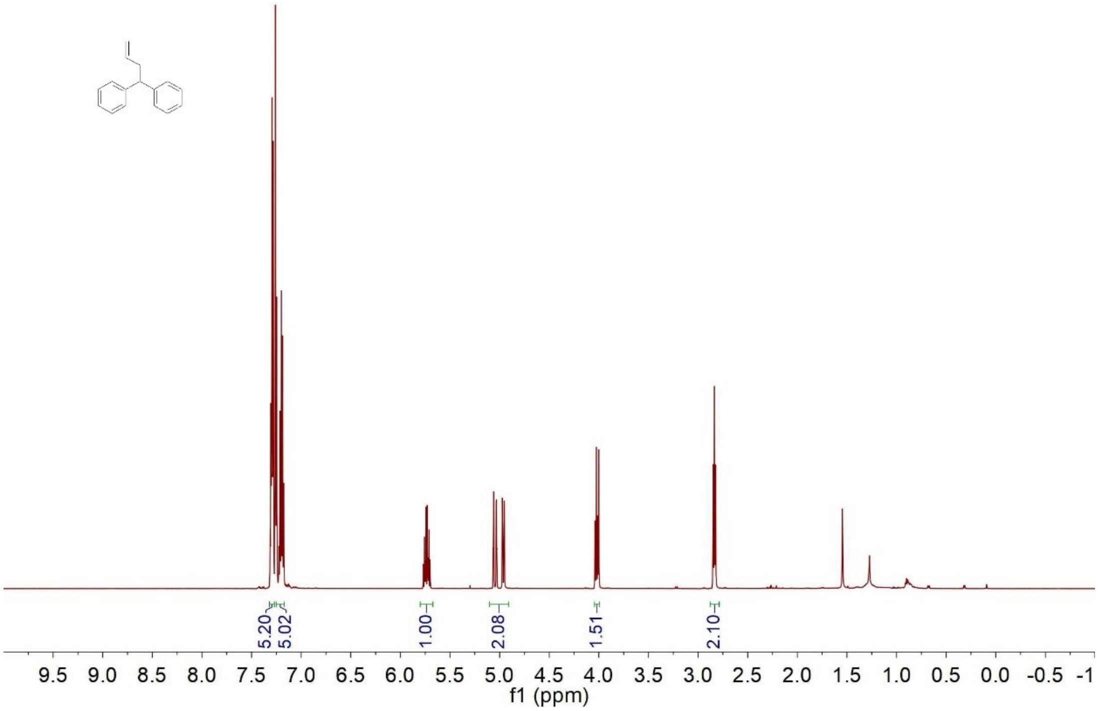

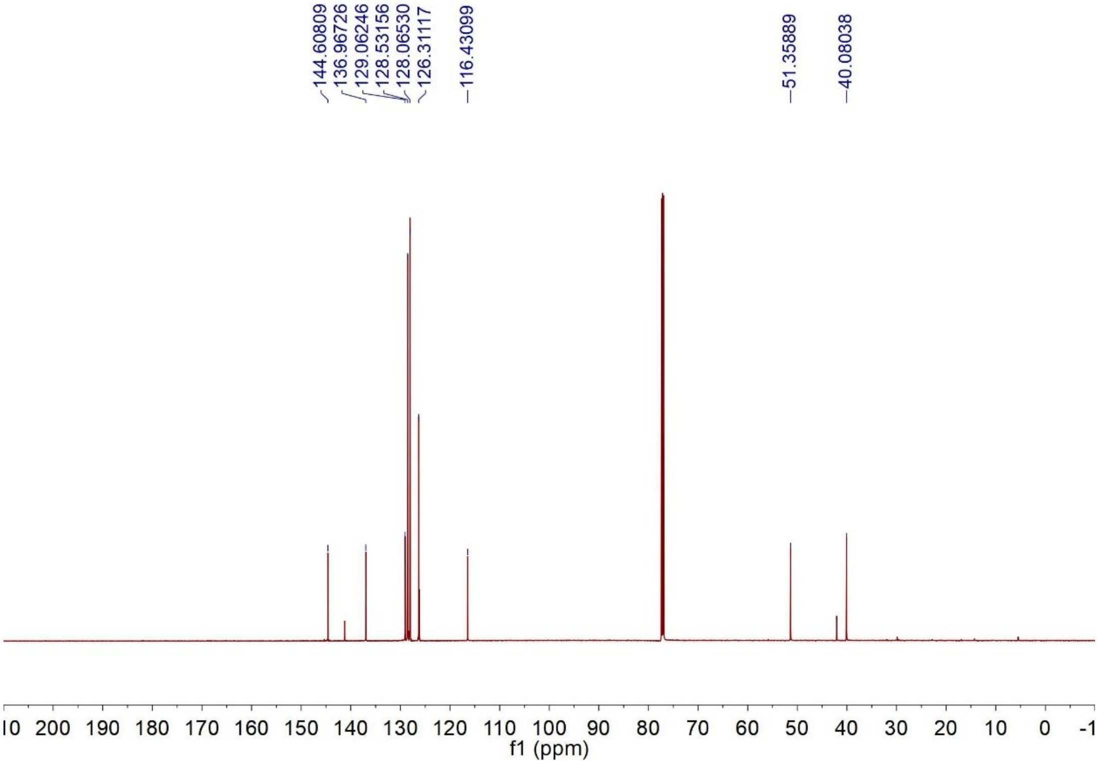

(2x) (isolated with alkene side product)

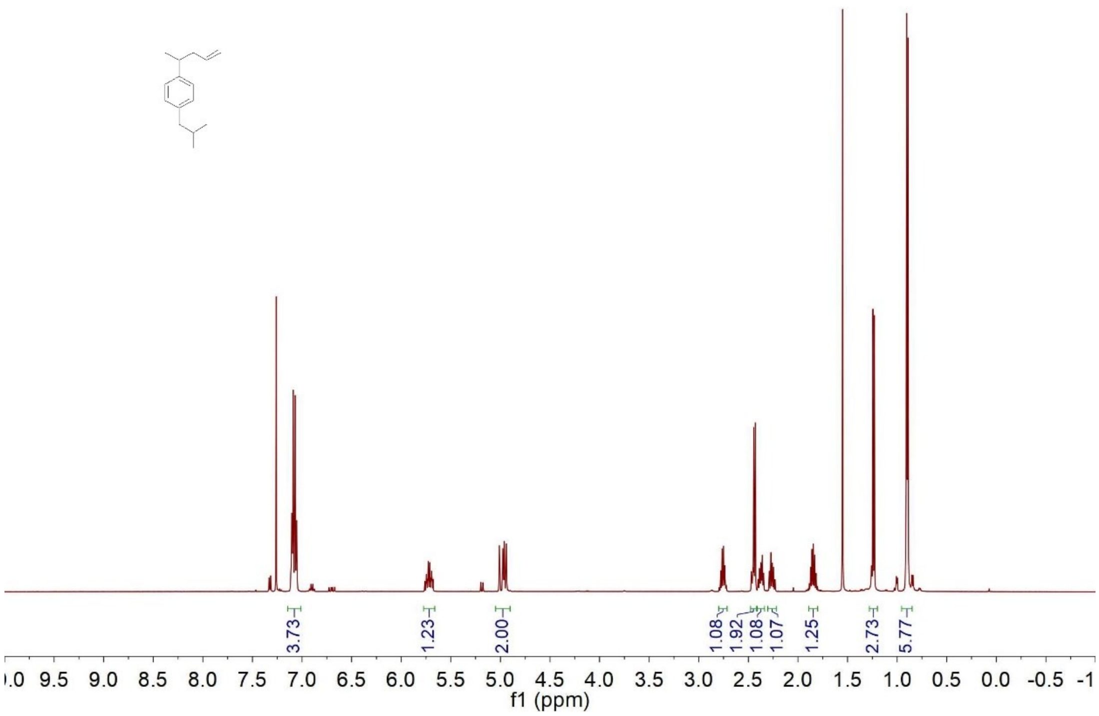

3.73
1.23
2.00
1.08
1.92
1.08
1.07
1.25
2.73
5.77
f1 (ppm)

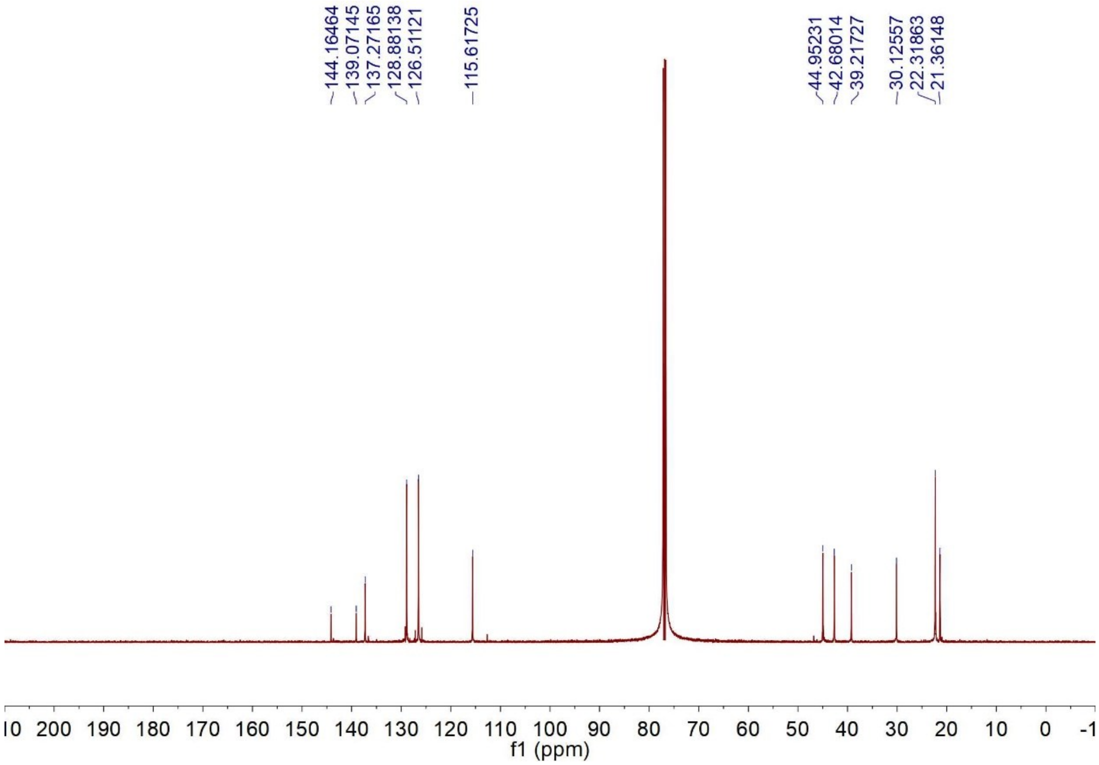

144.16464
139.07145
137.27165
128.88138
126.51121
115.61725
44.95231
42.68014
39.21727
30.12557
22.31863
21.36148
f1 (ppm)

(2y) (isolated with alkane side product)

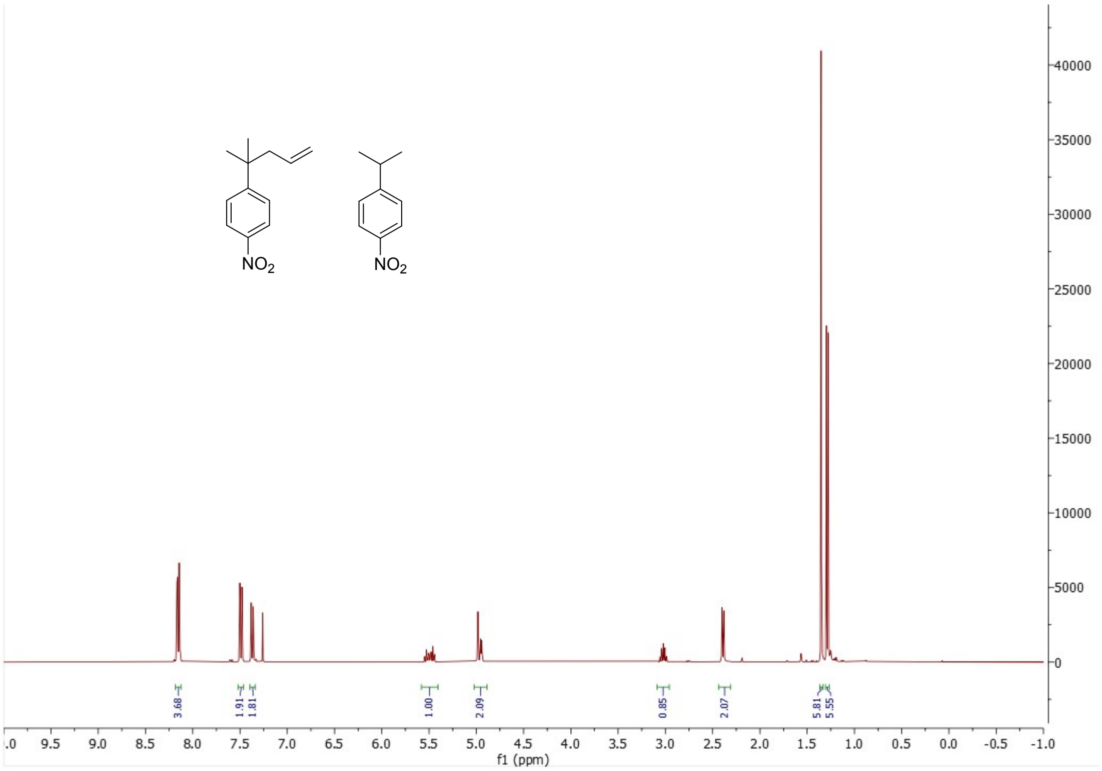

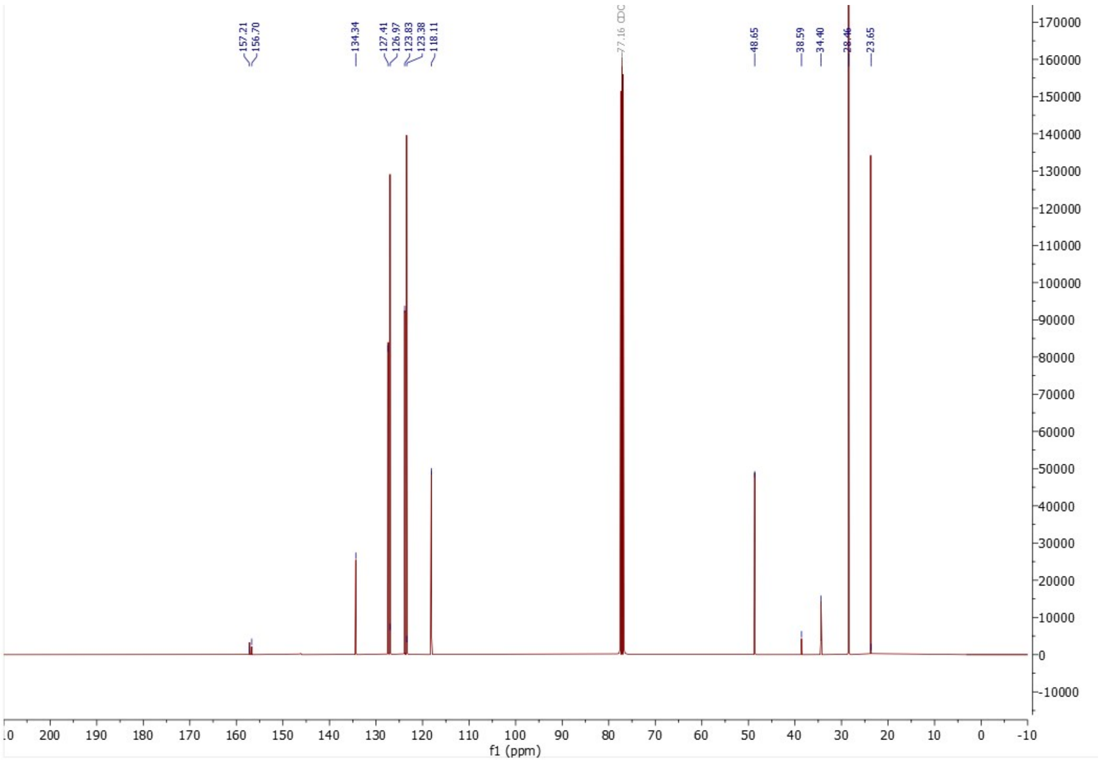

(2z)

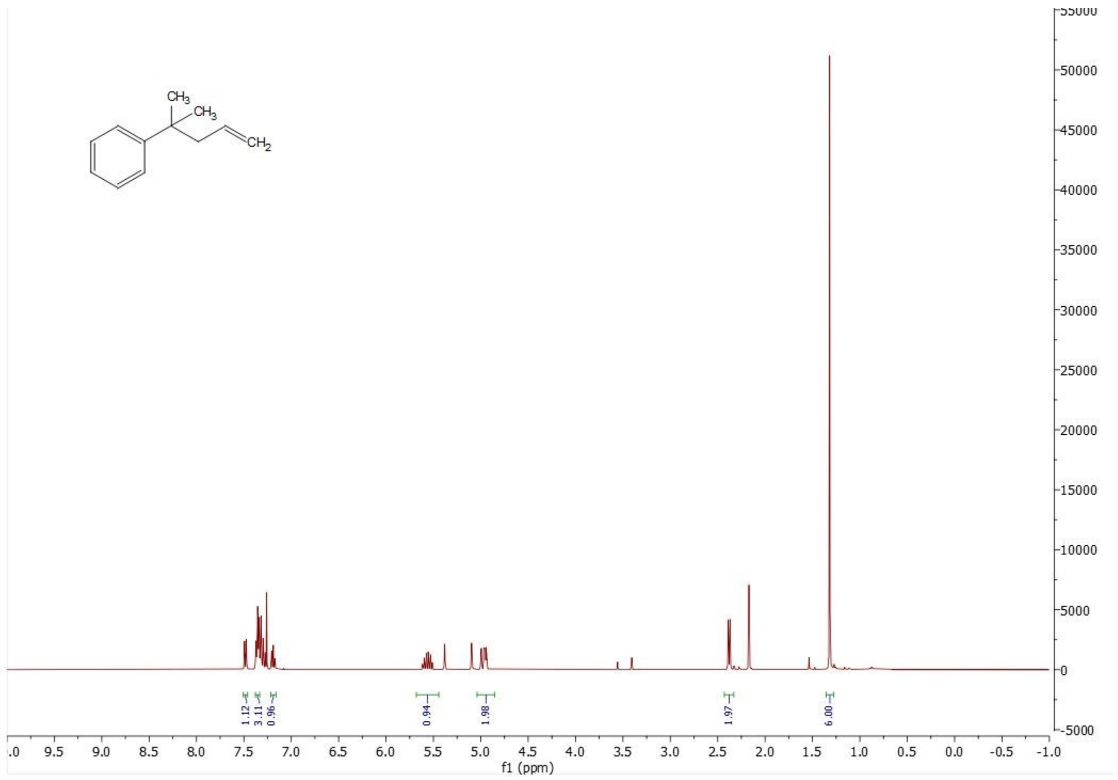

CH_3
CH_3
CH_2
1.12
3.11
0.96
0.94
1.98
1.97
6.00
f1 (ppm)

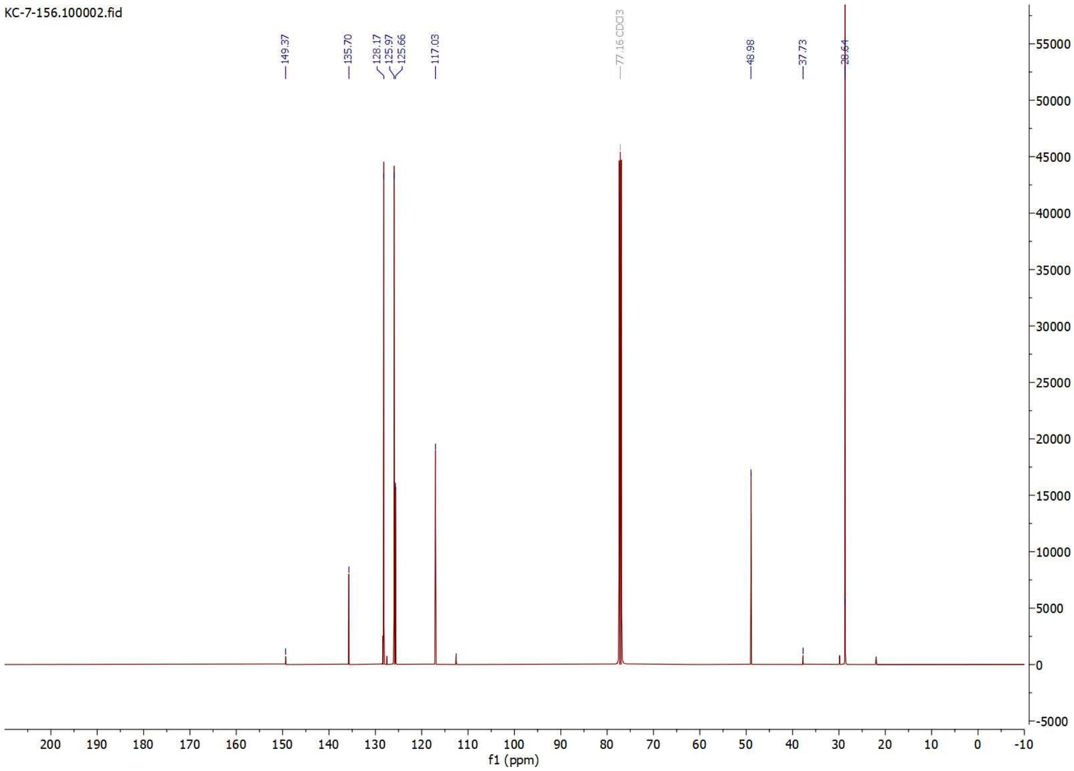

KC-7-156.100002.fid
149.37
135.70
128.17
125.97
125.66
117.03
77.16 CDCl3
48.98
37.73
28.64
f1 (ppm)

(2aa)

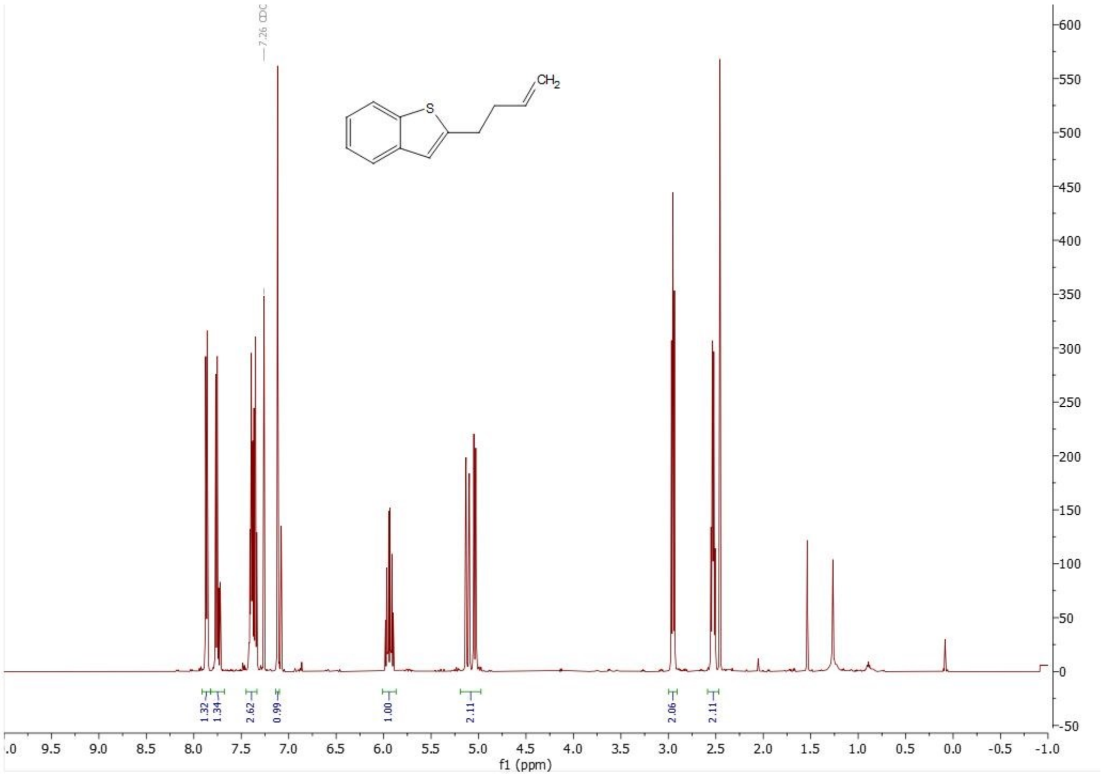
7.26 CDC
CH2
S
1.32
1.34
2.62
0.99
1.00
2.11
2.06
2.11
f1 (ppm)

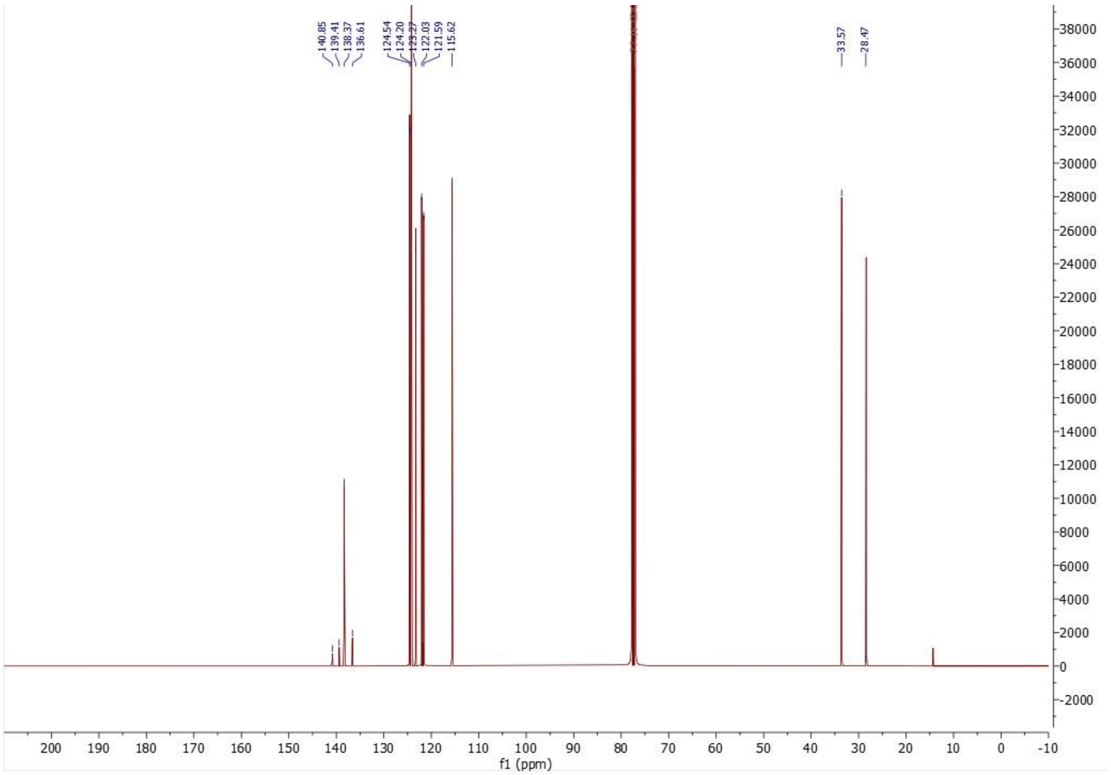
140.85
139.41
138.37
136.61
124.54
124.20
123.27
122.03
121.59
115.62
33.57
28.47
f1 (ppm)

(2bb)

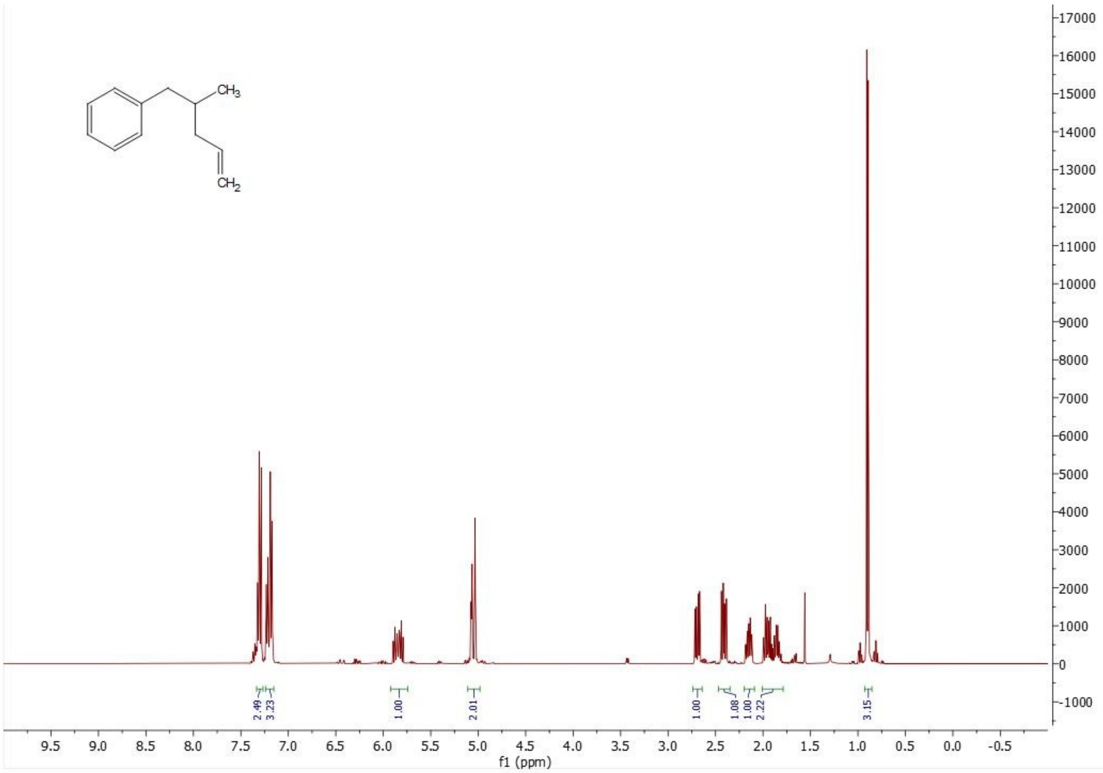
CH3
CH2
2.49
3.23
1.00
2.01
1.00
1.08
1.00
2.22
3.15
f1 (ppm)

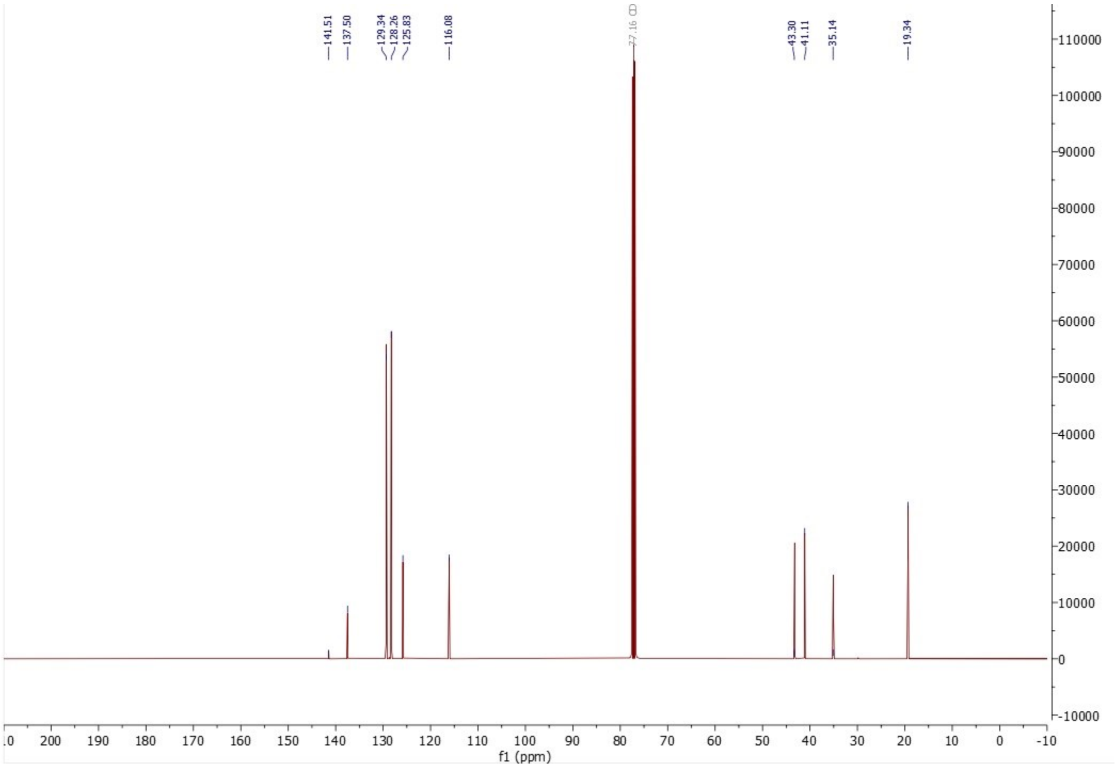
141.51
137.50
129.34
128.26
125.83
116.08
43.30
41.11
35.14
19.34
f1 (ppm)

(2cc)

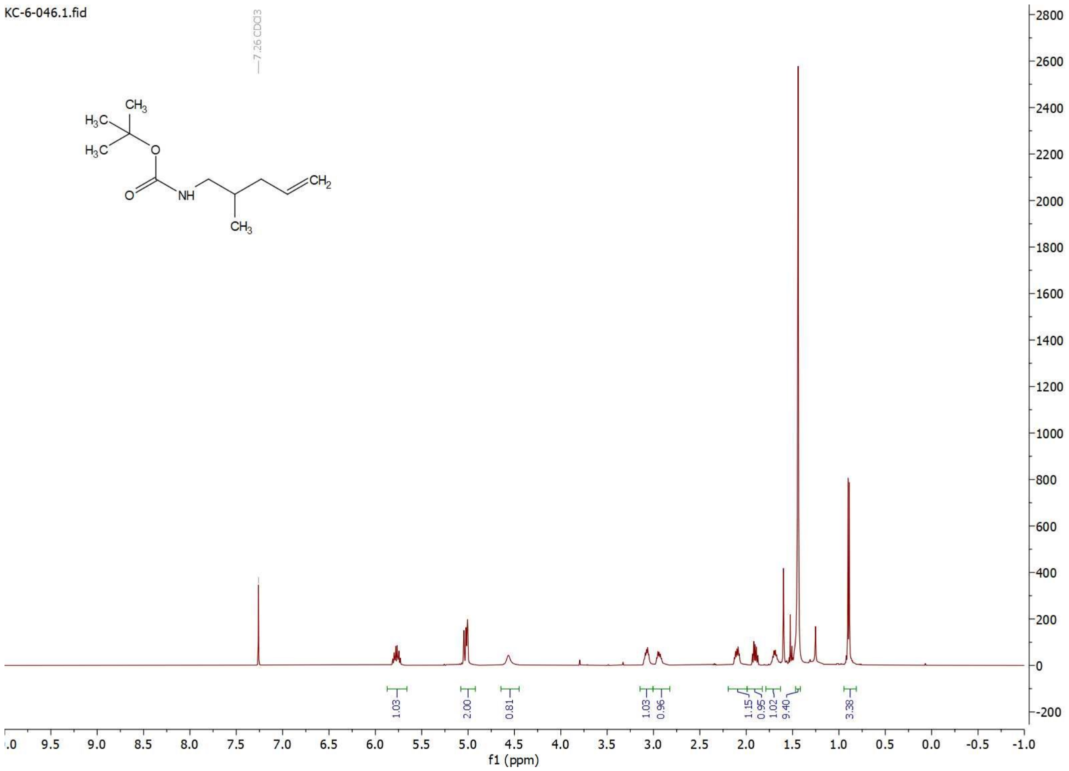
KC-6-046.1.fid
7.26 CDCl3
1.03
2.00
0.81
1.03
0.96
1.15
0.95
1.02
9.40
3.38
f1 (ppm)

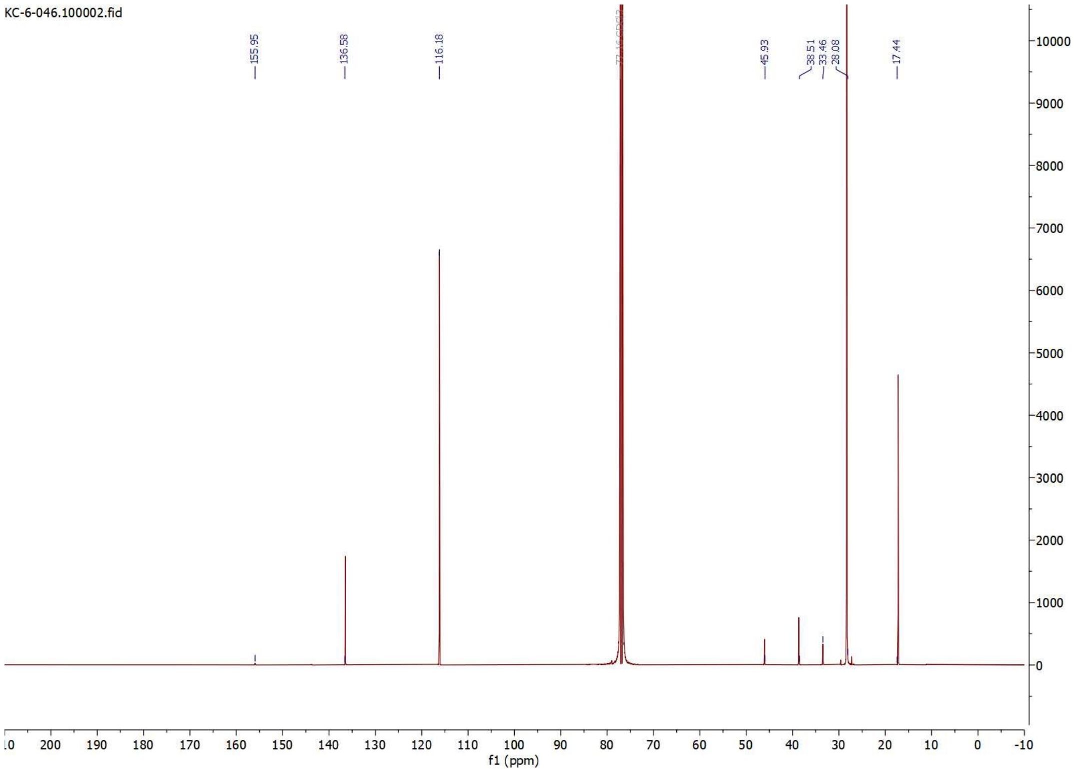
KC-6-046.100002.fid
155.95
136.58
116.18
45.93
38.51
33.46
28.08
17.44
f1 (ppm)

(2dd)

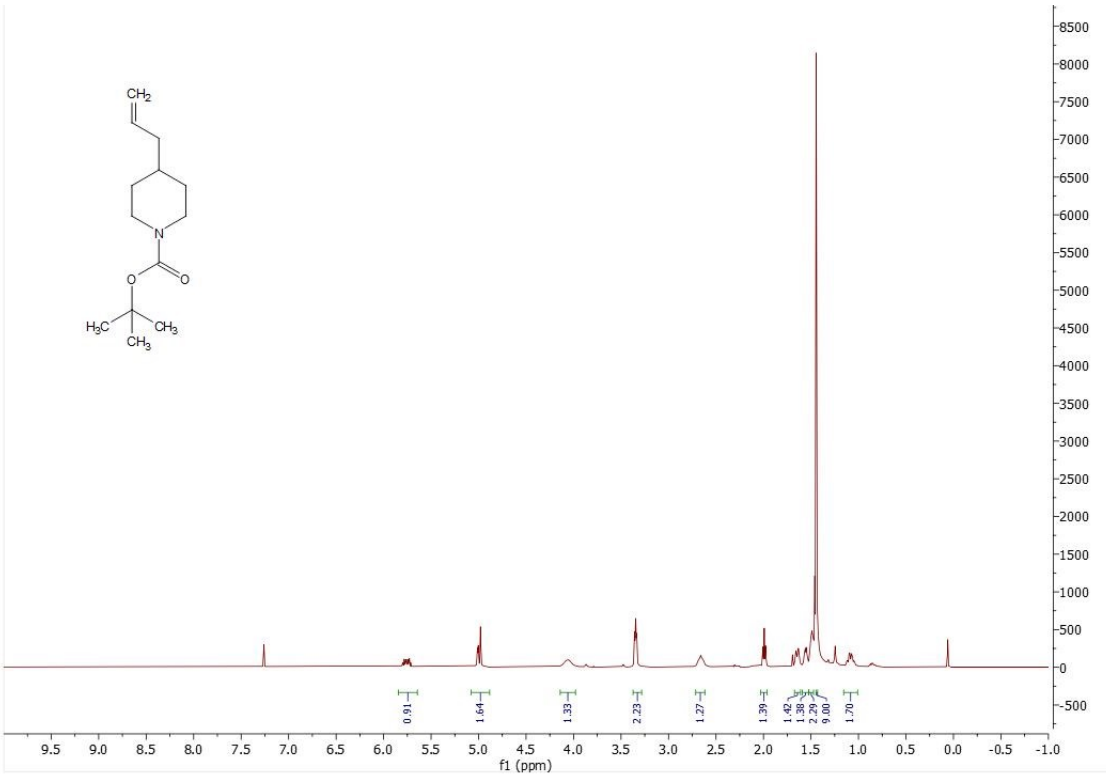
CH2
N
O
O
H3C
CH3
CH3
0.91
1.64
1.33
2.23
1.27
1.39
1.42
1.38
2.29
9.00
1.70
f1 (ppm)

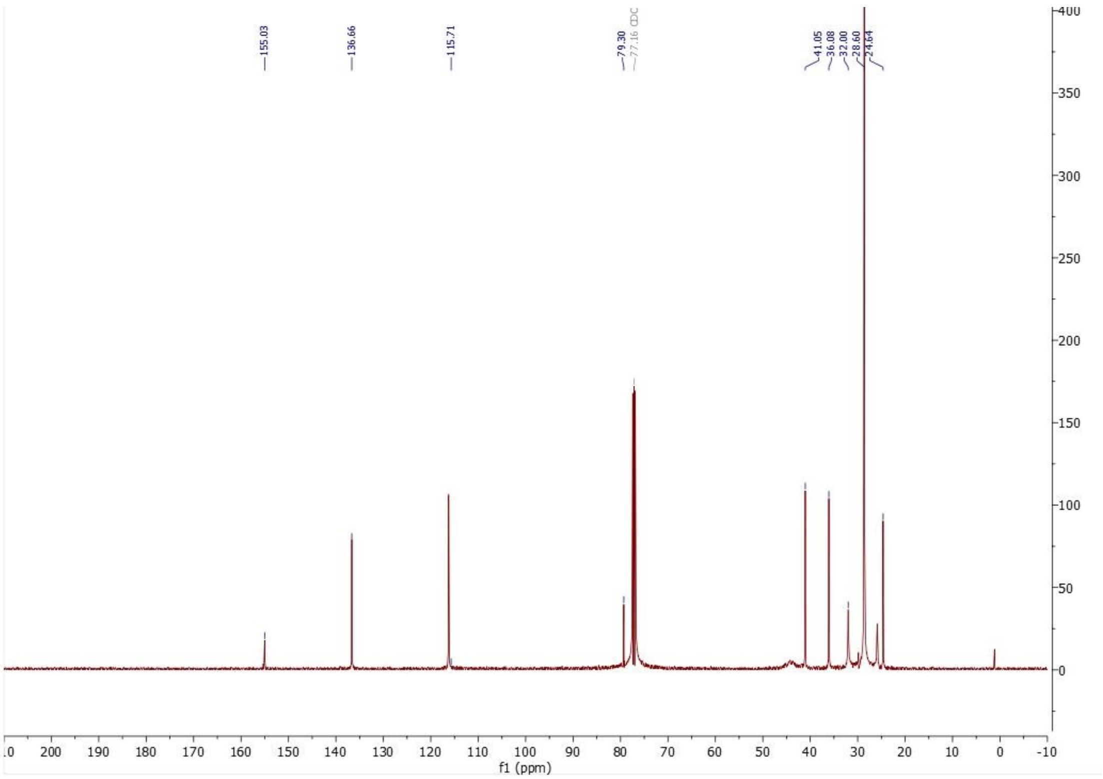
155.03
136.66
115.71
79.30
77.16 CDC
41.05
36.08
32.00
28.60
24.64
f1 (ppm)

(2ee)

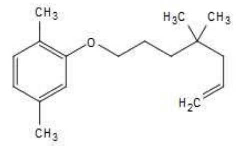
CH3
H3C CH3
O
H2C
CH3

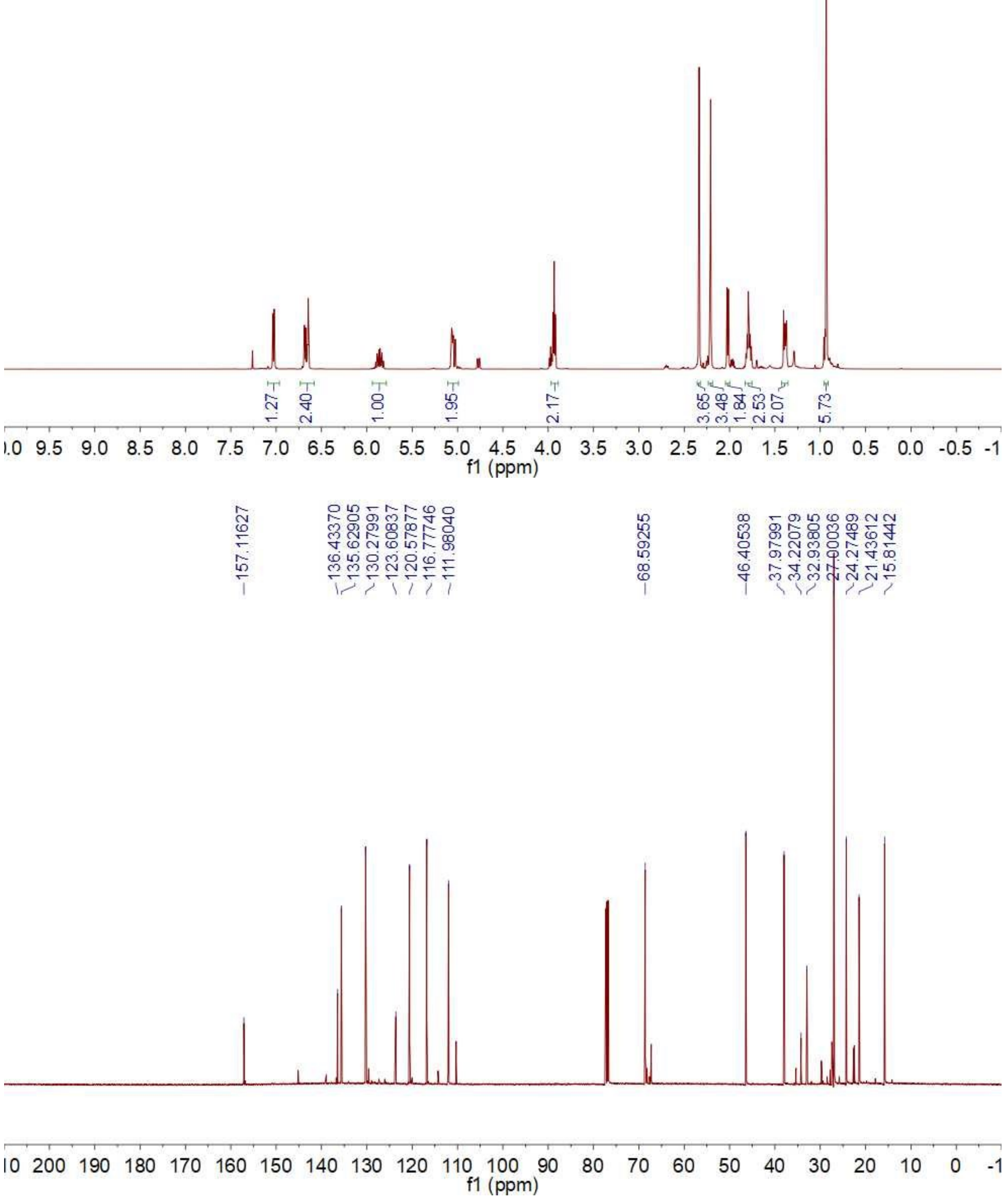
1.27
2.40
1.00
1.95
2.17
3.65
3.48
1.84
2.53
2.07
5.73
f1 (ppm)
157.11627
136.43370
135.62905
130.27991
123.60837
120.57877
116.77746
111.98040
68.59255
46.40538
37.97991
34.22079
32.93805
27.00036
24.27489
21.43612
15.81442
f1 (ppm)

(2ff)

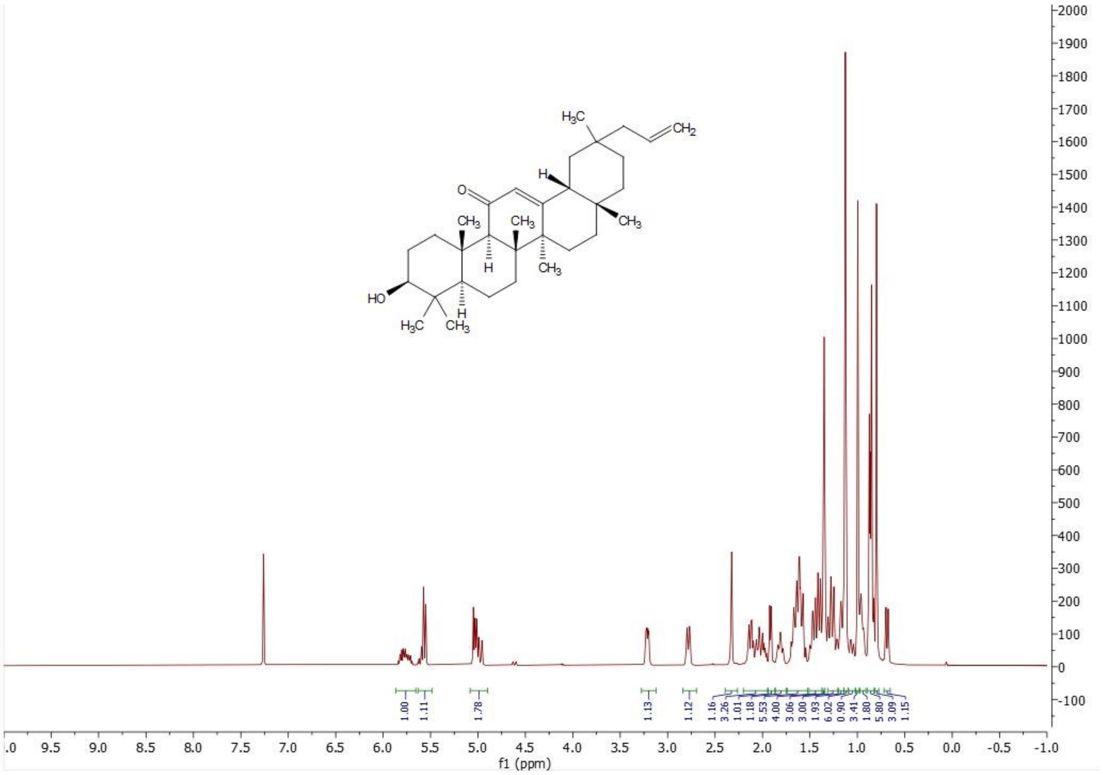

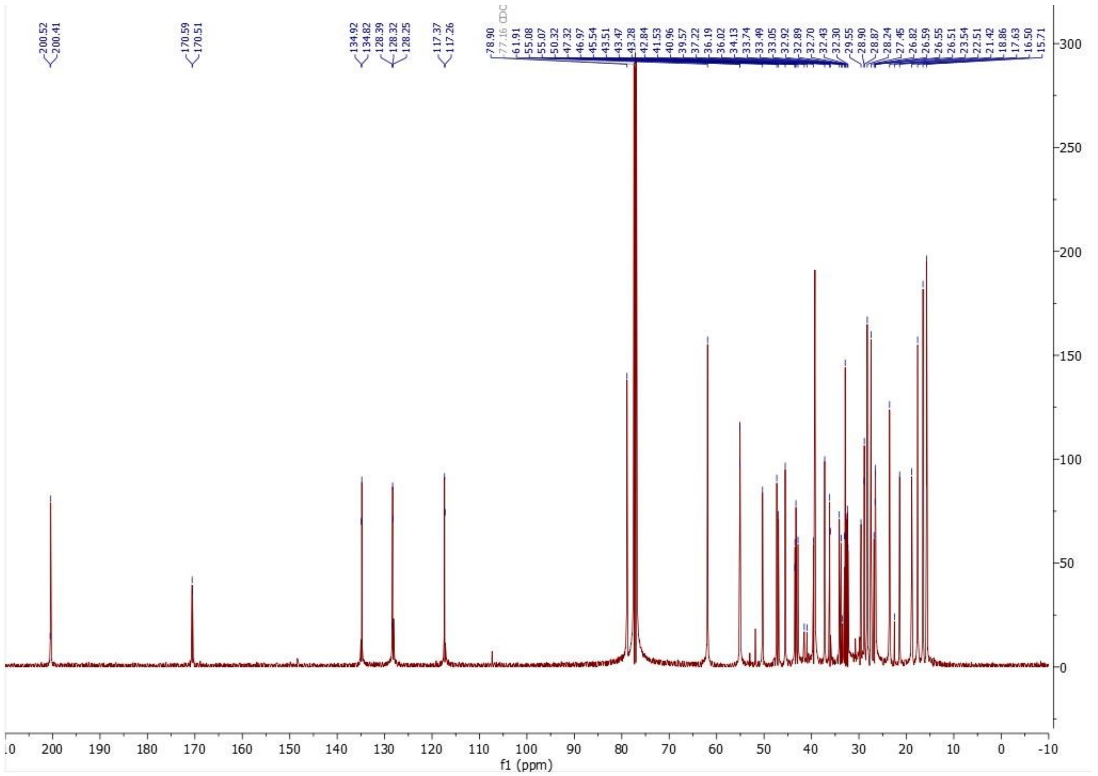

(2gg) (isolated with alkane product)

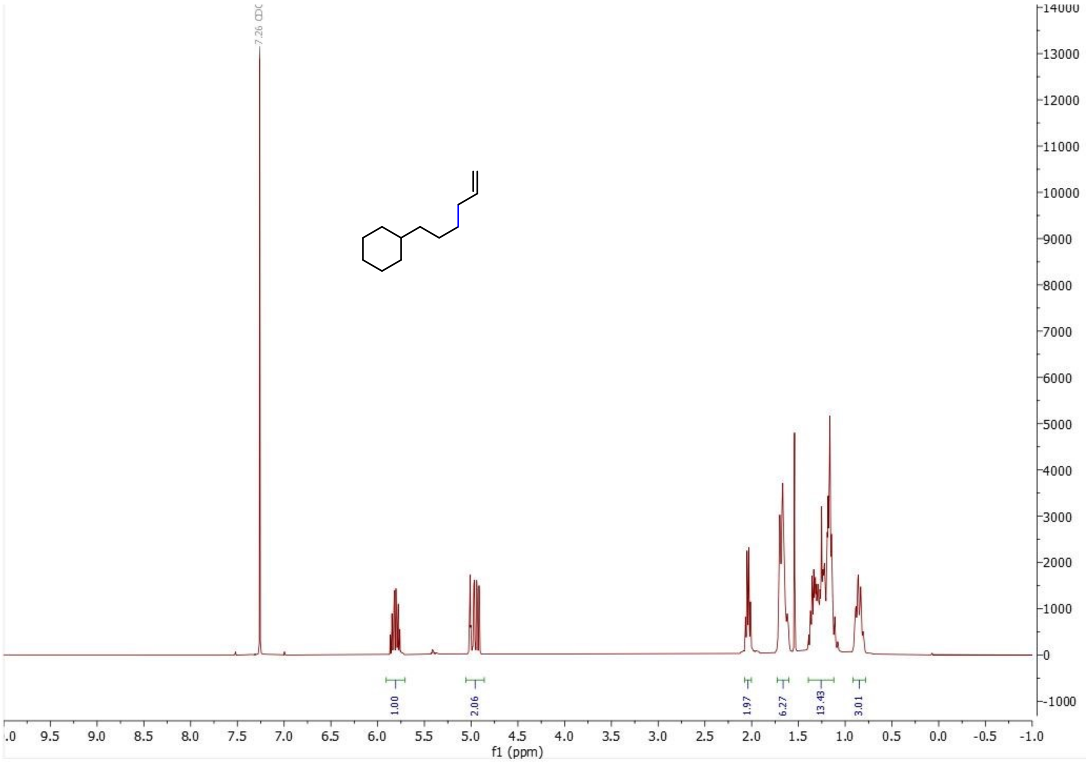
7.26 CDC
1.00
2.06
1.97
6.27
13.43
3.01
f1 (ppm)

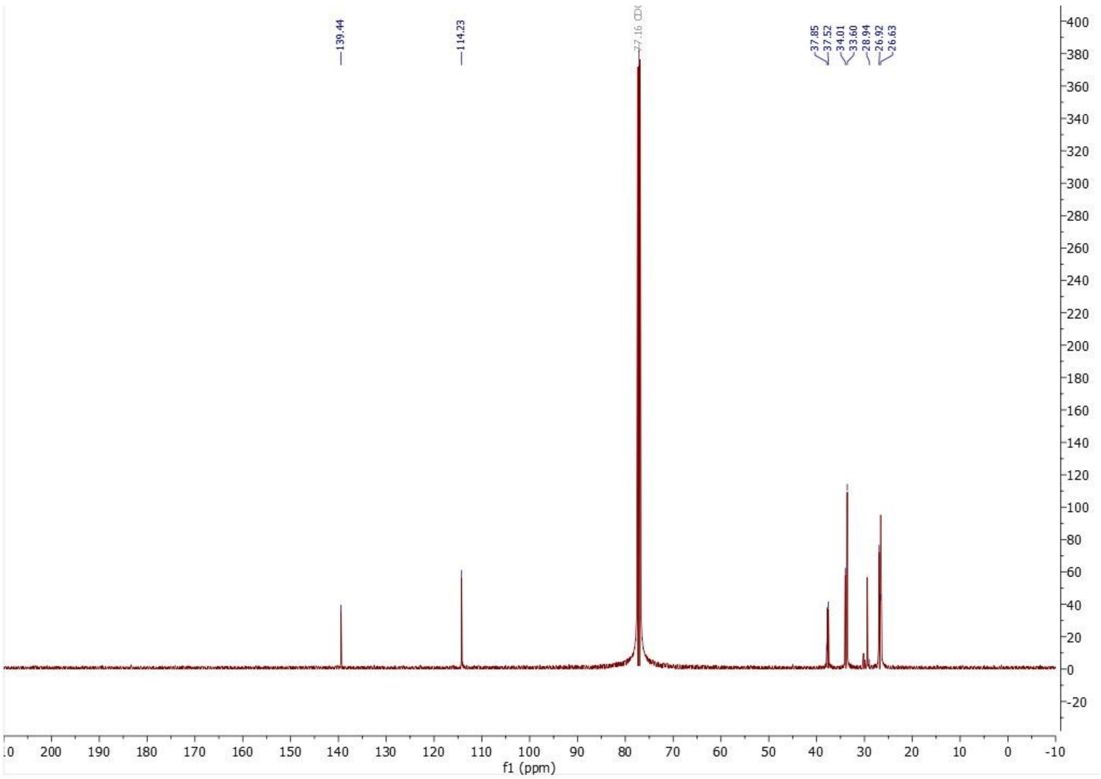
139.44
114.23
77.16 CDC
37.85
37.52
34.01
33.60
28.94
26.92
26.63
f1 (ppm)

(4b)

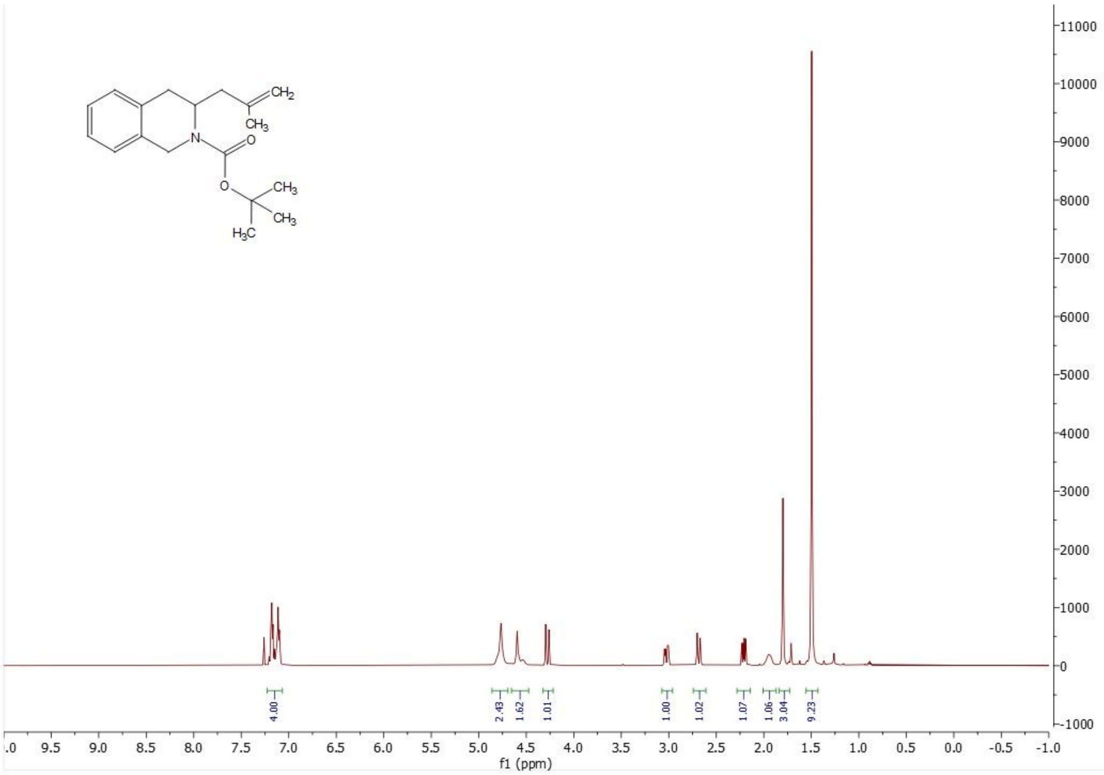

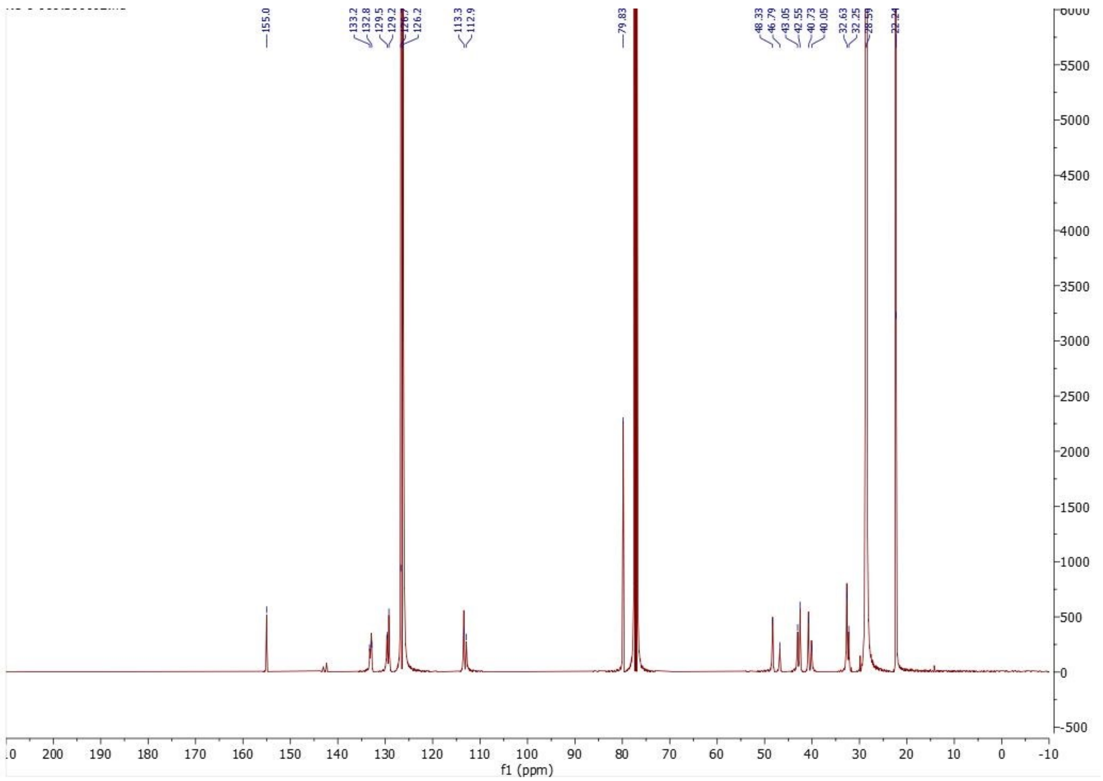

(4c) (mix of rotamers)

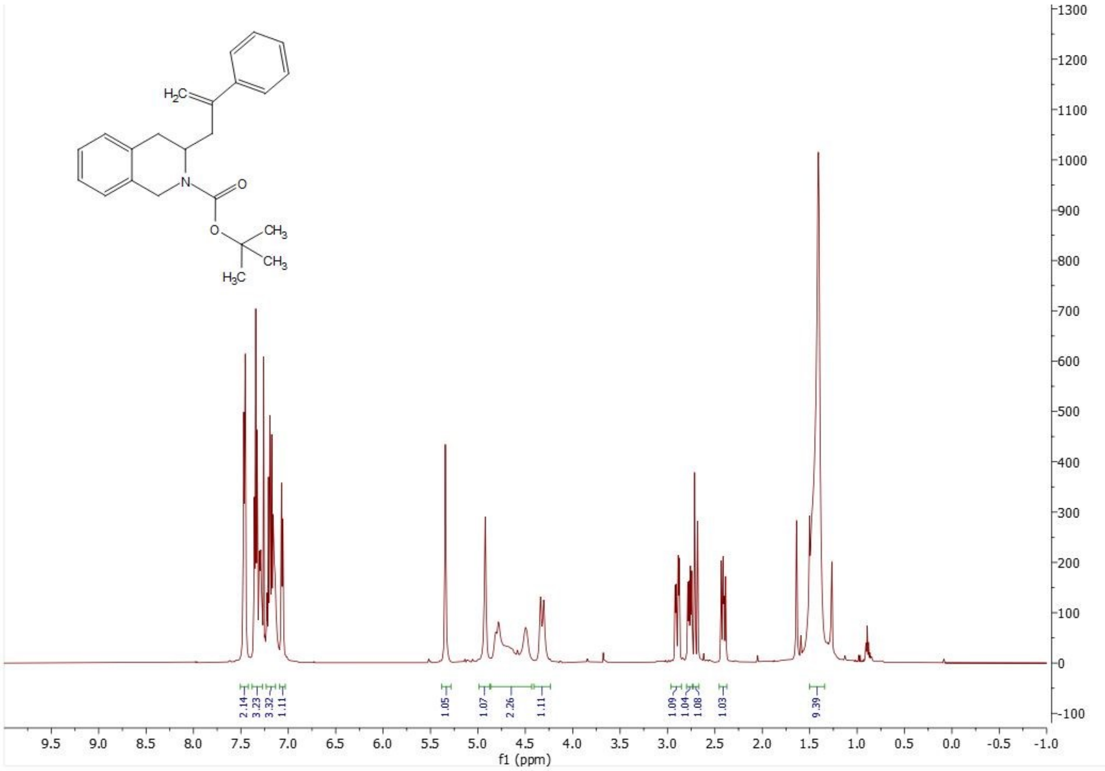

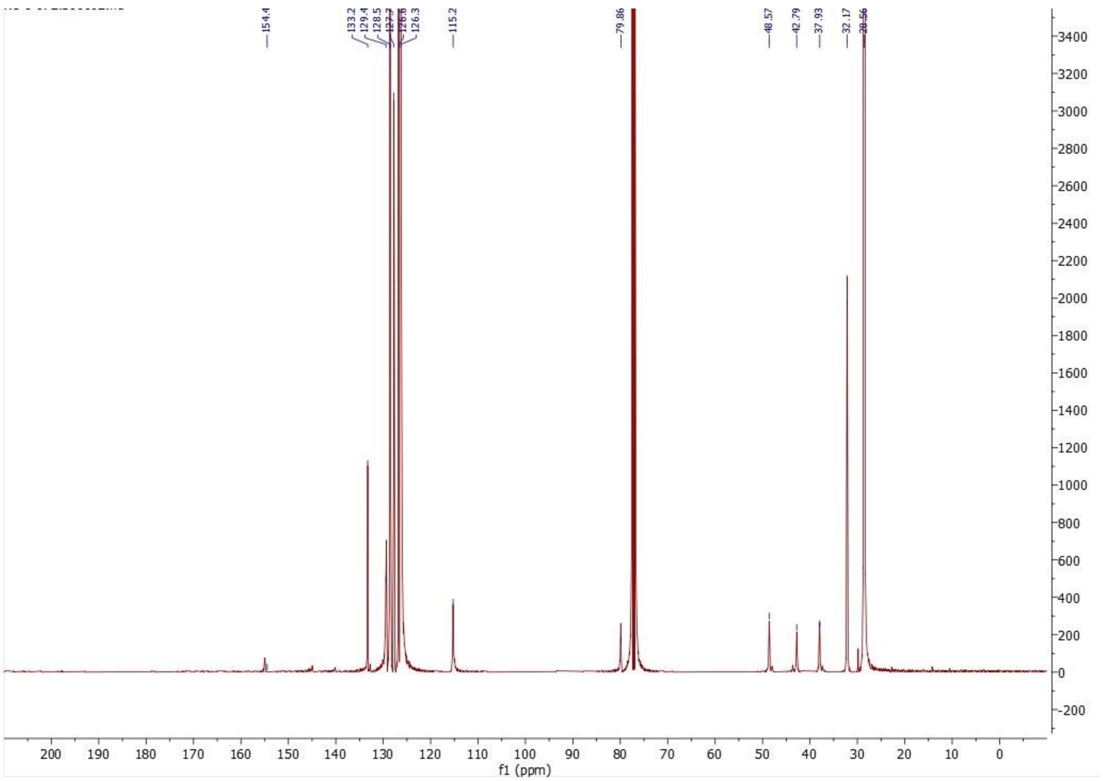

(4d) (mix of rotamers)

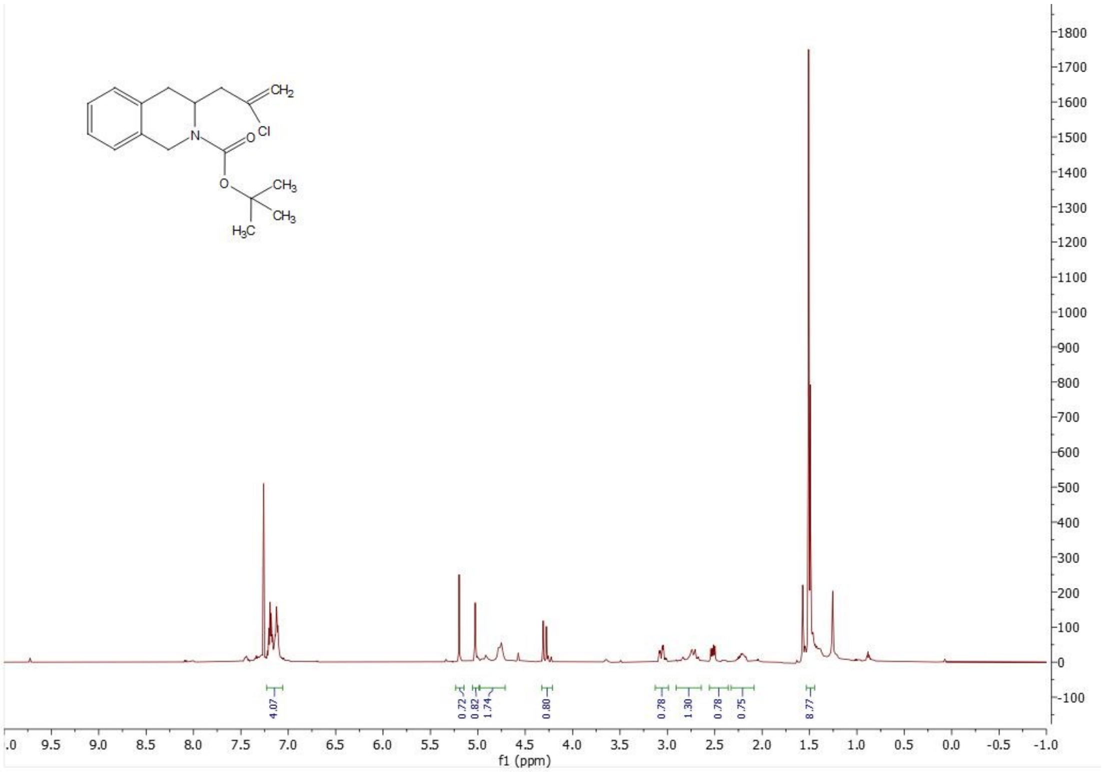

CH2
Cl
N
O
O
CH3
CH3
H3C
4.07
0.72
0.82
1.74
0.80
0.78
1.30
0.78
0.75
8.77
f1 (ppm)

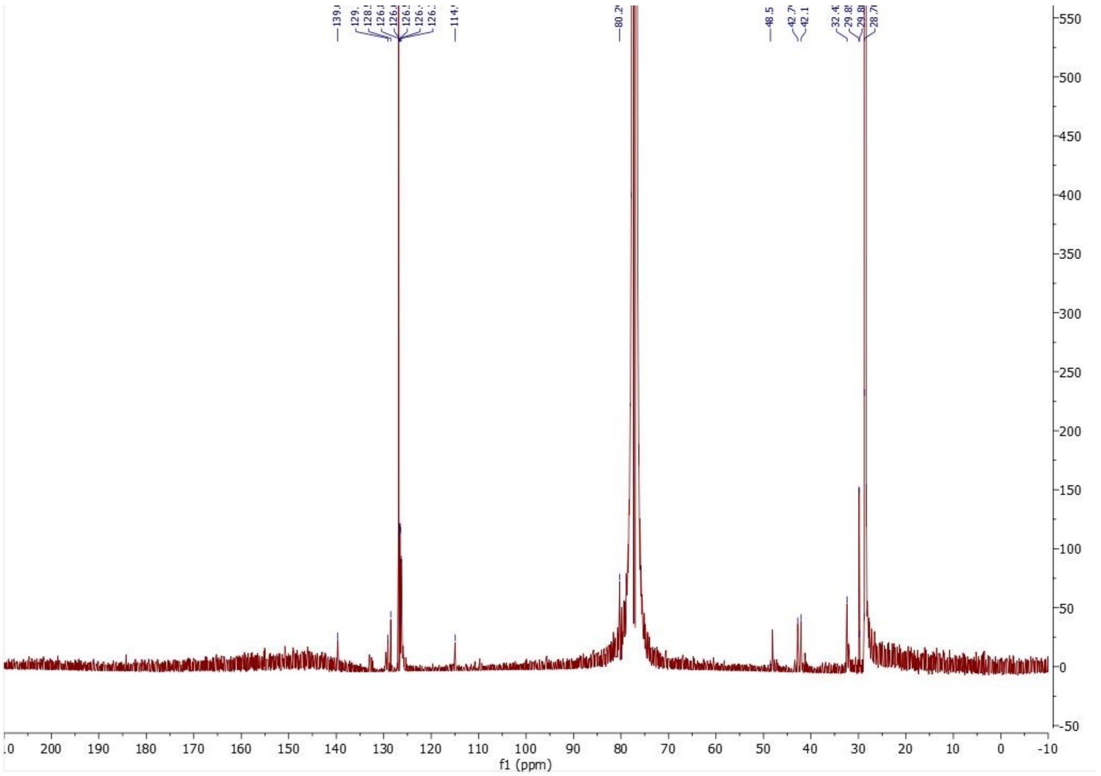

80.2
48.5
42.1
f1 (ppm)

(4e)

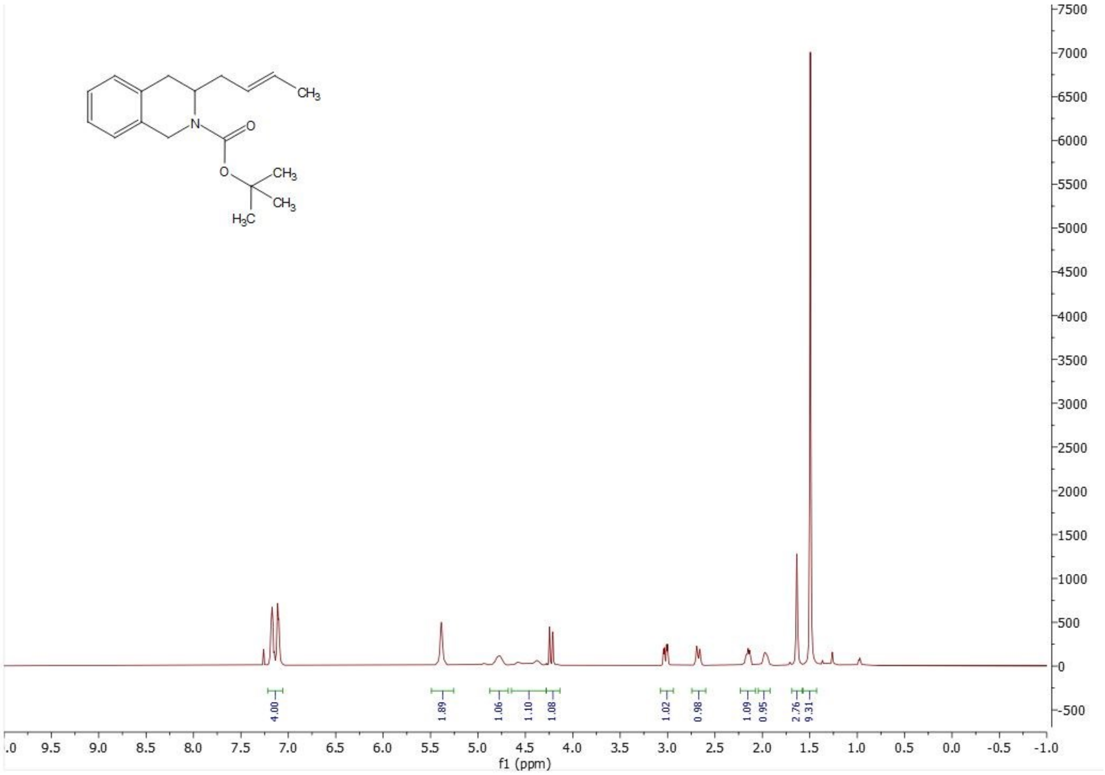
CH3
O
CH3
CH3
H3C
N
O
4.00
1.89
1.06
1.10
1.08
1.02
0.98
1.09
0.95
2.76
9.31
f1 (ppm)

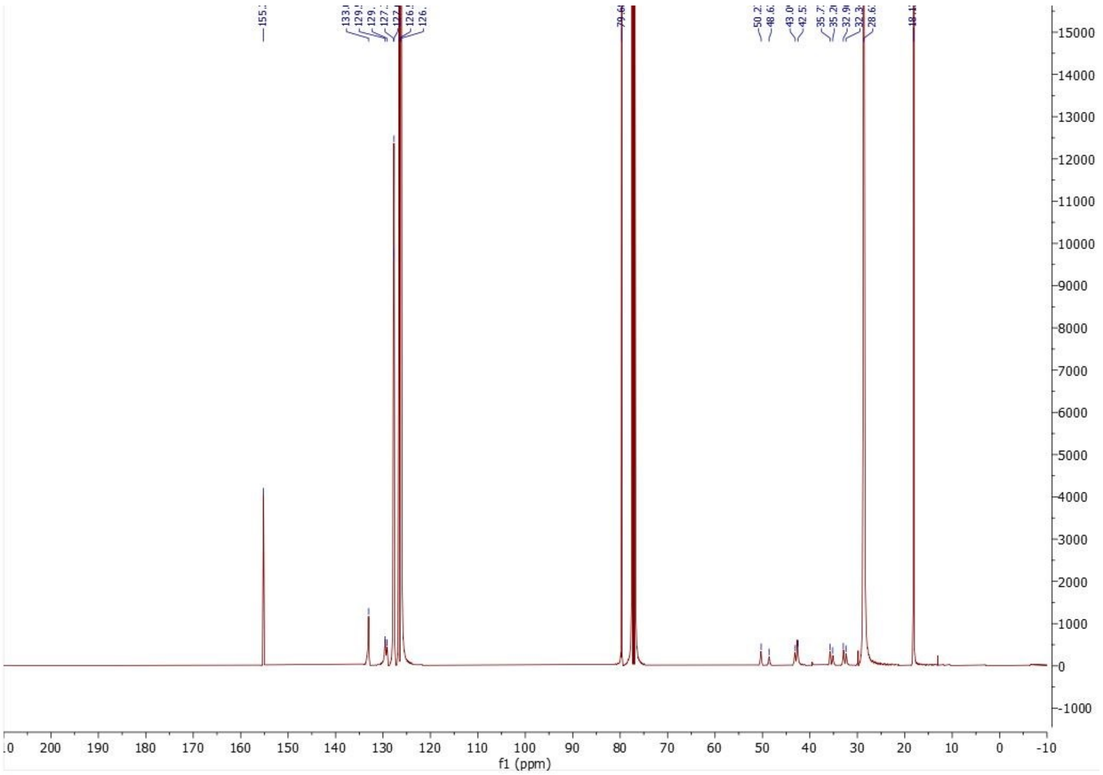
f1 (ppm)

(4f)

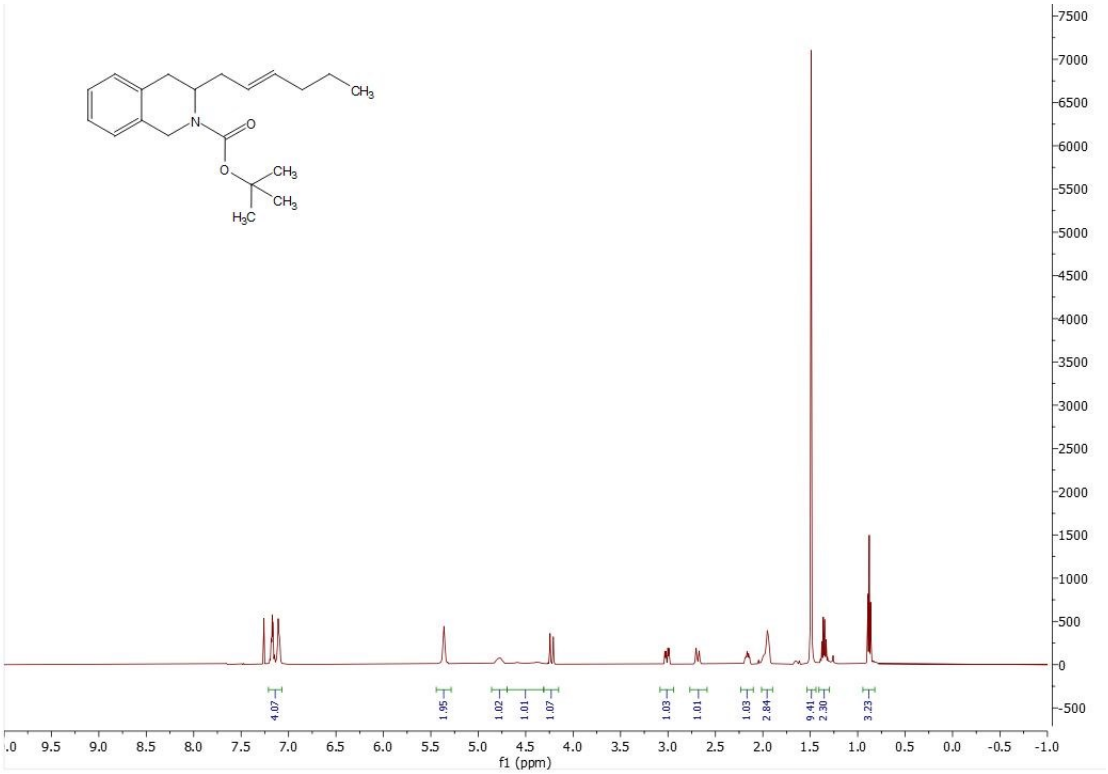
CH3
N
O
H3C
4.07
1.95
1.02
1.01
1.07
1.03
1.01
1.03
2.84
9.41
2.30
3.23
f1 (ppm)

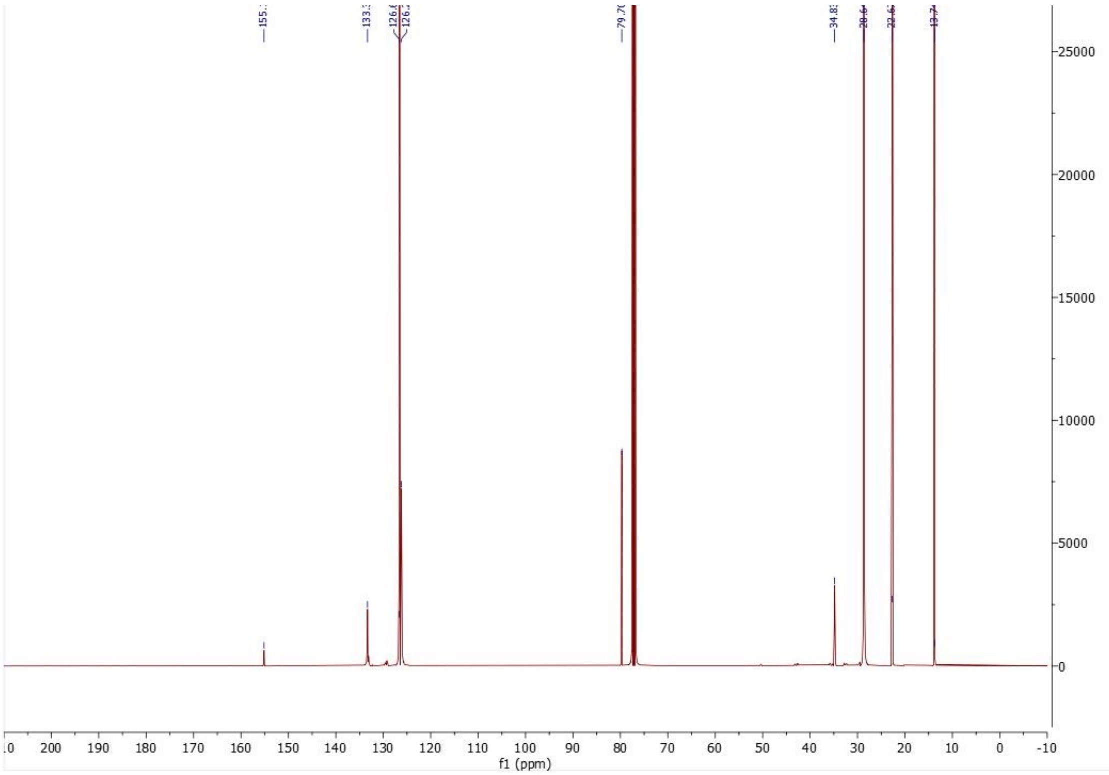
f1 (ppm)

(4g)

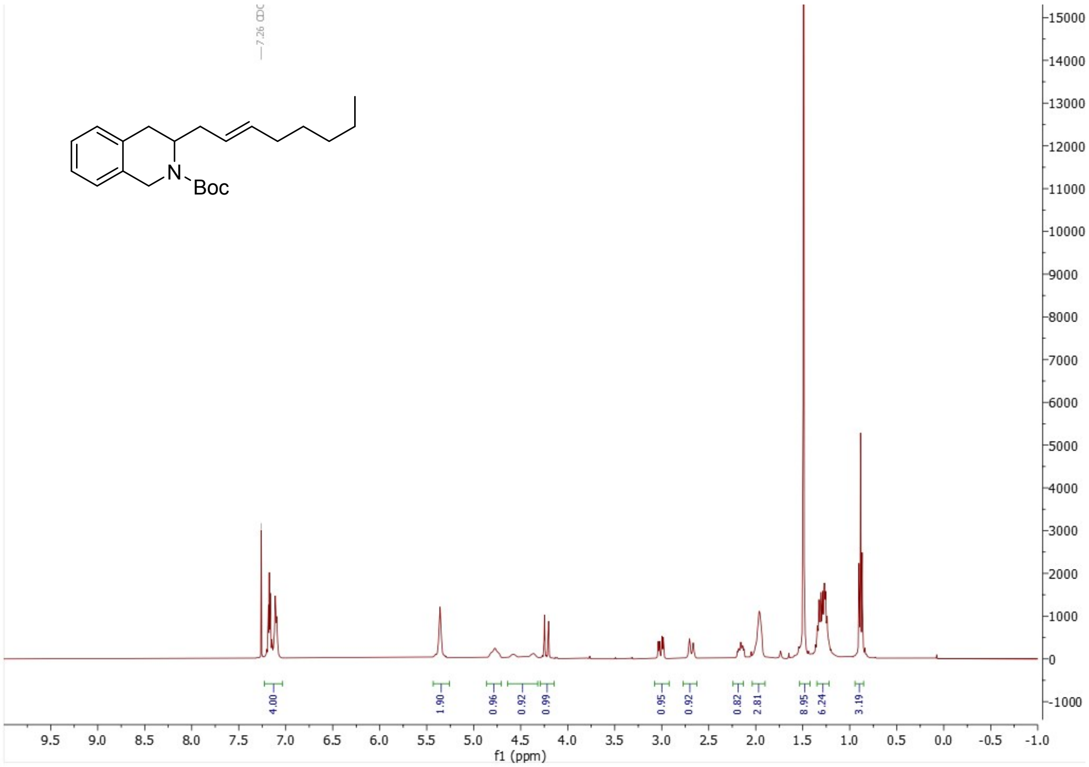

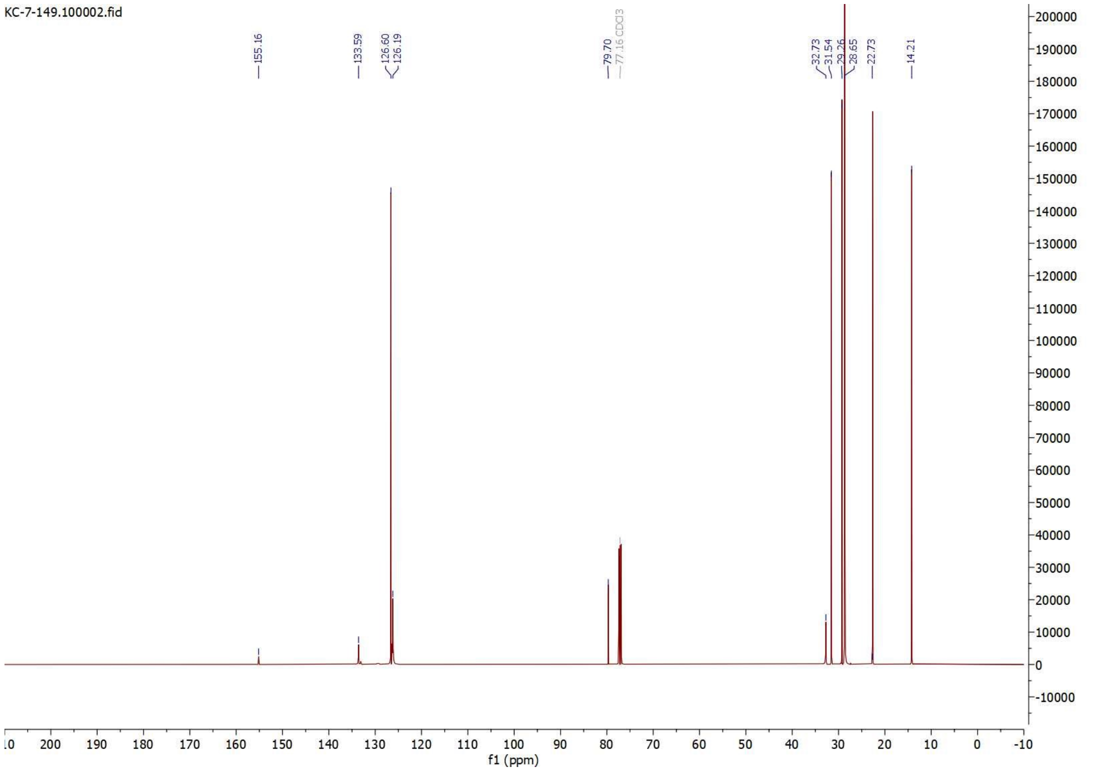

(4h)

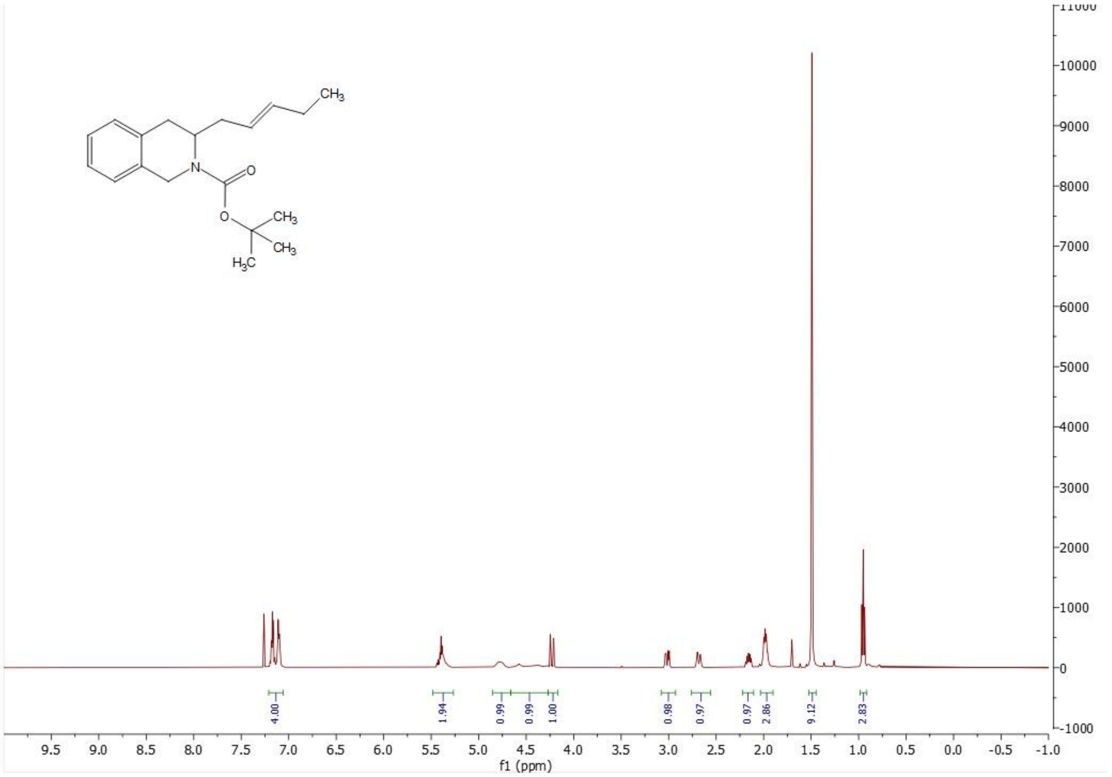

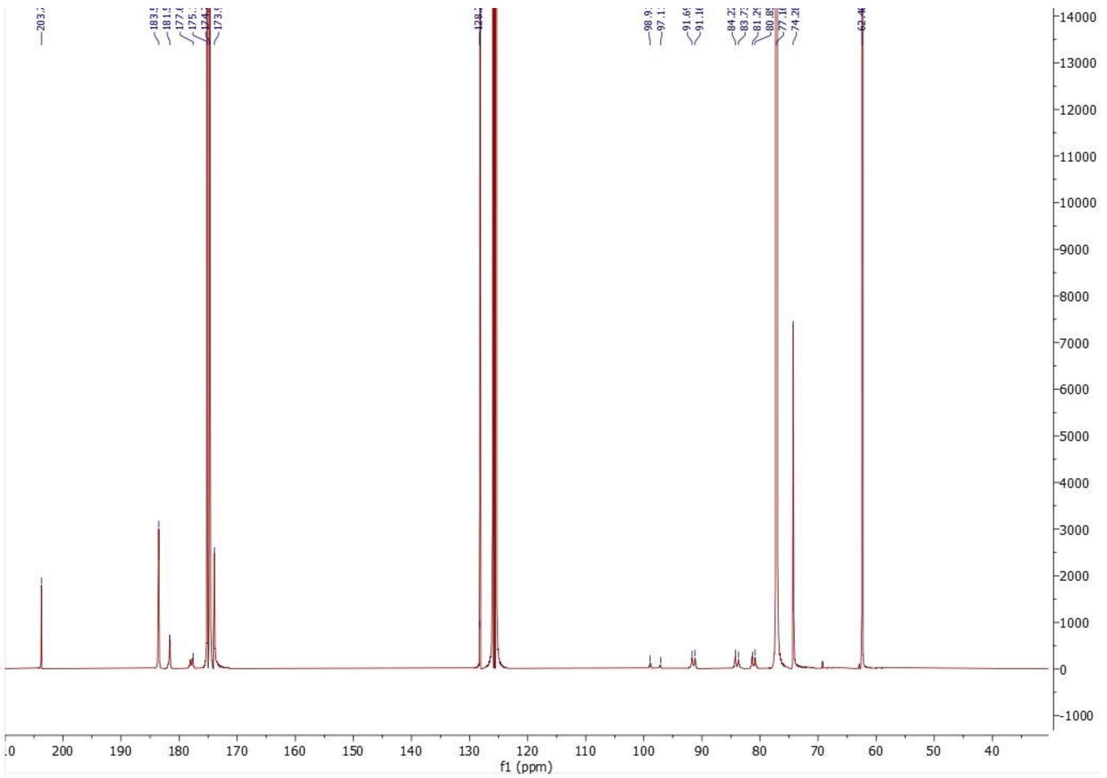

(4i) (mix of rotamers)

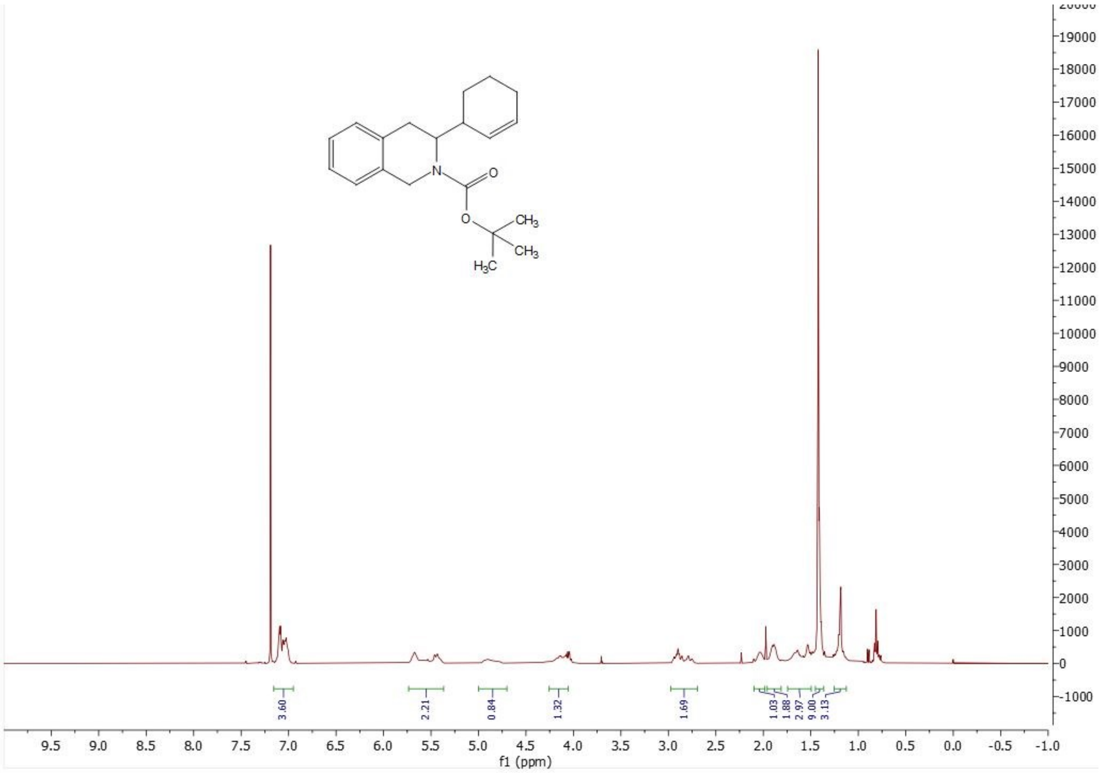

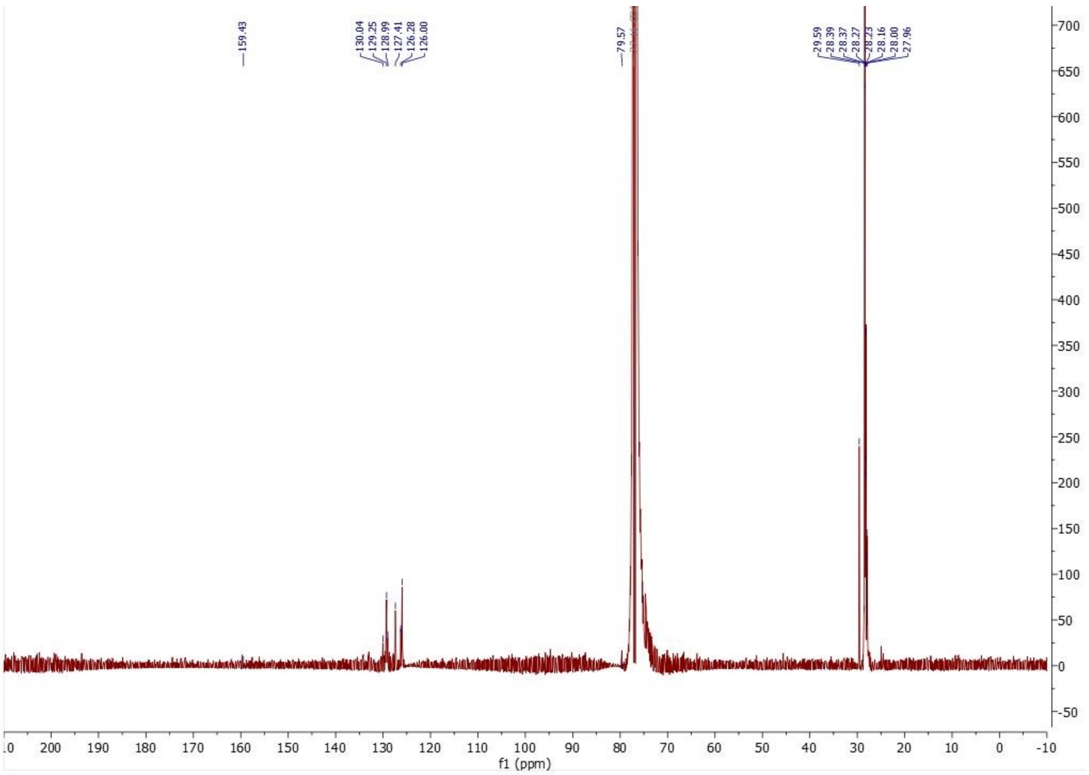

(4j) (mix of rotamers)

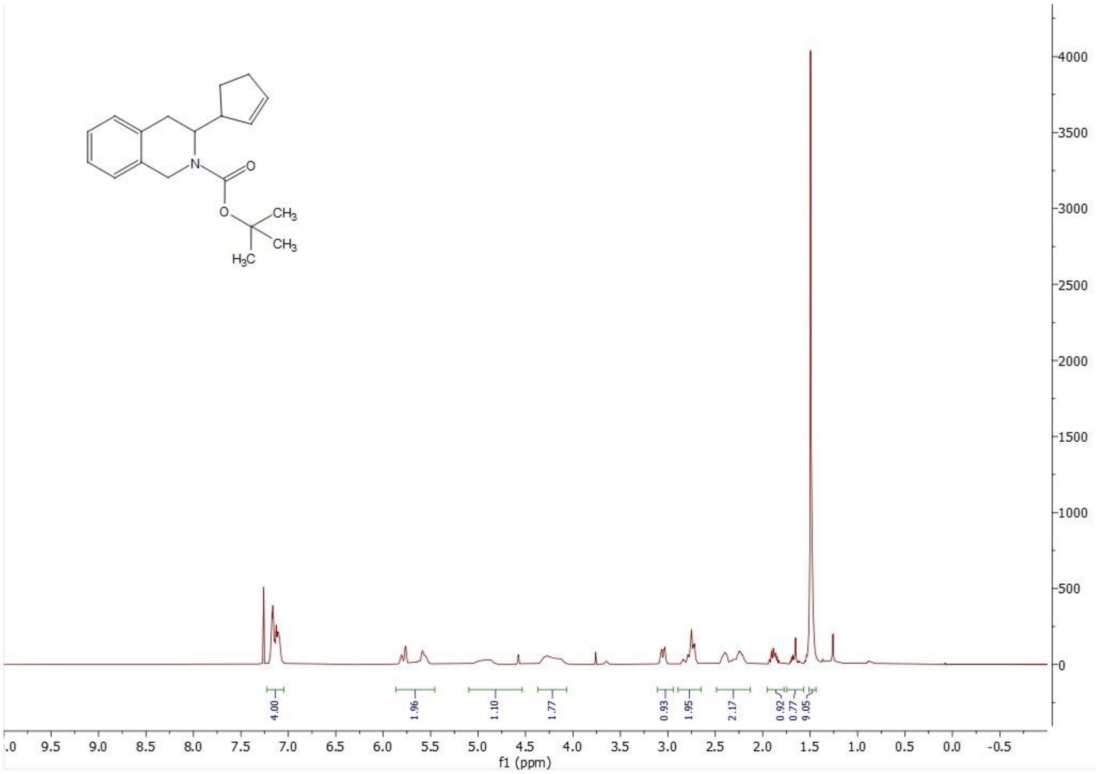
N
O
CH$_3$
H$_3$C
f1 (ppm)

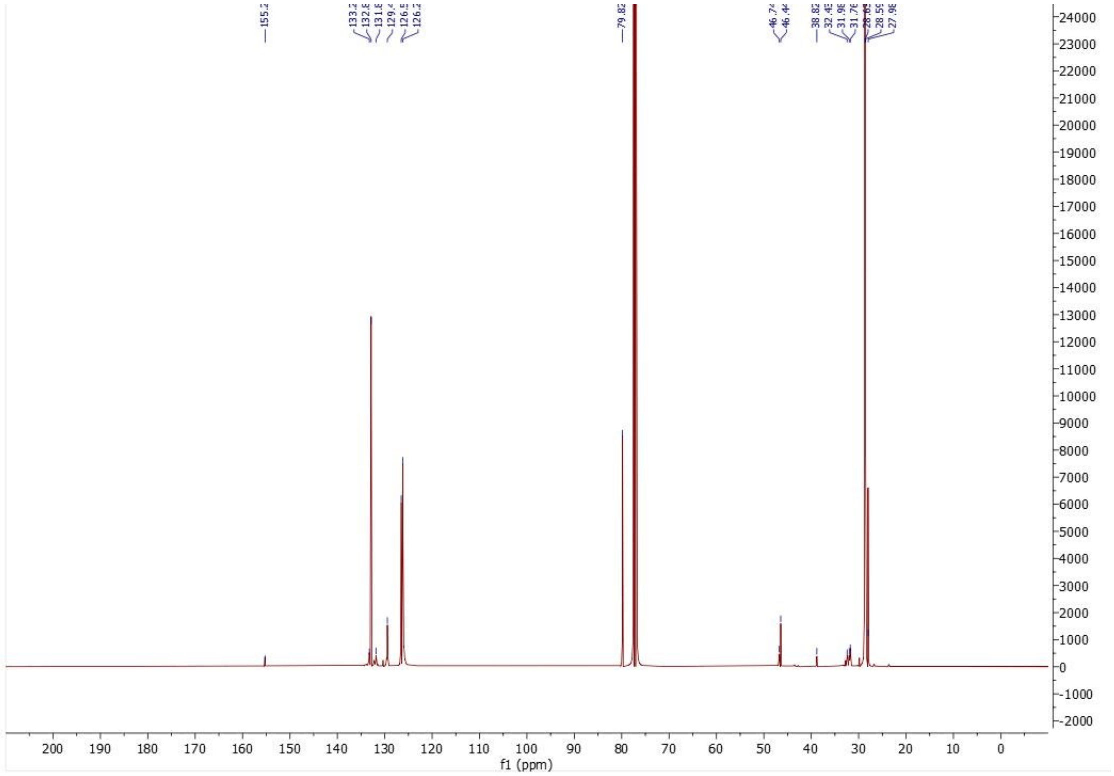
f1 (ppm)

(4k) (75:25 E:Z, mix of rotamers)

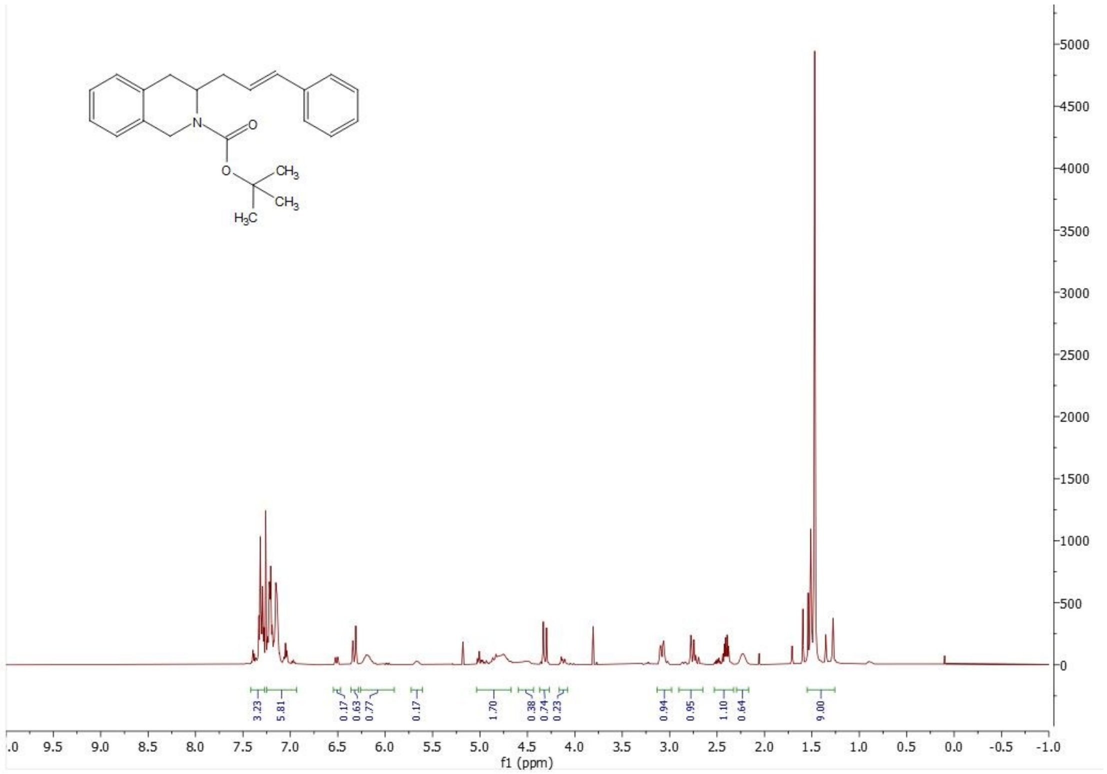

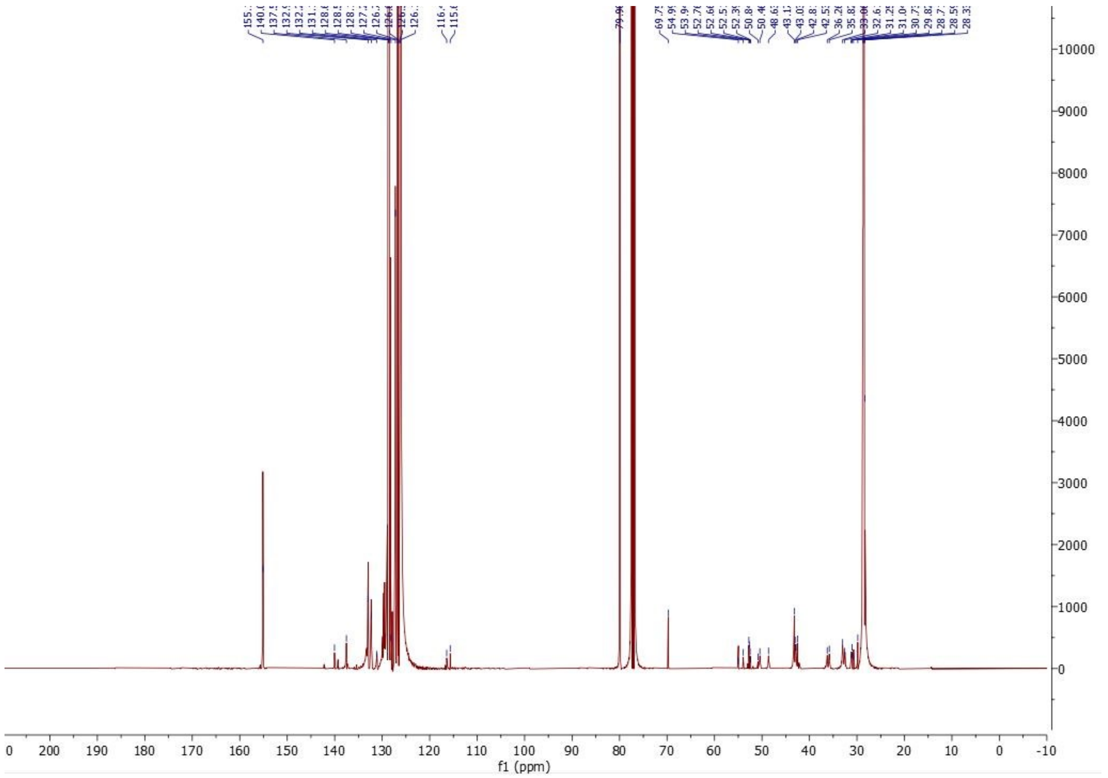

(4l) (68:32 E:Z, mix of rotamers)

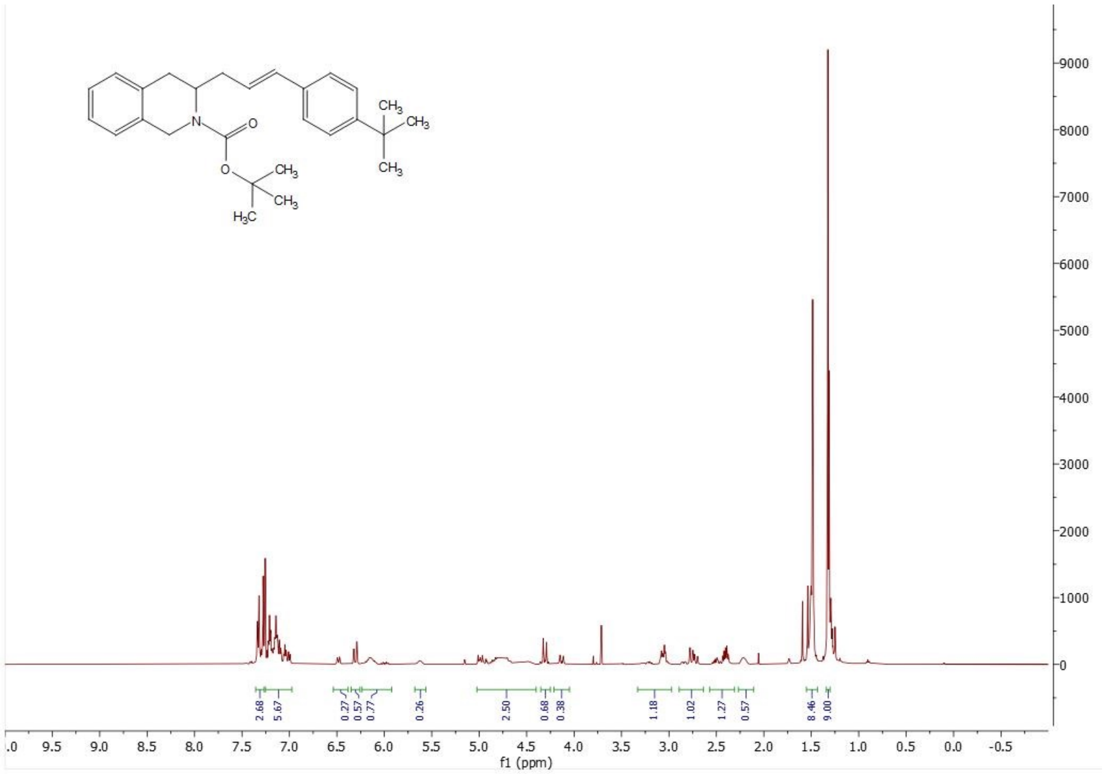

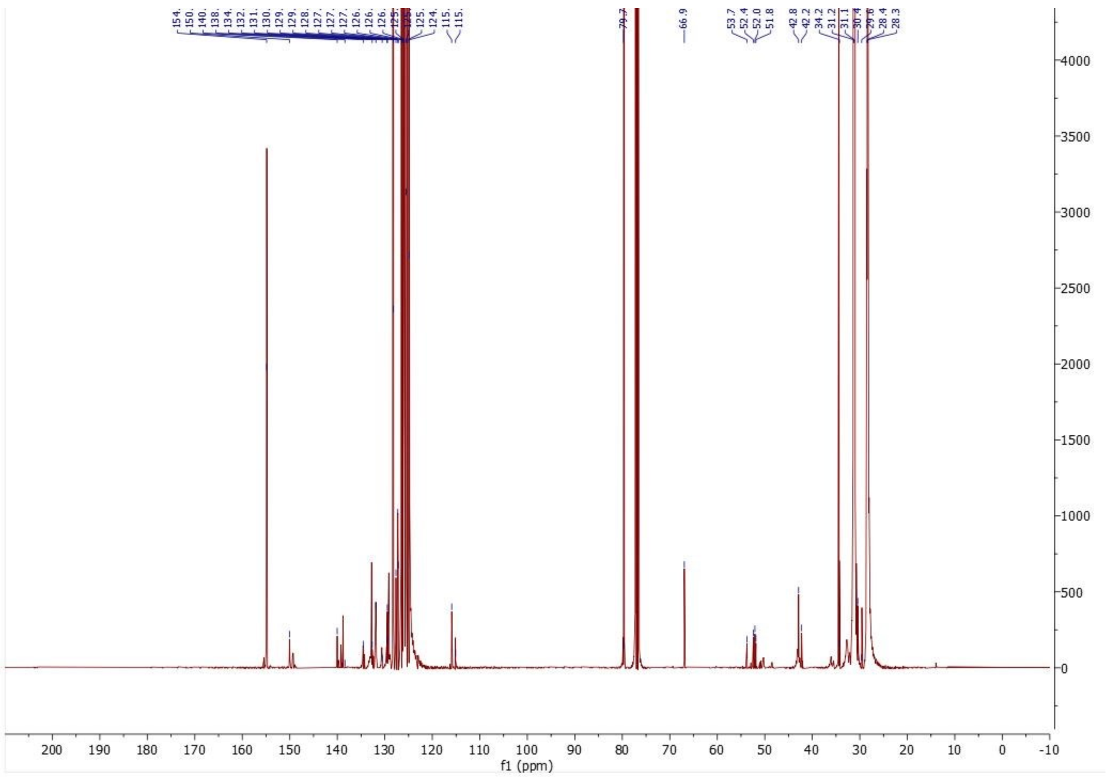

(4m) (91:9 E:Z, mix of rotamers)

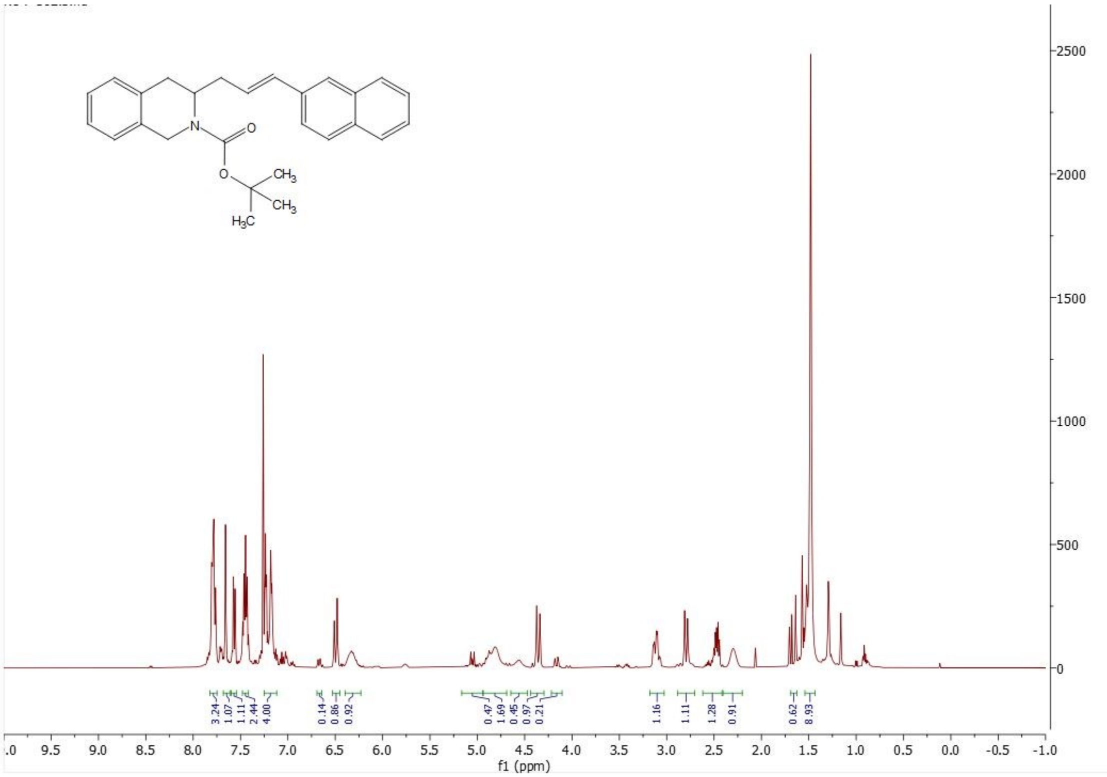

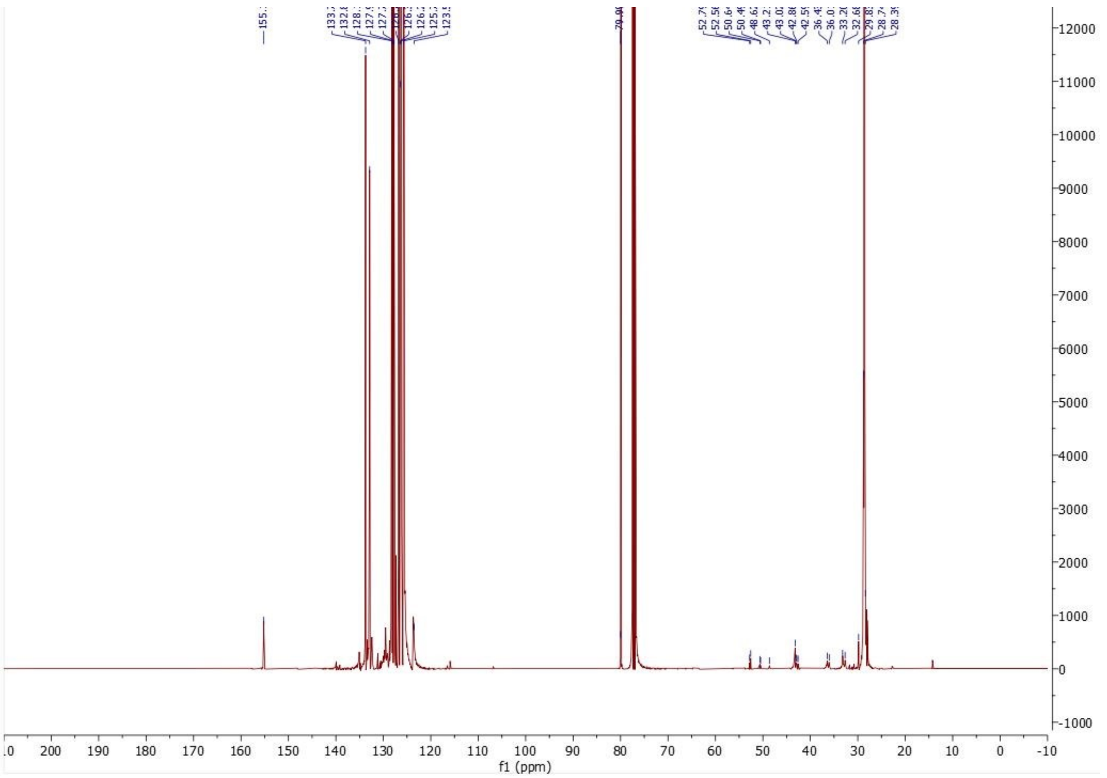

(4n) (76:24 E:Z, mix of rotamers)

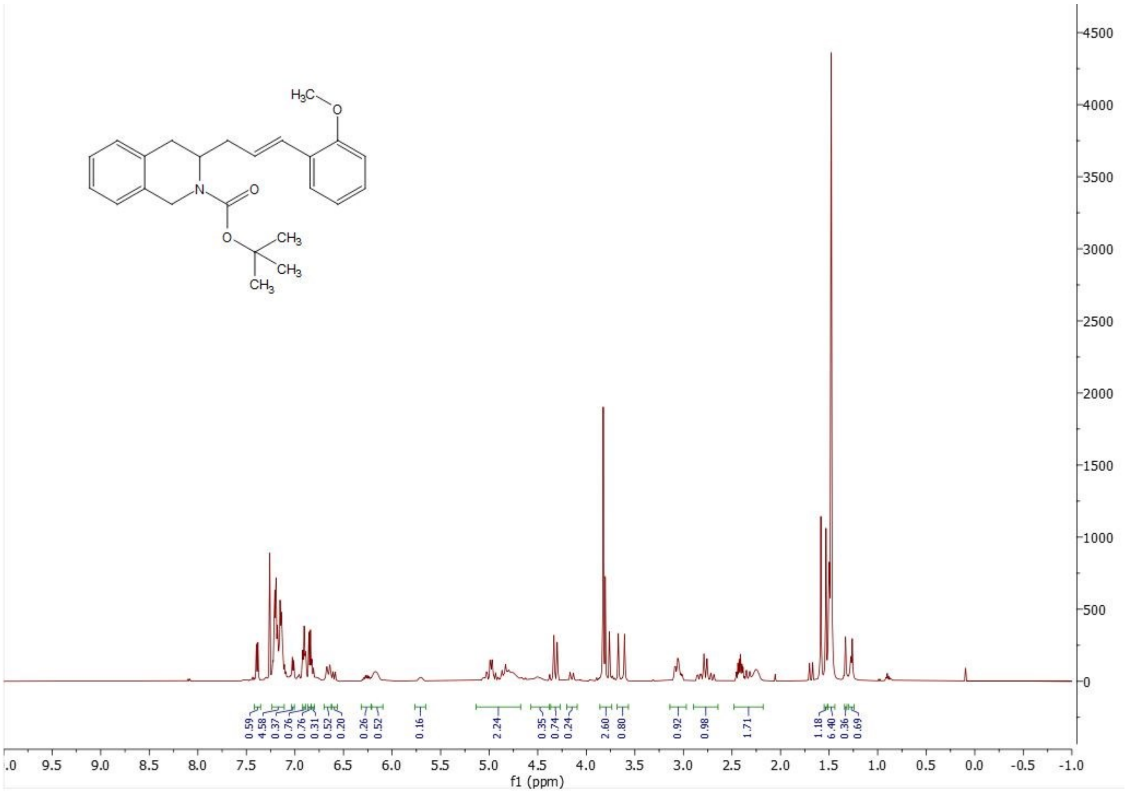
H3C
O
N
O
O
CH3
CH3
CH3
0.59 4.58 0.37 0.76 0.76 0.31 0.52 0.20 0.26 0.52 0.16 2.24 0.35 0.74 0.24 2.60 0.80 0.92 0.98 1.71 1.18 6.40 0.36 0.69
f1 (ppm)

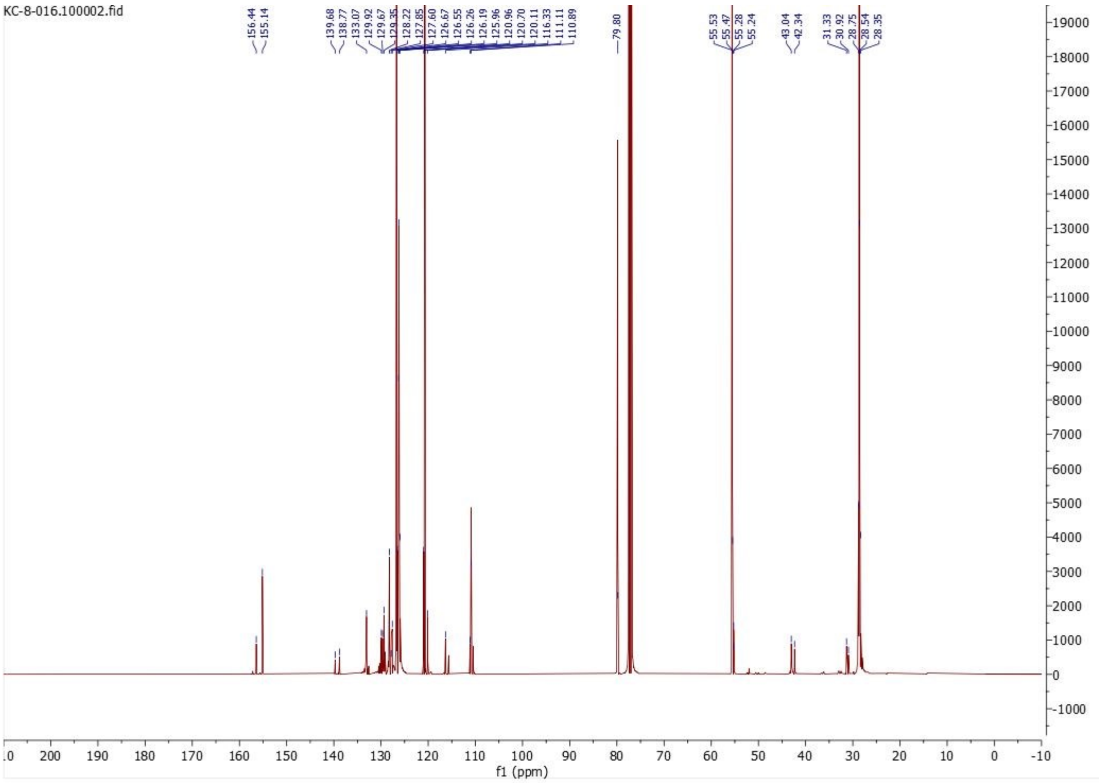
KC-8-016.100002.fid
156.44 155.14
139.68 138.77 133.07 129.92 129.67 129.35 128.22 127.85 127.60 126.67 126.55 126.26 126.19 125.96 120.96 120.70 120.11 116.33 111.11 110.89
79.80
55.53 55.47 55.28 55.24
43.04 42.34
31.33 30.92 28.75 28.54 28.35
f1 (ppm)

(4o) (>95:5 E:Z, mix of rotamers)

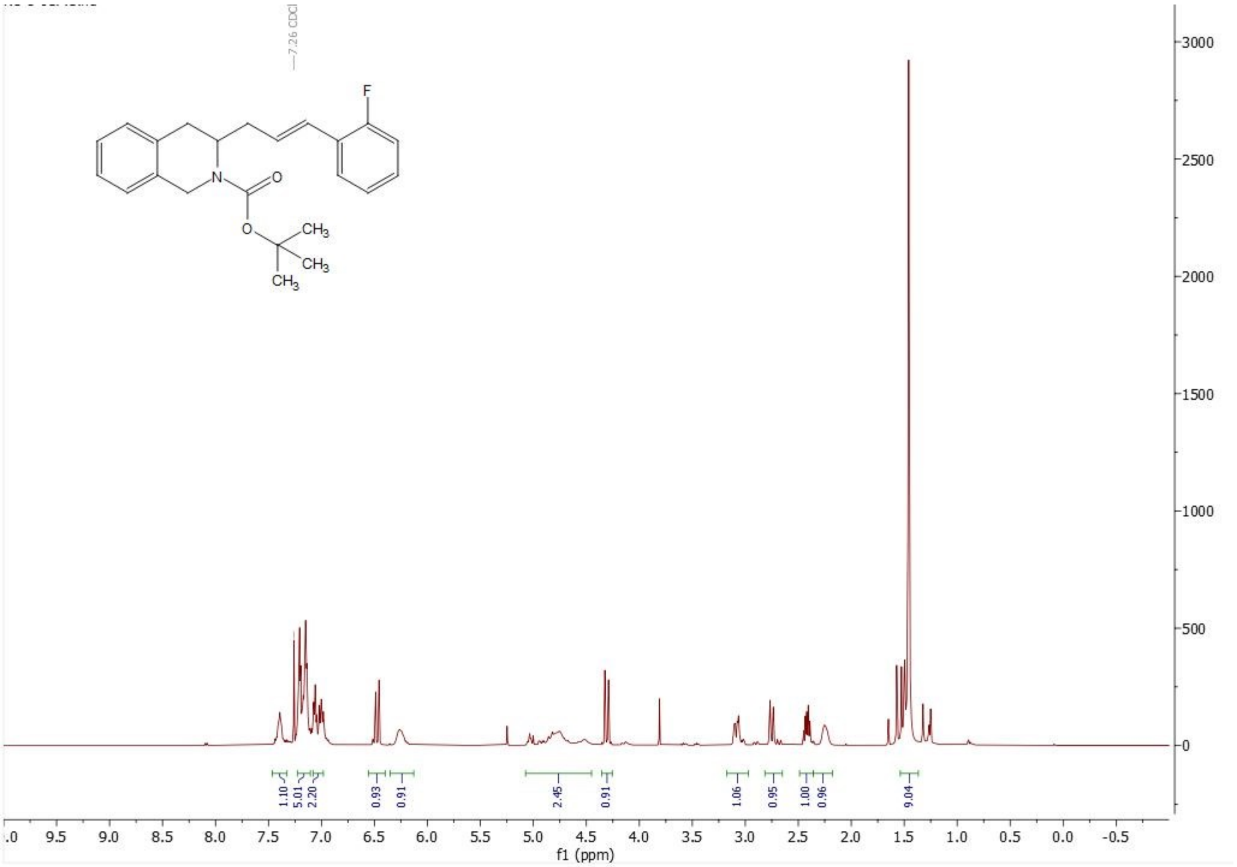

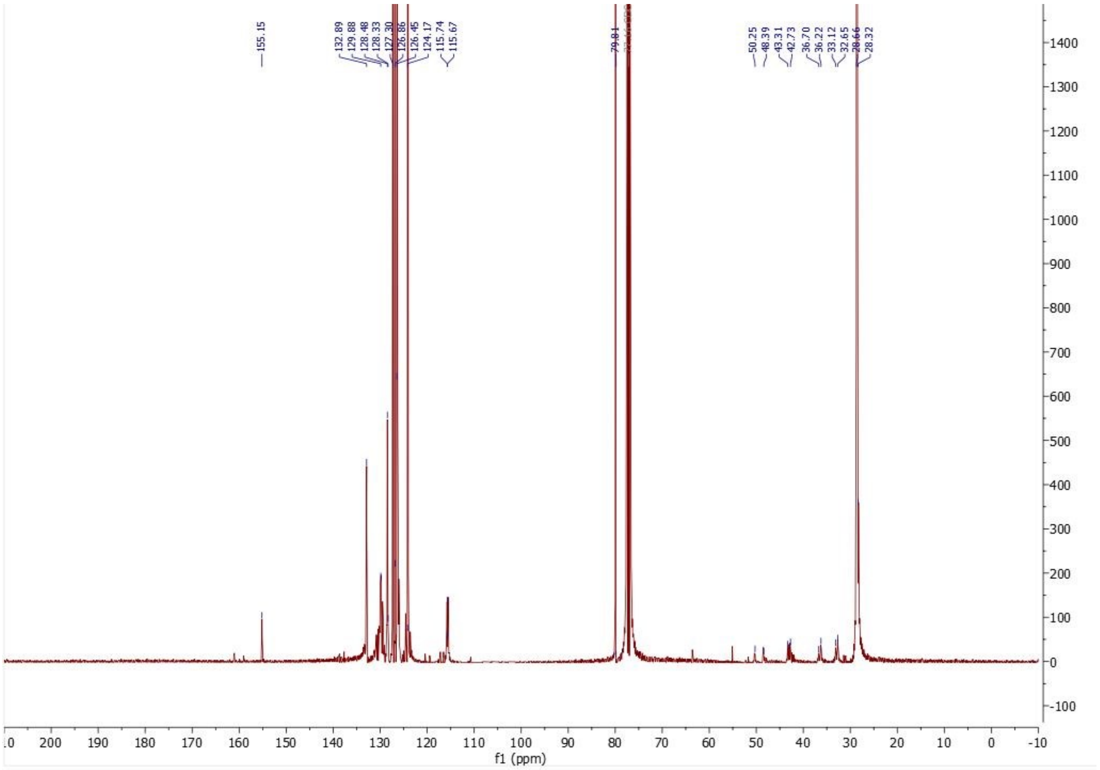

(4p) (67:33 E:Z, mix of rotamers)

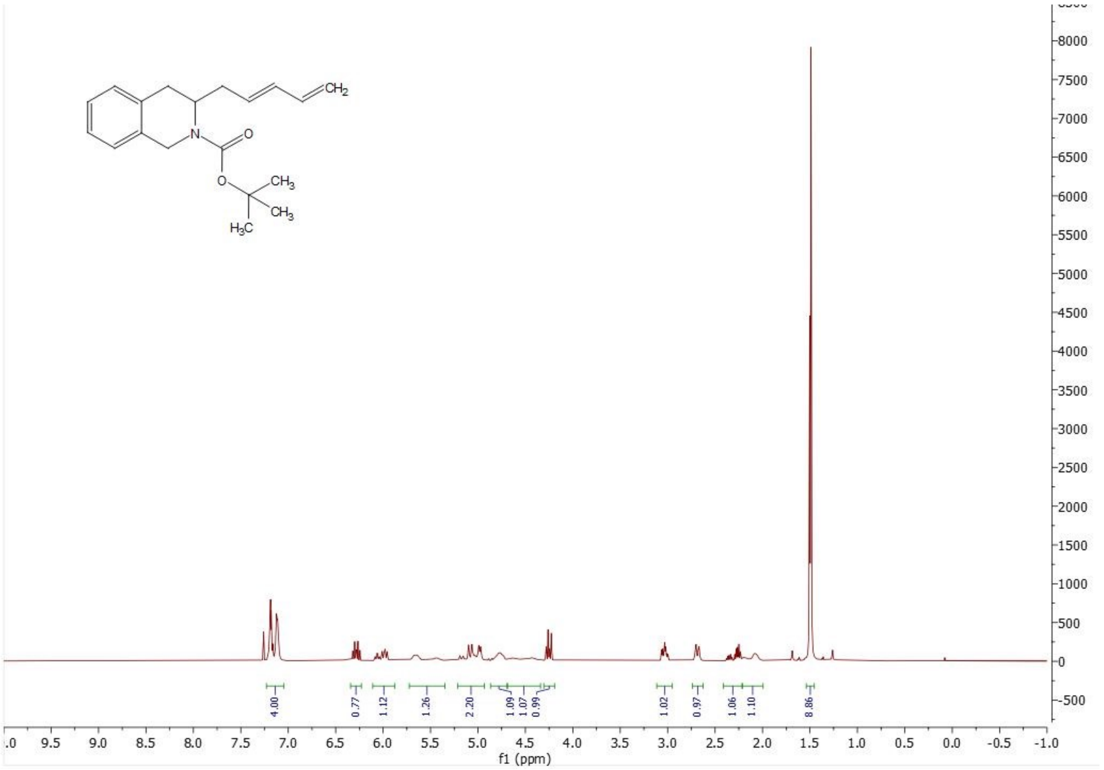
CH2
N
O
O
CH3
CH3
H3C
4.00
0.77
1.12
1.26
2.20
1.09
1.07
0.99
1.02
0.97
1.06
1.10
8.86
f1 (ppm)

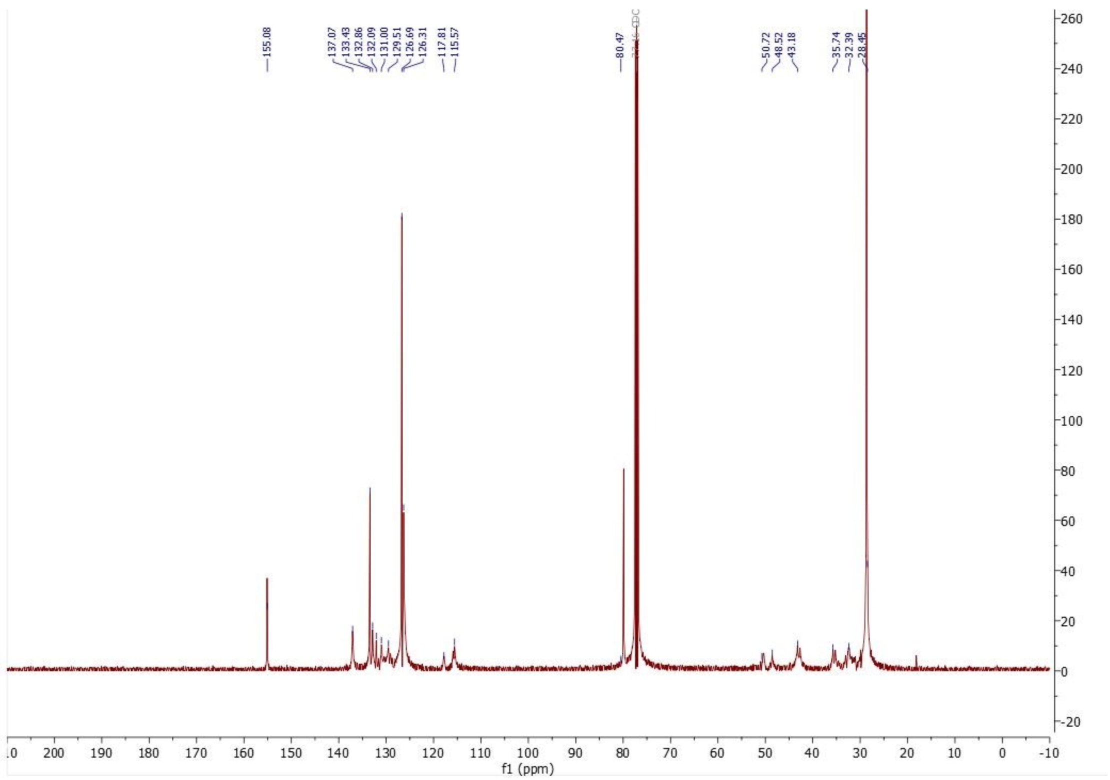
155.08
137.07
133.43
132.86
132.09
131.00
129.51
126.69
126.31
117.81
115.57
80.47
50.72
48.52
43.18
35.74
32.39
28.45
f1 (ppm)

(4q) (mix of E/Z isomers and rotamers)

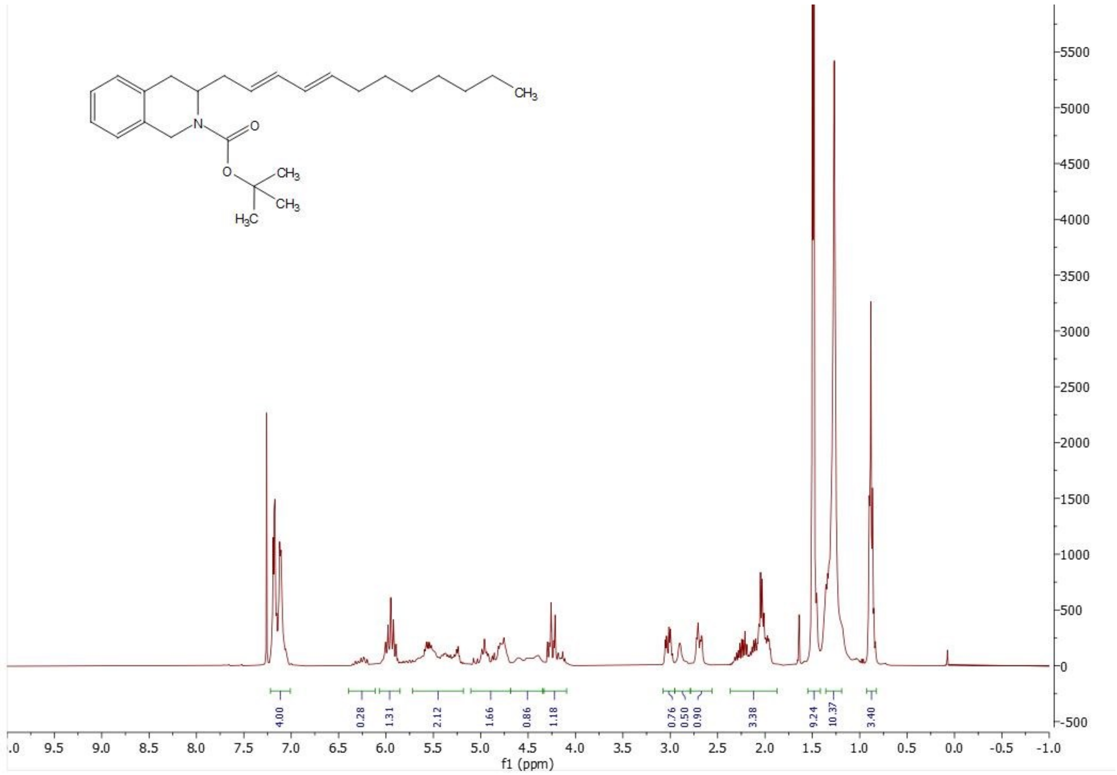

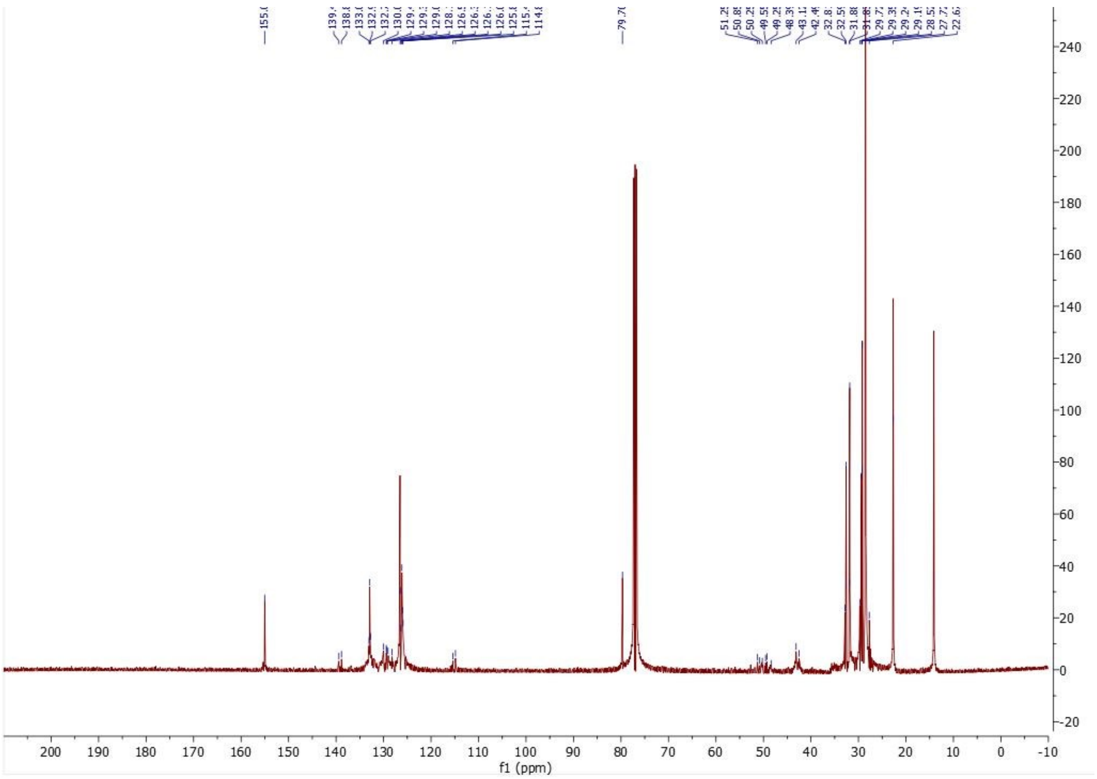

(4r) (mix of rotamers)

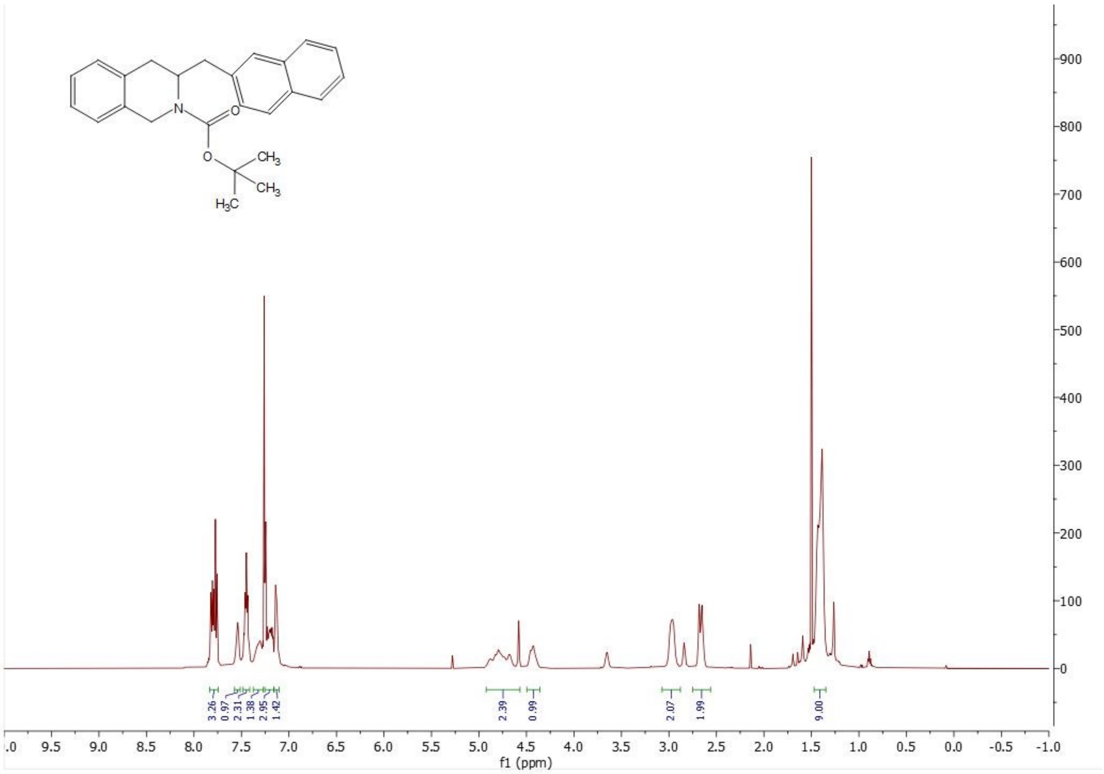

N
O
O
CH_3
CH_3
H_3C
3.26
0.97
2.31
1.38
2.95
1.42
2.39
0.99
2.07
1.99
9.00
f1 (ppm)

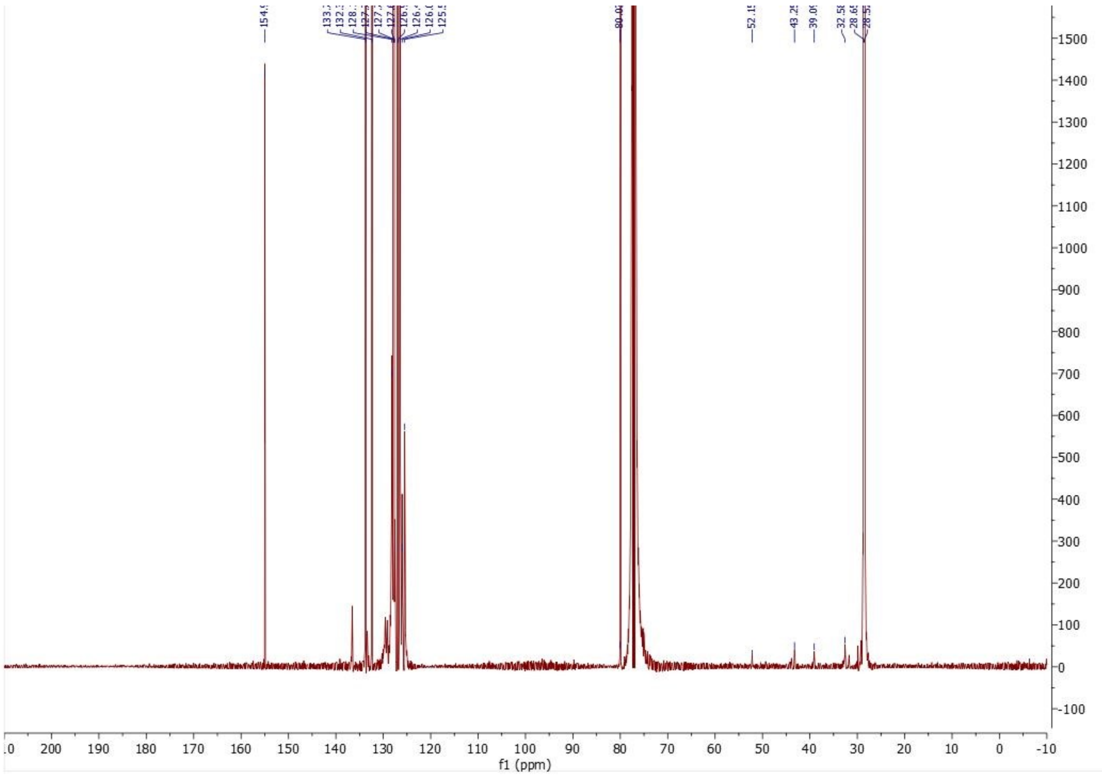

f1 (ppm)

(4s) (mix of rotamers)

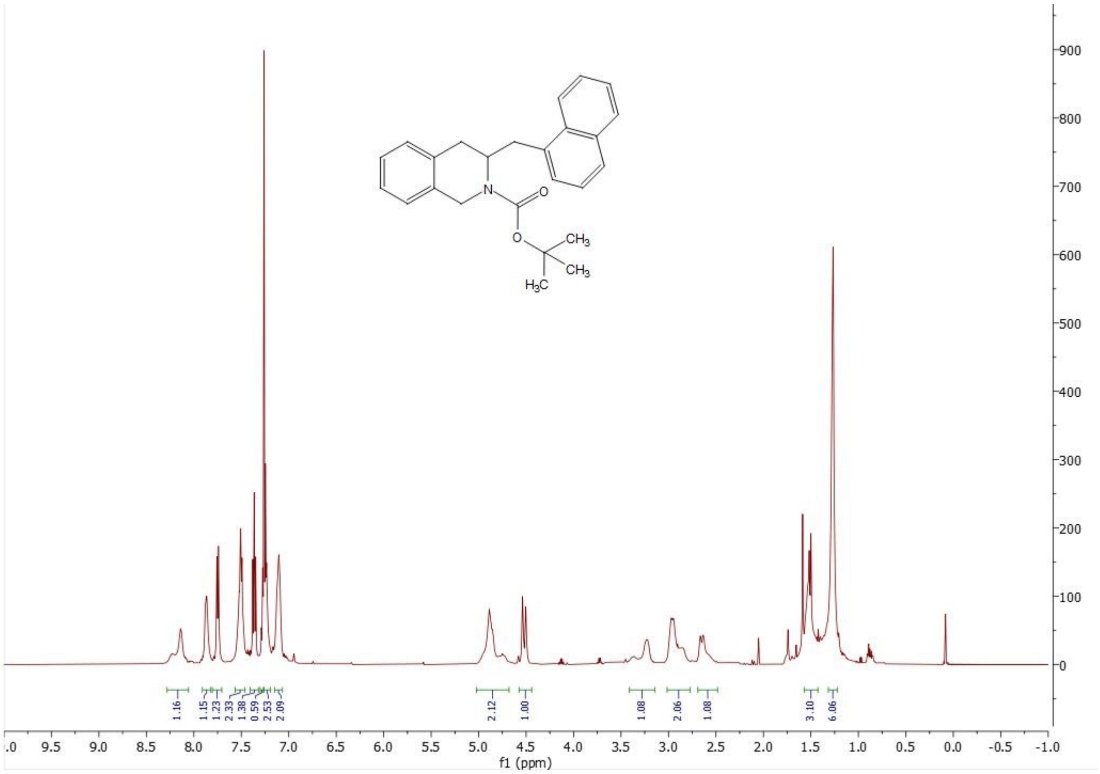

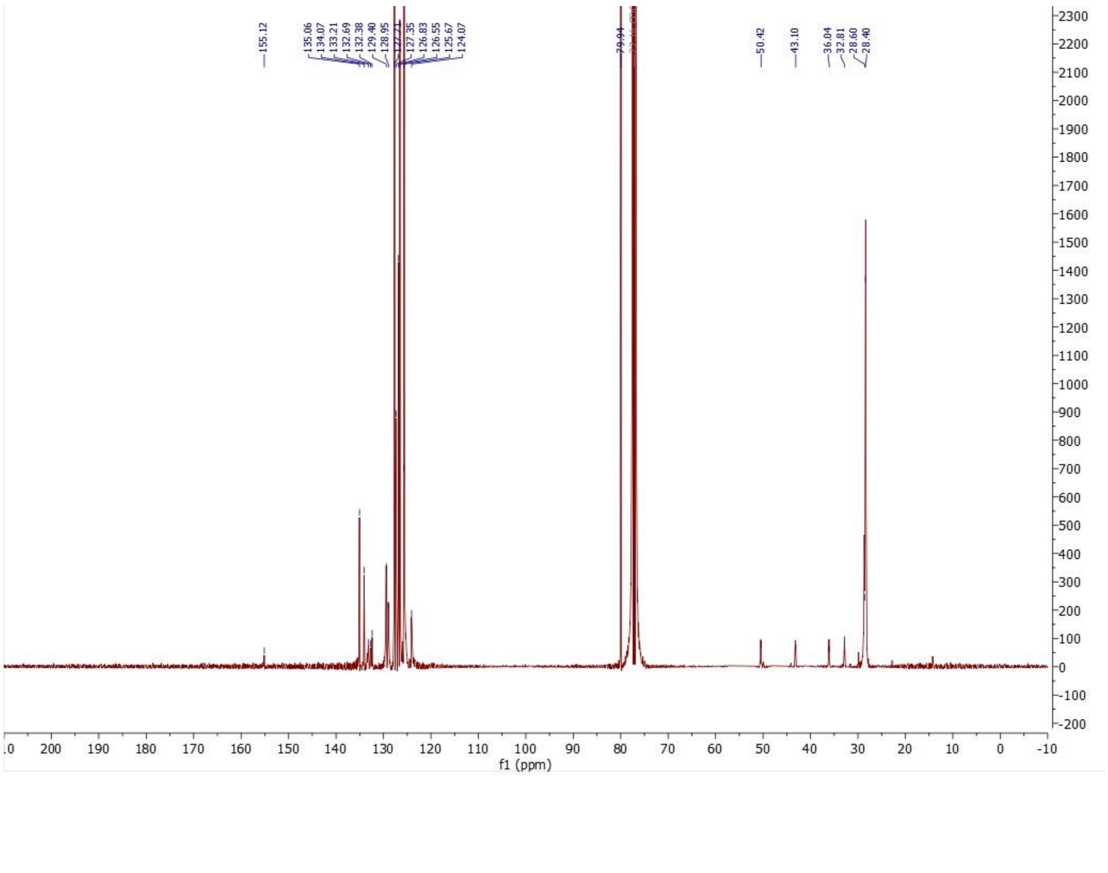

(4t) (mix of rotamers)

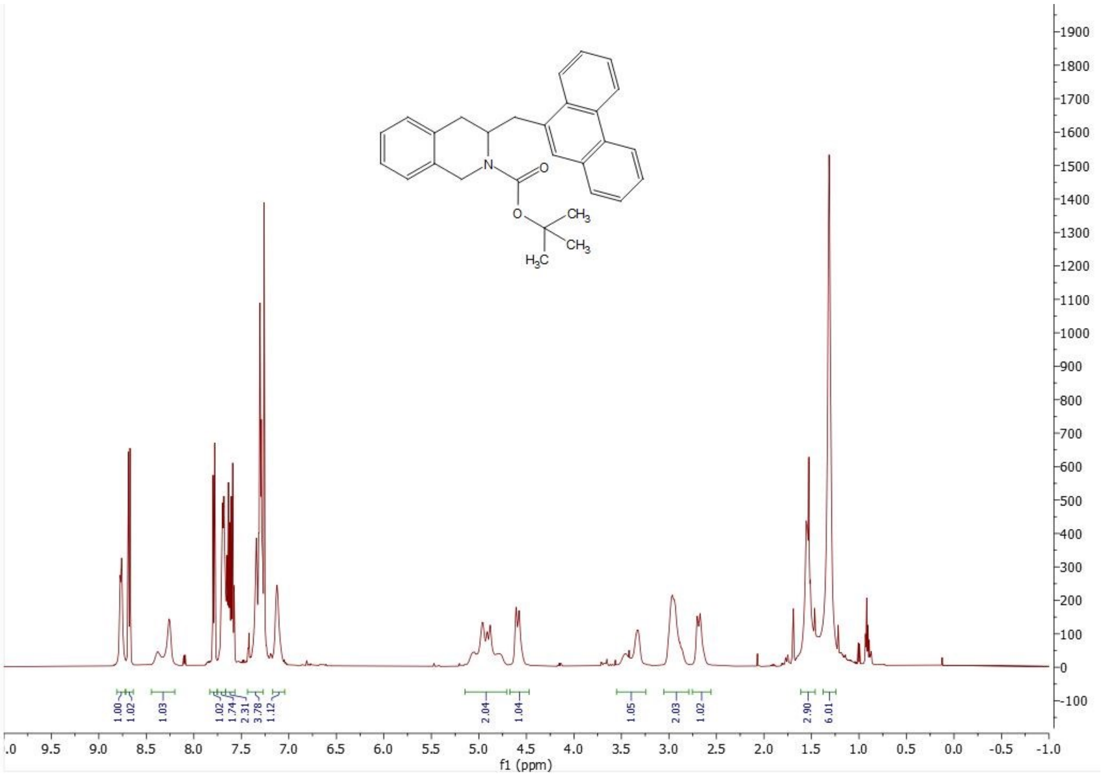
CH3
CH3
H3C
O
N
O
1.00
1.02
1.03
1.02
1.74
2.31
3.78
1.12
2.04
1.04
1.05
2.03
1.02
2.90
6.01
f1 (ppm)

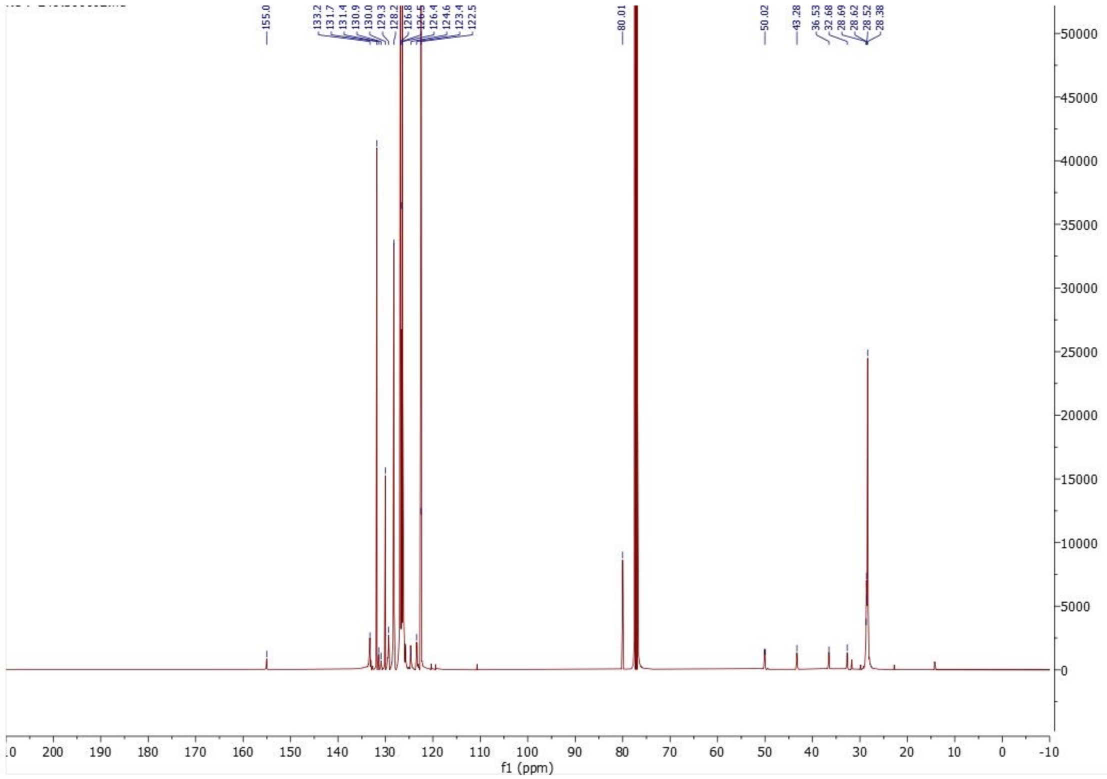
155.0
133.2
131.7
131.4
130.9
130.0
129.3
128.2
126.8
126.5
126.4
124.6
123.4
122.5
80.01
50.02
43.28
36.53
32.68
28.69
28.62
28.52
28.38
f1 (ppm)

(4u) (mix of rotamers)

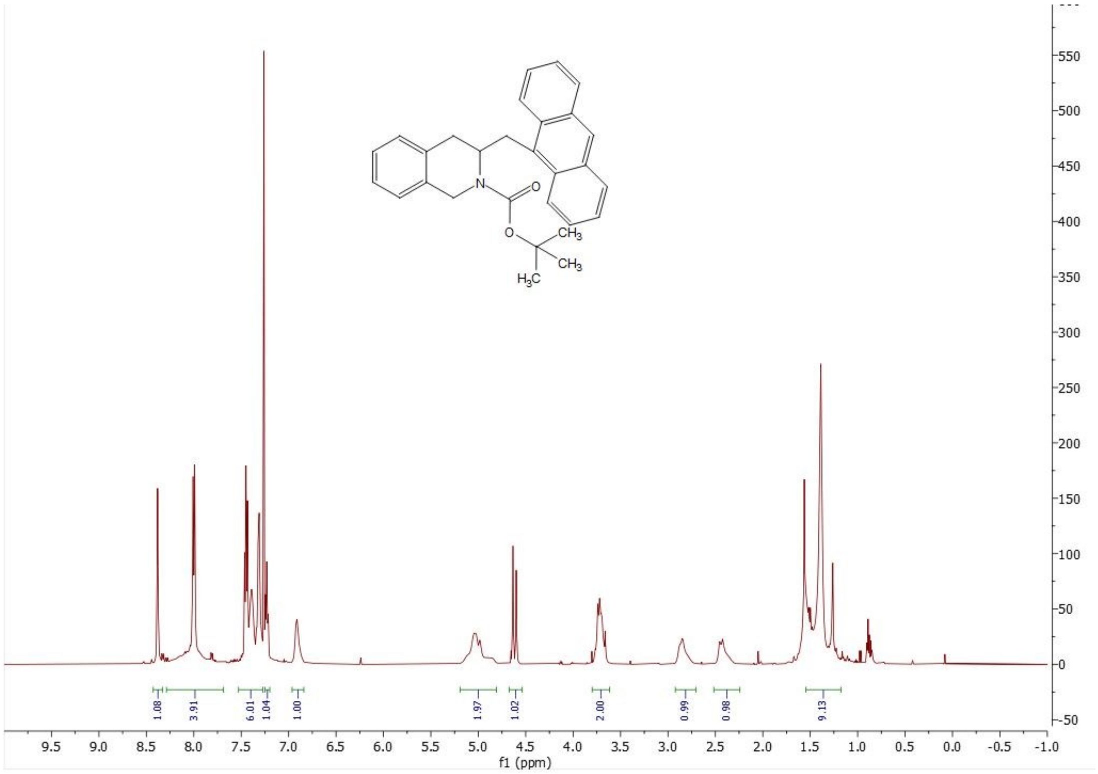

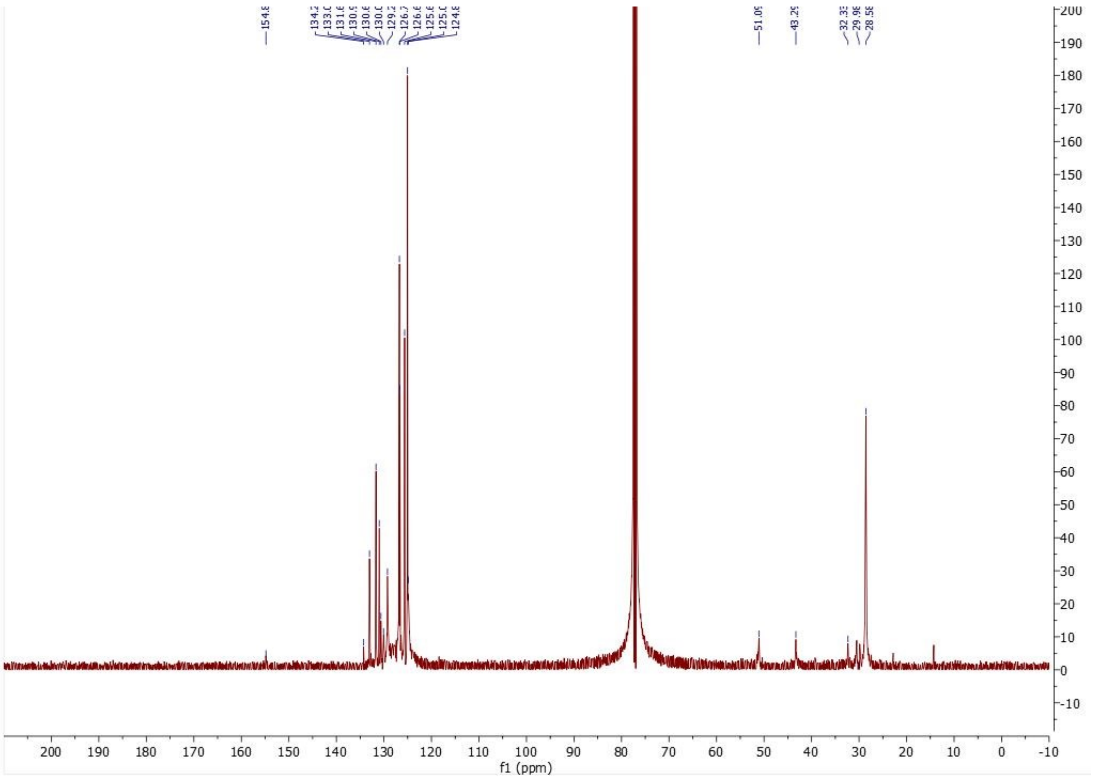

(4v) (mix of rotamers)

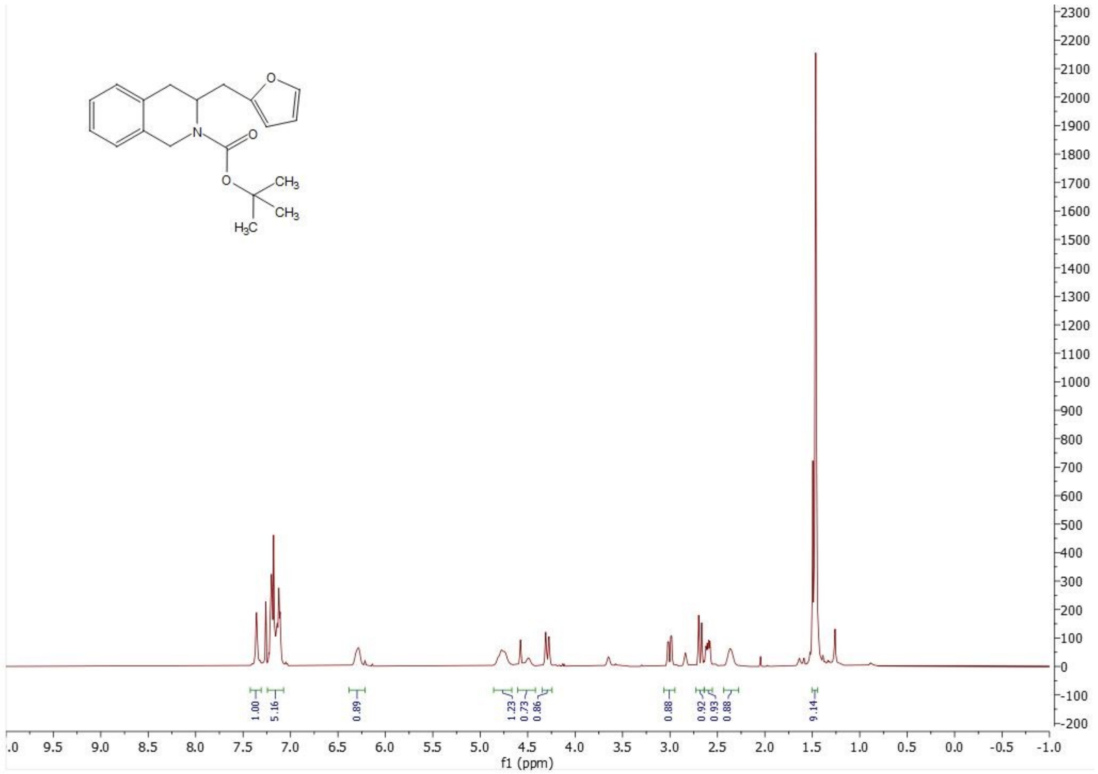

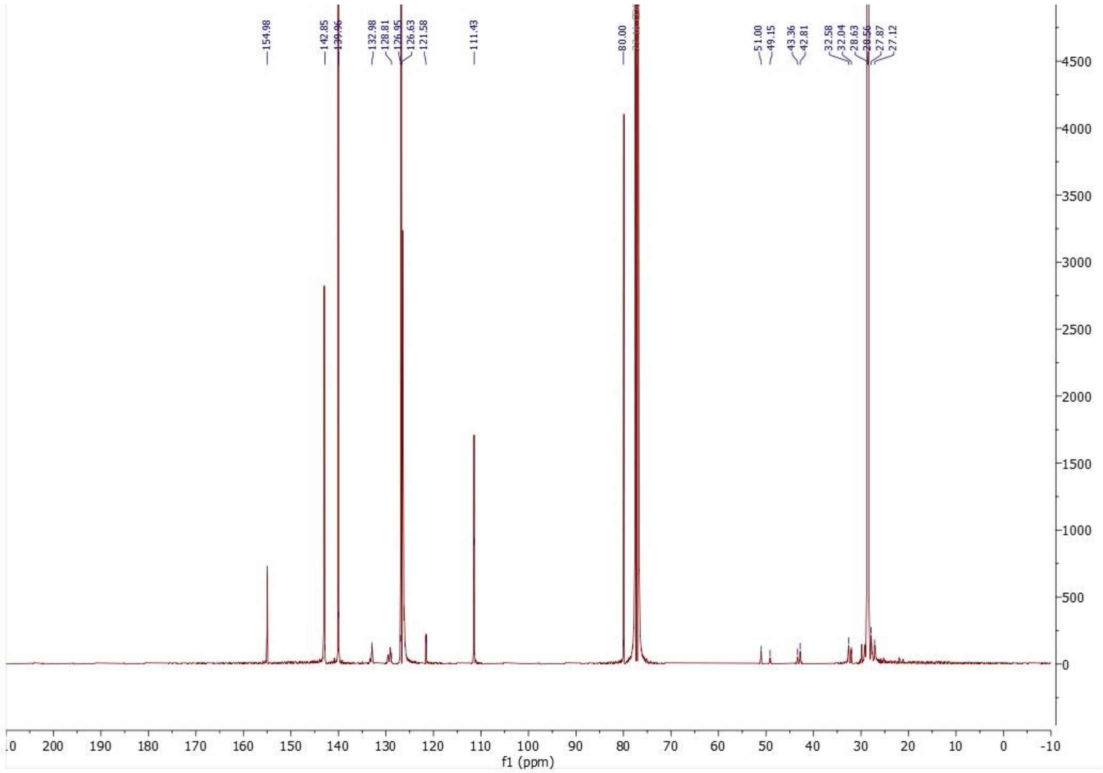

(4w) (mix of rotamers)

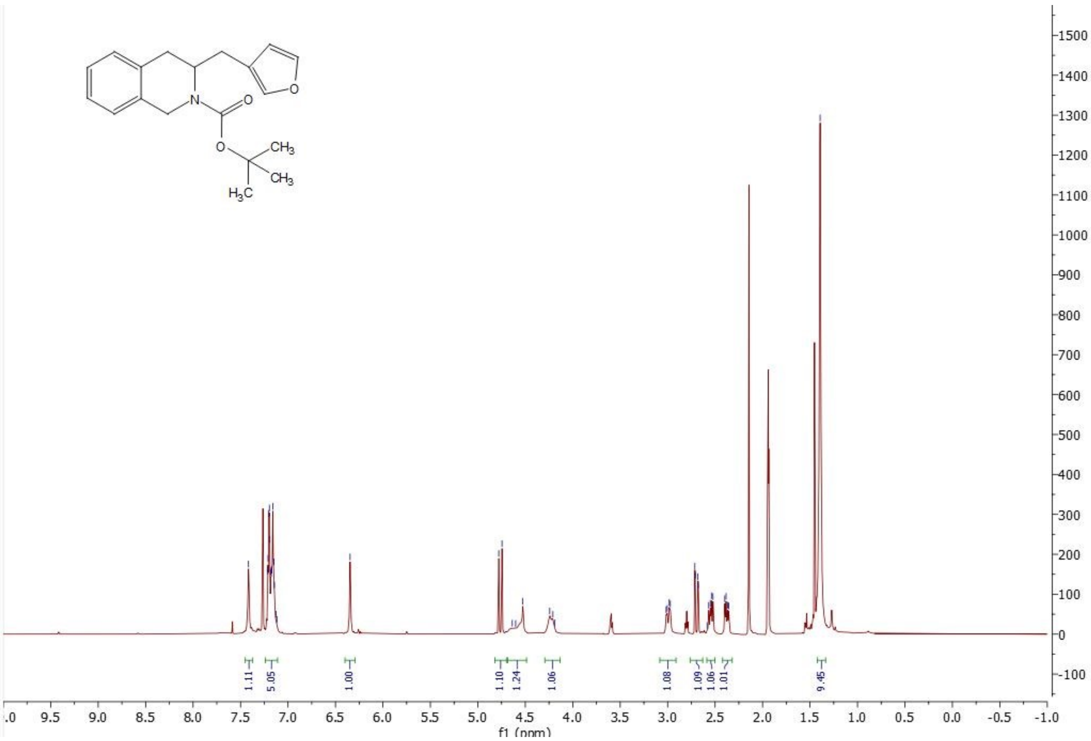

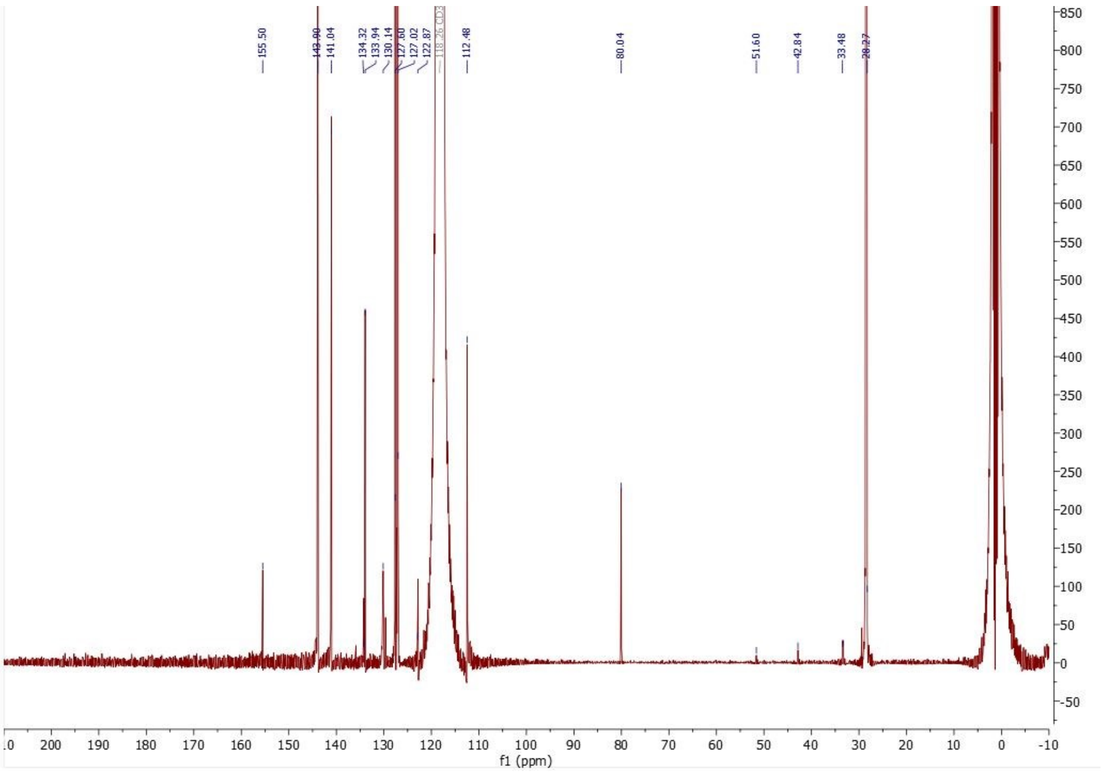

(4x) (mix of rotamers)

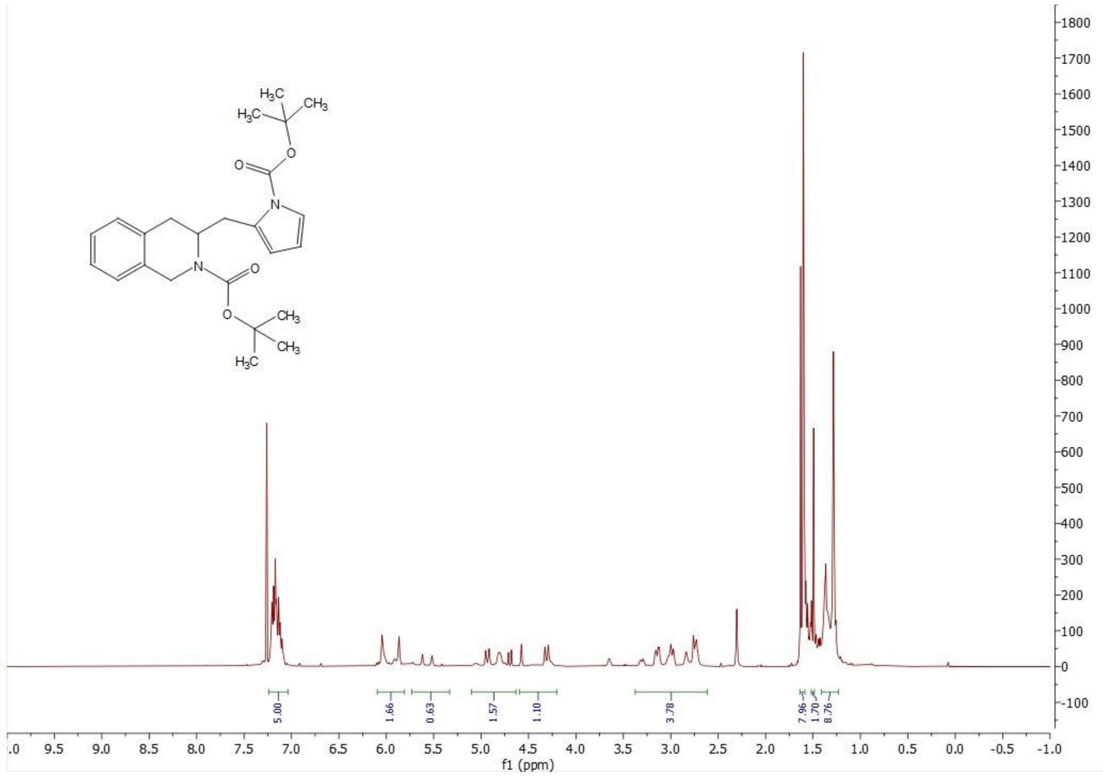

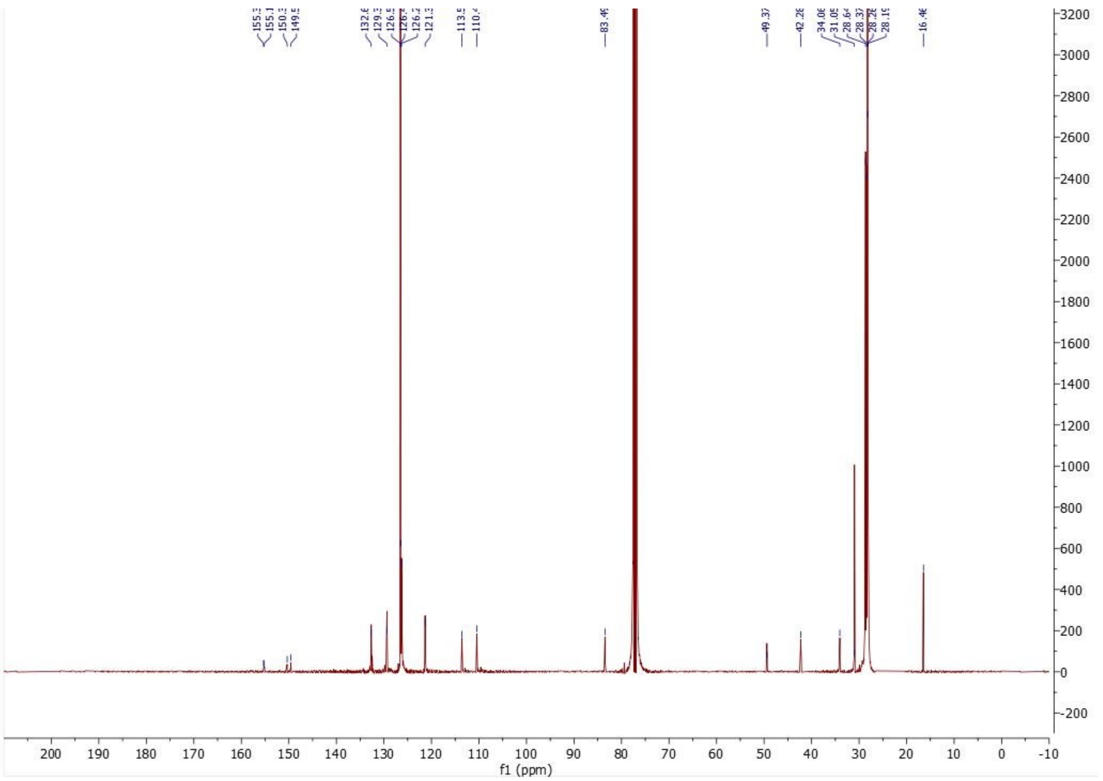

Allyl-4CzIPN (from Scheme 4)

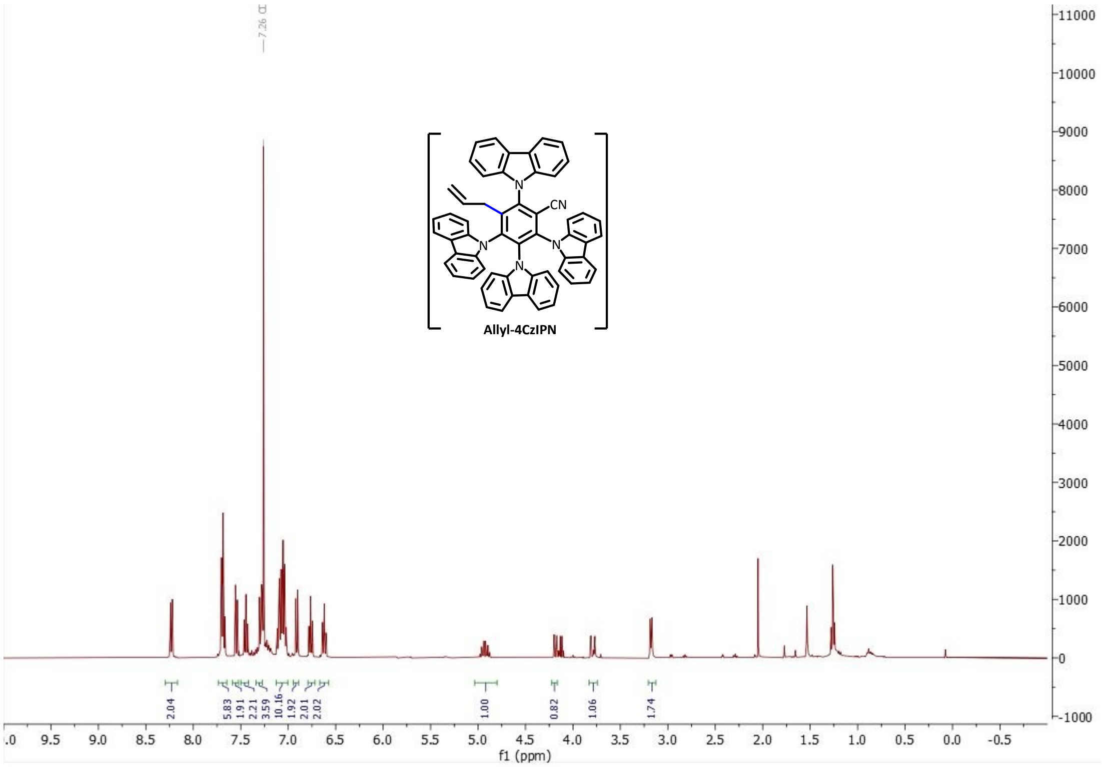

7.26 CDCl3
CN
N
Allyl-4CzIPN
2.04
5.83
1.91
2.21
3.59
10.16
1.92
2.01
2.02
1.00
0.82
1.06
1.74
f1 (ppm)
